# Evolution of the Correlated Genomic Variation Landscape Across a Divergence Continuum in the Genus *Castanopsis*

**DOI:** 10.1093/molbev/msae191

**Published:** 2024-09-09

**Authors:** Xue-Yan Chen, Biao-Feng Zhou, Yong Shi, Hui Liu, Yi-Ye Liang, Pär K Ingvarsson, Baosheng Wang

**Affiliations:** Guangdong Provincial Key Laboratory of Applied Botany, South China Botanical Garden, Chinese Academy of Sciences, Guangzhou, China; State Key Laboratory of Plant Diversity and Specialty Crops & Key Laboratory of National Forestry and Grassland Administration on Plant Conservation and Utilization in Southern China, South China Botanical Garden, Chinese Academy of Sciences, Guangzhou, China; South China National Botanical Garden, Guangzhou, China; University of the Chinese Academy of Sciences, Beijing, China; Guangdong Provincial Key Laboratory of Applied Botany, South China Botanical Garden, Chinese Academy of Sciences, Guangzhou, China; State Key Laboratory of Plant Diversity and Specialty Crops & Key Laboratory of National Forestry and Grassland Administration on Plant Conservation and Utilization in Southern China, South China Botanical Garden, Chinese Academy of Sciences, Guangzhou, China; South China National Botanical Garden, Guangzhou, China; Guangdong Provincial Key Laboratory of Applied Botany, South China Botanical Garden, Chinese Academy of Sciences, Guangzhou, China; State Key Laboratory of Plant Diversity and Specialty Crops & Key Laboratory of National Forestry and Grassland Administration on Plant Conservation and Utilization in Southern China, South China Botanical Garden, Chinese Academy of Sciences, Guangzhou, China; South China National Botanical Garden, Guangzhou, China; Guangdong Provincial Key Laboratory of Applied Botany, South China Botanical Garden, Chinese Academy of Sciences, Guangzhou, China; State Key Laboratory of Plant Diversity and Specialty Crops & Key Laboratory of National Forestry and Grassland Administration on Plant Conservation and Utilization in Southern China, South China Botanical Garden, Chinese Academy of Sciences, Guangzhou, China; South China National Botanical Garden, Guangzhou, China; Guangdong Provincial Key Laboratory of Applied Botany, South China Botanical Garden, Chinese Academy of Sciences, Guangzhou, China; State Key Laboratory of Plant Diversity and Specialty Crops & Key Laboratory of National Forestry and Grassland Administration on Plant Conservation and Utilization in Southern China, South China Botanical Garden, Chinese Academy of Sciences, Guangzhou, China; South China National Botanical Garden, Guangzhou, China; Linnean Center for Plant Biology, Department of Plant Biology, Uppsala BioCenter, Swedish University of Agricultural Sciences, Uppsala, Sweden; Guangdong Provincial Key Laboratory of Applied Botany, South China Botanical Garden, Chinese Academy of Sciences, Guangzhou, China; State Key Laboratory of Plant Diversity and Specialty Crops & Key Laboratory of National Forestry and Grassland Administration on Plant Conservation and Utilization in Southern China, South China Botanical Garden, Chinese Academy of Sciences, Guangzhou, China; South China National Botanical Garden, Guangzhou, China

**Keywords:** genomic variation, linked selection, gene flow, recombination rate, forest trees

## Abstract

The heterogeneous landscape of genomic variation has been well documented in population genomic studies. However, disentangling the intricate interplay of evolutionary forces influencing the genetic variation landscape over time remains challenging. In this study, we assembled a chromosome-level genome for *Castanopsis eyrei* and sequenced the whole genomes of 276 individuals from 12 *Castanopsis* species, spanning a broad divergence continuum. We found highly correlated genomic variation landscapes across these species. Furthermore, variations in genetic diversity and differentiation along the genome were strongly associated with recombination rates and gene density. These results suggest that long-term linked selection and conserved genomic features have contributed to the formation of a common genomic variation landscape. By examining how correlations between population summary statistics change throughout the species divergence continuum, we determined that background selection alone does not fully explain the observed patterns of genomic variation; the effects of recurrent selective sweeps must be considered. We further revealed that extensive gene flow has significantly influenced patterns of genomic variation in *Castanopsis* species. The estimated admixture proportion correlated positively with recombination rate and negatively with gene density, supporting a scenario of selection against gene flow. Additionally, putative introgression regions exhibited strong signals of positive selection, an enrichment of functional genes, and reduced genetic burdens, indicating that adaptive introgression has played a role in shaping the genomes of hybridizing species. This study provides insights into how different evolutionary forces have interacted in driving the evolution of the genomic variation landscape.

## Introduction

Population genomic studies have unveiled a heterogeneous landscape of genomic variation, generated by various evolutionary processes, including divergent selection (Nosil et al. 2009; Feder and Nosil 2010; Malinsky et al. 2015), linked selection (Renaut et al. 2013; Cruickshank and Hahn 2014; Burri et al. 2015; Vijay et al. 2016; Liang et al. 2022), and sorting of ancient polymorphisms (Guerrero and Hahn 2017; Han et al. 2017; Ma et al. 2018; Wang et al. 2019). Although levels of genetic variation vary across the genome of an organism, comparative analyses have consistently found that peaks and troughs of diversity and divergence tend to occur in the same genomic regions across diverse populations, species, and higher taxonomic groups; for example, in plants, such as sunflowers (Renaut et al. 2014), monkeyflowers (Stankowski et al. 2019), poplars (Shang et al. 2023) and in animals, such as butterflies (Kronforst et al. 2013) and birds (Burri et al. 2015; Irwin et al. 2016; Dutoit et al. 2017; Van Doren et al. 2017; Vijay et al. 2017; Delmore et al. 2018). Such correlated landscapes of genomic variation could indicate signatures of parallel evolution (Stern 2013; Magalhaes et al. 2021; Montejo-Kovacevich et al. 2022), but they may also arise from processes not necessarily related to adaptation and speciation (Ellegren and Wolf 2017, Ravinet et al. 2017; Burri 2017a, b; Semenov et al. 2019). Disentangling these intricate patterns of genetic diversity and differentiation remains a pivotal challenge for evolutionary genomics.

Long-term linked selection can generate similar patterns of genomic variation between species. Strong effects of linked selection are expected in genomic regions with low recombination rates and/or high gene density, leading to reduced genetic diversity within species and elevated relative genetic differentiation (*F*_ST_) between species (Maynard Smith and Haigh 1974; Kaplan et al. 1989; Charlesworth et al. 1993b; Charlesworth et al. 1995; Hudson and Kaplan 1995; Cruickshank and Hahn 2014; Slotte 2014). If the landscape of recombination rate variation and gene density remains stable throughout the divergence continuum, linked selection will act repeatedly on the same genomic regions, resulting in a common landscape of genomic variation between species (Renaut et al. 2013; Burri et al. 2015; Irwin et al. 2016; b, c; Van Doren et al. 2017, Vijay et al. 2017; Burri 2017a; Delmore et al. 2018; Stankowski et al. 2019; Chase et al. 2021; Jiang et al. 2023; Shang et al. 2023). Linked selection impacts patterns of genetic variations in two ways: background selection (BGS), which purges deleterious and linked neutral variants (Charlesworth et al. 1993a, 1995; Hudson and Kaplan 1995), and selective sweeps (SW), which fix beneficial alleles and associated variants through genetic hitchhiking (Maynard Smith and Haigh 1974; Kaplan et al. 1989). While both BGS and SW reduce genetic diversity around loci under selection, their impacts on genetic variation patterns differ across the genome and throughout the divergence continuum. For example, BGS is a dominant force in shaping the baseline of genomic variation (Comeron 2014; Rettelbach et al. 2019; Liang et al. 2022; Moreira et al. 2023), whereas SW plays a crucial role in generating highly differentiated regions (Elyashiv et al. 2016; Matthey-Doret and Whitlock 2019; Schrider 2020). Additionally, SW has emerged as the primary force driving genetic differentiation during the early stages of divergence (Delmore et al. 2018; Stankowski et al. 2019), while the influences of BGS and genetic drift on genetic variation accumulate more gradually (Burri 2017a, b). The relative contributions of BGS and SW to the development of a correlated genomic variation landscape remain a subject of ongoing debate.

Gene flow has also been proposed to be a contributing factor to the formation of correlated genomic variation landscapes (Irwin et al. 2016; Stankowski et al. 2019; Shang et al. 2023). When selection acts against gene flow, introgressed fragments tend to be maintained in genomic regions with high recombination rates and low functional element density (Martin and Jiggins 2017; Moran et al. 2021). If hybridizing species have conserved genomic features, frequent introgression may occur in the same genomic regions across multiple species, leading to similar patterns of genomic variation. Analogous patterns may also be generated by adaptive introgression, transmitting, and fixing globally beneficial alleles across species (Dasmahapatra et al. 2012; Irwin et al. 2016; Montejo-Kovacevich et al. 2022). Other evolutionary factors, such as demographic history, effective population size (*N*_e_), and mutation rate, may also be important in shaping genomic variation (Burri 2017b, c). This complex interplay of different forces contributing to the evolution of the genomic variation landscape has not been systematically investigated. Moreover, the correlated landscape of genomic variation has primarily been studied in recently diverged species, or small numbers of distantly related species, leaving understanding of the temporal dynamics of genetic variation landscapes largely incomplete. To address these questions, it is essential to conduct comparative analyses of multiple independent species-pairs across a greater divergence continuum and to integrate various summary statistics to assess the changes in the similarity of genomic variation patterns across long-term evolutionary history.

The genus *Castanopsis* (Fagaceae) includes roughly 120 species (Huang et al. 1999), which dominate tropical and subtropical evergreen broadleaf forests in East and Southeast Asia, offering substantial economic and ecological benefits (Ohsawa 1993; Zhu 1997). Paleontological evidence suggests that the genus *Castanopsis* diverged from its ancestor approximately 52.2 million years ago (MYA) (Wilf et al. 2019), with rapid diversification during the Neogene period giving rise to most extant species (Manos and Stanford 2001; Hai et al. 2022; Zhou et al. 2022a). In contrast to most temperate species in Eurasia and North America, which have undergone significant range shifts during the Quaternary glaciation cycles (Petit and Vendramin 2007; Shafer et al. 2010), *Castanopsis* species have historically stable distribution ranges (Sun et al. 2016; Tang et al. 2022). Thus, the pattern of genetic variation shaped by historical evolutionary processes may have persisted through multiple speciation events in this genus. Additionally, *Castanopsis* species are outcrossing and seldom domesticated (Huang et al. 1999). These features make *Castanopsis* an interesting system for exploring the interplay of distinct evolutionary forces in shaping the long-term evolution of a genomic landscape under natural conditions. While ecological and evolutionary genomic approaches have been applied to investigate the processes and mechanisms underlying adaptation, diversification, and speciation in forest trees (Neale and Kremer 2011; Ingvarsson et al. 2016; Isabel et al. 2020), most studies have focused on a few model species, such as the deciduous *Populus* (Jansson and Douglas 2007) and *Quercus* (Cavender-Bares 2019; Kremer and Hipp 2020) genera. Patterns and processes of genomic divergence in *Castanopsis* have not yet been studied, due to limited available genomic data.

**Fig. 1.**
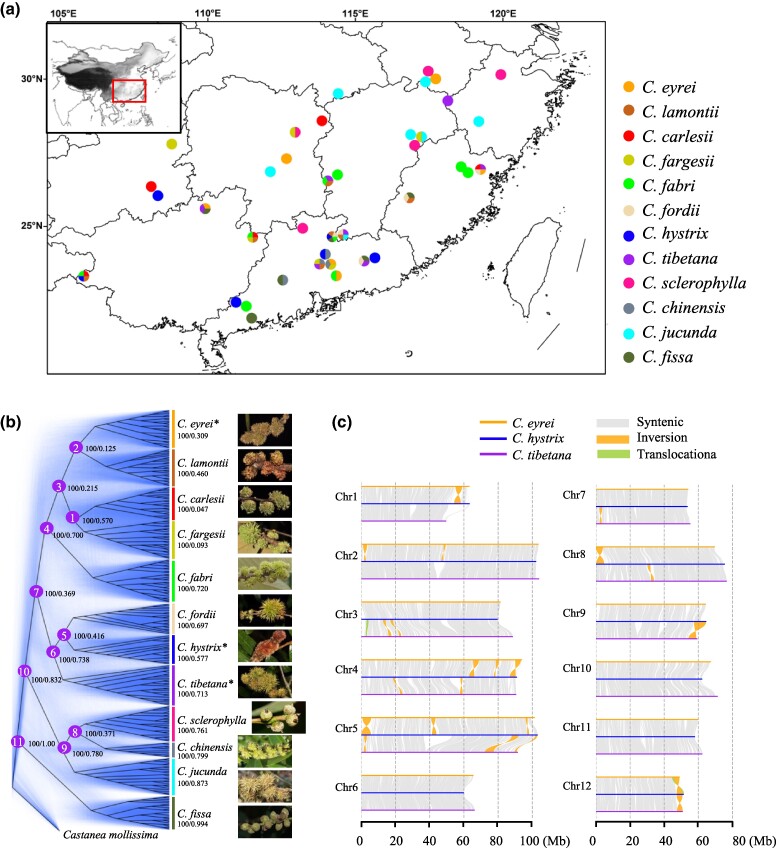
Geographic distribution, phylogenetic relationships, and genomic collinearity between *Castanopsis* species. a) Location of the 39 sampling sites. Pie charts for each site illustrating the numbers of species sampled. b) Phylogenetic trees constructed based on genome-wide SNPs (represented as a black tree) and 100 kb windows (shown as blue trees). For each node or species, bootstrap support values from the whole-genome tree are listed first, followed by the percentage of window-based trees that support the same clade. The 11 labeled nodes on the tree indicate representative contrasts derived using phylogenetic correction. Asterisks indicate the three *Castanopsis* genomes used for synteny analysis in Fig. 1c. c) Genomic synteny and rearrangement plot of the *C. eyrei*, *C. hystrix*, and *C. tibetana* genomes. Only rearrangements larger than 1 Mb are shown. See supplementary fig. S3, Supplementary Material online for all rearrangements larger than 100 kb.

In this study, we generated a chromosome-level genome assembly of *Castanopsis eyrei*, and conducted whole-genome sequencing of 267 individuals collected from the native ranges of 12 representative *Castanopsis* species, namely: *C. carlesii*, *C. chinensis*, *C. eyrei*, *C. fabri*, *C. fargesii*, *C. fissa*, *C. fordii*, *C. hystrix*, *C. jucunda*, *C. lamontii*, *C. sclerophylla*, and *C. tibetana* (Fig. 1a and supplementary table S1, Supplementary Material online). These species represent a divergence continuum spanning approximately 40 MYA (Zhou et al. 2022a), enabling exploration of the long-term evolution of a genomic variation landscape. The distribution ranges of the 12 *Castanopsis* species show significant overlap, often coexisting in mixed forests (supplementary fig. S1, Supplementary Material online; Huang et al. 1999). Previous studies based on genetic markers have uncovered high levels of genetic variation and extensive interspecific introgression in these species (Shi et al. 2011; Li et al. 2014; Sun et al. 2016; Jiang et al. 2020; Sun and Wen 2020; Li et al. 2022), suggesting that numerous evolutionary forces, including selection, gene flow, and population demography, may have contributed to shaping the patterns of genomic variation in these species. We aimed to address three questions: (i) Do these *Castanopsis* species have correlated genomic variation landscapes, and to what extent do they show similarity in various genetic parameters? (ii) Which mechanisms drive the patterns of genomic variation observed in these species, with particular attention to distinguishing the effects of SW from those of BGS? (3) What are the dynamics of gene flow between these species, and how does gene flow contribute to the genomic variation landscape?

## Results

### High-quality *Castanopsis eyrei* Genome Assembly

To facilitate the investigation of genomic variation in the genus *Castanopsis*, we generated a chromosome-level genome assembly of *C. eyrei* by integrating approximately 110 Gb (approximately 120×) PacBio long reads, 118 Gb (∼130×) Hi-C reads, and 48 Gb (∼50×) Illumina paired-end reads. The final assembly was 891.03 Mb, including 441.67 Mb (49.57%) repetitive sequences (supplementary table S2, Supplementary Material online). The *C. eyrei* genome assembly displayed high quality and completeness, with 873.94 Mb (98.1%) of sequences anchored to 12 chromosomes, a scaffold N50 of 69.73 Mb, and a Benchmarking the Universal Single-Copy Orthologs (BUSCO) score of 94.9% (supplementary fig. S2, Supplementary Material online, supplementary table S2, Supplementary Material online). Using RNA-seq-based, protein-homology-based, and ab initio predictions, 45,904 protein-coding genes were predicted in the *C. eyrei* genome (supplementary table S2, Supplementary Material online). Furthermore, comparative genomic analyses of the *C. eyrei* genome with the previously published genomes of *C. hystrix* (Huang et al. 2023) and *C. tibetana* (Sun et al. 2022) demonstrated high collinearity (Fig. 1c and supplementary fig. S3, Supplementary Material online) and similar patterns of gene density (Spearman's *ρ* = 0.55 to 0.64, *P* < 2.2e^−16^, *n* = 1,234 to 1,340; supplementary fig. S4, Supplementary Material online), suggesting conserved genomic architectures among these *Castanopsis* species.

### Population Structure and Demographic Histories of *Castanopsis* Species

To investigate the phylogenetic relationships and population structure of 12 species representing major *Castanopsis* lineages, we performed whole-genome re-sequencing of 267 individuals. Samples were collected from 39 locations across the species' geographic range, encompassing 5 to 7 populations per species, except that two populations were sampled for *C*. *chinensis* (Fig. 1a; supplementary fig. S1, Supplementary Material online and supplementary table S1, Supplementary Material online). Re-sequencing data were mapped to the *C*. *eyrei* reference genome, and 52,385,983 high-quality single nucleotide polymorphisms (SNPs) were obtained for subsequent analyses (see Materials and Methods). Population structure analyses demonstrated remarkable differentiation among the 12 species. First, a whole-genome phylogeny demonstrated well-resolved relationships between these species, and individuals from each species formed monophyletic clades, with 100% bootstrap support (Fig. 1b and supplementary fig. S5, Supplementary Material online). The phylogenetic relationships between these species were generally consistent with those previously reported, based on single-copy nuclear genes (Zhou et al. 2022a). Second, admixture analyses using ADMIXTURE v1.3.0 (Alexander et al. 2009) revealed that each species was dominated by a distinct genetic ancestry, with the lowest cross-validation error obtained when *K* = 12 (supplementary fig. S6, Supplementary Material online). Third, a principal component analysis (PCA) performed using EIGENSOFT v6.0 (Patterson et al. 2006) clearly separated the 12 species along axes PC1 to PC6 (supplementary fig. S7, Supplementary Material online), further confirming the high genetic differentiation between these species. Despite the clear population structure detected, window-based trees conflicted strongly with the genome-wide tree regarding the relationships between recently diverged species (Fig. 1b and supplementary fig. S5, Supplementary Material online), indicating the possibility of incomplete lineage sorting or interspecific hybridization.

To gain insight into the demographic histories of the 12 *Castanopsis* species, we conducted multiple sequential Markovian coalescent (MSMC) analysis using MSMC v.2.0.0 (Schiffels and Durbin 2014). By assuming a mutation rate of 8.21 × 10^−10^ per site per year and a generation time of 100 yr for *Castanopsis* species (see Materials and Methods for details), we converted the demographic inferences into absolute values. Our results suggested that all species experienced population fluctuations during the Pleistocene epoch, with varying degrees of decline and expansion (supplementary fig. S8, Supplementary Material online). Specifically, seven species, *C. carlesii*, *C. eyrei*, *C. fargesii*, *C. fabri*, *C. fordii*, *C. hystrix*, and *C. lamontii*, underwent population expansions approximately 1.0 to 0.3 MYA, followed by short periods of population bottlenecks between 0.05 and 0.02 MYA (supplementary fig. S8, Supplementary Material online). The remaining five species (*C. tibetana*, *C. jucunda*, *C. chinensis*, *C. sclerophylla*, and *C. fissa*), on the other hand, experienced longer population bottlenecks (1.0 to 0.1 MYA), followed by subsequent population expansions (supplementary fig. S8, Supplementary Material online). Notably, *N*_e_ was generally large (ranging from 10^4^ to 10^5^) during the long-term evolutionary history of all species except *C. jucunda*, *C. tibetana*, and *C. fissa*, which had relatively small *N*_e_ (ca. 3 × 10^3^) during the periods of population bottlenecks (supplementary fig. S8, Supplementary Material online). It is worth noting that the inferred population demographic parameters are dependent on the assumed mutation rate and generation time, both of which are challenging to estimate accurately for *Castanopsis* species in their natural habitat. Consequently, any discrepancies in these estimates would require adjustments to the inferred times and population sizes. However, given a conserved mutation rate and generation time across *Castanopsis* species, the relative trends in demographic histories uncovered in this study appear to be reasonable.

The existence of population structure can bias demographic inference and lead to overestimations of effective population size (Chikhi et al. 2010). To examine how population structure may impact demographic inferences for *Castanopsis* species, we conducted PCA analyses to explore population subdivisions within each species. Our analyses identified 2 to 4 genetic groups per species, with geographically neighboring populations generally clustering together (supplementary fig. S1, Supplementary Material online). We then performed MSMC analyses for each genetic group and compared the results with those from the pooled samples. The demographic histories reconstructed for the genetic groups were largely consistent with those of the entire species, except for a few discrepancies observed in marginal populations of several species (supplementary fig. S1, Supplementary Material online). These findings suggest that population structure may have minimal effects on demographic reconstruction for *Castanopsis* species. Future studies will be essential to comprehensively capture the demographic history of these *Castanopsis* species. This will require extensive sampling across their distribution range and the application of methods capable of simultaneously inferring multiple population demographic parameters, such as effective population sizes (*N*_e_), divergence times, and gene flow.

### Genomic Variation Landscapes in *Castanopsis* Species

**Fig. 2.**
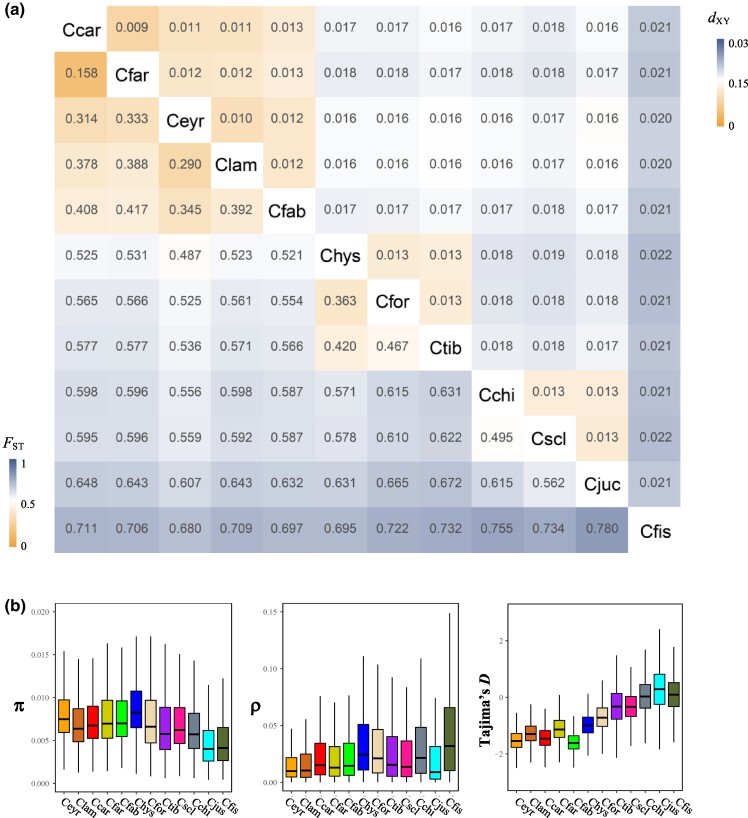
Comparisons of genomic divergence and diversity across *Castanopsis* species. a) Heatmap showing relative genetic differentiation (*F*_ST_) and absolute genetic divergence (*d*_XY_) across 66 species pairs. b) Boxplot showing nucleotide diversity (π), population-scaled recombination rate (ρ), and Tajima's *D* across 12 species. In each plot, the horizontal line indicates the median, with the bottom and top of the boxes representing the first and third quartiles, respectively. Whiskers extend to 1.5 times the interquartile range, and outliers are excluded from the plots. All summary statistics were estimated in non-overlapping windows of 100 kb. Abbreviations: Ceyr, *C. eyrei*; Clam, *C. lamontii*; Ccar, *C. carlesii*; Cfar, *C. fargesii*; Cfab, *C. fabri*; Chys, *C. hystrix*; Cfor, *C. fordii*; Ctib, *C. tibetana*; Cscl, *C. sclerophylla*; Cchi, *C. chinensis*; Cjuc, *C. jucunda*; Cfis, *C. fissa*.

To assess the level of genetic variation between and within *Castanopsis* species, we calculated a set of summary statistics in non-overlapping windows of 10, 100, and 500 kb. The major conclusions were not altered by using different windows sizes, although smaller windows generated a more heterogeneous genomic variation landscape. Here, we present the results based on 100 kb windows, reflecting a stable genome-scale pattern, and provide the results for the 10 and 500 kb windows in supplementary data, Supplementary Material online. Among all 66 species-pairs, *F*_ST_ and absolute genetic divergence (*d*_XY_) values ranged from 0.158 to 0.781 and from 0.009 to 0.022, respectively (Fig. 2 and supplementary table S3, Supplementary Material online), suggesting that the 12 species analyzed represent a long divergence continuum in the genus *Castanopsis*. Genome-wide nucleotide diversity (π = 0.005 to 0.010) and population-scaled recombination rate (ρ = 0.020 to 0.055) were comparable between species (Fig. 2; supplementary fig. S9, Supplementary Material online and supplementary table S4, Supplementary Material online). By contrast, the average Tajima's *D* was highly variable between species, ranging from −1.565 to 0.274 (supplementary fig. S9, Supplementary Material online and supplementary table S4, Supplementary Material online), likely reflecting their distinct recent demographic histories (supplementary fig. S8, Supplementary Material online).

Levels of genetic variation varied across genomic regions in *Castanopsis* species. We found non-randomly distributed genomic windows exhibiting elevated *F*_ST_ or *d*_XY_ scattered across the genome for all species-pairs we examined (supplementary fig. S10, Supplementary Material online), which tended to cluster in short genomic regions (*P* < 0.01, 10,000 permutations in autocorrelation analyses; supplementary fig. S11, Supplementary Material online). Similarly, a non-random distribution pattern of within-species summary statistics was also observed (π, ρ, and Tajima's *D*; *P* < 0.01, 10,000 permutations in autocorrelation analyses; supplementary fig. S11, Supplementary Material online). Notably, the non-random distribution of the summary statistics was particularly evident at small window sizes (10 kb), suggesting that windows with similar summary statistics tend to cluster in relatively short genomic regions. On comparison of window-based estimates of genetic differentiation between species-pairs, we observed strong correlations for each pairwise comparison (Spearman's *ρ* = 0.58 to 0.91 and 0.17 to 0.92 for *F*_ST_ and *d*_XY_, respectively, *P* < 0.001, *n* = 5,608 to 6,258; supplementary fig. S12, Supplementary Material online). Due to the phylogenetic relatedness among the 12 *Castanopsis*, pairwise comparisons may lack evolutionary and statistical independence. In order to address the issue of non-independence between comparisons, 11 representative contrasts were generated by applying a phylogenetic correction approach (Felsenstein 1985; Coyne and Orr 1989). Significant correlations of *F*_ST_ (Spearman's *ρ* = 0.60 to 0.95, *P* < 0.001, *n* = 5,793 to 6,331) and *d*_XY_ (Spearman's *ρ* = 0.28 to 0.88, *P* < 0.001, *n* = 5,793 to 6,331) were observed among the 11 contrasts (supplementary fig. S12, Supplementary Material online). Similar to the correlated patterns of genetic differentiation, we observed a significant positive correlation of nucleotide diversity (π) between species (Spearman's *ρ* = 0.65 to 0.88, *P* < 0.001, *n* = 5,875 to 6,355; supplementary fig. S12, Supplementary Material online). Genome-wide patterns of population-scaled recombination rate were also conserved among species (Spearman's ρ = 0.15 to 0.71, *P* < 0.001, *n* = 5,045 to 6,335; supplementary fig. S12, Supplementary Material online). However, the correlation coefficient of recombination rates was generally lower than that of π (supplementary fig. S12, Supplementary Material online), suggesting that factors other than recombination rate have influenced patterns of genetic diversity. Further, we observed much weaker correlations of Tajima's *D* between species (Spearman's ρ = 0.06 to 0.53, *P* < 0.001, *n* = 7,617 to 8,529; supplementary fig. S12, Supplementary Material online) than those for genetic diversity and recombination rate, again likely reflecting the varying demographic histories we observed in these species (supplementary fig. S8, Supplementary Material online).

To further assess the similarity of genomic variation landscapes, we implemented a methodology developed by Stankowski et al. (2019). We normalized the window-based estimates, and utilized PCA to summarize the variation of each statistic across the 12 species (π, ρ, and Tajima's *D*) or 11 representative contrasts (*F*_ST_ and *d*_XY_). The first principal component (PC1) explained 78.6% and 65.9% of the variation in *F*_ST_ and *d*_XY_, respectively (supplementary fig. S13, Supplementary Material online), and all contrast nodes were loaded positively onto PC1 (0.72 to 0.89 and 0.69 to 0.97 for *F*_ST_ and *d*_XY_, respectively; supplementary table S5, Supplementary Material online). Similarly, PC1 captured 77.3%, 42.4%, and 29.1% of the variance in π, ρ, and Tajima's *D* between species using 100 kb windows (supplementary fig. S13, Supplementary Material online), with all species showing positive loadings on PC1 (0.82 to 0.91, 0.43 to 0.76, and 0.25 to 0.69 for π, ρ, and Tajima's *D*, respectively; supplementary table S5, Supplementary Material online). Furthermore, PC1 score values were positively correlated with the average values for each summary statistic (Spearman's *ρ* = 0.93 to 1.00, *P* < 0.001, *n* = 4,844 to 7,289; supplementary fig. S14, Supplementary Material online). These results strongly support a conserved pattern of genomic diversity and divergence across all species, and suggest that PC1 scores can be used to effectively represent the common variation in these summary statistics. Additionally, using the PC1 score to assess correlations among summary statistics can help eliminate the issue of intercorrelations among variables.

### Correlations Between Genetic Variation and Genomic Features

**Fig. 3.**
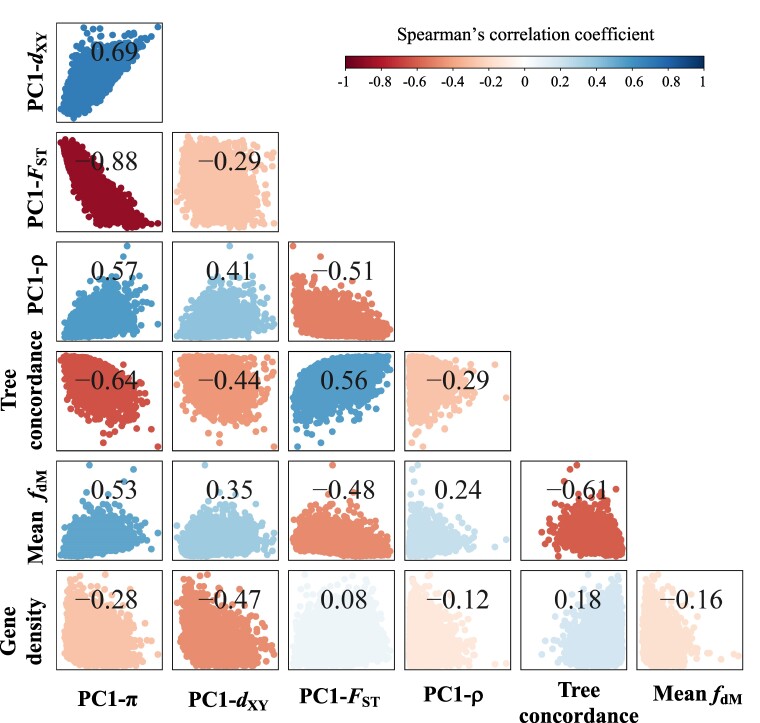
Significant correlations between population genetic variation and genomic features in *Castanopsis* species. Principal component analysis (PCA) was employed to summarize the variation in each summary statistic. The first principal component (PC1) score was calculated across 12 species for π, ρ, and Tajima's *D*, and across 11 representative contrasts for *F*_ST_ and *d*_XY_, which were derived using a phylogenetic correction approach. PC1-π, PC1-ρ, PC1-*F*_ST_, and PC1-*d*_XY_ refer to the PC1 scores of π, ρ, *F*_ST_, and *d*_XY_, respectively. Spearman's correlation coefficient values were calculated for comparisons between PC1-π, PC1-*d*_XY_, PC1-*F*_ST_, PC1-ρ, tree concordance, averaged *f*_dM_, and gene density. All summary statistics were estimated in 100 kb non-overlapping windows. The heatmap illustrates the magnitude and direction of Spearman's correlation test; all correlations were significant (*P* < 0.001).

To explore the evolutionary processes that have influenced the common pattern of genomic variation in *Castanopsis*, we examined the relationship between the PC1 scores from the summary statistics and various genomic features, and detected several significant correlations between these parameters. First, we found a positive correlation between PC1-π and PC1-*d*_XY_ and a negative correlation between PC1-π and PC1-*F*_ST_ (Spearman's *ρ* = 0.69 and −0.88, respectively; *P* < 0.001, *n* = 5,454; Fig. 3 and supplementary fig. S15, Supplementary Material online), indicating that genomic regions with higher genetic diversity also exhibited greater absolute genetic divergence and lower relative genetic differentiation. Furthermore, we observed consistent positive correlations between π and *d*_XY_, and negative correlations between π and *F*_ST_ across the 11 representative contrasts (supplementary table S6, Supplementary Material online), confirming the robustness of our results based on PC1 scores. Additionally, PC1-π was negatively correlated with tree concordance (Spearman's *ρ* = −0.64, *P* < 0.001, *n* = 5,510; Fig. 3 and supplementary fig. S15, Supplementary Material online), suggesting that regions with lower genetic diversity experienced more rapid sorting of ancient polymorphisms.

We also found significant associations between genomic features and genomic variation. The population-scaled recombination rate (PC1-ρ) was positively correlated with PC1-π (Spearman's *ρ* = 0.57, *P* < 0.001, *n* = 4,844) and PC1-*d*_XY_ (Spearman's *ρ* = 0.41, *P* < 0.001, *n* = 4,801), while it was negatively correlated with PC1-*F*_ST_ (Spearman's *ρ* = −0.51, *P* < 0.001, *n* = 4,801; Fig. 3 and supplementary fig. S15, Supplementary Material online). Conversely, gene density was negatively correlated with both PC1-π (Spearman's *ρ* = −0.28, *P* < 0.001, *n* = 4,844) and PC1-*d*_XY_ (Spearman's *ρ* = −0.47, *P* < 0.001, *n* = 4,801), while a weak but significant positive correlation was detected with PC1-*F*_ST_ (Spearman's *ρ* = 0.08, *P* < 0.001, *n* = 4,801; Fig. 3 and supplementary fig. S15, Supplementary Material online). These associations suggest that recombination rate and gene density contributed to the patterns of genomic variation observed in *Castanopsis*.

**Fig. 4.**
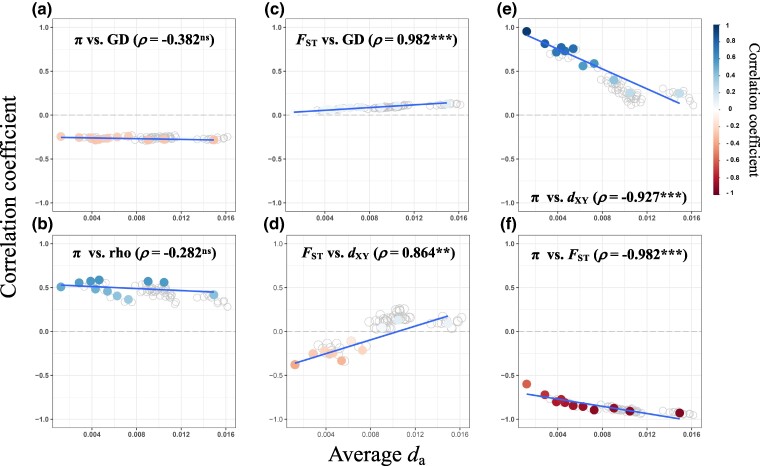
Changes in population summary statistic correlations over the *Castanopsis* divergence continuum. Correlations coefficient values between summary statistics (100 kb windows) for all 66 *Castanopsis* species-pairs (represented as gray open dots) plotted against averaged *d*_a_ as a proxy of divergence time. Left panels: evolving relationships between a) nucleotide divrsity (π) and gene density (GD) and b) π and recombination rate (rho), across increasing divergence time. Middle panels: relationships between c) *F*_ST_ and GD, and d) *F*_ST_ and *d*_XY_. Right panels: dynamics between e) π and *d*_XY_, and f) π and *F*_ST_ over time. The π and rho values were averaged across species-pairs. In each plot, the blue line indicates a linear regression fit to the 11 representative contrasts (shown as filled colored dots) derived using phylogenetic correction. The color gradient of dots indicates the strength of correlation. **P < 0.01, ***P < 0.001, ^ns^non-significant.

To further investigate how the correlations between population statistics evolved through the divergence continuum, we conducted linear regression analysis of correlation coefficients and divergence time across the 11 representative contrasts (see Materials and Methods for details). Our results provide insights into the temporal patterns of genomic landscape evolution over an extended divergence time. Correlations between π and genomic features (recombination rate and gene density) remained unchanged with increasing divergence time between species (Fig. 4 and supplementary table S7, Supplementary Material online). Conversely, the correlation between π and *d*_XY_ was initially strong, but became weaker as divergence time increased (Spearman's *ρ* = −0.927, *P* < 2.2e^−16^, *n* = 11; Fig. 4 and supplementary table S7, Supplementary Material online). Further, the correlations between *F*_ST_ and π, and between *F*_ST_ and gene density, became stronger with increasing divergence time (Spearman's *ρ* = −0.982 and 0.982, respectively; *P* < 2.2e^−16^, *n* = 11; Fig. 4 and supplementary table S7, Supplementary Material online). By contrast, the association between *F*_ST_ and recombination rate was not correlated with *d*_a_ (Spearman's *ρ* = 0.428, *P* = 0.189, *n* = 11; supplementary table S7, Supplementary Material online). Finally, the correlation between *F*_ST_ and *d*_XY_ was significantly associated with divergence time (Spearman's *ρ* = 0.864, *P* = 0.00128, *n* = 11; Fig. 4 and supplementary table S7, Supplementary Material online). Interestingly, *F*_ST_ was negatively correlated with *d*_XY_ for 16 recently diverged species-pairs (Spearman's *ρ* ranged from −0.379 to −0.111; *P* < 0.001, *n* = 5,793 to 6,318), but positively correlated with *d*_XY_ for the remaining 50 species-pairs (Spearman's *ρ* ranged from 0.031 to 0.256, *P* < 0.001, *n* = 5,829 to 6,331; Fig. 4 and supplementary table S6, Supplementary Material online).

### Gene Flow Between *Castanopsis* Species

**Fig. 5.**
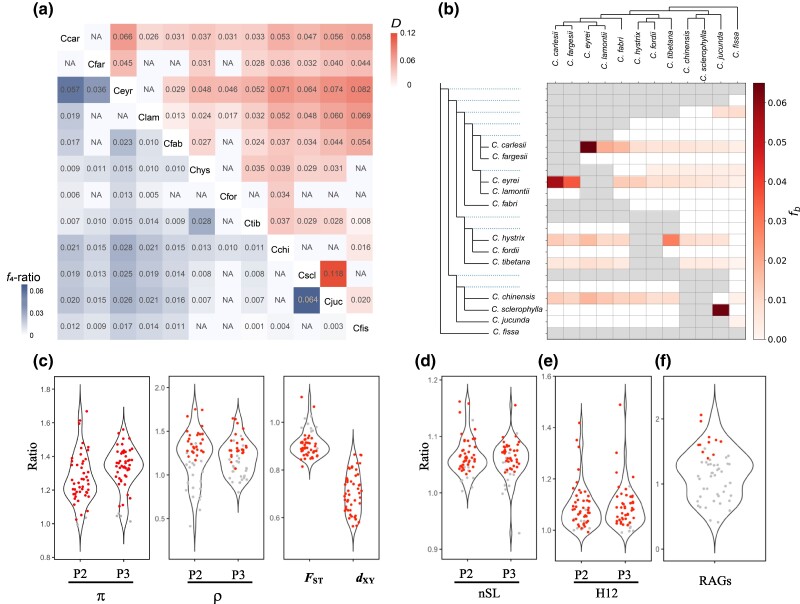
Patterns of gene flow between 12 *Castanopsis* species. a) Heatmap showing the maximum *D* value (top diagonal) and *f*_4_-ratio (bottom diagonal) for each pair of species across all trio combinations. Entries labeled “NA” denote species-pairs for which introgression tests were not applicable or where non-significant *D* values were observed (FDR <0.05). b) Matrix of *f*_b_ statistics, illustrating gene flow between tree branches and species. At the top of the matrix, a tree shows the phylogenetic relationship between the species, whereas the expanded tree on the left represents both terminal and ancestral branches. c) Estimates of summary statistics, including π, recombination rate (ρ), *F*_ST_, and *d*_XY_ within introgression regions compared with the genomic background. d, e) Assessment of adaptive introgression by comparing nSL d) and H12 e) values in introgression regions against the genomic background. f) Ratio of resistance gene analogs (RAGs) in introgression regions relative to the genomic background. In panels c to f, red dots indicate significant differences (*P* < 0.05, determined using the Wilcoxon-Mann Whitney *U*–test in c–e and the hypergeometric test in f), while gray dots represent non-significant findings.

Gene flow can facilitate the evolution of correlated differentiation landscapes, particularly when reproductive isolation is highly polygenic and common among species (Stankowski et al. 2019). To assess the extent of gene flow between *Castanopsis* species, we calculated the *D*-statistic (Green et al. 2010; Durand et al. 2011) and the *f*_4_ admixture ratio (*f*_4_-ratio) (Patterson et al. 2012) for 220 combinations of trios extracted from the species tree, using *Castanea mollissima* as the outgroup (supplementary table S8, Supplementary Material online). For P2–P3 species-pairs tested using different “control” P1 species, we retained comparisons with maximum *D* values, resulting in 51 non-redundant trios (Fig. 5a and supplementary table S8, Supplementary Material online). We observed extensive gene flow between *Castanopsis* species, with all 51 trios having significant *D* values (FDR <0.05). Further, *f*_4_-ratio values were negatively correlated with divergence time (Spearman's *ρ* = −0.46, *P* < 0.001, *n* = 51; supplementary fig. S16, Supplementary Material online), suggesting higher levels of introgression between closely related species. Moreover, calculation of the *f*-branch statistic (Malinsky et al. 2018) revealed that the majority of introgression events occurred between extant species, with only two internal branch nodes having significant *f*-branch values (Fig. 5b). These findings suggest that the extensive introgression signal observed in *Castanopsis* species can likely be attributed to multiple introgression events between different, extant species rather than to a few ancestral events.

To explore variations in interspecific introgression across the genome, we calculated *f*_d_ (Martin et al. 2015) and *f*_dM_ (Malinsky et al. 2015) in 100 kb non-overlapping windows. These two statistics were highly correlated along the chromosome for each trio (Spearman's *ρ* = 0.97 to 1.00, *P* < 2.2e^−16^, *n* = 2,950 to 3,773; supplementary fig. S17, Supplementary Material online). Because the *f*_dM_ statistic is a modified version of *f*_d_ with positive values indicating gene flow between P2 and P3 results, we used results of *f*_dM_ for subsequent analyses (Malinsky et al. 2015). Patterns of introgression estimated by *f*_dM_ showed considerable heterogeneity across the genome (supplementary fig. S10, Supplementary Material online) and were significantly correlated with genetic variation and genomic feature landscapes (Fig. 3). First, the mean *f*_dM_ (averaged over 51 trios) was positively correlated with PC1-π (Spearman's *ρ* = 0.53, *P* < 0.001, *n* = 5,509) and negatively correlated with PC1-*F*_ST_ (Spearman's *ρ* = −0.48, *P* < 0.001, *n* = 5,605; Fig. 3). Notably, mean *f*_dM_ was positively correlated with PC1-*d*_XY_ (Spearman's *ρ* = 0.35, *P* < 0.001, *n* = 5,605; Fig. 3), while 47 of 51 trios showed negative correlations between *d*_XY_ and *f*_dM_ (Spearman's *ρ* ranged from −0.221 to −0.0034, *P* < 0.001, *n* = 2,964 to 3,780; supplementary table S9, Supplementary Material online). These results suggest that gene flow increases the genetic variation within species and counteracts the genetic differentiation between species. Second, mean *f*_dM_ was positively correlated with recombination rate (Spearman's *ρ =* 0.24, *P* < 0.001, *n* = 4,843) and negatively correlated with gene density (Spearman's *ρ =* −0.16, *P* < 0.001, *n* = 4,843; Fig. 3), suggesting that introgression is more likely to occur in genomic regions with higher recombination and lower gene density. Similar results were obtained using *f*_dM_ estimated in 10 and 500 kb non-overlapping windows (Supplementary fig. S15, Supplementary Material online and supplementary table S9, Supplementary Material online).

### Inferring Adaptive Introgression

Adaptive introgression can introduce advantageous variants from the donor species and enhance the fitness of the recipient species (Martin and Jiggins 2017; Moran et al. 2021). To search for signals of adaptive introgression between the *Castanopsis* species, we identified a total of 4,862 100 kb windows as putative introgression regions across the 51 trios (see Materials and Methods for details). Numbers of introgression windows scattered across the genome ranged from eight in the trio (*C. fordii*, *C. tibetana*, and *C. fissa*) to 371 in the trio (*C. chinensis*, *C. sclerophylla*, and *C. jucunda*) (Supplementary fig. S18, Supplementary Material online and supplementary table S10, Supplementary Material online). Compared with the genomic background, introgression regions generally showed lower genetic divergence (*F*_ST_ and *d*_XY_) between species, with higher nucleotide diversity (π) and recombination rates (ρ) in both P2 and P3 species for most trios (*W* ranges from 7,656 to 1,077,523, *n*1 = 8 to 371, *n*2 = 7,494 to 8,291, *P* < 0.05, Wilcoxon-Mann-Whitney *U*-test; Fig. 5c and supplementary table S10, Supplementary Material online). Using *C. eyrei* gene model, we observed that introgression regions tended to be concentrated in genomic regions with lower gene density in 39 of the 51 trios, although significant comparisons were only detected in three trios (*W* ranges from 14,862 to 764,550, *n*1 = 8 to 371, *n*2 = 7,494 to 8,291, *P* < 0.05, Wilcoxon-Mann-Whitney *U*-test; supplementary table S10, Supplementary Material online), possibly due to the impact of small sample size and incomplete gene annotation on the statistical power of the analysis.

To test for signals of selection in introgression regions, we scanned the genome for signatures of positive selection using two haplotype-based statistics: number of segregating sites by length (nSL) (Ferrer-Admetlla et al. 2014) and H12 (Garud et al. 2015). Relative to genomic background, introgression regions exhibited higher nSL and H12 values in either P2 or P3 species in 42 of 51 trios (*W* ranges from 27,607 to 1,814,290, *n*1 = 8 to 371, *n*2 = 7,494 to 8,291, *P* < 0.05, Wilcoxon-Mann Whitney *U*-test; Fig. 5d and e and supplementary table S10, Supplementary Material online), suggesting that these regions may have experienced adaptive introgression.

We further evaluated the potential roles of introgression regions in adaptation by testing for functional enrichment of disease resistance genes. Using a RGAugury (Li et al. 2016) to predict resistance gene analogs (RGAs), we identified 1,678 RGAs in the *C. eyrei* genome, representing 3.66% of all predicted genes. RGAs include 568 nucleotide-binding leucine-rich repeat sequence (NBS-LRR) genes, 190 receptor-like proteins, 740 receptor-like kinases, and 180 transmembrane-coiled-coil (TM-CC) genes. RGAs were enriched in introgression regions of 33 trios, with 11 showing significant enrichment (*P* < 0.05, hypergeometric test; Fig. 5f and supplementary table S11, Supplementary Material online). Importantly, most of these 33 trios showed a higher ratio of all four types of RGAs in introgression regions relative to genomic background (supplementary table S11, Supplementary Material online). Further, gene ontology (GO) analyses using GOWINDA (Kofler and Schlötterer 2012) revealed that introgression regions in 30 of 51 trios exhibited significant enrichment of 1 to 8 GO terms, including response to metal ions, pollen recognition, and auxin response (FDR < 0.05; supplementary table S12, Supplementary Material online). These results suggest that specific functional gene classes were more likely to be introgressed in specific trios, indicating potential adaptive advantages associated with introgression regions.

Finally, we hypothesized that adaptive introgression could reduce the genetic burden of deleterious mutations in introgression regions, and tested this by comparing the genetic burdens between introgression regions and the reminder of the genome in P2 and P3 species of each trio. To do that, we combined SIFT4G (Vaser et al. 2016) and PROVEAN (Choi et al. 2012) criteria, to identify deleterious SNPs (dSNPs). To remove the influence of sequencing quality across the genome, genetic burden was estimated as a ratio of derived dSNP alleles versus neutral SNPs (4-fold degenerate sites) under both additive and recessive models (see Materials and Methods for details). For all trios, the genetic burden estimated under an additive model was higher than that estimated under a recessive model in both introgression regions and the genomic background, suggesting that deleterious mutations were mainly maintained in a heterozygous state in *Castanopsis* species. Under the additive model, introgression regions showed significantly lower genetic burden than genomic background in either P2 or P3 species for 11 trios (*W* ranges from 4 to 650, *n*1 = 8 to 371, *n*2 = 7,494 to 8,291, *P* < 0.05, Wilcoxon-Mann Whitney *U*-test; supplementary table S10, Supplementary Material online). Under the recessive model, reduced genetic burden was detected in the introgression regions of at least one species for 24 trios (*W* ranges from 20 to 599, *n*1 = 8 to 371, *n*2 = 7,494 to 8,291, *P* < 0.05, Wilcoxon-Mann Whitney *U*-test; supplementary table S10, Supplementary Material online).

As natural selection is more efficient at purging deleterious mutations in species with larger effective population size (*N*_e_), introgression from donor species with large *N*_e_ is expected to reduce the genetic burden in recipient species with smaller *N*_e_ (Harris and Nielsen 2016; Wang, et al. 2017; Kim, et al. 2018). To test this hypothesis, we focused on 14 trios with contrasting long-term *N*_e_ between the hybridizing species; specifically, where the *N*_e_ of P2 was approximately 1.5 times that of P3 (supplementary table S10, Supplementary Material online). Our results revealed mixed evidence regarding the effect of long-term *N*_e_ in shaping the burden of deleterious mutations in introgression regions. Under the recessive model, we found a lower genetic burden in P3 species introgression regions in four trios, suggesting that introgression from P2 with a larger *N*_e_ may have reduced the genetic load in P3. However, an increased genetic burden was observed in introgression regions of P3 species in the other eight trios (supplementary table S10, Supplementary Material online).

Considering that the 12 *Castanopsis* species experienced population fluctuations during their evolutionary history (supplementary fig. S8, Supplementary Material online), it would be interesting to investigate whether *N*_e_ inferred from MSMC provides results more consistent with the observed differences in mutation loads. To do that, we compared the changes in *N*_e_ between P2 and P3 species across 24 trios (supplementary fig. S19, Supplementary Material online and supplementary table S10, Supplementary Material online), each of which showed a reduced recessive genetic burden in the introgression regions of at least one species. We found that ten trios showed a reduced genetic burden in species with a lower *N*_e_ during the period of 0.03 to 0.1 MYA (supplementary fig. S19, Supplementary Material online), supporting the hypothesis that introgression from a donor species with a larger *N*_e_ reduces the genetic burden in recipient species with a smaller *N*_e_. However, the remaining 14 trios exhibited a reduced genetic burden in species with a larger *N*_e_ or in those with similar *N*_e_ between P2 and P3 species (supplementary fig. S19, Supplementary Material online), conflicting with the proposed hypothesis.

## Discussion

### Common Genomic Landscapes Shaped by Linked Selection and Conserved Genomic Features

Although correlated patterns of genomic variation are well documented, our understanding of the temporal dynamics of genetic variation landscapes remains limited. In this study, we conducted comparative population genomics analyses of 12 *Castanopsis* species, representing a long-term divergence continuum, which revealed a high level of similarity in their genomic diversity and differentiation landscapes. Multiple lines of evidence support the crucial role of linked selection in shaping the observed common pattern of genomic variation across these species. First, variation in genetic diversity and differentiation were found to be non-randomly distributed across genomes of *Castanopsis* species. Heterogenous landscapes and non-random distribution patterns of genomic variation are considered signals of selective processes shaping genomic variation in many species, including monkeyflowers (Stankowski et al. 2019), poplars (Rendón-Anaya et al. 2021; Shang et al. 2023), sunflowers (Renaut et al. 2013, 2014), oaks (Leroy et al. 2020; Fu et al. 2022; Liang et al. 2022), and birds (Burri et al. 2015; Irwin et al. 2016; Vijay et al. 2016; Han et al. 2017; Van Doren et al. 2017; Delmore et al. 2018). Second, genetic diversity (π) was positively correlated with absolute genetic divergence (*d*_XY_) and negatively correlated with relative genetic divergence (*F*_ST_), consistent with a model of long-term linked selection that reduces genetic variation in both ancestor and descendent species, resulting in locally reduced *d*_XY_ and elevated *F*_ST_ (Cruickshank and Hahn 2014; Burri 2017b). Third, genetic diversity was positively correlated with population-scaled recombination rate and negatively correlated with gene density, consistent with the expectation that the efficacy of linked selection is influenced by genomic features such as gene density and recombination rate (Maynard Smith and Haigh 1974; Kaplan et al. 1989; Charlesworth et al. 1995; Hudson and Kaplan 1995; Cruickshank and Hahn 2014; Slotte 2014).

Given the importance of recombination rate and gene density as key factors influencing the effects of linked selection, common genomic landscapes can be attributed to conserved genomic features (Burri 2017a, b). In agreement with this expectation, we found that genomes of *Castanopsis* species are evolutionarily stable, with similar genomic distribution patterns of population-scaled recombination rates and gene densities. Therefore, it is plausible that long-term linked selection has influenced the same genomic regions in *Castanopsis* species, due to their conserved genomic characteristics, resulting in common patterns of genomic variation among them. Additionally, we observed a significant negative correlation between recombination rate and gene density (Fig. 3 and supplementary fig. S15, Supplementary Material online). The interplay between these two factors may have further enhanced the effects of linked selection and facilitated the formation of correlated genomic variation landscapes in the *Castanopsis* species.

The correlated genomic variation landscapes may also be further attributed to conserved variation in mutation rate across species. Following previous studies (Irwin et al. 2016; Van Doren et al. 2017), we examined the correlation of *d*_XY_ between *Castanopsis* species-pairs and between a pair of species from the distantly diverged genus, *Quercus* (see Materials and Methods for details). We found that *d*_XY_ correlation values for *Castanopsis–Quercus* comparisons (Spearman's *ρ* = 0.27 to 0.37) were comparable to previous estimates between two bird genera, which attributed the correlation between *d*_XY_ to a conserved mutation rate influencing the genomic variation landscape (Irwin et al. 2016). Therefore, we propose that a contribution of conserved variation in mutation rates to the observed similar genomic variation patterns is plausible.

In addition to genetic features, genomic landscape patterns of *Castanopsis* species may have been influenced by genome-wide effective population size (*N*_e_) and population demography. Species with large *N*_e_ and similar demographic histories are expected to have more strongly correlated differentiation landscapes (Van Doren et al. 2017; Burri 2017c). Our findings reveal that all 12 *Castanopsis* species had large *N*_e_, where the influence of selection can overwhelm genetic drift, leading to reduced genetic diversity in the same genomic regions across multiple species. However, we also observed that these *Castanopsis* species have experienced different magnitudes and durations of bottleneck and expansion events. These distinct demographic histories may contribute to the low correlation of genome-wide estimates of Tajima's *D* values between species, but have a lesser impact on correlations of genetic diversity and differentiation between species. The significant influence of demographic history on correlation of Tajima's *D* may be because Tajima's *D* is particularly influenced by the site frequency spectrum of rare alleles, which is sensitive to recent changes in population size (Tajima 1989; Depaulis et al. 2003). By contrast, other parameters, such as π, *F*_ST_, and *d*_XY_, are likely less influenced by population fluctuations in the *Castanopsis* species analysed.

### Positive Selection Contributed to the Formation of Correlated Differentiation Landscape in *Castanopsis*

Although linked selection has influenced the patterns of genomic variation in the *Castanopsis* species, it remains unclear whether this is primarily due to BGS or recurrent SW. To understand the relative contributions of BGS and SW in the formation of correlated genomic landscapes between *Castanopsis* species, we investigated the temporal dynamics of genomic landscape correlation throughout the continuum of species divergence. Under a scenario of BGS, genetic diversity (π) is expected to consistently correlate with both genomic features and absolute genetic divergence (*d*_XY_) since species split, while the correlation between *F*_ST_ and genomic features would be low (or even nonexistent) at the beginning of divergence and increase with divergence time (Burri 2017b). Consistent with this expectation, we observed strong correlations between π and genomic features (gene density and recombination rate) throughout the divergence time. We also found that the correlations between *F*_ST_ and both π and gene density increased significantly with increasing differentiation time. However, contrary to Burri's prediction (Burri 2017b), the correlation between π and *d*_XY_ decreased rapidly with increased divergence time, suggesting that BGS alone cannot fully account for the similar patterns of genomic variation we observed between *Castanopsis* species.

Recurrent SW may be responsible for driving the evolution of genomic variation landscapes in *Castanopsis* species. We discovered negative correlations between *F*_ST_ and *d*_XY_ among recently diverged species, and positive correlations between these two parameters among species that have undergone a long period of divergence. Additionally, *d*_XY_ was comparable to genetic diversity (π) for recently diverged species, but much higher than π in anciently diverged species (supplementary tables S3 and S4, Supplementary Material online). These results align with the expectations of a scenario where SW shape genomic variation during species divergence (Chase et al. 2021). During the initial stages of divergence, *d*_XY_ between descendent species primarily reflects the level of genetic diversity inherited from their ancestral species. Consequently, recurrent SW that reduce genetic diversity in the ancestral population lead to a negative correlation between *d*_XY_ and *F*_ST_. However, as species divergence progresses, substitutions become a more prominent contributor to *d*_XY_, relative to ancestral polymorphisms, resulting in a positive correlation between *d*_XY_ and *F*_ST_. Similar changes in the relationship between *d*_XY_ and *F*_ST_, due to a shift in the relative contribution of inherited polymorphisms and substitutions, were reported in flycatchers (Chase et al. 2021) and oaks (Shi et al. 2024).

If SW has contributed to the formation of a correlated differentiation landscape, it is necessary to assume that positive selection has acted on the same genomic regions across multiple species. These conditions may be plausible. Recent studies have revealed that the genetic structure of adaptive traits is highly polygenic and mainly contributed by ancient polymorphisms (Barrett and Schluter 2008; Barghi et al. 2020; Bock et al. 2023). Therefore, positive selection may repeatedly impact the same genomic regions over long evolutionary time scales, leading to similar genomic variation landscapes among species. Importantly, parallel selection may not necessarily target identical genes or exert influence in a uniform direction. Rather, selection operating on analogous regions is likely to produce similar effects, such as reduced genetic diversity within species and elevated genetic differentiation between species. The geographical distribution patterns of the *Castanopsis* species studied, whether sympatric or parapatric, suggest that they inhabit similar ecological niches and may face similar selective pressure. Therefore, it is highly likely that positive selection has acted on the same sets of genes across multiple *Castanopsis* species, resulting in correlated landscapes of genomic variation among these species. Previous studies have also suggested that SW contributes to the development of correlated genomic variation landscapes in birds and monkeyflowers (Irwin et al. 2016; Van Doren et al. 2017; Delmore et al. 2018; Stankowski et al. 2019). Future studies to determine the genomic basis of adaptive traits and the role of such genomic parallels in driving local adaptation and genomic divergence will be of particular interest.

### The Role of Gene Flow in Shaping Patterns of Genomic Variation

Gene flow may have played a significant role in the evolution of the genomic variation landscape in *Castanopsis* species. We found prevalent introgression between *Castanopsis* species, affecting 0.1% to 6.4% of genomic regions, with the portion of genome affected by gene flow reducing with increased divergence time between hybridizing species. This finding supports the expectation that isolating barriers gradually accumulate after speciation, resulting in decreasing gene flow across an increasing number of genomic regions (Matute et al. 2010). The pattern of introgression may also be influenced by species distribution, with greater gene flow expected between species that have a higher degree of overlap in their distribution ranges. Additionally, the co-occurrence of species may vary across different geographical regions, potentially affecting interspecific hybridization patterns. In this study, the 12 *Castanopsis* species exhibited significant overlap in their distribution ranges, which is consistent with the observed high gene flow among them. However, it remains unclear whether certain species are more frequently found together in mixed forests and exhibit higher gene flow in specific geographic regions. Future studies with detailed surveys within the distribution ranges of these species will be crucial for uncovering population-level introgression patterns, their relationship with species distributions, and their impacts on ecological traits.

We observed a positive correlation between admixture proportion (*f*_dM_) and genetic diversity (π), as well as a negative correlation between *f*_dM_ and genetic differentiation (*F*_ST_ and *d*_XY_) across the genome. Additionally, *f*_dM_ was positively correlated with recombination rate and negatively correlated with gene density. These results are consistent with the expectation of selection against gene flow, in which purifying selection eliminates introgression fragments that contain deleterious or maladaptive variants from admixture populations (Moran et al. 2021). During this process, regions with high recombination rates would exhibit higher rates of admixture because low linkage disequilibrium between mutations allows neutral and beneficial mutations to escape the effects of selection (Schumer et al. 2018; Martin et al. 2019). Furthermore, regions with low gene density are also expected to display higher levels of introgression, since mutations in these areas are less likely to be deleterious (Calfee et al. 2021; Feng et al. 2023).

Adaptive introgression is another important process shaping the genomes of hybridizing species. During this process, advantageous alleles are introduced by hybridization and subsequently spread and fixed across species by positive selection, resulting in a high proportion of foreign ancestry in the regions surrounding the adaptive alleles (Martin and Jiggins 2017; Moran et al. 2021). Consistent with these findings, we found that introgressed regions showed stronger SW signals, including elevated haplotype homozygosity and reduced genetic differentiation between donor and recipient species. We also observed significant enrichment of disease resistance genes in the introgressed regions of 11 trios, consistent with previous studies in grape (Morales-Cruz et al. 2021) and *Arabidopsis* (Bechsgaard et al. 2017), which suggests that resistance gene families serve as hot spots for introgression, possibly because their high levels of polymorphism can provide advantages in terms of disease resistance. It is important to note that current population genetic methods generally lack sufficient power to detect recurrent soft sweeps in scenarios involving complex demographic histories (Harris et al. 2018). Furthermore, neither the outlier SNPs nor the enriched resistance genes found in introgressed regions have been validated through molecular functional analyses. Direct evidence supporting adaptive introgression could be obtained in future studies by applying plant molecular biology approaches to investigate the genomic basis of ecological and functional traits in *Castanopsis* species.

Adaptive introgression may also occur when introduced foreign fragments present a low genetic burden. Natural selection is more efficient at removing deleterious mutations in species with larger *N*_e_, resulting in a reduced genetic load relative to species with smaller *N*_e_ (Gossmann et al. 2010; Ellegren and Galtier 2016). Consequently, introgression from large *N*_e_ species would reduce genetic burden in the introgression region, and vice versa (Harris and Nielsen 2016; Wang et al. 2017; Kim et al. 2018). In accordance with this expectation, there is evidence of reduced genetic load of deleterious mutations in introgression regions in poplar (Liu et al. 2022) and maize (Wang et al. 2017), while elevated genetic burden was detected in introgression regions in wine grape (Xiao et al. 2023). In *Castanopsis* species, we observed both reduced and elevated genetic load in introgressed regions compared with genomic background in various comparisons. This pattern held true even for hybridizing species with substantial differences in *N*_e_. The accumulation of deleterious mutations in the introgression regions of *Castanopsis* may be explained by a dominance of gene flow from species with small *N*_e_ into those with large *N*_e_. However, this is unlikely because purifying selection in species with large *N*_e_ would effectively remove such introgressed fragments from species with small *N*_e_ (Martin and Jiggins 2017; Moran et al. 2021). Alternatively, the high genetic burden in introgression regions may be due to complex interactions between deleterious and beneficial mutations. For example, the fitness impacts of deleterious mutations may be offset by compensatory mutations (Gan et al. 2011), and deleterious alleles may also be driven to high frequency by genetic hitchhiking (Hartfield and Otto 2011; Marsden et al. 2016; Zhang et al. 2016). Investigations to clarify the effects of different evolutionary factors on the accumulation of deleterious mutations in hybridizing species are warranted.

In conclusion, our study revealed remarkable similarities in the patterns of genomic variation among 12 *Castanopsis* species. These similarities are likely due to long-term linked selection and the presence of conserved genomic features across millions of years and multiple speciation events. Both BGS and SW appear to have influenced the landscapes of genomic diversity and differentiation. Our findings also uncovered widespread hybridization between *Castanopsis* species, and demonstrated that both selection against gene flow and adaptive introgression have played fundamental roles in molding the patterns of genomic variation. Our results point to the need to explore the dynamically evolving correlations of genomic variation landscapes over a broad divergence continuum, and analyse how various selection models, genomic features, and population demographics jointly influence genomic variation.

## Materials and Methods

### Plant Material Collection and Genome Sequencing

A healthy mature tree of *C. eyrei* located in the Xiangtou Mountain Natural Reserve (latitude 23.276°N, longitude 114.369°E) was selected for genome sequencing and *de novo* assembly. Fresh leaves and flowers harvested from the selected tree were rapidly frozen in liquid nitrogen and then stored at −80 °C. Genomic DNA was extracted from the collected leaves using a DNeasy Plant MiniKit (Qiagen, Germany) and sequenced as follows: (i) a 350-bp Illumina library was sequenced on the NovaSeq 6000 platform (Illumina, USA) to generate 150-bp paired-end reads; (ii) a 20 kb SMART library was constructed using the PacBio SMRTbell Template Prep Kit 4.0 V2 (Pacific Biosciences, USA) and sequenced on the PacBio Sequel system; (iii) a Hi-C sequencing library was prepared and sequenced on the Illumina NovaSeq 6000 platform (150-bp paired-end reads).

Total RNA was isolated from both leaves and flowers using the RNAprep Pure Plus Kit (Tiangen, China) and purified using poly-T oligo-attached magnetic beads. The resultant complementary DNA library was sequenced with paired-end (150 bp) reads on the Illumina NovaSeq 6000 platform.

### Genome Survey and *de novo* Assembly

To estimate the genome size of *C. eyrei*, 17-bp *K*-mers among Illumina clean reads were counted using Jellyfish v1.1.11 (Marçais and Kingsford 2011), with default settings. For genome assembly, corrected PacBio long reads were used to construct a preliminary assembly within Falcon v0.3.0 (Chin et al. 2016). This initial assembly was polished using pilon v1.22 (Walker et al. 2014) with Illumina short reads. Hi-C reads were mapped to the assembly using BWA v0.7.15 (Li and Durbin 2010), and PCR duplications were removed using samtools v1.8 (Danecek et al. 2021). A chromosome-level genome assembly was generated using LACHESIS (Burton et al. 2013) and JuiceBox (Durand et al. 2016), leveraging the Hi-C read mapping results. The integrity and completeness of the assembled genome were evaluated using BUSCO pipeline (Simão et al. 2015).

### Genome Annotation

A combined strategy that integrated both homology-based and *de novo* approaches was employed to characterize the repetitive sequences in the *C. eyrei* genome. Initially, a comprehensive repeat database was constructed from the assembled genome using RepeatModeler v2.0.1 (Flynn et al. 2020), LTR_FINDER v1.0.7 (Xu and Wang 2007) and RepeatScout v1.0.5 (Price et al. 2005). This database was further combined with REPBASE (Jurka et al. 2005), and repetitive elements then identified using RepeatMasker v4.07 (Tarailo-Graovac and Chen 2009). Additionally, RepeatProteinMask v4.07 (Tarailo-Graovac and Chen 2009) was used to detect repeat sequences based on the protein version of REPBASE, and Tandem Repeats Finder v4.09 (Benson 1999) was applied to identify tandem repeats.

A multi-faceted approach that encompassed homology-based, ab initio, and transcript-based predictions was implemented to annotate *C. eyrei* protein-coding genes. For homology-based prediction, protein sequences of five representative species were downloaded from GenBank, including *Quercus robur* (Bodénès et al. 2016), *Quercus lobata* (Sork et al. 2016), *Fagus sylvatica* (Mishra et al. 2018), *Arabidopsis thaliana* (Michael et al. 2018), and *Juglans regia* (Martínez-García et al. 2016). Then, TBLASTN v2.2.26 (Yu et al. 2006) was applied to blast these sequences against the *C. eyrei* genome. An E-value cutoff of 1e^−5^ was used to identify homologous sequences. Subsequently, *C. eyrei* gene models were predicted using GeneWise v2.4.1 (Birney et al. 2004), based on these alignments. For ab initio prediction, coding regions were predicted using a suite of five software tools: AUGUSTUS v3.2.3 (Stanke et al. 2006), Geneid v1.4 (Alioto et al. 2018), GENSCAN v1.0 (Burge and Karlin 1997), GlimmerHMM v3.04 (Majoros et al. 2004), and SNAP v2013-11-29 (Korf 2004). For transcript-based prediction, a *C. eyrei* transcriptome was assembled using Trinity v2.1.1 (Grabherr et al. 2011), Hisat v2.0.4 (Kim et al. 2015), and Stringtie v1.3.3 (Pertea et al. 2015). The protein-coding genes predicted by the three approaches were merged into a non-redundant gene-set using EvidenceModeler v1.1.1 (Haas et al. 2008) and further improved using the PASAv2.02 pipeline (Haas et al. 2003). The completeness of these gene models was evaluated using BUSCO (Simão et al. 2015).

To infer the functions of predicted genes, searches were conducted against multiple databases, including the NCBI non-redundant (nr) protein database, Swiss-Prot, the Kyoto Encyclopedia of Genes and Genomes, and the protein family database. Domain architectures were defined using InterProScan v5.31 (Mulder and Apweiler 2007) and GO terms assigned based on corresponding entries in the InterPro database.

Non-coding genes in the *C. eyrei* genome were also annotated, and tRNAs predicted using tRNAscan-SE v1.4 (Lowe and Eddy 1997), while other non-coding RNAs, such as miRNAs and snRNAs, were identified by searches against the Rfam database (Griffiths-Jones et al. 2005) using INFERNAL v1.1.2 (Nawrocki 2014), with default parameters.

### Collinearity Analysis

To assess the collinearity among *Castanopisis* species, we aligned the genomes of *C. hystrix* (Huang et al. 2023) and *C. tibetana* (Sun et al. 2022) with that of *C. eyrei* using minimap2 (Li 2018). Alignments were then sorted using Samtools v1.8 (Danecek et al. 2021). Synteny blocks and structural rearrangements among the three *Castanopsis* genomes were analysed with SyRi v1.6 (Goel et al. 2019), and visualized using plotsr (Goel and Schneeberger 2022). To further evaluate the similarity in gene density landscapes among these three *Castanopsis* genomes, we identified collinear blocks based on protein sequences using MCScan, implemented in jcvi v1.2.7 (Tang et al. 2008). We then calculated the correlation of gene density across corresponding 500 kb windows for each pair of species.

### Population Sampling, Whole-genome Re-sequencing, and SNP Calling

Individuals (*N* = 267) were collected from 12 *Castanopsis* species, including: 21 *C. carlesii*; 25 *C. fargesii*; 25 *C. eyrei*; 24 *C. lamontii*; 28 *C. fabri*; 19 *C. hystrix*; 20 *C. fordii*; 26 *C. tibetana*; 10 *C. chinensis*; 23 *C. sclerophylla*; 24 *C. jucunda*; and 22 *C. fissa* (supplementary table S1, Supplementary Material online). For each individual, genomic DNA was extracted from silica-dried leaves using a Plant DNA Kit (Bioteke, Beijing, China) and sequenced on the Illumina NovaSeq 6000 platform (150-bp paired-end reads) with a target coverage of 30×.

Raw sequencing data were cleaned using Trimmomatic v.0.38 (Bolger et al. 2014) to remove low quality sequences. Cleaned reads were then aligned to the *C. eyrei* reference genome using BWA v.0.7.15 (Li and Durbin 2010). All individuals included in this study exhibited a high mapping rate (90.26% to 98.32%), with a relatively low mapping rate appearing to be individual-specific rather than species-specific (supplementary table S1, Supplementary Material online and Fig S20), suggesting that there is no species-specific bias due to divergence from the reference. These results suggested that the effects of reference bias were likely minimal in this study.

Genotypes were called using HaplotypeCaller implemented in GATK v.4.1 (Depristo et al. 2011). To minimize bias in SNP and genotype calling, SNPs that met any of the following conditions were discarded: (1) located within repetitive regions of the *C. eyrei* reference genome; (2) more than two alleles present; (3) sequencing depth > 100 or < 5; (4) missing rate ≥ 0.3; (5) heterozygosity rate (proportion of heterozygotes among all genotypes) > 0.5; (6) indels. Additionally, only homozygous genotypes supported by ≥ 4 reads were considered. For heterozygous genotypes, the minor allele was required to be supported by ≥ 2 reads, and the read ratio (number of reads supporting the minor allele/number of reads supporting the major allele) was required to be > 0.1 and < 0.9. A total of 52,385,983 high-quality SNPs were retained for data analysis and have been deposited in DRYAD (https://doi.org/10.5061/dryad.kkwh70scm).

### Population Structure and Phylogenetic Analyses

A model-based clustering algorithm in ADMIXTURE v1.3.0 (Alexander et al. 2009) and PCA in EIGENSOFT v6.0 (Patterson et al. 2006) were applied to investigate the population structure of the 12 *Castanopsis* species. ADMIXTURE analysis was conducted with a range of predefined numbers of clusters (*K*) from 1 to 20, with each iteration running 20 times. The *K* value with the lowest cross-validation error was chosen as the most likely number of genetic clusters. Population structure analyses were conducted based on common SNPs with minor allele frequency >1%. To mitigate the influence of highly correlated SNPs, the dataset was pruned by randomly selecting one SNP every 5 kb, resulting in a set of 131,263 independent SNPs.

To further explore the phylogenetic relationships between species, maximum-likelihood (ML) trees were constructed using the GTR + Gamma model in IQtree2 v2.0.6 (Nguyen et al. 2015) with *Castanea mollissima* as an outgroup. ML trees were constructed based on SNPs within non-overlapping windows of 10, 100, and 500 kb, as well as genome-wide SNPs. To streamline computing time, we randomly selected 1,000,000 SNPs across the genome for constructing the genome-wide trees and repeated this process five times. The resulting trees displayed highly similar topologies, and one was selected for further analyses. To assess the extent of phylogenetic incongruence across the genome, tree concordance was computed by comparing the genome-wide tree with window-based trees (10, 100, and 500 kb). These phylogenetic trees were transformed into distance matrices using the Ape package in R (Paradis et al. 2004). Then, correlation coefficient values were calculated to quantify the correspondence between each window-based distance matrix and that of the whole-genome tree, using Mantel tests. To determine whether variations in tree concordance were randomly distributed across the genome, the autocorrelation of tree concordance with genomic position was evaluated and the significance assessed using 1,000 random permutations.

### Demographic History Analyses

To track the temporal fluctuations in effective population size (*N*_e_) for each species, MSMC was implemented in MSMC v2.0.0 (Schiffels and Durbin 2014). Genotypes were phased using Beagle v4.1 (Browning and Browning 2009) with default settings. MSMC analyses were performed on 50 combinations of haplotypes (four haplotypes per species) to calculate their mean and standard deviation values. To convert the demographic inferences into absolute values, we calculated the mutation rate (μ) in *Castanopsis* species using the formula μ = *d*/2*T* (Nei 1987), where *d* and *T* represent the genetic divergence and divergence time between species. By aligning the genome assemblies of *C. eyrei* and *Castanea mollissima* (Xing et al. 2019), we determined the genetic divergence between these two species to be 0.0857. Assuming a divergence time (*T*) of 52.2 MYA between the genera *Castanopsis* and *Castanea* (Wilf et al. 2019), we calculated a mutation rate of 8.21 × 10^−10^ per site per year for *Castanopsis*. This estimate closely aligns with a recent finding in other Fagaceae species (Yuan et al 2023). Further, we utilized a generation time of 100 yr, as proposed for *Castanopsis* species in a previous study (Aoki et al. 2019).

### Estimation of Population Genetic Statistics

To investigate patterns of genomic variation within and between the 12 *Castanopsis* species, nucleotide diversity (π) (Nei 1987) and Tajima's *D* (Tajima 1989) were calculated within each species, alongside relative genetic differentiation (*F*_ST_) (Weir and Cockerham 1984) and absolute genetic divergence (*d*_XY_) (Nei 1987) between each pair of species. Further, *d*_XY_ between a pair of species from the genus *Quercus*, *Quercus dentata* and *Quercus variabilis*, which diverged from *Castanopsis* more than 60 MYA (Zhou et al. 2022a), was also calculated. To do this, whole-genome re-sequencing data of 20 *Q. dentata* and 20 *Q. variabilis* (supplementary table S1, Supplementary Material online) were extracted from previous studies (Liang et al. 2022; Zhou et al. 2022b). Genome-wide SNPs were called by aligning reads to the *C. eyrei* reference genome, as described above. All summary statistics were estimated in non-overlapping windows of 10, 100, and 500 kb across the genome. Values of π, Tajima's *D*, and *F*_ST_ were estimated using ANGSD (Korneliussen et al. 2014), and *d*_XY_ was calculated using a Perl script developed by Nagarjun Vijay (https://github.com/mfumagalli/ngsPopGen). To account for uncertainty in genotype calls, these statistics were estimated based on genotype likelihoods.

The population-scaled recombination rate (ρ = 4*N*_e_c) was also estimated for each species using LDHELMET v.1.9 (Chan et al. 2012) with default parameters (100,000 burn-in iterations, 1,000,000 Markov chain iterations, and a block penalty of 50), and the estimated ρ between each pair of SNPs was averaged over non-overlapping windows. To mitigate the effects of rare variants, only SNPs with MAF >5% were included. Gene density was estimated as the total length of coding sequences within each window.

Following Stankowski et al. (2019), PCA was employed to summarize the variance in each statistic derived from various pairwise comparisons. To evaluate the null hypothesis that the observed genome-wide patterns of these statistics were generated by stochastic processes, an autocorrelation analysis was performed, as applied to analysis of tree concordance (see “Population structure and phylogenetic analyses”). To elucidate the dynamics of correlation between population genetic statistics over divergence time, correlation coefficients between seven distinct pairs of statistics were examined, specifically: π versus gene density, π versus ρ, π versus *F*_ST_, π versus *d*_XY_, *F*_ST_ versus gene density, *F*_ST_ versus ρ, and *F*_ST_ versus *d*_XY_. Sequence divergence (*d*_a_), computed as *d*_a_ = *d*_XY_—mean π, was employed as an approximate measure of the divergence time between each species-pair (Nei and Li 1979; Burri 2017b). To mitigate the issue of non-independence between pairwise comparisons (e.g. *F*_ST_, *d*_XY_, and *d*_a_), we conducted a phylogenetic correction approach (Felsenstein 1985; Coyne and Orr 1989), and derived 11 representative contrasts based on the phylogenetic tree of the 12 species (Fig. 1b). For each contrast, we averaged estimates of all species pairs spanning the contrast to obtain a single estimate. As π and ρ were estimated within each species, mean π and ρ were derived for interspecific comparisons (e.g. π vs. *F*_ST_ and π vs. *d*_XY_).

### Assessing Genome-wide Introgression Patterns

To investigate gene flow between the 12 *Castanopsis* species, Dsuite (Malinsky et al. 2021) was used to calculated the *D*-statistic and *f*_4_ admixture ratio (*f*_4_-ratio) for 220 valid trios extracted from the phylogenetic tree, with *Castanea mollissima* as the outgroup. The *D*-statistic and *f*_4_-ratio were measured based on a four-taxon framework (((P1, P2), P3), O), where P1, P2, and P3 are the ingroups and O represents the outgroup, as described by Patterson et al. (2012). To calculate the *f*_4_-ratio in Dsuite, alleles from P3 were randomly split into two subsets, denoted P3a and P3b. The significance of the *D-*statistic was determined through block jackknifing, with adjustment for multiple comparisons using the Benjamini & Hochberg procedure (Benjamini and Hochberg 1995). The analysis revealed significant signals of gene flow in 178 trios (FDR <0.05, supplementary table S8, Supplementary Material online). As the same P2–P3 species-pairs were tested with different P1 species, the trio with the maximum *D-*statistic value was selected from each group to reduce redundancy. This criterion distilled the dataset to 51 distinct trios for further investigation. Additionally, to unravel the correlated *f*_4_-ratio results and accurately attribute specific gene flow events to distinct internal branches, the *f*-branch statistic (Malinsky et al. 2018) was calculated using Dsuite.

To identify introgression fragments between *Castanopsis* species, two window-based statistics, *f*_d_ and *f*_dM_, were calculated using a Python script (https://github.com/simonhmartin/genomics_general) in non-overlapping windows of 10, 100, and 500 kb. Following Morales-Cruz et al. (2021), candidate introgression regions were defined as those windows with the top X% of *f*_dM_ values, where the X% threshold was set based on the *f*_4_-ratio for each trio. To test whether any functional classes of genes were over-represented in introgression regions, GO enrichment analysis was conducted using GOWINDA (Kofler and Schlötterer 2012). The Benjamini-Hochberg FDR procedure was implemented for correction of multiple testing, and GO terms with FDR <0.05 were considered significantly enriched.

### Adaptive Introgression Testing

To assess adaptive introgression between *Castanopsis* species, evidence of SW was sought in each species using two haplotype-based statistics, the nSL (Ferrer-Admetlla et al. 2014) and the H12 (Garud et al. 2015). The H12 statistic was developed to identify both hard and soft SW (Garud et al. 2015; but see Harris et al. 2018), whereas the nSL though originally predicted using a hard selective model, can also identify soft sweeps (Ferrer-Admetlla et al. 2014). H12 was calculated using the SelectionHapStat tool (Garud et al. 2015), and nSL was estimated using selscan v1.3.0 (Szpiech and Hernandez 2014), based on SNPs with MAF > 0.05. To ascertain whether introgression regions exhibited stronger selection signals than the genomic background, average nSL and H12 values were computed across sliding windows. For each comparison, the genomic background was defined as windows lacking introgression signals involving any of the two species under consideration. To test whether the introgression regions were enriched for resistance genes, the RGAugury pipeline (Li et al. 2016) was used to identify RGAs in the *C. eyrei* genome, and then the enrichment of RGAs was examined in introgression regions using a hypergeometric test.

### Identification of Deleterious Mutations and Quantification of Genetic Burden

Two methods, PROVEAN v1.1.5 (Choi et al. 2012) and SIFT4G (Vaser et al. 2016), were employed to predict deleterious mutations. PROVEAN scores were calculated by assessing the sequence similarity between a query sequence and protein homologs in the NCBI nr database (Choi et al. 2012). SIFT4G was used to evaluate whether an amino acid substitution impacted protein function, based on the SIFT prediction algorithm (Vaser et al. 2016). As there was no SIFT database for any *Castanopsis* species, a database was generated using *C. eyrei* genes as models, following the instructions for SIFT4G (Vaser et al. 2016). To minimize reference bias, deleterious mutations were inferred using the ancestral allele, where ancestral allelic state was inferred using the outgroup species, *Castanea mollissima*. A nonsynonymous SNP was considered deleterious if it had a PROVEAN score ≤ −2.5 and a SIFT score ≥0.05, and as tolerant otherwise.

To evaluate the genetic burden of deleterious mutations in introgression regions, numbers of deleterious variants per individual were counted under both additive and recessive models. Derived alleles present in both heterozygous and homozygous states were counted under the additive model, whereas only homozygous alleles were considered under the recessive model. To normalize differences in sequencing quality between individuals, derived alleles at fourfold degenerate sites (serving as proxies for neutral variation) were counted, and the genetic burden defined as the ratio of deleterious to neutral variants for each genome. For each trio, the genetic burden of introgression regions was contrasted with that of the genomic background in P2 and P3 species independently.

## Supplementary Material

msae191_Supplementary_Data

## Data Availability

The reference genome and the whole genome re-sequencing data obtained in this study have been deposited in Genbank (under the accession number: PRJNA1097334 and PRJNA1097337) and NGDC (under accession number: PRJCA026947 and PRJCA026948). The SNPs and custom scripts have been deposited in DRYAD (https://doi.org/10.5061/dryad.kkwh70scm).
