# Supplementary material for: Evolution of the Correlated Genomic Variation Landscape Across a Divergence Continuum in the Genus Castanopsis
Source: Mol Biol Evol. 2024 Sep 9;41(9):msae191. doi: 10.1093/molbev/msae191 (PMC11421576; doi:10.1093/molbev/msae191)
Supplement: msae191_Supplementary_Data [file msae191_supplementary_data.pdf]

Table S1 Geographic locations, average sequencing coverage, and mapping rates for the 267 individuals of 12 *Castanopsis* species sequenced in this study, along with 40 individuals of two oak species. Sample ID: A unique tag assigned to each individual to track their genome sequencing data in Genbank. Each row represents one individual sequenced in this or previous studies.

Sample ID	Species	Latitude (°N)	Longitude (°E)	Location (Population)	Genetic group within species	Mean coverage	Mapping rate	Origin
BDSW190632123-1a+C.carl_PJX_9-1-1_L2	*C. carlesii*	28.5980	113.9084	Pingjiang ,Yueyang, Hunan (PJX)	Ccar-1	29.83	97.56%	This study
BDSW190632124-1a+C.carl_PJX_9-2-1_L1	*C. carlesii*	28.5981	113.9086	Pingjiang ,Yueyang, Hunan (PJX)	Ccar-1	29.11	97.80%	This study
BDSW190632126-1a+C.carl_PJX_9-4-1_L3	*C. carlesii*	28.5919	113.9271	Pingjiang ,Yueyang, Hunan (PJX)	Ccar-1	29.29	96.65%	This study
C.carl_PJX_9-3_BDSW190632125-1a	*C. carlesii*	28.5983	113.9090	Pingjiang, Yueyang , Hunan (PJX)	Ccar-1	29.83	97.53%	This study
BDSW190632133-1a+C.carl_PNX_4-3-1_L1	*C. carlesii*	26.9514	119.1517	Pingnan, Ningde, Fujian (PNX)	Ccar-1	30.87	90.51%	This study
BDSW190632134-1a+C.carl_PNX_4-5-1_L1	*C. carlesii*	26.9511	119.1510	Pingnan, Ningde, Fujian (PNX)	Ccar-1	33.35	95.73%	This study
BDSW190632135-1a+C.carl_PNX_4-7-1_L1	*C. carlesii*	26.9525	119.1514	Pingnan, Ningde, Fujian (PNX)	Ccar-1	29.67	97.28%	This study
C.carl_PNX_4-9_BDSW190632136-1a	*C. carlesii*	26.9155	119.1989	Pingnan, Ningde, Fujian (PNX)	Ccar-1	29.12	96.90%	This study
C.carl_LSX_2-1_BDSW192012418-1a	*C. carlesii*	26.3023	108.0580	Leishan, Guizhou (LSX)	Ccar-2	33.48	97.12%	This study
C.carl_LSX_2-2_BDSW192012419-1a	*C. carlesii*	26.2687	108.1652	Leishan, Guizhou (LSX)	Ccar-2	32.94	96.35%	This study
C.carl_LSX_2-3_BDSW192012420-1a	*C. carlesii*	26.2671	108.1655	Leishan, Guizhou (LSX)	Ccar-2	29.39	96.86%	This study
C.carl_PGQ_6-1_BDSW192012428-1a	*C. carlesii*	24.6312	111.5277	Pinggui, Hezhou, Guangxi (PGQ)	Ccar-2	33.51	97.86%	This study
C.carl_PGQ_6-3_BDSW192012429-1a	*C. carlesii*	24.6333	111.5246	Pinggui, Hezhou, Guangxi (PGQ)	Ccar-2	34.37	96.63%	This study
C.carl_PGQ_6-5_BDSW192012430-1a	*C. carlesii*	24.6337	111.5238	Pinggui, Hezhou, Guangxi (PGQ)	Ccar-2	30.96	97.72%	This study
C.carl_PGQ_6-7_BDSW192012431-1a	*C. carlesii*	24.6350	111.5226	Pinggui, Hezhou, Guangxi (PGQ)	Ccar-2	30.29	96.98%	This study
C.carl_PGQ_6-9_BDSW192012432-1a	*C. carlesii*	24.6358	111.5211	Pinggui, Hezhou, Guangxi (PGQ)	Ccar-2	33.82	97.93%	This study
C.carl_NPX_6-1_BDSW192012421-1a	*C. carlesii*	23.2939	105.8006	Napo, Baise, Guangxi (NPX)	Ccar-3	32.13	92.83%	This study
C.carl_NPX_6-2_BDSW192012422-1a	*C. carlesii*	23.2949	105.7997	Napo, Baise, Guangxi (NPX)	Ccar-3	33.91	94.44%	This study
C.carl_NPX_6-3_BDSW192012423-1a	*C. carlesii*	23.2950	105.7996	Napo, Baise, Guangxi (NPX)	Ccar-3	29.00	92.11%	This study
C.carl_NPX_6-4_BDSW192012424-1a	*C. carlesii*	23.2951	105.7980	Napo, Baise, Guangxi (NPX)	Ccar-3	29.87	92.64%	This study
C.carl_NPX_6-6_BDSW192012425-1a	*C. carlesii*	23.2933	105.7946	Napo, Baise, Guangxi (NPX)	Ccar-3	32.38	97.37%	This study
BDSW190632151-1a+C.chin_CHQ_4-1-1_L2	*C. chinensis*	23.7739	113.9306	Conghua, Guangzhou, Guangdong (CHQ)	Cchi-1	26.52	97.32%	This study
BDSW190632152-1a+C.chin_CHQ_4-3-1_L2	*C. chinensis*	23.7746	113.9320	Conghua, Guangzhou, Guangdong (CHQ)	Cchi-1	28.21	95.85%	This study
BDSW190632153-1a+C.chin_CHQ_4-5-1_L1	*C. chinensis*	23.7847	113.9958	Conghua, Guangzhou, Guangdong (CHQ)	Cchi-1	30.28	97.53%	This study
BDSW190632154-1a+C.chin_CHQ_4-7-1_L1	*C. chinensis*	23.7854	113.9988	Conghua, Guangzhou, Guangdong (CHQ)	Cchi-1	26.77	97.04%	This study
BDSW190632155-1a+C.chin_CHQ_4-9-1_L1	*C. chinensis*	23.8461	114.0094	Conghua, Guangzhou, Guangdong (CHQ)	Cchi-1	30.65	95.23%	This study
BDSW190632156-1a+C.chin_DHS_2-1-1_L1	*C. chinensis*	23.1724	112.5398	Dinghu, Zhaoqing, Guangdong (DHS)	Cchi-2	28.17	97.60%	This study
BDSW190632157-1a+C.chin_DHS_2-3-1_L3	*C. chinensis*	23.1725	112.5390	Dinghu, Zhaoqing, Guangdong (DHS)	Cchi-2	32.23	96.42%	This study
BDSW190632158-1a+C.chin_DHS_2-5-1_L1	*C. chinensis*	23.1723	112.5390	Dinghu, Zhaoqing, Guangdong (DHS)	Cchi-2	34.70	96.79%	This study
BDSW190632159-1a+C.chin_DHS_2-7-1_L2	*C. chinensis*	23.1726	112.5399	Dinghu, Zhaoqing, Guangdong (DHS)	Cchi-2	28.25	96.91%	This study
BDSW190632160-1a+C.chin_DHS_2-9-1_L2	*C. chinensis*	23.1742	112.5400	Dinghu, Zhaoqing, Guangdong (DHS)	Cchi-2	28.37	97.31%	This study
C.eyre_BLX_9-1_BDSW190635368-1a	*C. eyrei*	23.2643	114.3770	Boluo, Huizhou, Guangdong (BLX)	Ceyr-1	26.21	98.14%	This study
C.eyre_BLX_9-10_BDSW192000269-1a	*C. eyrei*	23.2643	114.3770	Boluo, Huizhou, Guangdong (BLX)	Ceyr-1	28.78	97.85%	This study
C.eyre_BLX_9-13_BDSW192000270-1a	*C. eyrei*	23.2643	114.3770	Boluo, Huizhou, Guangdong (BLX)	Ceyr-1	30.11	97.36%	This study
C.eyre_BLX_9-4_BDSW190635369-1a	*C. eyrei*	23.2643	114.3770	Boluo, Huizhou, Guangdong (BLX)	Ceyr-1	25.56	97.63%	This study
C.eyre_BLX_9-7_BDSW190635370-1a	*C. eyrei*	23.2643	114.3770	Boluo, Huizhou, Guangdong (BLX)	Ceyr-1	30.63	97.88%	This study
C.eyre_PNX_3-10_BDSW190635337-1a	*C. eyrei*	26.9099	119.1957	Pingnan, Ningde, Fujian (PNX)	Ceyr-1	28.38	0.972	This study
C.eyre_PNX_3-2_BDSW190635333-1a	*C. eyrei*	26.9519	119.1515	Pingnan, Ningde, Fujian (PNX)	Ceyr-1	23.98	97.16%	This study
C.eyre_PNX_3-4_BDSW190635334-1a	*C. eyrei*	26.9510	119.1510	Pingnan, Ningde, Fujian (PNX)	Ceyr-1	24.86	97.44%	This study
C.eyre_PNX_3-6_BDSW190635335-1a	*C. eyrei*	26.9507	119.1514	Pingnan, Ningde, Fujian (PNX)	Ceyr-1	24.15	96.79%	This study
C.eyre_PNX_3-8_BDSW190635336-1a	*C. eyrei*	26.9541	119.1509	Pingnan, Ningde, Fujian (PNX)	Ceyr-1	24.26	97.51%	This study
C.eyre_QMX_1-11_BDSW190635361-1a	*C. eyrei*	30.0198	117.5404	Qimen, Huangshan, Anhui (QMX)	Ceyr-1	23.70	97.86%	This study
C.eyre_QMX_1-13_BDSW190635362-1a	*C. eyrei*	30.0141	117.5317	Qimen, Huangshan, Anhui (QMX)	Ceyr-1	22.56	97.21%	This study
C.eyre_QMX_1-3_BDSW190635358-1a	*C. eyrei*	30.0430	117.5621	Qimen, Huangshan, Anhui (QMX)	Ceyr-1	28.11	0.979	This study
C.eyre_QMX_1-5_BDSW190635359-1a	*C. eyrei*	30.0398	117.5587	Qimen, Huangshan, Anhui (QMX)	Ceyr-1	25.30	97.52%	This study
C.eyre_QMX_1-9_BDSW190635360-1a	*C. eyrei*	30.0271	117.5485	Qimen, Huangshan, Anhui (QMX)	Ceyr-1	25.40	96.59%	This study
C.eyre_NYQ_1-1_BDSW190635363-1a	*C. eyrei*	27.2795	112.6737	Nanyue, Hengyang, Hunan (NYQ)	Ceyr-2	26.61	97.69%	This study
C.eyre_NYQ_1-3_BDSW190635364-1a	*C. eyrei*	27.2775	112.6728	Nanyue, Hengyang, Hunan (NYQ)	Ceyr-2	26.27	97.45%	This study
C.eyre_NYQ_1-5_BDSW190635365-1a	*C. eyrei*	27.2763	112.6723	Nanyue, Hengyang, Hunan (NYQ)	Ceyr-2	29.04	94.76%	This study
C.eyre_NYQ_1-7_BDSW190635366-1a	*C. eyrei*	27.2767	112.6721	Nanyue, Hengyang, Hunan (NYQ)	Ceyr-2	24.95	96.73%	This study
C.eyre_NYQ_1-8_BDSW190635367-1a	*C. eyrei*	27.2768	112.6722	Nanyue, Hengyang, Hunan (NYQ)	Ceyr-2	27.93	96.98%	This study
C.eyre_SMZ_6-1_BDSW192012385-1a	*C. eyrei*	25.6464	109.9014	Sanmen, Guilin , Guangxi (SMZ)	Ceyr-3	29.04	96.92%	This study
C.eyre_SMZ_6-3_BDSW192012386-1a	*C. eyrei*	25.6446	109.9016	Sanmen, Guilin , Guangxi (SMZ)	Ceyr-3	30.11	96.51%	This study
C.eyre_SMZ_6-5_BDSW192012387-1a	*C. eyrei*	25.6379	109.9074	Sanmen, Guilin , Guangxi (SMZ)	Ceyr-3	37.00	95.43%	This study
C.eyre_SMZ_6-7_BDSW192012388-1a	*C. eyrei*	25.6378	109.9072	Sanmen, Guilin , Guangxi (SMZ)	Ceyr-3	38.48	97.34%	This study
C.eyre_SMZ_6-9_BDSW192012389-1a	*C. eyrei*	25.6355	109.9081	Sanmen, Guilin , Guangxi (SMZ)	Ceyr-3	33.99	96.49%	This study
C.fabr_NPX_4-1_BDSW192017307-1a	*C. fabri*	23.3069	105.8029	Napo, Baise, Guangxi (NPX)	Cfab-1	38.08	97.26%	This study
C.fabr_NPX_4-3_BDSW192017308-1a	*C. fabri*	23.2959	105.8014	Napo, Baise, Guangxi (NPX)	Cfab-1	32.29	97.24%	This study
C.fabr_NPX_4-5_BDSW192017309-1a	*C. fabri*	23.2953	105.7995	Napo, Baise, Guangxi (NPX)	Cfab-1	32.93	95.17%	This study
C.fabr_NPX_4-7_BDSW192017310-1a	*C. fabri*	23.2943	105.7960	Napo, Baise, Guangxi (NPX)	Cfab-1	34.34	97.23%	This study
C.fabr_NPX_4-8_BDSW192017311-1a	*C. fabri*	23.2932	105.7946	Napo, Baise, Guangxi (NPX)	Cfab-1	32.76	97.65%	This study
C.fabr_BLX_7-1_BDSW192000281-1a	*C. fabri*	23.2687	114.3699	Boluo, Huizhou, Guangdong (BLX)	Cfab-2	29.51	96.91%	This study
C.fabr_BLX_7-2_BDSW192000282-1a	*C. fabri*	23.2687	114.3699	Boluo, Huizhou, Guangdong (BLX)	Cfab-2	41.48	97.60%	This study
C.fabr_BLX_7-3_BDSW192000283-1a	*C. fabri*	23.2687	114.3699	Boluo, Huizhou, Guangdong (BLX)	Cfab-2	35.14	97.52%	This study
C.fabr_BLX_7-4_BDSW192000284-1a	*C. fabri*	23.2687	114.3699	Boluo, Huizhou, Guangdong (BLX)	Cfab-2	31.84	97.75%	This study
C.fabr_PNX_11-2_BDSW192000276-1a	*C. fabri*	26.8963	118.7542	Pingnan, Ningde, Fujian (PNX)	Cfab-2	31.60	96.66%	This study
C.fabr_PNX_11-4_BDSW192000277-1a	*C. fabri*	26.9260	118.7134	Pingnan, Ningde, Fujian (PNX)	Cfab-2	30.73	95.07%	This study
C.fabr_PNX_11-5_BDSW192000278-1a	*C. fabri*	26.9285	118.7134	Pingnan, Ningde, Fujian (PNX)	Cfab-2	28.91	96.15%	This study
C.fabr_PNX_11-7_BDSW192000279-1a	*C. fabri*	26.9311	118.7135	Pingnan, Ningde, Fujian (PNX)	Cfab-2	26.00	95.66%	This study
C.fabr_PNX_11-8_BDSW192000280-1a	*C. fabri*	26.9312	118.7147	Pingnan, Ningde, Fujian (PNX)	Cfab-2	30.59	96.36%	This study
C.fabr_WYX_6-10_BDSW192000275-1a	*C. fabri*	24.6224	114.2322	Wengyuan, Shaoguan, Guangdong (WYX)	Cfab-2	34.90	96.17%	This study
C.fabr_WYX_6-5_BDSW192000271-1a	*C. fabri*	24.6263	114.2387	Wengyuan, Shaoguan, Guangdong (WYX)	Cfab-2	29.16	96.54%	This study
C.fabr_WYX_6-7_BDSW192000272-1a	*C. fabri*	24.6198	114.2354	Wengyuan, Shaoguan, Guangdong (WYX)	Cfab-2	31.28	95.30%	This study
C.fabr_WYX_6-8_BDSW192000273-1a	*C. fabri*	24.6179	114.2357	Wengyuan, Shaoguan, Guangdong (WYX)	Cfab-2	28.03	92.33%	This study
C.fabr_WYX_6-9_BDSW192000274-1a	*C. fabri*	24.6231	114.2339	Wengyuan, Shaoguan, Guangdong (WYX)	Cfab-2	31.54	92.60%	This study
C.lamo_JGS_5-16_BDSW192017341-1a	*C. fabri*	26.6428	114.1707	Jinggangshan, Jiangxi (JGS)	Cfab-3	33.65	97.99%	This study
C.lamo_JGS_5-20_BDSW192017342-1a	*C. fabri*	26.7482	114.2736	Jinggangshan, Jiangxi (JGS)	Cfab-3	32.24	96.81%	This study
C.fabr_PGQ_3-1_BDSW192017312-1a	*C. fabri*	24.6195	111.5402	Pinggui, Hezhou, Guangxi (PGQ)	Cfab-3	32.47	97.00%	This study
C.fabr_PGQ_3-3_BDSW192017313-1a	*C. fabri*	24.6219	111.5391	Pinggui, Hezhou, Guangxi (PGQ)	Cfab-3	36.89	96.59%	This study
C.fabr_PGQ_3-5_BDSW192017314-1a	*C. fabri*	24.6284	111.5329	Pinggui, Hezhou, Guangxi (PGQ)	Cfab-3	33.48	96.70%	This study
C.fabr_XYX_5-1_BDSW192017305-1a	*C. fabri*	22.2906	111.2174	Xinyi, Maoming, Guangdong (XYX)	Cfab-3	34.36	95.70%	This study
C.fabr_XYX_5-2_BDSW192017306-1a	*C. fabri*	22.2908	111.2150	Xinyi, Maoming, Guangdong (XYX)	Cfab-3	30.82	95.25%	This study
C.fabr_PGQ_3-7_BDSW192017315-1a	*C. fabri*	24.6329	111.5253	Pinggui, Hezhou, Guangxi (PGQ)	Cfab-4	33.75	96.42%	This study
C.fabr_PGQ_3-9_BDSW192017316-1a	*C. fabri*	24.6361	111.5185	Pinggui, Hezhou, Guangxi (PGQ)	Cfab-4	41.47	97.69%	This study
C.farg_CHQ_2-1_BDSW192000289-1a	*C. fargesii*	23.7741	113.9316	Conghua, Guangzhou, Guangdong (CHQ)	Cfar-1	34.03	96.51%	This study
C.farg_CHQ_2-12_BDSW192000293-1a	*C. fargesii*	23.7854	113.9976	Conghua, Guangzhou, Guangdong (CHQ)	Cfar-1	28.66	96.92%	This study
C.farg_CHQ_2-3_BDSW192000290-1a	*C. fargesii*	23.7744	113.9315	Conghua, Guangzhou, Guangdong (CHQ)	Cfar-1	37.00	94.00%	This study
C.farg_CHQ_2-6_BDSW192000291-1a	*C. fargesii*	23.7841	113.9943	Conghua, Guangzhou, Guangdong (CHQ)	Cfar-1	35.01	96.11%	This study
C.farg_CHQ_2-9_BDSW192017372-1a	*C. fargesii*	23.7846	113.9958	Conghua, Guangzhou, Guangdong (CHQ)	Cfar-1	30.65	97.82%	This study
C.farg_GXS_4-5_BDSW192004230-1a	*C. fargesii*	28.0340	117.1055	Guixi, Yingtan, Jiangxi (GXS)	Cfar-1	40.95	97.16%	This study
C.farg_GXS_4-6_BDSW192004231-1a	*C. fargesii*	28.0342	117.1097	Guixi, Yingtan, Jiangxi (GXS)	Cfar-1	43.02	97.69%	This study
C.farg_GXS_4-7_BDSW192004232-1a	*C. fargesii*	28.0322	117.1112	Guixi, Yingtan, Jiangxi (GXS)	Cfar-1	43.35	97.90%	This study
C.farg_GXS_4-8_BDSW192004233-1a	*C. fargesii*	28.0286	117.1179	Guixi, Yingtan, Jiangxi (GXS)	Cfar-1	44.23	98.29%	This study
C.farg_GXS_4-9_BDSW192004234-1a	*C. fargesii*	28.0199	117.1346	Guixi, Yingtan, Jiangxi (GXS)	Cfar-1	38.69	97.27%	This study
C.farg_PGQ_1-1_BDSW192012370-1a	*C. fargesii*	24.6205	111.5398	Pinggui, Hezhou, Guangxi (PGQ)	Cfar-1	37.30	96.42%	This study
C.farg_PGQ_1-2_BDSW192012371-1a	*C. fargesii*	24.6177	111.5406	Pinggui, Hezhou, Guangxi (PGQ)	Cfar-1	37.03	96.79%	This study
C.farg_PGQ_1-3_BDSW192012372-1a	*C. fargesii*	24.6178	111.5407	Pinggui, Hezhou, Guangxi (PGQ)	Cfar-1	30.94	94.80%	This study
C.farg_PGQ_1-4_BDSW192012373-1a	*C. fargesii*	24.6178	111.5407	Pinggui, Hezhou, Guangxi (PGQ)	Cfar-1	30.99	97.70%	This study
C.farg_PGQ_1-5_BDSW192012374-1a	*C. fargesii*	24.6178	111.5406	Pinggui, Hezhou, Guangxi (PGQ)	Cfar-1	32.11	97.27%	This study
C.farg_JKX_1-1_BDSW192012360-1a	*C. fargesii*	27.7940	108.7842	Jiangkou, Tongren, Guizhou (JKX)	Cfar-2	31.07	97.33%	This study
C.farg_JKX_1-2_BDSW192012361-1a	*C. fargesii*	27.7939	108.7845	Jiangkou, Tongren, Guizhou (JKX)	Cfar-2	25.70	97.61%	This study

C.farg_JKX_1-3_BDSW192012362-1a	*C. fargesii*	27.7954	108.7855	Jiangkou, Tongren, Guizhou (JKX)	Cfar-2	29.38	97.63%	This study
C.farg_JKX_1-4_BDSW192012363-1a	*C. fargesii*	27.8452	108.7714	Jiangkou, Tongren, Guizhou (JKX)	Cfar-2	29.51	96.09%	This study
C.farg_JKX_1-6_BDSW192012364-1a	*C. fargesii*	27.7679	108.7433	Jiangkou, Tongren, Guizhou (JKX)	Cfar-2	30.60	97.48%	This study
C.farg_YLQ_3-10_BDSW192004239-1a	*C. fargesii*	28.1971	112.9525	Yuelu, Changsha, Hunan (YLQ)	Cfar-3	39.52	98.32%	This study
C.farg_YLQ_3-2_BDSW192004235-1a	*C. fargesii*	28.1971	112.9525	Yuelu, Changsha, Hunan (YLQ)	Cfar-3	34.18	97.32%	This study
C.farg_YLQ_3-4_BDSW192004236-1a	*C. fargesii*	28.1971	112.9525	Yuelu, Changsha, Hunan (YLQ)	Cfar-3	35.05	97.69%	This study
C.farg_YLQ_3-6_BDSW192004237-1a	*C. fargesii*	28.1971	112.9525	Yuelu, Changsha, Hunan (YLQ)	Cfar-3	41.14	97.36%	This study
C.farg_YLQ_3-8_BDSW192004238-1a	*C. fargesii*	28.1971	112.9525	Yuelu, Changsha, Hunan (YLQ)	Cfar-3	36.90	97.70%	This study
C.fiss_YCS_1-1_BDSW192004260-1a	*C. fissa*	21.9000	111.5000	Yangchun, Yangjiang, Guangdong (YCS)	Cfis-1	50.94	95.94%	This study
C.fiss_YCS_1-2_BDSW192004261-1a	*C. fissa*	21.9000	111.5000	Yangchun, Yangjiang, Guangdong (YCS)	Cfis-1	46.07	93.27%	This study
C.fiss_YCS_1-3_BDSW192004262-1a	*C. fissa*	21.9000	111.5000	Yangchun, Yangjiang, Guangdong (YCS)	Cfis-1	43.11	97.41%	This study
C.fiss_YCS_1-4_BDSW192004263-1a	*C. fissa*	21.9000	111.5000	Yangchun, Yangjiang, Guangdong (YCS)	Cfis-1	45.63	94.59%	This study
C.fiss_YCS_1-5_BDSW192004264-1a	*C. fissa*	21.9000	111.5000	Yangchun, Yangjiang, Guangdong (YCS)	Cfis-1	45.60	97.56%	This study
C.fiss_QLX_6-1_BDSW192004254-1a	*C. fissa*	25.9775	116.8402	Qingliu, Sanming, Fujian (QLX)	Cfis-2	35.75	97.53%	This study
C.fiss_SMZ_5-1_BDSW192017353-1a	*C. fissa*	25.6991	109.8761	Sanmen,Guangxi (SMZ)	Cfis-2	37.97	97.43%	This study
C.fiss_SMZ_5-2_BDSW192017354-1a	*C. fissa*	25.6382	109.9078	Sanmen,Guangxi (SMZ)	Cfis-2	32.26	97.11%	This study
C.fiss_WYX_7-4_BDSW192004245-1a	*C. fissa*	24.6230	114.2382	Wengyuan, Shaoguan, Guangdong (WYX)	Cfis-2	44.90	97.43%	This study
C.fiss_WYX_7-5_BDSW192004246-1a	*C. fissa*	24.6216	114.2356	Wengyuan, Shaoguan, Guangdong (WYX)	Cfis-2	40.88	97.69%	This study
C.fiss_WYX_7-6_BDSW192004247-1a	*C. fissa*	24.6232	114.2347	Wengyuan, Shaoguan, Guangdong (WYX)	Cfis-2	43.73	97.36%	This study
C.fiss_WYX_7-7_BDSW192004248-1a	*C. fissa*	24.6227	114.2326	Wengyuan, Shaoguan, Guangdong (WYX)	Cfis-2	41.42	97.77%	This study
C.fiss_WHX_4-1_BDSW192004249-1a	*C. fissa*	23.8294	115.3781	Wuhua, Meizhou, Guangdong (WHX)	Cfis-2	41.31	96.85%	This study
C.fiss_WHX_4-3_BDSW192004250-1a	*C. fissa*	23.8281	115.3778	Wuhua, Meizhou, Guangdong (WHX)	Cfis-2	37.92	95.60%	This study
C.fiss_WHX_4-5_BDSW192004251-1a	*C. fissa*	23.8264	115.3755	Wuhua, Meizhou, Guangdong (WHX)	Cfis-2	40.42	94.00%	This study
C.fiss_WHX_4-7_BDSW192004252-1a	*C. fissa*	23.8195	115.3688	Wuhua, Meizhou, Guangdong (WHX)	Cfis-2	38.64	95.97%	This study
C.fiss_WHX_4-9_BDSW192004253-1a	*C. fissa*	23.8181	115.3664	Wuhua, Meizhou, Guangdong (WHX)	Cfis-2	42.52	96.26%	This study
C.fiss_DHQ_1-1_BDSW192004255-1a	*C. fissa*	23.1724	112.5398	Dinghu, Zhaoqing, Guangdong (DHQ)	Cfis-3	41.92	96.95%	This study
C.fiss_DHQ_1-3_BDSW192004256-1a	*C. fissa*	23.1725	112.5390	Dinghu, Zhaoqing, Guangdong (DHQ)	Cfis-3	40.54	96.38%	This study
C.fiss_DHQ_1-5_BDSW192004257-1a	*C. fissa*	23.1723	112.5390	Dinghu, Zhaoqing, Guangdong (DHQ)	Cfis-3	40.82	91.51%	This study
C.fiss_DHQ_1-6_BDSW192004258-1a	*C. fissa*	23.1724	112.5392	Dinghu, Zhaoqing, Guangdong (DHQ)	Cfis-3	44.65	96.37%	This study
C.fiss_DHQ_1-7_BDSW192004259-1a	*C. fissa*	23.1726	112.5399	Dinghu, Zhaoqing, Guangdong (DHQ)	Cfis-3	39.26	96.84%	This study
C_ford_QNX_8-2_BDSW192004270-1a	*C. fordii*	24.6818	114.4610	Quannan, Ganzhou, Jiangxi (QNX)	Cfor-1	46.05	96.65%	This study
C_ford_QNX_8-3_BDSW192004271-1a	*C. fordii*	24.6819	114.4600	Quannan, Ganzhou, Jiangxi (QNX)	Cfor-1	44.28	97.01%	This study
C_ford_QNX_8-4_BDSW192004272-1a	*C. fordii*	24.6502	114.2557	Quannan, Ganzhou, Jiangxi (QNX)	Cfor-1	37.07	95.01%	This study
C_ford_QNX_8-5_BDSW192004273-1a	*C. fordii*	24.6533	114.2534	Quannan, Ganzhou, Jiangxi (QNX)	Cfor-1	39.48	95.82%	This study
C_ford_QNX_8-6_BDSW192004274-1a	*C. fordii*	24.6551	114.2507	Quannan, Ganzhou, Jiangxi (QNX)	Cfor-1	49.08	98.08%	This study
C_ford_CHQ_8-1_BDSW192004265-1a	*C. fordii*	23.7541	113.9549	Conghua, Guangzhou, Guangdong (CHQ)	Cfor-2	37.25	97.59%	This study
C_ford_CHQ_8-2_BDSW192004266-1a	*C. fordii*	23.7536	113.9538	Conghua, Guangzhou, Guangdong (CHQ)	Cfor-2	43.53	97.21%	This study
C_ford_CHQ_8-3_BDSW192004267-1a	*C. fordii*	23.7528	113.9537	Conghua, Guangzhou, Guangdong (CHQ)	Cfor-2	41.24	97.25%	This study
C_ford_CHQ_8-4_BDSW192004268-1a	*C. fordii*	23.7523	113.9536	Conghua, Guangzhou, Guangdong (CHQ)	Cfor-2	37.93	97.28%	This study
C_ford_CHQ_8-5_BDSW192004269-1a	*C. fordii*	23.7509	113.9532	Conghua, Guangzhou, Guangdong (CHQ)	Cfor-2	34.17	96.64%	This study
C_ford_PNX_7-2_BDSW192004280-1a	*C. fordii*	26.8955	119.1619	Pingnan, Ningde, Fujian (PNX)	Cfor-3	37.66	97.67%	This study
C_ford_PNX_7-3_BDSW192004281-1a	*C. fordii*	26.9515	119.1516	Pingnan, Ningde, Fujian (PNX)	Cfor-3	46.41	96.16%	This study
C_ford_PNX_7-4_BDSW192004282-1a	*C. fordii*	26.9091	119.1812	Pingnan, Ningde, Fujian (PNX)	Cfor-3	44.54	97.20%	This study
C_ford_PNX_7-5_BDSW192017369-1a	*C. fordii*	26.9082	119.1925	Pingnan, Ningde, Fujian (PNX)	Cfor-3	40.22	97.42%	This study
C_ford_PNX_7-6_BDSW192017370-1a	*C. fordii*	26.9099	119.1957	Pingnan, Ningde, Fujian (PNX)	Cfor-3	33.17	97.74%	This study
C_ford_QLX_4-1_BDSW192004277-1a	*C. fordii*	25.9618	116.8343	Qingliu, Sanming, Fujian (QLX)	Cfor-3	39.80	96.47%	This study
C_ford_QLX_4-2_BDSW192004278-1a	*C. fordii*	26.0136	116.8587	Qingliu, Sanming, Fujian (QLX)	Cfor-3	37.49	97.13%	This study
C_ford_QLX_4-3_BDSW192004279-1a	*C. fordii*	26.0148	116.8589	Qingliu, Sanming, Fujian (QLX)	Cfor-3	38.76	97.58%	This study
C_ford_WHX_5-1_BDSW192004275-1a	*C. fordii*	23.8282	115.3775	Wuhua, Meizhou, Guangdong (WHX)	Cfor-3	43.30	95.72%	This study
C_ford_WHX_5-2_BDSW192017368-1a	*C. fordii*	23.8294	115.3783	Wuhua, Meizhou, Guangdong (WHX)	Cfor-3	30.53	94.46%	This study
C.hyst_NPX_1-1_BDSW192012443-1a	*C. hystrix*	23.3180	105.8001	Napo, Baise, Guangxi (NPX)	Chys-1	33.49	97.26%	This study
C.hyst_RJX_5-3_BDSW192012439-1a	*C. hystrix*	26.1524	108.2463	Rongjiang , Guizhou (RJX)	Chys-1	30.73	97.63%	This study
C.hyst_RJX_5-5_BDSW192012440-1a	*C. hystrix*	26.1476	108.2474	Rongjiang , Guizhou (RJX)	Chys-1	28.51	97.70%	This study
C.hyst_RJX_5-6_BDSW192012441-1a	*C. hystrix*	26.1466	108.2485	Rongjiang , Guizhou (RJX)	Chys-1	32.50	95.74%	This study
C.hyst_RJX_5-7_BDSW192020798-1a	*C. hystrix*	26.1395	108.2502	Rongjiang , Guizhou (RJX)	Chys-1	29.98	97.41%	This study
C.hyst_CHQ_3-1_BDSW192004285-1a	*C. hystrix*	23.8267	114.0114	Conghua, Guangzhou, Guangdong (CHQ)	Chys-2	41.64	95.67%	This study
C.hyst_CHQ_3-2_BDSW192004286-1a	*C. hystrix*	23.8450	114.0121	Conghua, Guangzhou, Guangdong (CHQ)	Chys-2	38.09	94.51%	This study
C.hyst_CHQ_3-3_BDSW192004287-1a	*C. hystrix*	23.8771	114.0066	Conghua, Guangzhou, Guangdong (CHQ)	Chys-2	40.33	97.59%	This study
C.hyst_CHQ_3-4_BDSW192004288-1a	*C. hystrix*	23.8856	114.0076	Conghua, Guangzhou, Guangdong (CHQ)	Chys-2	28.51	98.14%	This study
C.hyst_NPX_1-2_BDSW192012444-1a	*C. hystrix*	23.3085	105.8006	Napo, Baise, Guangxi (NPX)	Chys-2	28.78	97.87%	This study
C.hyst_NPX_1-3_BDSW192017302-1a	*C. hystrix*	23.3082	105.8008	Napo, Baise, Guangxi (NPX)	Chys-2	30.84	95.70%	This study
C.hyst_NPX_1-4_BDSW192017303-1a	*C. hystrix*	23.3077	105.8019	Napo, Baise, Guangxi (NPX)	Chys-2	32.47	95.47%	This study
C.hyst_NPX_1-5_BDSW192017304-1a	*C. hystrix*	23.3068	105.8029	Napo, Baise, Guangxi (NPX)	Chys-2	27.53	94.75%	This study
Ca.carl_WYX_10-1_BDSW192017373-1a	*C. hystrix*	24.6263	114.2387	Wengyuan, Shaoguan, Guangdong (WYX)	Chys-2	32.04	96.98%	This study
C.hyst_WHX_2-1_BDSW192004289-1a	*C. hystrix*	23.8858	115.4903	Wuhua , Meizhou, Guangdong (WHX)	Chys-2	39.83	97.52%	This study
C.hyst_WHX_2-3_BDSW192004290-1a	*C. hystrix*	23.8809	115.4695	Wuhua , Meizhou, Guangdong (WHX)	Chys-2	38.45	96.25%	This study
C.hyst_WHX_2-5_BDSW192004291-1a	*C. hystrix*	23.8817	115.4636	Wuhua , Meizhou, Guangdong (WHX)	Chys-2	41.76	96.78%	This study
C.hyst_WHX_2-7_BDSW192004293-1a	*C. hystrix*	23.8819	115.4694	Wuhua , Meizhou, Guangdong (WHX)	Chys-2	29.01	96.71%	This study
C.hyst_XYX_1-3_BDSW192012434-1a	*C. hystrix*	22.3677	111.0355	Xinyi , Maoming, Guangdong (XYX)	Chys-2	36.47	97.39%	This study
C.jucu_QNX_2-1_BDSW192004470-1a	*C. jucunda*	24.6986	114.4975	Quannan, Ganzhou, Jiangxi (QNX)	Cjus-1	23.77	96.44%	This study
C.jucu_QNX_2-2_BDSW192004471-1a	*C. jucunda*	24.6987	114.4972	Quannan, Ganzhou, Jiangxi (QNX)	Cjus-1	27.28	97.23%	This study
C.jucu_TSX_2-1_BDSW192004480-1a	*C. jucunda*	29.4643	114.4089	Tongshan, Xianning , Hubei (TSX)	Cjus-1	20.60	96.96%	This study
C.jucu_TSX_2-11_BDSW192004484-1a	*C. jucunda*	29.4498	114.4187	Tongshan, Xianning , Hubei (TSX)	Cjus-1	21.56	97.86%	This study
C.jucu_TSX_2-5_BDSW192004481-1a	*C. jucunda*	29.4514	114.4154	Tongshan, Xianning , Hubei (TSX)	Cjus-1	23.29	97.26%	This study
C.jucu_TSX_2-7_BDSW192004482-1a	*C. jucunda*	29.4520	114.4157	Tongshan, Xianning , Hubei (TSX)	Cjus-1	23.85	98.20%	This study
C.jucu_TSX_2-9_BDSW192004483-1a	*C. jucunda*	29.4512	114.4183	Tongshan, Xianning , Hubei (TSX)	Cjus-1	22.89	96.79%	This study
C.jucu_HSX_7-1_BDSW192004485-1a	*C. jucunda*	26.8674	112.1350	Hengnan, Hengyang, Hunan (HSX)	Cjus-2	19.60	97.97%	This study
C.jucu_HSX_7-11_BDSW192004489-1a	*C. jucunda*	26.8695	112.1333	Hengnan, Hengyang, Hunan (HSX)	Cjus-2	22.36	97.14%	This study
C.jucu_HSX_7-3_BDSW192004486-1a	*C. jucunda*	26.8678	112.1355	Hengnan, Hengyang, Hunan (HSX)	Cjus-2	27.54	97.71%	This study
C.jucu_HSX_7-7_BDSW192004487-1a	*C. jucunda*	26.8690	112.1362	Hengnan, Hengyang, Hunan (HSX)	Cjus-2	24.06	97.84%	This study
C.jucu_HSX_7-9_BDSW192004488-1a	*C. jucunda*	26.8691	112.1360	Hengnan, Hengyang, Hunan (HSX)	Cjus-2	21.14	97.49%	This study
C.jucu_GXS_3-1_BDSW192004472-1a	*C. jucunda*	28.0915	116.9572	Guixi, Yingtan, Jiangxi (GXS)	Cjus-3	33.69	98.20%	This study
C.jucu_GXS_3-2_BDSW192004473-1a	*C. jucunda*	28.0315	117.0877	Guixi, Yingtan, Jiangxi (GXS)	Cjus-3	25.34	96.58%	This study
C.jucu_GXS_3-3_BDSW192004474-1a	*C. jucunda*	28.0294	117.0912	Guixi, Yingtan, Jiangxi (GXS)	Cjus-3	21.20	95.86%	This study
C.jucu_GXS_3-4_BDSW192004475-1a	*C. jucunda*	28.0310	117.0982	Guixi, Yingtan, Jiangxi (GXS)	Cjus-3	23.25	97.78%	This study
C.jucu_GXS_3-5_BDSW192004476-1a	*C. jucunda*	27.9522	117.1486	Guixi, Yingtan, Jiangxi (GXS)	Cjus-3	22.54	95.79%	This study
C.jucu_QMX_5-1_BDSW192004477-1a	*C. jucunda*	30.0141	117.5274	Qimen, Huangshan, Anhui (QMX)	Cjus-3	18.78	97.74%	This study
C.jucu_QMX_5-2_BDSW192004478-1a	*C. jucunda*	30.0136	117.5275	Qimen, Huangshan, Anhui (QMX)	Cjus-3	22.28	97.49%	This study
C.jucu_QMX_5-3_BDSW192004479-1a	*C. jucunda*	29.9579	117.5085	Qimen, Huangshan, Anhui (QMX)	Cjus-3	22.32	96.74%	This study
C.jucu_SCX_4-1_BDSW192009053-1a	*C. jucunda*	28.5461	119.2176	Suichang, Lishui, Zhejiang (SCX)	Cjus-4	40.17	97.13%	This study
C.jucu_SCX_4-2_BDSW192009054-1a	*C. jucunda*	28.5473	119.2194	Suichang, Lishui, Zhejiang (SCX)	Cjus-4	33.68	96.50%	This study
C.jucu_SCX_4-3_BDSW192009055-1a	*C. jucunda*	28.5485	119.2210	Suichang, Lishui, Zhejiang (SCX)	Cjus-4	25.37	97.64%	This study
C.jucu_SCX_4-4_BDSW192009056-1a	*C. jucunda*	28.5648	119.2265	Suichang, Lishui, Zhejiang (SCX)	Cjus-4	32.61	97.78%	This study
C.lamo_NPX_5-1_BDSW192017343-1a	*C. lamontii*	23.3156	105.8001	Napo, Baise, Guangxi (NPX)	Clam-1	34.23	96.52%	This study
C.lamo_NPX_5-3_BDSW192017344-1a	*C. lamontii*	23.2974	105.8028	Napo, Baise, Guangxi (NPX)	Clam-1	36.75	96.68%	This study
C.lamo_NPX_5-5_BDSW192017345-1a	*C. lamontii*	23.2975	105.8029	Napo, Baise, Guangxi (NPX)	Clam-1	29.12	96.99%	This study
C.lamo_NPX_5-6_BDSW192017346-1a	*C. lamontii*	23.2935	105.8007	Napo, Baise, Guangxi (NPX)	Clam-1	33.35	94.47%	This study
C.lamo_NPX_5-7_BDSW192017347-1a	*C. lamontii*	23.2950	105.8022	Napo, Baise, Guangxi (NPX)	Clam-1	32.97	97.18%	This study
C.lamo_CHQ_7-1_BDSW192004490-1a	*C. lamontii*	23.7541	113.9549	Conghua, Guangzhou, Guangdong (CHQ)	Clam-2	18.79	96.91%	This study
C.lamo_CHQ_7-2_BDSW192004491-1a	*C. lamontii*	23.7540	113.9546	Conghua, Guangzhou, Guangdong (CHQ)	Clam-2	22.61	95.49%	This study
C.lamo_CHQ_7-3_BDSW192004492-1a	*C. lamontii*	23.7493	113.9533	Conghua, Guangzhou, Guangdong (CHQ)	Clam-2	24.57	95.74%	This study
C.lamo_CHQ_7-4_BDSW192004493-1a	*C. lamontii*	23.7489	113.9535	Conghua, Guangzhou, Guangdong (CHQ)	Clam-2	19.70	97.33%	This study
C.lamo_CHQ_7-6_BDSW192004494-1a	*C. lamontii*	23.8462	114.0092	Conghua, Guangzhou, Guangdong (CHQ)	Clam-2	22.46	91.85%	This study
C.lamo_QLX_5-1_BDSW192004499-1a	*C. lamontii*	25.9775	116.8401	Qingliu, Sanming, Fujian (QLX)	Clam-2	21.18	97.10%	This study
C.lamo_QLX_5-2_BDSW192004500-1a	*C. lamontii*	25.9800	116.8405	Qingliu, Sanming, Fujian (QLX)	Clam-2	21.02	97.01%	This study
C.lamo_QNX_4-1_BDSW192004495-1a	*C. lamontii*	24.6985	114.4974	Quannan, Ganzhou, Jiangxi (QNX)	Clam-2	24.20	94.38%	This study
C.lamo_QNX_4-2_BDSW192004496-1a	*C. lamontii*	24.6989	114.4968	Quannan, Ganzhou, Jiangxi (QNX)	Clam-2	19.51	90.26%	This study

C.lamo_QNX_4-3_BDSW192004497-1a	*C. lamontii*	24.6502	114.2555	Quannan, Ganzhou, Jiangxi (QNX)	Clam-2	23.39	97.15%	This study
C.lamo_QNX_4-4_BDSW192004498-1a	*C. lamontii*	24.6502	114.2556	Quannan, Ganzhou, Jiangxi (QNX)	Clam-2	17.70	96.78%	This study
C.lamo_JGS_5-1_BDSW192017338-1a	*C. lamontii*	26.5501	114.1748	Jinggangshan, Jiangxi (JGS)	Clam-3	28.03	97.47%	This study
C.lamo_JGS_5-11_BDSW192017340-1a	*C. lamontii*	26.5374	114.1933	Jinggangshan, Jiangxi (JGS)	Clam-3	26.56	95.45%	This study
C.lamo_JGS_5-6_BDSW192017339-1a	*C. lamontii*	26.5496	114.1754	Jinggangshan, Jiangxi (JGS)	Clam-3	31.40	97.90%	This study
C.lamo_PGQ_4-5_BDSW192017348-1a	*C. lamontii*	24.6211	111.5396	Pinggui , Hezhou, Guangxi (PGQ)	Clam-4	29.03	94.70%	This study
C.lamo_PGQ_4-6_BDSW192017349-1a	*C. lamontii*	24.6215	111.5393	Pinggui , Hezhou, Guangxi (PGQ)	Clam-4	32.34	96.29%	This study
C.lamo_PGQ_4-7_BDSW192017350-1a	*C. lamontii*	24.6219	111.5391	Pinggui , Hezhou, Guangxi (PGQ)	Clam-4	32.20	95.50%	This study
C.lamo_PGQ_4-8_BDSW192017351-1a	*C. lamontii*	24.6233	111.5380	Pinggui , Hezhou, Guangxi (PGQ)	Clam-4	28.06	96.23%	This study
C.lamo_PGQ_4-9_BDSW192017352-1a	*C. lamontii*	24.6252	111.5365	Pinggui , Hezhou, Guangxi (PGQ)	Clam-4	33.56	97.49%	This study
C.scle_YLQ_1-12_BDSW192009022-1a	*C. sclerophylla*	28.1795	112.9286	Yuelu, Changsha, Hunan (YLQ)	Cscl-1	30.37	97.44%	This study
C.scle_YLQ_1-4_BDSW192009019-1a	*C. sclerophylla*	28.1971	112.9525	Yuelu, Changsha, Hunan (YLQ)	Cscl-1	23.49	94.59%	This study
C.scle_YLQ_1-7_BDSW192009020-1a	*C. sclerophylla*	28.1971	112.9525	Yuelu, Changsha, Hunan (YLQ)	Cscl-1	33.91	96.39%	This study
C.scle_NL_3-1_BDSW192017317-1a	*C. sclerophylla*	24.9513	113.1489	Nanling, Shaoguan, Guangdong (NL)	Cscl-2	34.75	96.68%	This study
C.scle_NL_3-3_BDSW192017318-1a	*C. sclerophylla*	24.9508	113.1491	Nanling, Shaoguan, Guangdong (NL)	Cscl-2	33.66	97.75%	This study
C.scle_NL_3-5_BDSW192017319-1a	*C. sclerophylla*	24.9487	113.1462	Nanling, Shaoguan, Guangdong (NL)	Cscl-2	32.43	96.00%	This study
C.scle_NL_3-7_BDSW192017320-1a	*C. sclerophylla*	24.9504	113.1497	Nanling, Shaoguan, Guangdong (NL)	Cscl-2	33.06	97.74%	This study
C.scle_NL_3-9_BDSW192017321-1a	*C. sclerophylla*	24.9503	113.1503	Nanling, Shaoguan, Guangdong (NL)	Cscl-2	28.87	96.56%	This study
C.scle_FYQ_2-10_BDSW192009010-1a	*C. sclerophylla*	30.1529	119.9382	Fuyang, Hangzhou, Zhejiang (FYQ)	Cscl-3	41.77	96.53%	This study
C.scle_FYQ_2-12_BDSW192009011-1a	*C. sclerophylla*	30.1543	119.9381	Fuyang, Hangzhou, Zhejiang (FYQ)	Cscl-3	30.33	95.73%	This study
C.scle_FYQ_2-4_BDSW192009007-1a	*C. sclerophylla*	30.1455	119.9396	Fuyang, Hangzhou, Zhejiang (FYQ)	Cscl-3	30.68	95.81%	This study
C.scle_FYQ_2-6_BDSW192009008-1a	*C. sclerophylla*	30.1507	119.9377	Fuyang, Hangzhou, Zhejiang (FYQ)	Cscl-3	29.48	95.71%	This study
C.scle_FYQ_2-8_BDSW192009009-1a	*C. sclerophylla*	30.1520	119.9375	Fuyang, Hangzhou, Zhejiang (FYQ)	Cscl-3	29.02	96.56%	This study
C.scle_STX_2-1_BDSW192009012-1a	*C. sclerophylla*	30.1511	117.5158	Shitai, Chizhou, Anhui (STX)	Cscl-3	34.57	97.10%	This study
C.scle_STX_2-2_BDSW192009013-1a	*C. sclerophylla*	30.1574	117.4975	Shitai, Chizhou, Anhui (STX)	Cscl-3	32.28	96.35%	This study
C.scle_STX_2-3_BDSW192009014-1a	*C. sclerophylla*	30.1560	117.4953	Shitai, Chizhou, Anhui (STX)	Cscl-3	37.15	96.66%	This study
C.scle_STX_2-4_BDSW192009015-1a	*C. sclerophylla*	30.1334	117.4969	Shitai, Chizhou, Anhui (STX)	Cscl-3	27.00	95.48%	This study
C.scle_STX_2-5_BDSW192009016-1a	*C. sclerophylla*	30.1294	117.4960	Shitai, Chizhou, Anhui (STX)	Cscl-3	32.12	94.79%	This study
C.scle_ZXX_2-4_BDSW192008997-1a	*C. sclerophylla*	27.8069	117.1046	Zixi, Fuzhou, Jiangxi (ZXX)	Cscl-3	30.75	94.40%	This study
C.scle_ZXX_2-5_BDSW192008998-1a	*C. sclerophylla*	27.8077	117.1048	Zixi, Fuzhou, Jiangxi (ZXX)	Cscl-3	28.11	96.84%	This study
C.scle_ZXX_2-6_BDSW192008999-1a	*C. sclerophylla*	27.8089	117.1075	Zixi, Fuzhou, Jiangxi (ZXX)	Cscl-3	35.84	94.52%	This study
C.scle_ZXX_2-7_BDSW192009000-1a	*C. sclerophylla*	27.8107	117.1127	Zixi, Fuzhou, Jiangxi (ZXX)	Cscl-3	29.32	94.83%	This study
C.scle_ZXX_2-8_BDSW192009001-1a	*C. sclerophylla*	27.8181	117.1138	Zixi, Fuzhou, Jiangxi (ZXX)	Cscl-3	31.25	97.92%	This study
C.tibe_JGS_3-1_BDSW192017327-1a	*C. tibetana*	26.5644	114.1234	Jinggangshan, Jiangxi (JGS)	Ctib-1	30.26	96.78%	This study
C.tibe_JGS_3-4_BDSW192017328-1a	*C. tibetana*	26.5500	114.1750	Jinggangshan, Jiangxi (JGS)	Ctib-1	24.82	94.56%	This study
C.tibe_JGS_3-7_BDSW192017329-1a	*C. tibetana*	26.5508	114.1732	Jinggangshan, Jiangxi (JGS)	Ctib-1	29.26	96.02%	This study
C.tibe_JGS_3-10_BDSW192017330-1a	*C. tibetana*	26.5503	114.1746	Jinggangshan, Jiangxi (JGS)	Ctib-2	32.30	96.33%	This study
C.tibe_JGS_3-13_BDSW192017331-1a	*C. tibetana*	26.6511	114.1987	Jinggangshan, Jiangxi (JGS)	Ctib-2	28.79	97.01%	This study
C.tibe_KHX_5-1_BDSW192009044-1a	*C. tibetana*	29.2451	118.1154	Kaihua, Quzhou, Zhejiang (KHX)	Ctib-2	35.99	95.56%	This study
C.tibe_KHX_5-10_BDSW192009048-1a	*C. tibetana*	29.2493	118.1369	Kaihua, Quzhou, Zhejiang (KHX)	Ctib-2	32.44	93.89%	This study
C.tibe_KHX_5-3_BDSW192009045-1a	*C. tibetana*	29.2453	118.1208	Kaihua, Quzhou, Zhejiang (KHX)	Ctib-2	28.87	97.05%	This study
C.tibe_KHX_5-5_BDSW192009046-1a	*C. tibetana*	29.2481	118.1325	Kaihua, Quzhou, Zhejiang (KHX)	Ctib-2	37.28	96.55%	This study
C.tibe_KHX_5-8_BDSW192009047-1a	*C. tibetana*	29.2491	118.1341	Kaihua, Quzhou, Zhejiang (KHX)	Ctib-2	29.92	96.98%	This study
C.tibe_PNX_6-4_BDSW192017337-1a	*C. tibetana*	26.9090	119.1882	Pingnan, Ningde, Fujian (PNX)	Ctib-2	28.60	96.34%	This study
C.tibe_PNX_6-5_BDSW192009039-1a	*C. tibetana*	26.9100	119.1957	Pingnan, Ningde, Fujian (PNX)	Ctib-2	36.09	96.91%	This study
C.tibe_PNX_6-6_BDSW192009040-1a	*C. tibetana*	26.9153	119.1989	Pingnan, Ningde, Fujian (PNX)	Ctib-2	31.98	97.43%	This study
C.tibe_PNX_6-7_BDSW192009041-1a	*C. tibetana*	26.9150	119.1992	Pingnan, Ningde, Fujian (PNX)	Ctib-2	31.31	97.36%	This study
C.tibe_PNX_6-8_BDSW192009042-1a	*C. tibetana*	26.9149	119.1988	Pingnan, Ningde, Fujian (PNX)	Ctib-2	34.82	97.70%	This study
C.tibe_QNX_9-1_BDSW192009033-1a	*C. tibetana*	24.6818	114.4599	Quannan, Ganzhou, Jiangxi (QNX)	Ctib-2	42.51	96.61%	This study
C.tibe_QNX_9-2_BDSW192009034-1a	*C. tibetana*	24.6818	114.4598	Quannan, Ganzhou, Jiangxi (QNX)	Ctib-2	37.75	97.12%	This study
C.tibe_QNX_9-3_BDSW192009035-1a	*C. tibetana*	24.6816	114.4602	Quannan, Ganzhou, Jiangxi (QNX)	Ctib-2	34.25	97.33%	This study
C.tibe_QNX_9-4_BDSW192009036-1a	*C. tibetana*	24.6818	114.4610	Quannan, Ganzhou, Jiangxi (QNX)	Ctib-2	36.69	96.71%	This study
C.tibe_WHX_6-1_BDSW192009037-1a	*C. tibetana*	23.8178	115.3668	Wuhua, Meizhou, Guangdong (WHX)	Ctib-2	33.94	96.47%	This study
C.tibe_WHX_6-2_BDSW192009038-1a	*C. tibetana*	23.8180	115.3670	Wuhua, Meizhou, Guangdong (WHX)	Ctib-2	31.19	95.99%	This study
C.tibe_SMZ_2-1_BDSW192017322-1a	*C. tibetana*	25.6478	109.8965	Sanmen, Guilin, Guangxi (SMZ)	Ctib-3	35.32	97.81%	This study
C.tibe_SMZ_2-3_BDSW192017323-1a	*C. tibetana*	25.6476	109.8967	Sanmen, Guilin, Guangxi (SMZ)	Ctib-3	32.29	96.51%	This study
C.tibe_SMZ_2-5_BDSW192017324-1a	*C. tibetana*	25.6489	109.9018	Sanmen, Guilin, Guangxi (SMZ)	Ctib-3	39.07	97.17%	This study
C.tibe_SMZ_2-7_BDSW192017325-1a	*C. tibetana*	25.6381	109.9077	Sanmen, Guilin, Guangxi (SMZ)	Ctib-3	28.95	96.38%	This study
C.tibe_SMZ_2-8_BDSW192017326-1a	*C. tibetana*	25.6379	109.9073	Sanmen, Guilin, Guangxi (SMZ)	Ctib-3	32.13	97.43%	This study
s0326-1-6_NDSW54417	*Q. variabilis*	29.5203	103.3394	Emeishan, SiChuan province	nan	30.28	89.07%	Liang et al. 2021
s0702-1-7_NDSW54401	*Q. variabilis*	24.0196	101.9746	XinPing, YuXi, YunNan province	nan	26.19	89.48%	Liang et al. 2021
s0712-4-3_NDSW49829	*Q. variabilis*	28.1866	112.9272	Changsha, HuNan province	nan	28.42	89.43%	Liang et al. 2021
s0714-2-10_NDSW49839	*Q. variabilis*	30.4208	111.2142	YiChang, HuBei province	nan	26.38	89.31%	Liang et al. 2021
s0716-1-4_NDSW45819	*Q. variabilis*	32.1156	114.0171	XinYang, HeNan province	nan	25.26	89.34%	Liang et al. 2021
s0717-1-9_NDSW49917	*Q. variabilis*	32.1358	119.0877	ZhenJiang, JiangSu province	nan	31.01	89.33%	Liang et al. 2021
s0719-1-9_NDSW49841	*Q. variabilis*	34.6474	119.2922	LianYunGang, JiangSu province	nan	26.81	89.53%	Liang et al. 2021
s0723-1-7_NDSW49849	*Q. variabilis*	29.4981	115.8809	JiuJiang, JiangXi province	nan	25.75	89.26%	Liang et al. 2021
s0804-1-3_NDSW49946	*Q. variabilis*	26.8695	112.1335	HengYang, HuNan province	nan	25.97	89.22%	Liang et al. 2021
s0809-1-6_NDSW49854	*Q. variabilis*	25.4750	105.0839	XingRen, GuiZhou province	nan	24.03	88.37%	Liang et al. 2021
s0830-1-13_NDSW49969	*Q. variabilis*	33.3517	105.5740	LongNan, GanSu province	nan	25.19	88.38%	Liang et al. 2021
s0904-1-7_NDSW54436	*Q. variabilis*	34.0583	108.3248	XiAn, ShaanXi province	nan	27.10	88.38%	Liang et al. 2021
s0907-1-11_NDSW54385	*Q. variabilis*	35.5373	110.1072	YanAn, ShaanXi province	nan	26.31	89.49%	Liang et al. 2021
s0911-1-7_NDSW54402	*Q. variabilis*	38.6794	113.8481	ShiJiaZhuang, HeBei province	nan	28.95	89.57%	Liang et al. 2021
s0916-1-3_NDSW54395	*Q. variabilis*	36.2698	117.0347	TaiAn, ShanDong province	nan	31.58	89.48%	Liang et al. 2021
s1006-1-6_NDSW54460	*Q. variabilis*	29.0196	122.0747	Zhoushan, ZheJIang province	nan	28.93	89.22%	Liang et al. 2021
s1009-2-3_NDSW54462	*Q. variabilis*	28.0155	118.6817	NanPing, FuJian province	nan	25.42	87.59%	Liang et al. 2021
s1013-1-12_NDSW54450	*Q. variabilis*	23.2249	108.2552	NanNing, GuangXi province	nan	29.93	89.70%	Liang et al. 2021
Z0913-1-1_NDSW54403	*Q. variabilis*	39.6731	115.8101	Beijing	nan	26.77	89.30%	Liang et al. 2021
Z0916-2-3_NDSW54409	*Q. variabilis*	38.8648	121.6612	DaLian, LiaoNing province	nan	28.54	89.15%	Liang et al. 2021
0809-4-3	*Q. dentata*	25.4600	105.0900	XingRen, GuiZhou province	nan	31.01	81.93%	Zhou et al. 2022
0902-2-10	*Q. dentata*	34.2700	107.2500	BaoJi, Shaanxi province	nan	31.54	78.62%	Zhou et al. 2022
0902-2-12	*Q. dentata*	34.2700	107.2500	BaoJi, Shaanxi province	nan	31.09	81.81%	Zhou et al. 2022
0915-4-2	*Q. dentata*	37.3000	121.7000	YanTai, ShanDong province	nan	31.28	87.87%	Zhou et al. 2022
0915-4-5	*Q. dentata*	37.3000	121.7000	YanTai, ShanDong province	nan	30.70	88.93%	Zhou et al. 2022
s0902-2-1_NDSW64940	*Q. dentata*	34.2700	107.2500	BaoJi, Shaanxi province	nan	31.29	87.86%	Zhou et al. 2022
s0902-2-3_NDSW64941	*Q. dentata*	34.2700	107.2500	BaoJi, Shaanxi province	nan	33.37	88.28%	Zhou et al. 2022
s0911-4-1_NDSW64934	*Q. dentata*	38.6800	113.8600	ShiJiaZhuang, HeBei province	nan	35.54	87.57%	Zhou et al. 2022
s0915-4-1_NDSW64999	*Q. dentata*	37.3000	121.7000	YanTai, ShanDong province	nan	32.61	89.48%	Zhou et al. 2022
s0915-4-3_NDSW65000	*Q. dentata*	37.3000	121.7000	YanTai, ShanDong province	nan	31.74	89.28%	Zhou et al. 2022
s0916-5-3_NDSW64939	*Q. dentata*	38.8600	121.6600	DaLian, LiaoNing province	nan	30.29	87.82%	Zhou et al. 2022
YS20190725-2-2_BDSW190633862-1a	*Q. dentata*	29.6361	102.1343	LuDing, SiChuang province	nan	25.53	87.57%	Zhou et al. 2022
Z0914-4-1_NDSW64936	*Q. dentata*	40.2200	119.5600	QinHuangDao, HeBei province	nan	35.74	89.26%	Zhou et al. 2022
Z0914-4-10	*Q. dentata*	40.2200	119.5600	QinHuangDao, HeBei province	nan	27.49	88.76%	Zhou et al. 2022
Z0914-4-2	*Q. dentata*	40.2200	119.5600	QinHuangDao, HeBei province	nan	30.57	88.94%	Zhou et al. 2022
Z0914-4-4	*Q. dentata*	40.2200	119.5600	QinHuangDao, HeBei province	nan	29.62	88.43%	Zhou et al. 2022
Z0916-5-10	*Q. dentata*	38.8600	121.6600	DaLian, LiaoNing province	nan	28.02	89.36%	Zhou et al. 2022
Z0916-5-2	*Q. dentata*	38.8600	121.6600	DaLian, LiaoNing province	nan	28.83	87.94%	Zhou et al. 2022
Z0916-5-4	*Q. dentata*	38.8600	121.6600	DaLian, LiaoNing province	nan	30.16	88.58%	Zhou et al. 2022
Z0916-5-5	*Q. dentata*	38.8600	121.6600	DaLian, LiaoNing province	nan	31.58	88.24%	Zhou et al. 2022

nan, not estimated.

Table S2 Summary of the *C. eyrei* genome assembly, genome annotation, and functional annotation. BUSCOs, Benchmarking the Universal Single-Copy Orthologs score of the *C. eyrei* genome assembly, with complete single-copy and duplicated BUSCOs indicated in parentheses. The rows "Percentage of repeat sequences" and "Percentage of LTR" represent the proportion of the total length of all repeat sequences and LTRs in the *C. eyrei* genome, respectively. The functional annotation section provides the numbers (percentage) genes with known functions detected in six databases: Swiss-Prot, NCBI non-redundant (NR) protein database), of predicted KEGG, InterPro, GO, and Pfam database. The "Total" row indicates the number (percentage) of genes annotated in at least one of the six databases.

Parameter	Value
**Genome assembly**	
Total length of contigs (bp)	891,034,772
Number of contigs (> 1,000 bp)	765
Number of scaffolds (> 10,000 bp)	141
No. of pseudo-chromosome	12
Total length of pseudo-chromosome (bp)	873,942,207
Percentage of pseudo-chromosome	98.08%
Contig N50 (bp)	1,701,098
Contig N90 (bp)	708,088
Scaffold N50 (bp)	69,725,657
Scaffold N90 (bp)	54,126,457
Maximum contig length (bp)	6,279,500
Maximum scaffold length (bp)	103,642,724
BUSCOs	94.90% (90.10%/4.80%)*
GC content	35.35%
**Genome annotation**	
Percentage of repeat sequences	49.57%
Percentage of LTR	41.76%
Number of protein-coding genes	45,904
Average gene length (bp)	4,825
Average CDS length (bp)	981
Average intron length (bp)	1,273
**Functonal annotation**	
Swissprot	29,647 (64%)
NCBI non-redundant (NR) protein database	44,449 (96%)
Kyoto Encyclopedia of Genes and Genomes (KEGG)	33,821 (73%)
InterPro database	37,786 (81.6%)
Gene ontology (GO)	21,179 (45.7%)
Protein family (Pfam) database	30,087 (64.9%)
Total	44,771 (96.6%)

*The complete single-copy and duplicated BUSCOs were give in parenthess.

Table S3 Summary statistics (mean ± standard error) of F_{ST} and d_{XY} estimated in non-overlapping windows (10, 100, and 500 kb) for the 66 species-pairs. The column "Species pairs" indicates the two species in each pairwise comparison, with each row representing a comparison.

	**10 kb**		**100 kb**		**500 kb**	
**Species pairs**	F_{ST}	d_{XY}	F_{ST}	d_{XY}	F_{ST}	d_{XY}
C. carlesii_C. chinensis	0.5833±0.0007	0.0177±0.0000	0.5977±0.0017	0.0171±0.0000	0.5923±0.0034	0.0170±0.0000
C. carlesii C. eyrei	0.3024±0.0006	0.0117±0.0000	0.3144±0.0017	0.0111±0.0000	0.3073±0.0032	0.0111±0.0000
C.carlesii_C. fabri	0.3930±0.0007	0.0132±0.0000	0.4076±0.0017	0.0126±0.0000	0.4010±0.0033	0.0125±0.0000
C. carlesii_C. fargesii	0.1531±0.0004	0.0095±0.0000	0.1584±0.0010	0.0089±0.0000	0.1552±0.0018	0.0089±0.0001
C. carlesii C. fissa	0.7000±0.0006	0.0213±0.0000	0.7110±0.0014	0.0206±0.0000	0.7068±0.0027	0.0204±0.0001
C. carlesii_C. fordii	0.5506±0.0007	0.0178±0.0000	0.5650±0.0018	0.0171±0.0000	0.5575±0.0036	0.0170±0.0001
C. carlesii_C. hystrix	0.5105±0.0007	0.0178±0.0000	0.5254±0.0018	0.0171±0.0000	0.5204±0.0034	0.0170±0.0001
C. carlesii C. jucunda	0.6337±0.0007	0.0170±0.0000	0.6478±0.0017	0.0165±0.0000	0.6424±0.0033	0.0164±0.0001
C. carlesii_C. lamontii	0.3642±0.0006	0.0119±0.0000	0.3785±0.0017	0.0113±0.0000	0.3717±0.0033	0.0113±0.0000
C. carlesii C. sclerophylla	0.5816±0.0007	0.0182±0.0000	0.5952±0.0018	0.0176±0.0000	0.5898±0.0034	0.0174±0.0001
C. carlesii_C. tibetana	0.5636±0.0007	0.0171±0.0000	0.5767±0.0019	0.0165±0.0000	0.5712±0.0038	0.0164±0.0001
C. chinensis_C. eyrei	0.5420±0.0007	0.0170±0.0000	0.5562±0.0017	0.0164±0.0000	0.5516±0.0033	0.0164±0.0000
C.chinensis C. fabri	0.5727±0.0007	0.0179±0.0000	0.5866±0.0017	0.0173±0.0000	0.5807±0.0034	0.0172±0.0001
C. chinensis_C. fargesii	0.5828±0.0007	0.0183±0.0000	0.5960±0.0018	0.0177±0.0000	0.5901±0.0035	0.0176±0.0001
C. chinensis_C. fissa	0.7463±0.0006	0.0221±0.0000	0.7552±0.0014	0.0214±0.0000	0.7497±0.0028	0.0213±0.0001
C. chinensis C. fordii	0.6032±0.0007	0.0188±0.0000	0.6151±0.0019	0.0182±0.0000	0.6080±0.0037	0.0181±0.0001
C. chinensis_C. hystrix	0.5580±0.0007	0.0189±0.0000	0.5709±0.0018	0.0183±0.0000	0.5656±0.0035	0.0182±0.0001
C. chinensis C. jucunda	0.6055±0.0007	0.0136±0.0000	0.6152±0.0018	0.0130±0.0000	0.6091±0.0034	0.0130±0.0000
C. chinensis_C. lamontii	0.5827±0.0007	0.0171±0.0000	0.5976±0.0017	0.0165±0.0000	0.5919±0.0034	0.0164±0.0000
C. chinensis_C. sclerophylla	0.4850±0.0007	0.0136±0.0000	0.4951±0.0019	0.0130±0.0000	0.4892±0.0036	0.0129±0.0000
C. chinensis C. tibetana	0.6202±0.0007	0.0182±0.0000	0.6307±0.0020	0.0176±0.0000	0.6243±0.0039	0.0175±0.0001
C.eyrei_C. fabri	0.3329±0.0006	0.0126±0.0000	0.3454±0.0015	0.0120±0.0000	0.3402±0.0029	0.0119±0.0000
C. eyrei_C. fargesii	0.3224±0.0006	0.0124±0.0000	0.3334±0.0017	0.0118±0.0000	0.3274±0.0034	0.0117±0.0000
C. eyrei C. fissa	0.6693±0.0006	0.0206±0.0000	0.6802±0.0014	0.0199±0.0000	0.6759±0.0027	0.0198±0.0001
C. eyrei_C. fordii	0.5114±0.0007	0.0172±0.0000	0.5252±0.0018	0.0165±0.0000	0.5189±0.0035	0.0164±0.0001
C. eyrei C. hystrix	0.4718±0.0007	0.0171±0.0000	0.4868±0.0017	0.0165±0.0000	0.4824±0.0034	0.0164±0.0001
C. eyrei_C. jucunda	0.5934±0.0006	0.0163±0.0000	0.6071±0.0017	0.0158±0.0000	0.6025±0.0033	0.0157±0.0000
C. eyrei_C. lamontii	0.2777±0.0006	0.0110±0.0000	0.2898±0.0014	0.0105±0.0000	0.2856±0.0026	0.0104±0.0000
C. eyrei C. sclerophylla	0.5454±0.0007	0.0175±0.0000	0.5591±0.0017	0.0169±0.0000	0.5542±0.0034	0.0168±0.0000
C. eyrei_C. tibetana	0.5231±0.0007	0.0165±0.0000	0.5363±0.0019	0.0159±0.0000	0.5307±0.0037	0.0158±0.0000
C. fabri_C.fargesii	0.4036±0.0007	0.0138±0.0000	0.4167±0.0017	0.0132±0.0000	0.4099±0.0034	0.0131±0.0000
C. fabri C.fissa	0.6861±0.0006	0.0214±0.0000	0.6970±0.0014	0.0207±0.0000	0.6923±0.0028	0.0206±0.0001
C. fabri_C.fordii	0.5409±0.0007	0.0179±0.0000	0.5544±0.0018	0.0172±0.0000	0.5474±0.0036	0.0171±0.0001
C. fabri C.hystrix	0.5054±0.0007	0.0179±0.0000	0.5207±0.0018	0.0173±0.0000	0.5151±0.0035	0.0172±0.0001
C. fabri C.jucunda	0.6190±0.0007	0.0172±0.0000	0.6322±0.0017	0.0166±0.0000	0.6266±0.0033	0.0166±0.0001
C. fabri_C.lamontii	0.3772±0.0006	0.0126±0.0000	0.3920±0.0016	0.0120±0.0000	0.3865±0.0030	0.0119±0.0000
C. fabri C.sclerophylla	0.5736±0.0007	0.0184±0.0000	0.5870±0.0017	0.0177±0.0000	0.5809±0.0034	0.0176±0.0001
C. fabri_C.tibetana	0.5527±0.0007	0.0173±0.0000	0.5656±0.0019	0.0166±0.0000	0.5601±0.0038	0.0165±0.0001
C. fargesii_C. fissa	0.6950±0.0006	0.0218±0.0000	0.7056±0.0014	0.0210±0.0000	0.7005±0.0028	0.0209±0.0001
C. fargesii C. fordii	0.5527±0.0007	0.0184±0.0000	0.5658±0.0019	0.0176±0.0000	0.5583±0.0037	0.0175±0.0001
C. fargesii_C. hystrix	0.5164±0.0007	0.0183±0.0000	0.5306±0.0018	0.0177±0.0000	0.5250±0.0036	0.0176±0.0001
C. fargesii_C. jucunda	0.6304±0.0007	0.0176±0.0000	0.6431±0.0017	0.0170±0.0000	0.6372±0.0034	0.0169±0.0001
C. fargesii C. lamontii	0.3750±0.0007	0.0125±0.0000	0.3881±0.0018	0.0119±0.0000	0.3811±0.0034	0.0119±0.0000
C. fargesii_C. sclerophylla	0.5828±0.0007	0.0187±0.0000	0.5956±0.0018	0.0181±0.0000	0.5897±0.0035	0.0180±0.0001
C. fargesii C. tibetana	0.5647±0.0007	0.0177±0.0000	0.5769±0.0019	0.0170±0.0000	0.5714±0.0039	0.0169±0.0001
C. fissa_C. fordii	0.7126±0.0006	0.0223±0.0000	0.7220±0.0015	0.0215±0.0000	0.7158±0.0029	0.0214±0.0001
C. fissa_C. hystrix	0.6848±0.0006	0.0225±0.0000	0.6952±0.0014	0.0217±0.0000	0.6900±0.0027	0.0216±0.0001
C. fissa C. jucunda	0.7711±0.0006	0.0215±0.0000	0.7805±0.0013	0.0208±0.0000	0.7742±0.0026	0.0207±0.0001
C. fissa_C. lamontii	0.6978±0.0006	0.0206±0.0000	0.7092±0.0014	0.0199±0.0000	0.7038±0.0027	0.0198±0.0001
C. fissa_C. sclerophylla	0.7246±0.0006	0.0224±0.0000	0.7342±0.0014	0.0217±0.0000	0.7290±0.0027	0.0216±0.0001
C. fissa C. tibetana	0.7226±0.0006	0.0218±0.0000	0.7317±0.0015	0.0210±0.0000	0.7252±0.0030	0.0210±0.0001
C. fordii_C. hystrix	0.3531±0.0006	0.0134±0.0000	0.3626±0.0016	0.0127±0.0000	0.3570±0.0031	0.0127±0.0001
C. fordii C. jucunda	0.6538±0.0007	0.0183±0.0000	0.6650±0.0018	0.0176±0.0000	0.6592±0.0035	0.0175±0.0001
C. fordii_C. lamontii	0.5470±0.0007	0.0172±0.0000	0.5608±0.0018	0.0165±0.0000	0.5545±0.0036	0.0164±0.0001
C. fordii_C. sclerophylla	0.5988±0.0007	0.0192±0.0000	0.6104±0.0018	0.0185±0.0000	0.6050±0.0037	0.0184±0.0001
C. fordii C. tibetana	0.4589±0.0007	0.0137±0.0000	0.4674±0.0019	0.0129±0.0000	0.4585±0.0037	0.0129±0.0001
C. hystrix_C. jucunda	0.6185±0.0007	0.0184±0.0000	0.6313±0.0017	0.0177±0.0000	0.6265±0.0034	0.0177±0.0001
C. hystrix_C. lamontii	0.5084±0.0007	0.0172±0.0000	0.5232±0.0018	0.0165±0.0000	0.5196±0.0034	0.0165±0.0001
C. hystrix C. sclerophylla	0.5644±0.0007	0.0193±0.0000	0.5777±0.0018	0.0186±0.0000	0.5723±0.0035	0.0186±0.0001
C. hystrix_C. tibetana	0.4112±0.0007	0.0138±0.0000	0.4201±0.0018	0.0131±0.0000	0.4127±0.0034	0.0130±0.0001
C. jucunda C. lamontii	0.6291±0.0007	0.0164±0.0000	0.6429±0.0017	0.0159±0.0000	0.6374±0.0033	0.0158±0.0000
C. jucunda_C. sclerophylla	0.5522±0.0007	0.0133±0.0000	0.5625±0.0017	0.0127±0.0000	0.5571±0.0033	0.0127±0.0000
C. jucunda_C. tibetana	0.6612±0.0007	0.0177±0.0000	0.6724±0.0019	0.0171±0.0000	0.6671±0.0037	0.0170±0.0001
C. lamontii C. sclerophylla	0.5786±0.0007	0.0176±0.0000	0.5924±0.0017	0.0169±0.0000	0.5863±0.0034	0.0169±0.0000
C. lamontii_C. tibetana	0.5578±0.0007	0.0165±0.0000	0.5712±0.0019	0.0159±0.0000	0.5661±0.0038	0.0158±0.0001
C. sclerophylla C. tibetana	0.6103±0.0007	0.0186±0.0000	0.6216±0.0019	0.0179±0.0000	0.6158±0.0039	0.0179±0.0001

Table S4 Summary statistics (mean ± standard error) of π, Tajima's *D* , and recombination rate, estimated in non-overlapping windows (10, 100, and 500 kb) for the 12 *Castanopsis* species. Each row represents one of the 12 species.

	**10 kb windows**			**100 kb windows**			**500 kb windows**		
**Speceis**	***π***	**Tajima's *D***	**Recombiantiaon rate**	***π***	**Tajima's *D***	**Recombiantiaon rate**	***π***	**Tajima's *D***	**Recombiantiaon rate**
C. carlesii	0.0080±0.0000	-1.3662±0.0021	0.0331±0.0002	0.0075±0.0000	-1.4156±0.0044	0.0307±0.0005	0.0075±0.0001	-1.4269±0.0064	0.0339±0.0008
C. chinensis	0.0071±0.0000	0.0238±0.0032	0.0394±0.0003	0.0065±0.0000	0.0389±0.0068	0.0406±0.0005	0.0065±0.0001	0.0513±0.0104	0.0451±0.0008
C. eyrei	0.0088±0.0000	-1.4544±0.0020	0.0230±0.0002	0.0082±0.0000	-1.5043±0.0042	0.0203±0.0003	0.0082±0.0001	-1.5159±0.0062	0.0228±0.0006
C.fabri	0.0086±0.0000	-1.5157±0.0020	0.0328±0.0002	0.0080±0.0000	-1.5647±0.0040	0.0312±0.0005	0.0080±0.0001	-1.5799±0.0057	0.0354±0.0008
C. fargesii	0.0086±0.0000	-1.0593±0.0024	0.0296±0.0002	0.0080±0.0000	-1.0997±0.0050	0.0280±0.0004	0.0080±0.0001	-1.1074±0.0077	0.0324±0.0007
C. fissa	0.0057±0.0000	0.0239±0.0036	0.0536±0.0003	0.0051±0.0000	0.1011±0.0072	0.0551±0.0006	0.0051±0.0001	0.1363±0.0103	0.0636±0.0010
C. fordii	0.0085±0.0000	-0.6687±0.0027	0.0392±0.0003	0.0078±0.0000	-0.6788±0.0055	0.0410±0.0005	0.0079±0.0001	-0.6746±0.0081	0.0466±0.0008
C. hystrix	0.0098±0.0000	-0.9286±0.0023	0.0461±0.0003	0.0092±0.0000	-0.9609±0.0047	0.0458±0.0006	0.0092±0.0001	-0.9663±0.0073	0.0520±0.0009
C. jucunda	0.0053±0.0000	0.2375±0.0039	0.0283±0.0002	0.0047±0.0000	0.2738±0.0085	0.0277±0.0005	0.0047±0.0001	0.3017±0.0127	0.0319±0.0007
C. lamontii	0.0077±0.0000	-1.2070±0.0022	0.0245±0.0002	0.0072±0.0000	-1.2483±0.0045	0.0223±0.0003	0.0072±0.0001	-1.2518±0.0065	0.0259±0.0006
C. sclerophylla	0.0077±0.0000	-0.3055±0.0029	0.0293±0.0002	0.0072±0.0000	-0.3092±0.0060	0.0304±0.0004	0.0072±0.0001	-0.2992±0.0089	0.0361±0.0007
C. tibetana	0.0076±0.0000	-0.2972±0.0033	0.0331±0.0002	0.0070±0.0000	-0.3049±0.0073	0.0342±0.0005	0.0070±0.0001	-0.2903±0.0118	0.0389±0.0007

Table S5 First principal component (PC1) loading values calculated across (A) 12 species for π, ρ, and Tajima's D or (B) 66 pairwise comparisons for F_{ST} and d_{XY}.
In Table S5A, the column "Species" indicates the species name, with each row representing one species.
In Table S5B, the column "Species-pairs" indicates the two species in each pairwise comparison, with each row representing a comparison.

Table S5A

	**10 kb windows**			**100 kb windows**			**500 kb windows**		
**Speceis**	**π**	**ρ**	**Tajima's *D***	**π**	**ρ**	**Tajima's *D***	**π**	**ρ**	**Tajima's *D***
C. carlesii	0.8518	0.6665	0.6286	0.8867	0.7078	0.6872	0.9379	0.7912	0.7587
C. chinensis	0.8005	0.5627	0.3345	0.8594	0.5308	0.4127	0.9236	0.5249	0.5053
C. eyrei	0.8540	0.6450	0.5624	0.8892	0.6904	0.5937	0.9383	0.7648	0.6469
C. fabri	0.8682	0.7097	0.5593	0.9089	0.7617	0.5958	0.9536	0.8274	0.6103
C. fargesii	0.8765	0.7327	0.6148	0.9136	0.7509	0.6650	0.9547	0.8199	0.7345
C. fissa	0.7410	0.5051	0.2631	0.8163	0.4348	0.3398	0.8980	0.4677	0.3775
C. fordii	0.8612	0.6740	0.4830	0.9025	0.6482	0.5698	0.9503	0.6758	0.6463
C. hystrix	0.8520	0.7234	0.5223	0.8847	0.7458	0.6038	0.9334	0.7977	0.6576
C. jucunda	0.7632	0.5475	0.2454	0.8227	0.4747	0.2463	0.8898	0.4552	0.2414
C. lamontii	0.8570	0.6829	0.5273	0.8927	0.7257	0.5758	0.9465	0.7657	0.6191
C. sclerophylla	0.8352	0.6471	0.4437	0.8767	0.6189	0.5128	0.9276	0.6269	0.5957
C. tibetana	0.8525	0.6692	0.4265	0.8934	0.6215	0.4909	0.9394	0.6102	0.5703

Table S5B

	**10 kb windows**		**100 kb windows**		**500 kb windows**	
**Comparisons**	F_{ST}	d_{XY}	F_{ST}	d_{XY}	F_{ST}	d_{XY}
**Species-pair**						
C. carlesii_C. chinensis	0.9002	0.8684	0.9431	0.8733	0.9751	0.8933
C. carlesii_C. eyrei	0.7638	0.7484	0.8517	0.6993	0.9239	0.6505
C.carlesii_C. fabri	0.7973	0.7565	0.8803	0.7346	0.9447	0.7054
C. carlesii_C. fargesii	0.5330	0.5666	0.6347	0.4126	0.7647	0.2356
C. carlesii_C. fissa	0.8757	0.8107	0.9259	0.8235	0.9625	0.8485
C. carlesii_C. fordii	0.9032	0.8729	0.9458	0.8795	0.9758	0.9119
C. carlesii_C. hystrix	0.8943	0.8797	0.9386	0.8864	0.9718	0.9141
C. carlesii_C. jucunda	0.9006	0.8583	0.9417	0.8575	0.9729	0.8731
C. carlesii_C. lamontii	0.7677	0.7326	0.8527	0.6905	0.9247	0.6196
C. carlesii_C. sclerophylla	0.9077	0.8767	0.9455	0.8747	0.9739	0.8928
C. carlesii_C. tibetana	0.9058	0.8664	0.9452	0.8700	0.9735	0.9059
C. chinensis_C. eyrei	0.8818	0.8684	0.9334	0.8865	0.9691	0.9176
C.chinensis_C. fabri	0.8953	0.8710	0.9440	0.8889	0.9766	0.9158
C. chinensis_C. fargesii	0.9063	0.8827	0.9487	0.8947	0.9782	0.9236
C. chinensis_C. fissa	0.8494	0.8095	0.9027	0.8324	0.9473	0.8552

C. chinensis_C. fordii	0.8965	0.8636	0.9412	0.8767	0.9733	0.8967
C. chinensis_C. hystrix	0.8890	0.8596	0.9362	0.8666	0.9707	0.8763
C. chinensis_C. jucunda	0.7864	0.6806	0.8550	0.6600	0.9183	0.6270
C. chinensis_C. lamontii	0.8896	0.8629	0.9386	0.8834	0.9733	0.9144
C. chinensis_C. sclerophylla	0.7847	0.6837	0.8589	0.6573	0.9262	0.6295
C. chinensis_C. tibetana	0.8957	0.8514	0.9392	0.8662	0.9698	0.8852
C.eyrei_C. fabri	0.7409	0.7281	0.8368	0.6623	0.9192	0.5703
C. eyrei_C. fargesii	0.7708	0.7419	0.8563	0.6888	0.9276	0.6150
C. eyrei_C. fissa	0.8637	0.8102	0.9216	0.8346	0.9600	0.8665
C. eyrei_C. fordii	0.8833	0.8696	0.9361	0.8839	0.9710	0.9084
C. eyrei_C. hystrix	0.8748	0.8825	0.9299	0.9000	0.9678	0.9302
C. eyrei_C. jucunda	0.8876	0.8574	0.9349	0.8697	0.9678	0.8991
C. eyrei_C. lamontii	0.6714	0.6830	0.7708	0.5861	0.8749	0.4540
C. eyrei_C. sclerophylla	0.8936	0.8764	0.9371	0.8893	0.9684	0.9134
C. eyrei_C. tibetana	0.8875	0.8618	0.9364	0.8725	0.9706	0.9012
C. fabri_C.fargesii	0.8086	0.7524	0.8888	0.7260	0.9493	0.6692
C. fabri_C.fissa	0.8738	0.8171	0.9254	0.8436	0.9631	0.8656
C. fabri_C.fordii	0.8964	0.8653	0.9444	0.8831	0.9749	0.9143
C. fabri_C.hystrix	0.8894	0.8759	0.9395	0.8954	0.9722	0.9264
C. fabri_C.jucunda	0.8977	0.8616	0.9425	0.8751	0.9720	0.9046
C. fabri_C.lamontii	0.7554	0.7169	0.8430	0.6593	0.9252	0.5722
C. fabri_C.sclerophylla	0.9066	0.8776	0.9488	0.8940	0.9765	0.9177
C. fabri_C.tibetana	0.8999	0.8611	0.9452	0.8768	0.9747	0.9052
C. fargesii_C. fissa	0.8865	0.8277	0.9354	0.8446	0.9679	0.8790
C. fargesii_C. fordii	0.9088	0.8792	0.9505	0.8890	0.9772	0.9126
C. fargesii_C. hystrix	0.9019	0.8891	0.9459	0.9000	0.9749	0.9293
C. fargesii_C. jucunda	0.9072	0.8730	0.9468	0.8765	0.9760	0.9014
C. fargesii_C. lamontii	0.7797	0.7267	0.8619	0.6758	0.9318	0.5822
C. fargesii_C. sclerophylla	0.9143	0.8868	0.9510	0.8930	0.9777	0.9158
C. fargesii_C. tibetana	0.9108	0.8738	0.9497	0.8793	0.9754	0.9099
C. fissa_C. fordii	0.8775	0.8339	0.9233	0.8562	0.9616	0.8885
C. fissa_C. hystrix	0.8713	0.8310	0.9212	0.8515	0.9593	0.8685
C. fissa_C. jucunda	0.8512	0.7964	0.9031	0.8188	0.9480	0.8455
C. fissa_C. lamontii	0.8679	0.8103	0.9198	0.8324	0.9583	0.8632
C. fissa_C. sclerophylla	0.8722	0.8180	0.9203	0.8396	0.9585	0.8708
C. fissa_C. tibetana	0.8808	0.8282	0.9253	0.8536	0.9594	0.8774
C. fordii_C. hystrix	0.7266	0.6960	0.8160	0.6436	0.9024	0.5608
C. fordii_C. jucunda	0.8954	0.8591	0.9380	0.8722	0.9690	0.8920
C. fordii_C. lamontii	0.8921	0.8637	0.9403	0.8812	0.9731	0.9050
C. fordii_C. sclerophylla	0.9052	0.8709	0.9456	0.8858	0.9735	0.9071

C. fordii_C. tibetana	0.7973	0.6863	0.8708	0.6170	0.9369	0.5372
C. hystrix_C. jucunda	0.8912	0.8544	0.9361	0.8606	0.9686	0.8676
C. hystrix_C. lamontii	0.8855	0.8781	0.9357	0.8959	0.9698	0.9270
C. hystrix_C. sclerophylla	0.9002	0.8687	0.9432	0.8770	0.9726	0.8880
C. hystrix_C. tibetana	0.7851	0.7128	0.8625	0.6623	0.9325	0.5951
C. jucunda_C. lamontii	0.8929	0.8524	0.9383	0.8671	0.9707	0.8974
C. jucunda_C. sclerophylla	0.7850	0.6693	0.8556	0.6239	0.9202	0.5751
C. jucunda_C. tibetana	0.8996	0.8482	0.9410	0.8638	0.9698	0.8856
C. lamontii_C. sclerophylla	0.8999	0.8714	0.9434	0.8846	0.9726	0.9095
C. lamontii_C. tibetana	0.8965	0.8568	0.9412	0.8701	0.9728	0.8993
C. sclerophylla_C. tibetana	0.9057	0.8603	0.9451	0.8738	0.9712	0.8991
**Contrasts nodes**						
node1	0.7536	0.6013	0.7292	0.6907	0.7323	0.8057
node2	0.8253	0.7369	0.8297	0.8193	0.8524	0.9008
node3	0.8790	0.8716	0.8854	0.9244	0.9042	0.9632
node4	0.8713	0.8553	0.8922	0.9163	0.9193	0.9610
node5	0.8110	0.7743	0.8383	0.8489	0.8823	0.9200
node6	0.8265	0.8527	0.8491	0.9036	0.8854	0.9535
node7	0.8544	0.9318	0.8117	0.9599	0.7453	0.9800
node8	0.7593	0.8154	0.7801	0.8760	0.8234	0.9321
node9	0.7826	0.8363	0.8057	0.8880	0.8389	0.9350
node10	0.8474	0.9515	0.7694	0.9692	0.6365	0.9833
node11	0.7602	0.8755	0.7218	0.9200	0.5929	0.9537

Table S6 Spearman's correlation coefficient values between summary statistics estimated for 66 pairwise comparisons and 11 representative contrasts generated using a phylogenetic correction method (see Materials and Methods). For each pair of species, π and ρ values were averaged from two species. The column "Species pairs" indicates the two species in each pairwise comparison, with each row representing a comparison. Red numbers indicate non-significance ($P > 0.05$).

Species pairs	π vs. d_{XY}	π vs. F_{ST}	π vs. ρ	π vs. gene density	d_{XY} vs. F_{ST}	F_{ST} vs. ρ	F_{ST} vs. Gene density	d a
**10 kb window**								
C. carlesii_C. chinensis	0.4178	-0.8134	0.4839	-0.2784	0.1265	-0.4797	0.0954	0.0104
C. carlesii_C. eyrei	0.7986	-0.6324	0.4595	-0.2933	-0.1156	-0.5420	0.0548	0.0034
C. carlesii_C. fabri	0.7345	-0.6955	0.4897	-0.2965	-0.0868	-0.5302	0.0626	0.0051
C. carlesii_C. fargesii	0.9618	-0.4587	0.4878	-0.2752	-0.2574	-0.4798	0.0184	0.0013
C. carlesii_C. fissa	0.4134	-0.8695	0.5088	-0.3013	0.0426	-0.4718	0.1309	0.0148
C. carlesii_C. fordii	0.5288	-0.8021	0.5167	-0.2981	0.0341	-0.4952	0.0990	0.0097
C. carlesii_C. hystrix	0.5329	-0.7629	0.5094	-0.3047	0.0849	-0.5351	0.0895	0.0090
C. carlesii_C. jucunda	0.3905	-0.8383	0.4574	-0.2761	0.1246	-0.4545	0.1061	0.0108
C. carlesii_C. lamontii	0.7580	-0.6631	0.4909	-0.2862	-0.0863	-0.5274	0.0574	0.0042
C. carlesii_C. sclerophylla	0.4510	-0.8135	0.5182	-0.2909	0.1034	-0.5271	0.0970	0.0106
C. carlesii_C. tibetana	0.4915	-0.8201	0.5113	-0.2815	0.0450	-0.4879	0.0930	0.0096
C. chinensis_C. eyrei	0.4975	-0.7875	0.4858	-0.2878	0.0750	-0.4704	0.0975	0.0093
C. chinensis_C. fabri	0.4697	-0.8150	0.5117	-0.2853	0.0618	-0.4965	0.1007	0.0104
C. chinensis_C. fargesii	0.4549	-0.8312	0.5191	-0.2803	0.0520	-0.4862	0.0998	0.0107
C. chinensis_C. fissa	0.3622	-0.9061	0.5184	-0.2807	-0.0043	-0.4528	0.1447	0.0162
C. chinensis_C. fordii	0.4434	-0.8518	0.5231	-0.2825	0.0354	-0.4453	0.1149	0.0114
C. chinensis_C. hystrix	0.4499	-0.8090	0.5299	-0.2908	0.1023	-0.4853	0.1018	0.0108
C. chinensis_C. jucunda	0.5918	-0.8245	0.4793	-0.2534	-0.1077	-0.3630	0.0887	0.0078
C. chinensis_C. lamontii	0.4587	-0.8172	0.5069	-0.2754	0.0732	-0.4797	0.0975	0.0100
C. chinensis_C. sclerophylla	0.6536	-0.7671	0.5260	-0.2690	-0.0866	-0.4129	0.0811	0.0064
C. chinensis_C. tibetana	0.4058	-0.8642	0.5151	-0.2650	0.0435	-0.4358	0.1079	0.0113
C. eyrei_C. fabri	0.8109	-0.6466	0.4931	-0.3051	-0.1449	-0.5302	0.0567	0.0040
C. eyrei_C. fargesii	0.8004	-0.6552	0.5134	-0.2951	-0.1423	-0.5350	0.0510	0.0038
C. eyrei_C. fissa	0.4800	-0.8476	0.5150	-0.3117	0.0089	-0.4623	0.1299	0.0138
C. eyrei_C. fordii	0.5934	-0.7725	0.5243	-0.3070	0.0007	-0.4861	0.0963	0.0087
C. eyrei_C. hystrix	0.6005	-0.7367	0.5161	-0.3136	0.0389	-0.5312	0.0909	0.0080
C. eyrei_C. jucunda	0.4725	-0.8123	0.4650	-0.2885	0.0758	-0.4563	0.1075	0.0097
C. eyrei_C. lamontii	0.8568	-0.5786	0.4968	-0.2936	-0.1546	-0.5238	0.0357	0.0029
C. eyrei_C. sclerophylla	0.5238	-0.7907	0.5287	-0.2990	0.0552	-0.5305	0.0954	0.0095
C. eyrei_C. tibetana	0.5593	-0.7932	0.5188	-0.2906	0.0076	-0.4822	0.0927	0.0086
C. fabri_C. fargesii	0.7476	-0.7186	0.5355	-0.2967	-0.1341	-0.5383	0.0643	0.0054
C. fabri_C. fissa	0.4547	-0.8683	0.5320	-0.3069	-0.0008	-0.4823	0.1351	0.0147
C. fabri_C. fordii	0.5664	-0.8009	0.5446	-0.3038	-0.0090	-0.5108	0.1029	0.0096
C. fabri_C. hystrix	0.5728	-0.7654	0.5394	-0.3103	0.0337	-0.5496	0.0950	0.0089
C. fabri_C. jucunda	0.4430	-0.8387	0.4902	-0.2862	0.0664	-0.4729	0.1112	0.0107

C. fabri_C. lamontii	0.7795	-0.6793	0.5212	-0.2952	-0.1339	-0.5345	0.0598	0.0046
C. fabri_C. sclerophylla	0.5014	-0.8146	0.5496	-0.2977	0.0446	-0.5494	0.1022	0.0105
C. fabri_C. tibetana	0.5349	-0.8196	0.5411	-0.2888	-0.0032	-0.5041	0.0963	0.0095
C. fargesii_C. fissa	0.4424	-0.8809	0.5384	-0.3005	-0.0113	-0.4751	0.1330	0.0151
C. fargesii_C. fordii	0.5568	-0.8169	0.5513	-0.2977	-0.0230	-0.4980	0.1010	0.0100
C. fargesii_C. hystrix	0.5616	-0.7823	0.5465	-0.3056	0.0222	-0.5384	0.0920	0.0093
C. fargesii_C. jucunda	0.4273	-0.8548	0.5067	-0.2783	0.0562	-0.4709	0.1089	0.0111
C. fargesii_C. lamontii	0.7677	-0.6863	0.5375	-0.2873	-0.1282	-0.5272	0.0575	0.0045
C. fargesii_C. sclerophylla	0.4854	-0.8308	0.5625	-0.2915	0.0364	-0.5394	0.0994	0.0108
C. fargesii_C. tibetana	0.5240	-0.8343	0.5492	-0.2813	-0.0151	-0.4923	0.0948	0.0099
C. fissa_C. fordii	0.4502	-0.9032	0.5384	-0.3002	-0.0647	-0.4540	0.1485	0.0157
C. fissa_C. hystrix	0.4628	-0.8671	0.5487	-0.3103	-0.0081	-0.4872	0.1368	0.0152
C. fissa_C. jucunda	0.3275	-0.9248	0.5115	-0.2784	0.0125	-0.4205	0.1422	0.0165
C. fissa_C. lamontii	0.4436	-0.8731	0.5310	-0.2989	0.0039	-0.4732	0.1302	0.0143
C. fissa_C. sclerophylla	0.4037	-0.8989	0.5509	-0.2938	-0.0063	-0.4647	0.1407	0.0162
C. fissa_C. tibetana	0.4126	-0.9130	0.5351	-0.2838	-0.0483	-0.4430	0.1360	0.0157
C. fordii_C. hystrix	0.8236	-0.6615	0.5574	-0.3021	-0.1879	-0.4466	0.0699	0.0045
C. fordii_C. jucunda	0.4118	-0.8714	0.5099	-0.2813	0.0405	-0.4144	0.1186	0.0118
C. fordii_C. lamontii	0.5595	-0.8012	0.5457	-0.2964	-0.0018	-0.4957	0.0994	0.0093
C. fordii_C. sclerophylla	0.4736	-0.8436	0.5586	-0.2934	0.0270	-0.4762	0.1125	0.0114
C. fordii_C. tibetana	0.7651	-0.7743	0.5503	-0.2794	-0.2413	-0.4058	0.0826	0.0059
C. hystrix_C. jucunda	0.4225	-0.8272	0.5128	-0.2910	0.1060	-0.4547	0.1101	0.0112
C. hystrix_C. lamontii	0.5673	-0.7618	0.5396	-0.3048	0.0453	-0.5384	0.0934	0.0086
C. hystrix_C. sclerophylla	0.4799	-0.8035	0.5612	-0.3012	0.0843	-0.5225	0.1034	0.0109
C. hystrix_C. tibetana	0.7816	-0.7216	0.5542	-0.2886	-0.1922	-0.4492	0.0699	0.0053
C. jucunda_C. lamontii	0.4293	-0.8418	0.4888	-0.2759	0.0765	-0.4592	0.1071	0.0103
C. jucunda_C. sclerophylla	0.6419	-0.7978	0.5238	-0.2677	-0.1080	-0.3820	0.0917	0.0072
C. jucunda_C. tibetana	0.3709	-0.8877	0.5108	-0.2630	0.0519	-0.4161	0.1127	0.0117
C. lamontii_C. sclerophylla	0.4899	-0.8163	0.5531	-0.2886	0.0558	-0.5356	0.0979	0.0101
C. lamontii_C. tibetana	0.5250	-0.8205	0.5402	-0.2799	0.0059	-0.4872	0.0944	0.0091
C. sclerophylla_C. tibetana	0.4366	-0.8594	0.5601	-0.2777	0.0388	-0.4715	0.1087	0.0113
node1	0.9618	-0.4587	0.4878	-0.2752	-0.2574	-0.4798	0.0184	0.0013
node2	0.8568	-0.5786	0.4968	-0.2936	-0.1546	-0.5238	0.0357	0.0029
node3	0.8098	-0.6750	0.5354	-0.3006	-0.1760	-0.5908	0.0579	0.0040
node4	0.7966	-0.6999	0.5403	-0.3078	-0.1796	-0.5831	0.0638	0.0048
node5	0.8236	-0.6615	0.5574	-0.3021	-0.1879	-0.4466	0.0699	0.0045
node6	0.7929	-0.7588	0.5774	-0.2904	-0.2575	-0.4604	0.0795	0.0056
node7	0.6027	-0.7890	0.6078	-0.3185	-0.0332	-0.5913	0.1027	0.0093
node8	0.6536	-0.7671	0.5260	-0.2690	-0.0866	-0.4129	0.0811	0.0064
node9	0.6466	-0.8213	0.5259	-0.2703	-0.1567	-0.4042	0.0958	0.0075
node10	0.5173	-0.8325	0.6192	-0.3121	-0.0040	-0.5913	0.1162	0.0108
node11	0.4600	-0.8833	0.5873	-0.3163	-0.0313	-0.5236	0.1478	0.0154

100 kb window

C. carlesii_C. chinensis	0.1527	-0.8953	0.4637	-0.2516	0.2455	-0.4322	0.0852	0.0103
C. carlesii_C. eyrei	0.6972	-0.7721	0.5098	-0.2547	-0.1562	-0.5826	0.0551	0.0033
C. carlesii_C. fabri	0.6484	-0.8102	0.5383	-0.2695	-0.1381	-0.5666	0.0600	0.0049
C. carlesii_C. fargesii	0.9537	-0.5992	0.5080	-0.2440	-0.3787	-0.4737	0.0208	0.0012
C. carlesii_C. fissa	0.1900	-0.9183	0.4008	-0.2703	0.1702	-0.3416	0.1123	0.0145
C. carlesii_C. fordii	0.3316	-0.8792	0.4642	-0.2717	0.1078	-0.4287	0.0900	0.0095
C. carlesii_C. hystrix	0.2958	-0.8520	0.5136	-0.2782	0.1900	-0.5260	0.0731	0.0089
C. carlesii_C. jucunda	0.1135	-0.9130	0.3967	-0.2413	0.2558	-0.3758	0.0961	0.0106
C. carlesii_C. lamontii	0.6633	-0.7936	0.5309	-0.2526	-0.1367	-0.5550	0.0550	0.0041
C. carlesii_C. sclerophylla	0.1938	-0.8901	0.4795	-0.2572	0.2241	-0.4732	0.0872	0.0104
C. carlesii_C. tibetana	0.2853	-0.8973	0.4476	-0.2459	0.1200	-0.4099	0.0790	0.0094
C. chinensis_C. eyrei	0.2806	-0.8810	0.4643	-0.2586	0.1431	-0.4223	0.0863	0.0092
C. chinensis_C. fabri	0.2496	-0.8976	0.4987	-0.2669	0.1396	-0.4636	0.1048	0.0102
C. chinensis_C. fargesii	0.2224	-0.9044	0.4667	-0.2536	0.1540	-0.4111	0.0919	0.0105
C. chinensis_C. fissa	0.1626	-0.9432	0.3502	-0.2611	0.1122	-0.2719	0.1317	0.0158
C. chinensis_C. fordii	0.2265	-0.9145	0.4076	-0.2670	0.1366	-0.3173	0.1199	0.0111
C. chinensis_C. hystrix	0.1785	-0.8863	0.4839	-0.2738	0.2421	-0.4209	0.1004	0.0106
C. chinensis_C. jucunda	0.5380	-0.8956	0.3341	-0.2302	-0.1683	-0.2247	0.0818	0.0076
C. chinensis_C. lamontii	0.2371	-0.8983	0.4669	-0.2522	0.1550	-0.4128	0.0897	0.0098
C. chinensis_C. sclerophylla	0.5605	-0.8555	0.4049	-0.2493	-0.1105	-0.2974	0.0815	0.0063
C. chinensis_C. tibetana	0.1844	-0.9248	0.3859	-0.2418	0.1462	-0.3044	0.1046	0.0110
C. eyrei_C. fabri	0.7679	-0.7706	0.5558	-0.2740	-0.2411	-0.5769	0.0543	0.0039
C. eyrei_C. fargesii	0.7126	-0.7864	0.5377	-0.2544	-0.1937	-0.5535	0.0519	0.0037
C. eyrei_C. fissa	0.3035	-0.9022	0.3865	-0.2759	0.0895	-0.3138	0.1065	0.0134
C. eyrei_C. fordii	0.4380	-0.8599	0.4641	-0.2761	0.0305	-0.4161	0.0843	0.0085
C. eyrei_C. hystrix	0.4042	-0.8319	0.5228	-0.2828	0.1100	-0.5236	0.0693	0.0079
C. eyrei_C. jucunda	0.2470	-0.8992	0.4005	-0.2484	0.1509	-0.3735	0.0985	0.0095
C. eyrei_C. lamontii	0.8141	-0.7212	0.5553	-0.2553	-0.2551	-0.5671	0.0296	0.0028
C. eyrei_C. sclerophylla	0.3131	-0.8768	0.4885	-0.2606	0.1288	-0.4760	0.0839	0.0093
C. eyrei_C. tibetana	0.3942	-0.8798	0.4455	-0.2512	0.0409	-0.3955	0.0768	0.0084
C. fabri_C. fargesii	0.6723	-0.8243	0.5539	-0.2682	-0.1901	-0.5423	0.0598	0.0052
C. fabri_C. fissa	0.2648	-0.9168	0.4274	-0.2825	0.0959	-0.3582	0.1204	0.0143
C. fabri_C. fordii	0.4047	-0.8782	0.5008	-0.2842	0.0331	-0.4568	0.1008	0.0094
C. fabri_C. hystrix	0.3683	-0.8506	0.5484	-0.2915	0.1168	-0.5469	0.0863	0.0088
C. fabri_C. jucunda	0.2030	-0.9145	0.4394	-0.2571	0.1644	-0.4045	0.1096	0.0105
C. fabri_C. lamontii	0.7321	-0.7939	0.5684	-0.2691	-0.2218	-0.5602	0.0618	0.0045
C. fabri_C. sclerophylla	0.2859	-0.8906	0.5202	-0.2741	0.1307	-0.5079	0.1021	0.0103
C. fabri_C. tibetana	0.3609	-0.8949	0.4818	-0.2604	0.0457	-0.4339	0.0896	0.0093
C. fargesii_C. fissa	0.2434	-0.9254	0.3864	-0.2679	0.1000	-0.3043	0.1128	0.0147
C. fargesii_C. fordii	0.3875	-0.8875	0.4706	-0.2712	0.0328	-0.4062	0.0930	0.0098
C. fargesii_C. hystrix	0.3503	-0.8633	0.5309	-0.2769	0.1134	-0.5037	0.0758	0.0092
C. fargesii_C. jucunda	0.1829	-0.9216	0.4067	-0.2405	0.1686	-0.3563	0.1006	0.0108
C. fargesii_C. lamontii	0.6873	-0.8082	0.5500	-0.2517	-0.1857	-0.5274	0.0548	0.0044

C. fargesii_C. sclerophylla	0.2587	-0.9017	0.4931	-0.2575	0.1379	-0.4583	0.0902	0.0106
C. fargesii_C. tibetana	0.3495	-0.9042	0.4479	-0.2466	0.0403	-0.3822	0.0816	0.0097
C. fissa_C. fordii	0.2774	-0.9378	0.3414	-0.2794	0.0353	-0.2446	0.1379	0.0153
C. fissa_C. hystrix	0.2533	-0.9116	0.4285	-0.2873	0.1181	-0.3426	0.1221	0.0148
C. fissa_C. jucunda	0.1112	-0.9532	0.2811	-0.2471	0.1568	-0.1819	0.1190	0.0161
C. fissa_C. lamontii	0.2535	-0.9216	0.3923	-0.2681	0.1004	-0.3115	0.1093	0.0140
C. fissa_C. sclerophylla	0.1950	-0.9357	0.3380	-0.2680	0.1218	-0.2449	0.1240	0.0158
C. fissa_C. tibetana	0.2433	-0.9468	0.3199	-0.2546	0.0445	-0.2229	0.1230	0.0153
C. fordii_C. hystrix	0.7713	-0.7742	0.4834	-0.2835	-0.2549	-0.3696	0.0694	0.0043
C. fordii_C. jucunda	0.1820	-0.9280	0.3338	-0.2543	0.1573	-0.2387	0.1190	0.0115
C. fordii_C. lamontii	0.3981	-0.8810	0.4731	-0.2713	0.0371	-0.4131	0.0926	0.0091
C. fordii_C. sclerophylla	0.2550	-0.9054	0.4164	-0.2733	0.1365	-0.3292	0.1117	0.0112
C. fordii_C. tibetana	0.7470	-0.8573	0.3888	-0.2564	-0.3380	-0.2675	0.0830	0.0057
C. hystrix_C. jucunda	0.1404	-0.9003	0.4341	-0.2626	0.2553	-0.3589	0.1028	0.0110
C. hystrix_C. lamontii	0.3627	-0.8520	0.5363	-0.2794	0.1223	-0.5177	0.0763	0.0085
C. hystrix_C. sclerophylla	0.2114	-0.8759	0.4964	-0.2795	0.2338	-0.4456	0.0980	0.0106
C. hystrix_C. tibetana	0.7332	-0.8191	0.4704	-0.2630	-0.2602	-0.3629	0.0683	0.0051
C. jucunda_C. lamontii	0.1881	-0.9171	0.4081	-0.2410	0.1750	-0.3567	0.0973	0.0101
C. jucunda_C. sclerophylla	0.5816	-0.8794	0.3382	-0.2386	-0.1721	-0.2158	0.0907	0.0069
C. jucunda_C. tibetana	0.1447	-0.9411	0.3111	-0.2301	0.1613	-0.2202	0.1093	0.0114
C. lamontii_C. sclerophylla	0.2696	-0.8937	0.4956	-0.2576	0.1435	-0.4602	0.0888	0.0099
C. lamontii_C. tibetana	0.3553	-0.8987	0.4486	-0.2472	0.0466	-0.3841	0.0811	0.0089
C. sclerophylla_C. tibetana	0.2127	-0.9210	0.3884	-0.2490	0.1440	-0.3009	0.1053	0.0110
node1	0.9537	-0.5992	0.5080	-0.2440	-0.3787	-0.4737	0.0208	0.0012
node2	0.8141	-0.7212	0.5553	-0.2553	-0.2551	-0.5671	0.0296	0.0028
node3	0.7176	-0.8022	0.5732	-0.2603	-0.2198	-0.6048	0.0539	0.0039
node4	0.7304	-0.8110	0.5874	-0.2767	-0.2436	-0.6048	0.0587	0.0046
node5	0.7713	-0.7742	0.4834	-0.2835	-0.2549	-0.3696	0.0694	0.0043
node6	0.7573	-0.8459	0.4579	-0.2638	-0.3329	-0.3421	0.0774	0.0054
node7	0.4003	-0.8742	0.5716	-0.2836	0.0453	-0.5265	0.0886	0.0090
node8	0.5605	-0.8555	0.4049	-0.2493	-0.1105	-0.2974	0.0815	0.0063
node9	0.5880	-0.8952	0.3652	-0.2409	-0.2147	-0.2473	0.0912	0.0073
node10	0.2529	-0.9085	0.5602	-0.2761	0.1290	-0.4978	0.1070	0.0105
node11	0.2479	-0.9288	0.4175	-0.2823	0.0898	-0.3277	0.1286	0.0149
**500 kb window**								
C. carlesii_C. chinensis	-0.1072	-0.9432	0.5508	-0.2950	0.3941	-0.5518	0.1577	0.0100
C. carlesii_C. eyrei	0.6557	-0.8788	0.6338	-0.3062	-0.2769	-0.7070	0.1419	0.0032
C. carlesii_C. fabri	0.6077	-0.8890	0.6532	-0.3084	-0.2279	-0.6907	0.1361	0.0048
C. carlesii_C. fargesii	0.9658	-0.7503	0.6320	-0.2981	-0.5807	-0.5969	0.1049	0.0012
C. carlesii_C. fissa	-0.1065	-0.9536	0.4259	-0.2877	0.3694	-0.3961	0.1546	0.0143
C. carlesii_C. fordii	0.1466	-0.9349	0.5654	-0.3059	0.1742	-0.5498	0.1618	0.0093
C. carlesii_C. hystrix	0.0577	-0.9106	0.6269	-0.3201	0.3162	-0.6545	0.1454	0.0087
C. carlesii_C. jucunda	-0.1359	-0.9510	0.4901	-0.2820	0.4065	-0.4789	0.1577	0.0104

C. carlesii_C. lamontii	0.6388	-0.8911	0.6537	-0.2953	-0.2732	-0.6748	0.1306	0.0040
C. carlesii_C. sclerophylla	-0.0562	-0.9380	0.5669	-0.3055	0.3639	-0.5749	0.1565	0.0101
C. carlesii_C. tibetana	0.1061	-0.9427	0.5007	-0.2983	0.1940	-0.4918	0.1601	0.0092
C. chinensis_C. eyrei	0.0879	-0.9336	0.5468	-0.3015	0.2290	-0.5287	0.1549	0.0090
C. chinensis_C. fabri	0.0252	-0.9434	0.5837	-0.2978	0.2635	-0.5722	0.1607	0.0100
C. chinensis_C. fargesii	0.0038	-0.9498	0.5537	-0.2937	0.2686	-0.5290	0.1629	0.0103
C. chinensis_C. fissa	-0.1018	-0.9664	0.3172	-0.2763	0.3078	-0.2615	0.1577	0.0156
C. chinensis_C. fordii	0.0070	-0.9534	0.4529	-0.2988	0.2588	-0.3937	0.1781	0.0110
C. chinensis_C. hystrix	-0.0896	-0.9354	0.5600	-0.3081	0.4000	-0.5277	0.1648	0.0104
C. chinensis_C. jucunda	0.5244	-0.9423	0.3336	-0.2659	-0.2532	-0.2524	0.1491	0.0075
C. chinensis_C. lamontii	0.0371	-0.9451	0.5540	-0.2875	0.2493	-0.5260	0.1463	0.0096
C. chinensis_C. sclerophylla	0.5406	-0.9220	0.4427	-0.2922	-0.2178	-0.3664	0.1642	0.0061
C. chinensis_C. tibetana	-0.0195	-0.9607	0.3918	-0.2885	0.2571	-0.3328	0.1795	0.0108
C. eyrei_C. fabri	0.7707	-0.8638	0.6770	-0.3135	-0.3971	-0.6941	0.1330	0.0038
C. eyrei_C. fargesii	0.6839	-0.8891	0.6647	-0.3063	-0.3294	-0.6822	0.1405	0.0036
C. eyrei_C. fissa	0.0688	-0.9459	0.4049	-0.2950	0.2230	-0.3609	0.1552	0.0132
C. eyrei_C. fordii	0.3069	-0.9246	0.5658	-0.3122	0.0359	-0.5409	0.1575	0.0083
C. eyrei_C. hystrix	0.2265	-0.9016	0.6338	-0.3244	0.1688	-0.6555	0.1411	0.0077
C. eyrei_C. jucunda	0.0425	-0.9456	0.4770	-0.2882	0.2497	-0.4604	0.1562	0.0093
C. eyrei_C. lamontii	0.8338	-0.8421	0.6873	-0.3000	-0.4623	-0.6856	0.0941	0.0028
C. eyrei_C. sclerophylla	0.1284	-0.9307	0.5751	-0.3111	0.2043	-0.5655	0.1569	0.0092
C. eyrei_C. tibetana	0.2761	-0.9342	0.5034	-0.3042	0.0457	-0.4826	0.1586	0.0082
C. fabri_C. fargesii	0.6513	-0.9021	0.6738	-0.3048	-0.3079	-0.6783	0.1433	0.0051
C. fabri_C. fissa	-0.0052	-0.9518	0.4552	-0.2915	0.2747	-0.4176	0.1538	0.0141
C. fabri_C. fordii	0.2430	-0.9341	0.5980	-0.3087	0.0805	-0.5728	0.1574	0.0092
C. fabri_C. hystrix	0.1591	-0.9137	0.6578	-0.3190	0.2119	-0.6726	0.1419	0.0086
C. fabri_C. jucunda	-0.0264	-0.9536	0.5190	-0.2839	0.2935	-0.4987	0.1557	0.0103
C. fabri_C. lamontii	0.7458	-0.8784	0.6814	-0.2988	-0.3851	-0.6733	0.1198	0.0044
C. fabri_C. sclerophylla	0.0624	-0.9393	0.6027	-0.3097	0.2474	-0.5952	0.1593	0.0101
C. fabri_C. tibetana	0.2205	-0.9404	0.5450	-0.3033	0.0854	-0.5174	0.1571	0.0091
C. fargesii_C. fissa	-0.0151	-0.9588	0.4151	-0.2868	0.2679	-0.3736	0.1619	0.0145
C. fargesii_C. fordii	0.2387	-0.9411	0.5656	-0.3026	0.0660	-0.5285	0.1613	0.0096
C. fargesii_C. hystrix	0.1546	-0.9216	0.6440	-0.3145	0.1967	-0.6398	0.1472	0.0090
C. fargesii_C. jucunda	-0.0306	-0.9584	0.4948	-0.2808	0.2854	-0.4651	0.1619	0.0107
C. fargesii_C. lamontii	0.6753	-0.8996	0.6761	-0.2930	-0.3354	-0.6622	0.1346	0.0043
C. fargesii_C. sclerophylla	0.0572	-0.9440	0.5810	-0.3046	0.2404	-0.5620	0.1601	0.0104
C. fargesii_C. tibetana	0.2053	-0.9470	0.5130	-0.2965	0.0829	-0.4780	0.1596	0.0095
C. fissa_C. fordii	0.0473	-0.9628	0.3391	-0.2920	0.1935	-0.2704	0.1723	0.0151
C. fissa_C. hystrix	-0.0256	-0.9474	0.4563	-0.3017	0.3094	-0.4026	0.1522	0.0146
C. fissa_C. jucunda	-0.1605	-0.9754	0.2382	-0.2610	0.3493	-0.1853	0.1475	0.0159
C. fissa_C. lamontii	0.0168	-0.9556	0.4182	-0.2787	0.2484	-0.3709	0.1460	0.0138
C. fissa_C. sclerophylla	-0.0640	-0.9617	0.3087	-0.2892	0.3046	-0.2453	0.1659	0.0156
C. fissa_C. tibetana	0.0204	-0.9694	0.2827	-0.2837	0.1965	-0.2143	0.1719	0.0151

C. fordii_C. hystrix	0.7693	-0.8704	0.5576	-0.3157	-0.4062	-0.4757	0.1333	0.0042
C. fordii_C. jucunda	-0.0370	-0.9610	0.3790	-0.2838	0.2841	-0.3092	0.1717	0.0113
C. fordii_C. lamontii	0.2601	-0.9347	0.5767	-0.2994	0.0615	-0.5368	0.1533	0.0089
C. fordii_C. sclerophylla	0.0394	-0.9477	0.4574	-0.3076	0.2515	-0.3941	0.1783	0.0110
C. fordii_C. tibetana	0.7728	-0.9172	0.4203	-0.2997	-0.4868	-0.3298	0.1581	0.0055
C. hystrix_C. jucunda	-0.1344	-0.9448	0.5083	-0.2942	0.4198	-0.4612	0.1616	0.0108
C. hystrix_C. lamontii	0.1785	-0.9125	0.6504	-0.3091	0.1945	-0.6465	0.1305	0.0083
C. hystrix_C. sclerophylla	-0.0504	-0.9302	0.5776	-0.3185	0.3794	-0.5529	0.1681	0.0104
C. hystrix_C. tibetana	0.7340	-0.8935	0.5139	-0.3106	-0.3927	-0.4524	0.1336	0.0050
C. jucunda_C. lamontii	-0.0089	-0.9552	0.4832	-0.2713	0.2733	-0.4488	0.1490	0.0099
C. jucunda_C. sclerophylla	0.5773	-0.9382	0.3506	-0.2769	-0.2956	-0.2666	0.1673	0.0068
C. jucunda_C. tibetana	-0.0822	-0.9698	0.3139	-0.2759	0.2989	-0.2529	0.1771	0.0113
C. lamontii_C. sclerophylla	0.0771	-0.9433	0.5709	-0.2972	0.2250	-0.5464	0.1499	0.0097
C. lamontii_C. tibetana	0.2258	-0.9444	0.5040	-0.2896	0.0703	-0.4680	0.1499	0.0088
C. sclerophylla_C. tibetana	0.0072	-0.9570	0.3890	-0.2972	0.2530	-0.3346	0.1808	0.0109
node1	0.9658	-0.7503	0.6320	-0.2981	-0.5807	-0.5969	0.1049	0.0012
node2	0.8338	-0.8421	0.6873	-0.3000	-0.4623	-0.6856	0.0941	0.0028
node3	0.6750	-0.8966	0.7001	-0.3038	-0.3334	-0.7276	0.1378	0.0038
node4	0.7073	-0.8905	0.7007	-0.3095	-0.3568	-0.7205	0.1335	0.0045
node5	0.7693	-0.8704	0.5576	-0.3157	-0.4062	-0.4757	0.1333	0.0042
node6	0.7650	-0.9096	0.4946	-0.3073	-0.4629	-0.4168	0.1481	0.0053
node7	0.2091	-0.9316	0.6630	-0.3152	0.1215	-0.6424	0.1559	0.0088
node8	0.5406	-0.9220	0.4427	-0.2922	-0.2178	-0.3664	0.1642	0.0061
node9	0.5666	-0.9461	0.3703	-0.2745	-0.3044	-0.2859	0.1637	0.0072
node10	-0.0120	-0.9513	0.6153	-0.3041	0.2885	-0.5746	0.1688	0.0103
node11	-0.0330	-0.9611	0.4062	-0.2920	0.2814	-0.3515	0.1666	0.0147

Table S7 Linear regression analyses of correlation coefficient values and divergence time (d_a) for 11 representative contrasts (refer to Table S6). Variables, pairwise comparison of summary statistics; Regression equation, linear regression fit to the 11 representative contrasts; Spearman's ρ, spearman's correlation coefficient; *P*-value, significance of Spearman's correlation test. Each row represents a pair of summary statistics analyzed in 10, 100, or 500 kb windows.

**Variables**	**Regression equation**	Spearman's ρ	***P*-value**
**10 kb**			
π vs. Gene density	$y = -2.28x - 0.281$	-0.4455	0.1728
π vs. Recombination rate	$y = 8.07x + 0.498$	0.7364	0.0134
π vs. d_{XY}	$y = -37.2x + 0.965$	-0.9909	< 2.2e-16
π vs. F_{ST}	$y = -27x - 0.543$	-0.9818	< 2.2e-16
F_{ST} vs. Gene density	$y = 8.87x + 0.0205$	0.9909	< 2.2e-16
F_{ST} vs. Recombination rate	$y = -3.43x - 0.487$	-0.1230	0.7186
F_{ST} vs. d_{XY}	$y = 17.8x - 0.256$	0.7091	1.87e-02
**100 kb**			
π vs. Gene density	$y = -2.12x - 0.252$	-0.3818	0.2466
π vs. Recombination rate	$y = -5.77x + 0.535$	-0.2818	0.4011
π vs. d_{XY}	$y = -56.7x + 0.98$	-0.9273	< 2.2e-16
π vs. F_{ST}	$y = -20.6x - 0.688$	-0.9818	< 2.2e-16
F_{ST} vs. Gene density	$y = 7.73x + 0.024$	0.9818	< 2.2e-16
F_{ST} vs. Recombination rate	$y = 11.4x - 0.515$	0.4282	0.1888
F_{ST} vs. d_{XY}	$y = 39.2x - 0.409$	0.8636	1.28e-03
**500 kb**			
π vs. Gene density	$y = 0.608x - 0.305$	0.1455	0.6696
π vs. Recombination rate	$y = -15.7x + 0.668$	-0.5364	0.089
π vs. d_{XY}	$y = -84.1x + 1.07$	-0.9273	< 2.2e-16
π vs. F_{ST}	$y = -12.8x - 0.817$	-0.9636	< 2.2e-16
F_{ST} vs. Gene density	$y = 5.17x + 0.11$	0.9182	0.0001
F_{ST} vs. Recombination rate	$y = 18.5x - 0.647$	0.5545	0.0767
F_{ST} vs. d_{XY}	$y = 74x - 0.684$	0.8636	1.28e-03

Table S8 Genome-wide estimates of gene flow in 220 trios using D-statistic and f_4-ratio. The D-statistic and f_4-ratio were calculated with a four-taxon framework (((P1, P2), P3), O). In the table, the columns "P1", "P2", and "P3" indicate the three ingroups in each trio, with *Castanea mollissima* serving as the outgroup. The "D-statistic" column displays the estimated D-values, where a negative D-value suggests gene flow between P1 and P3, and a positive D-value indicates gene flow between P2 and P3. The "f_4-ratio" column illustrates admixture proportion between each pair of species linked by gene flow. The columns "nBBAA", "nABBA", and "nBABA" show the total number of sites with patterns of BBAA, ABBA, BABA, respectively. Each row corresponds to a trio, with the first 51 rows (highlighted in bold) indicating the non-redundant trios selected for P2 and P3 species-pairs, tested using various P1 species (see Materials and Methods).

P1	P2	P3	D- statistic	f_4-ratio	FDR*	BBAA	ABBA	BABA
C. chinensis	C. sclerophylla	C. jucunda	0.1179	0.0639	0.0000	227439	199984	157801
C. fargesii	C. carlesii	C. fabri	0.0308	0.0165	0.0000	436870	106318	99974
C. fargesii	C. carlesii	C. fordii	0.0311	0.0065	0.0000	720202	87115	81867
C. fargesii	C. carlesii	C. hystrix	0.0375	0.0086	0.0000	713452	88864	82436
C. fargesii	C. carlesii	C. lamontii	0.0256	0.0191	0.0000	399737	109637	104173
C. fargesii	C. carlesii	C. tibetana	0.0331	0.0069	0.0000	712796	87370	81777
C. fargesii	C. eyrei	C. fabri	0.0295	0.0235	0.0000	205285	157824	148778
C. fargesii	C. eyrei	C. fordii	0.0463	0.0133	0.0000	470884	121934	111148
C. fargesii	C. eyrei	C. hystrix	0.0480	0.0152	0.0000	464594	123755	112415
C. fargesii	C. eyrei	C. tibetana	0.0520	0.0150	0.0000	462933	122746	110607
C. fargesii	C. lamontii	C. fabri	0.0127	0.0105	0.0378	198600	160667	156626
C. fargesii	C. lamontii	C. fordii	0.0172	0.0051	0.0011	463500	122794	118643
C. fordii	C. carlesii	C. chinensis	0.0528	0.0213	0.0000	230483	206092	185406
C. fordii	C. carlesii	C. fissa	0.0583	0.0123	0.0000	450465	148646	132261
C. fordii	C. carlesii	C. jucunda	0.0565	0.0205	0.0000	235650	202351	180703
C. fordii	C. carlesii	C. sclerophylla	0.0472	0.0192	0.0000	242508	206171	187581
C. fordii	C. chinensis	C. fissa	0.0158	0.0035	0.0000	415318	150660	145985
C. fordii	C. eyrei	C. chinensis	0.0706	0.0277	0.0000	228676	205141	178086
C. fordii	C. eyrei	C. fissa	0.0816	0.0166	0.0000	449660	147636	125367
C. fordii	C. eyrei	C. jucunda	0.0739	0.0261	0.0000	233726	201095	173413
C. fordii	C. eyrei	C. sclerophylla	0.0639	0.0253	0.0000	240718	204848	180238
C. fordii	C. fabri	C. chinensis	0.0372	0.0150	0.0000	231085	203359	188780
C. fordii	C. fabri	C. fissa	0.0539	0.0114	0.0000	449120	148465	133286
C. fordii	C. fabri	C. jucunda	0.0444	0.0161	0.0000	235752	200702	183650
C. fordii	C. fabri	C. sclerophylla	0.0341	0.0139	0.0000	242566	204035	190565
C. fordii	C. fargesii	C. chinensis	0.0356	0.0146	0.0000	232465	206745	192542
C. fordii	C. fargesii	C. fissa	0.0438	0.0094	0.0000	451089	150159	137564
C. fordii	C. fargesii	C. jucunda	0.0404	0.0149	0.0000	237390	203352	187560
C. fordii	C. fargesii	C. sclerophylla	0.0324	0.0134	0.0000	244107	207391	194387
C. fordii	C. hystrix	C. fabri	0.0269	0.0100	0.0000	552911	130135	123323
C. fordii	C. hystrix	C. fargesii	0.0311	0.0107	0.0000	556398	132516	124523
C. fordii	C. hystrix	C. jucunda	0.0308	0.0071	0.0000	593481	126040	118510
C. fordii	C. hystrix	C. lamontii	0.0242	0.0097	0.0000	550789	128218	122155
C. fordii	C. hystrix	C. sclerophylla	0.0295	0.0077	0.0000	599775	130143	122673
C. fordii	C. hystrix	C. tibetana	0.0354	0.0284	0.0000	249580	187878	175045
C. fordii	C. lamontii	C. chinensis	0.0525	0.0208	0.0000	227184	203569	183263
C. fordii	C. lamontii	C. fissa	0.0686	0.0141	0.0000	445892	147171	128278
C. fordii	C. lamontii	C. jucunda	0.0597	0.0213	0.0000	231579	200340	177777
C. fordii	C. lamontii	C. sclerophylla	0.0477	0.0191	0.0000	238684	203515	184985
C. fordii	C. tibetana	C. fabri	0.0237	0.0094	0.0000	486137	138098	131691
C. fordii	C. tibetana	C. fargesii	0.0278	0.0102	0.0000	489544	140333	132746
C. fordii	C. tibetana	C. fissa	0.0082	0.0013	0.0056	760695	102837	101160
C. fordii	C. tibetana	C. jucunda	0.0284	0.0070	0.0000	527228	133293	125939
C. fordii	C. tibetana	C. lamontii	0.0321	0.0137	0.0000	482651	137692	129130
C. fordii	C. tibetana	C. sclerophylla	0.0293	0.0081	0.0000	532968	138208	130346
C. jucunda	C. chinensis	C. fordii	0.0337	0.0104	0.0000	507701	134027	125299
C. jucunda	C. chinensis	C. hystrix	0.0389	0.0132	0.0000	501762	136817	126573
C. jucunda	C. chinensis	C. tibetana	0.0369	0.0115	0.0000	500477	134832	125243
C. lamontii	C. eyrei	C. carlesii	0.0662	0.0568	0.0000	168188	169861	148771
C. lamontii	C. eyrei	C. fargesii	0.0450	0.0356	0.0000	178485	167875	153404
C. sclerophylla	C. jucunda	C. fissa	0.0198	0.0029	0.0000	770199	98789	94953
C. carlesii	C. eyrei	C. chinensis	0.0308	0.0067	0.0000	486930	107805	101363
C. carlesii	C. eyrei	C. fissa	0.0361	0.0045	0.0000	734928	85257	79320
C. carlesii	C. eyrei	C. fordii	0.0245	0.0067	0.0000	474295	113128	107727
C. carlesii	C. eyrei	C. hystrix	0.0212	0.0064	0.0000	467518	114480	109717
C. carlesii	C. eyrei	C. jucunda	0.0295	0.0058	0.0000	494009	105135	99103
C. carlesii	C. eyrei	C. sclerophylla	0.0285	0.0063	0.0000	502929	108403	102387
C. carlesii	C. eyrei	C. tibetana	0.0287	0.0079	0.0000	466176	113772	107416
C. carlesii	C. lamontii	C. fissa	0.0150	0.0019	0.0000	719331	86899	84330
C. chinensis	C. carlesii	C. fissa	0.0401	0.0085	0.0000	419095	145921	134682
C. chinensis	C. eyrei	C. fissa	0.0625	0.0128	0.0000	418713	144576	127575
C. chinensis	C. fabri	C. fissa	0.0355	0.0076	0.0000	415229	146382	136345
C. chinensis	C. fargesii	C. fissa	0.0261	0.0057	0.0000	418738	147650	140145
C. chinensis	C. lamontii	C. fissa	0.0497	0.0103	0.0000	415140	144383	130723

C. fabri	*C. carlesii*	*C. chinensis*	0.0255	0.0063	0.0000	445967	122446	116350
C. fabri	*C. carlesii*	*C. hystrix*	0.0211	0.0073	0.0000	426260	131692	126252
C. fabri	*C. carlesii*	*C. jucunda*	0.0197	0.0044	0.0000	452186	119183	114584
C. fabri	*C. carlesii*	*C. sclerophylla*	0.0209	0.0053	0.0000	460858	122973	117926
C. fabri	*C. carlesii*	*C. tibetana*	0.0182	0.0058	0.0000	425306	129678	125041
C. fabri	*C. eyrei*	*C. chinensis*	0.0544	0.0130	0.0000	442466	121883	109315
C. fabri	*C. eyrei*	*C. fissa*	0.0397	0.0054	0.0000	686570	93523	86379
C. fabri	*C. eyrei*	*C. fordii*	0.0341	0.0104	0.0000	428990	127423	119031
C. fabri	*C. eyrei*	*C. hystrix*	0.0411	0.0137	0.0000	423171	129881	119637
C. fabri	*C. eyrei*	*C. jucunda*	0.0471	0.0102	0.0000	448770	118369	107716
C. fabri	*C. eyrei*	*C. sclerophylla*	0.0478	0.0116	0.0000	457471	122133	111000
C. fabri	*C. eyrei*	*C. tibetana*	0.0447	0.0137	0.0000	421644	129006	117972
C. fabri	*C. lamontii*	*C. chinensis*	0.0245	0.0059	0.0000	442129	119871	114134
C. fabri	*C. lamontii*	*C. fissa*	0.0208	0.0029	0.0000	684377	93092	89291
C. fabri	*C. lamontii*	*C. jucunda*	0.0241	0.0053	0.0000	447797	117447	111919
C. fabri	*C. lamontii*	*C. sclerophylla*	0.0216	0.0053	0.0000	456609	120536	115446
C. fabri	*C. lamontii*	*C. tibetana*	0.0157	0.0049	0.0000	421812	127438	123491
C. fargesii	*C. carlesii*	*C. chinensis*	0.0390	0.0065	0.0000	733837	84059	77742
C. fargesii	*C. carlesii*	*C. eyrei*	0.0550	0.0650	0.0000	385483	116058	103963
C. fargesii	*C. carlesii*	*C. fissa*	0.0283	0.0028	0.0000	982258	67273	63571
C. fargesii	*C. carlesii*	*C. jucunda*	0.0358	0.0054	0.0000	740863	81971	76307
C. fargesii	*C. carlesii*	*C. sclerophylla*	0.0332	0.0057	0.0000	750167	84542	79103
C. fargesii	*C. eyrei*	*C. chinensis*	0.0586	0.0134	0.0000	483883	116678	103763
C. fargesii	*C. eyrei*	*C. fissa*	0.0560	0.0073	0.0000	730238	91854	82116
C. fargesii	*C. eyrei*	*C. jucunda*	0.0550	0.0113	0.0000	490673	113729	101867
C. fargesii	*C. eyrei*	*C. sclerophylla*	0.0522	0.0121	0.0000	499496	117061	105438
C. fargesii	*C. lamontii*	*C. chinensis*	0.0269	0.0063	0.0000	476000	116643	110541
C. fargesii	*C. lamontii*	*C. fissa*	0.0356	0.0048	0.0000	720138	92600	86237
C. fargesii	*C. lamontii*	*C. jucunda*	0.0301	0.0064	0.0000	482278	114879	108157
C. fargesii	*C. lamontii*	*C. sclerophylla*	0.0244	0.0058	0.0000	491241	117569	111969
C. fargesii	*C. lamontii*	*C. tibetana*	0.0208	0.0062	0.0000	455717	123407	118382
C. fordii	*C. hystrix*	*C. carlesii*	0.0362	0.0130	0.0000	549173	131829	122613
C. fordii	*C. hystrix*	*C. chinensis*	0.0362	0.0093	0.0000	586125	129331	120299
C. fordii	*C. hystrix*	*C. eyrei*	0.0340	0.0161	0.0000	544558	130517	121932
C. fordii	*C. jucunda*	*C. fissa*	0.0183	0.0041	0.0000	405917	151669	146215
C. fordii	*C. tibetana*	*C. carlesii*	0.0296	0.0113	0.0000	482555	139096	131096
C. fordii	*C. tibetana*	*C. chinensis*	0.0309	0.0084	0.0000	519933	136496	128301
C. fordii	*C. tibetana*	*C. eyrei*	0.0336	0.0170	0.0000	477159	138790	129759
C. hystrix	*C. carlesii*	*C. chinensis*	0.0298	0.0121	0.0000	234234	200729	189093
C. hystrix	*C. carlesii*	*C. fissa*	0.0563	0.0117	0.0000	458093	147145	131470
C. hystrix	*C. carlesii*	*C. jucunda*	0.0367	0.0133	0.0000	239960	197515	183541
C. hystrix	*C. carlesii*	*C. sclerophylla*	0.0284	0.0116	0.0000	247028	201262	190150
C. hystrix	*C. chinensis*	*C. fissa*	0.0137	0.0031	0.0000	422796	149197	145164
C. hystrix	*C. eyrei*	*C. chinensis*	0.0473	0.0187	0.0000	231887	199936	181880
C. hystrix	*C. eyrei*	*C. fissa*	0.0797	0.0161	0.0000	456865	146359	124764
C. hystrix	*C. eyrei*	*C. jucunda*	0.0538	0.0190	0.0000	237522	196415	176371
C. hystrix	*C. eyrei*	*C. sclerophylla*	0.0448	0.0178	0.0000	244704	200067	182905
C. hystrix	*C. fabri*	*C. fissa*	0.0515	0.0108	0.0000	454839	147486	133049
C. hystrix	*C. fargesii*	*C. fissa*	0.0417	0.0089	0.0000	457761	148891	136958
C. hystrix	*C. jucunda*	*C. fissa*	0.0164	0.0037	0.0000	412285	150519	145661
C. hystrix	*C. lamontii*	*C. fissa*	0.0664	0.0136	0.0000	451185	146446	128198
C. hystrix	*C. lamontii*	*C. jucunda*	0.0394	0.0142	0.0000	233707	196473	181562
C. jucunda	*C. carlesii*	*C. fissa*	0.0373	0.0080	0.0000	411190	146521	135980
C. jucunda	*C. chinensis*	*C. carlesii*	0.0301	0.0105	0.0000	485499	130190	122582
C. jucunda	*C. chinensis*	*C. eyrei*	0.0312	0.0143	0.0000	479872	129480	121633
C. jucunda	*C. chinensis*	*C. fabri*	0.0250	0.0090	0.0000	489882	128577	122302
C. jucunda	*C. chinensis*	*C. fargesii*	0.0276	0.0093	0.0000	492671	130996	123959
C. jucunda	*C. chinensis*	*C. lamontii*	0.0256	0.0100	0.0000	484162	127686	121309
C. jucunda	*C. eyrei*	*C. fissa*	0.0592	0.0123	0.0000	410567	145208	128970
C. jucunda	*C. fabri*	*C. fissa*	0.0332	0.0071	0.0000	408416	146645	137233
C. jucunda	*C. fargesii*	*C. fissa*	0.0236	0.0052	0.0000	411304	148175	141335
C. jucunda	*C. lamontii*	*C. fissa*	0.0471	0.0099	0.0000	408063	144617	131605
C. lamontii	*C. eyrei*	*C. chinensis*	0.0333	0.0071	0.0000	483795	105562	98760
C. lamontii	*C. eyrei*	*C. fabri*	0.0172	0.0129	0.0000	204072	146015	141088
C. lamontii	*C. eyrei*	*C. fissa*	0.0209	0.0025	0.0000	730678	81465	78130
C. lamontii	*C. eyrei*	*C. fordii*	0.0305	0.0081	0.0000	471631	111619	105022
C. lamontii	*C. eyrei*	*C. hystrix*	0.0417	0.0122	0.0000	466009	114046	104906
C. lamontii	*C. eyrei*	*C. jucunda*	0.0256	0.0049	0.0000	490491	102591	97479
C. lamontii	*C. eyrei*	*C. sclerophylla*	0.0291	0.0063	0.0000	499555	106129	100118
C. lamontii	*C. eyrei*	*C. tibetana*	0.0326	0.0088	0.0000	463382	112159	105083
C. sclerophylla	*C. carlesii*	*C. fissa*	0.0517	0.0114	0.0000	417606	153457	138357
C. sclerophylla	*C. chinensis*	*C. carlesii*	0.0382	0.0126	0.0000	553511	125397	116163
C. sclerophylla	*C. chinensis*	*C. eyrei*	0.0401	0.0173	0.0000	547999	124785	115159
C. sclerophylla	*C. chinensis*	*C. fabri*	0.0340	0.0116	0.0000	558211	124065	115914
C. sclerophylla	*C. chinensis*	*C. fargesii*	0.0342	0.0109	0.0000	560629	126037	117706
C. sclerophylla	*C. chinensis*	*C. fissa*	0.0155	0.0022	0.0000	794961	96237	93303
C. sclerophylla	*C. chinensis*	*C. fordii*	0.0290	0.0086	0.0000	573864	127548	120347
C. sclerophylla	*C. chinensis*	*C. hystrix*	0.0356	0.0115	0.0000	567759	130173	121229
C. sclerophylla	*C. chinensis*	*C. lamontii*	0.0373	0.0137	0.0000	552598	123265	114395
C. sclerophylla	*C. chinensis*	*C. tibetana*	0.0306	0.0091	0.0000	566248	128114	120503

C. sclerophylla	*C. eyrei*	*C. fissa*	0.0737	0.0157	0.0000	416970	152197	131298
C. sclerophylla	*C. fabri*	*C. fissa*	0.0471	0.0104	0.0000	414309	153412	139608
C. sclerophylla	*C. fargesii*	*C. fissa*	0.0379	0.0085	0.0000	417894	155001	143667
C. sclerophylla	*C. lamontii*	*C. fissa*	0.0613	0.0132	0.0000	413797	151647	134131
C. tibetana	*C. carlesii*	*C. chinensis*	0.0324	0.0130	0.0000	231236	199099	186589
C. tibetana	*C. carlesii*	*C. fissa*	0.0537	0.0110	0.0000	454218	144553	129831
C. tibetana	*C. carlesii*	*C. jucunda*	0.0378	0.0136	0.0000	236812	195590	181344
C. tibetana	*C. carlesii*	*C. sclerophylla*	0.0273	0.0110	0.0000	243440	199142	188547
C. tibetana	*C. eyrei*	*C. chinensis*	0.0501	0.0195	0.0000	230084	197842	178958
C. tibetana	*C. eyrei*	*C. fissa*	0.0775	0.0154	0.0000	454249	143453	122827
C. tibetana	*C. eyrei*	*C. jucunda*	0.0551	0.0192	0.0000	235506	193961	173691
C. tibetana	*C. eyrei*	*C. sclerophylla*	0.0439	0.0172	0.0000	242233	197449	180832
C. tibetana	*C. fabri*	*C. fissa*	0.0490	0.0101	0.0000	451615	144789	131253
C. tibetana	*C. fargesii*	*C. fissa*	0.0389	0.0082	0.0000	454500	146178	135222
C. tibetana	*C. lamontii*	*C. chinensis*	0.0318	0.0125	0.0000	228292	196422	184319
C. tibetana	*C. lamontii*	*C. fissa*	0.0643	0.0129	0.0000	450108	143094	125810
C. tibetana	*C. lamontii*	*C. jucunda*	0.0408	0.0144	0.0000	233174	193516	178342
C. hystrix	*C. lamontii*	*C. chinensis*	0.0291	0.0117	0.0001	228665	199060	187791
C. lamontii	*C. carlesii*	*C. hystrix*	0.0187	0.0059	0.0001	455760	119394	115015
C. fargesii	*C. fabri*	*C. fissa*	0.0131	0.0019	0.0002	689499	98961	96396
C. tibetana	*C. jucunda*	*C. fissa*	0.0132	0.0029	0.0002	409132	147630	143797
C. tibetana	*C. lamontii*	*C. sclerophylla*	0.0276	0.0110	0.0002	240050	196446	185886
C. hystrix	*C. lamontii*	*C. sclerophylla*	0.0286	0.0115	0.0003	240914	199473	188383
C. tibetana	*C. chinensis*	*C. fissa*	0.0112	0.0025	0.0012	418910	146378	143137
C. tibetana	*C. fargesii*	*C. jucunda*	0.0219	0.0080	0.0014	238326	196759	188330
C. tibetana	*C. fabri*	*C. jucunda*	0.0254	0.0092	0.0015	235728	194337	184720
C. fabri	*C. carlesii*	*C. fordii*	0.0117	0.0037	0.0059	432220	128646	125671
C. hystrix	*C. fabri*	*C. jucunda*	0.0243	0.0089	0.0060	238224	196373	187050
C. hystrix	*C. fargesii*	*C. jucunda*	0.0210	0.0078	0.0066	240865	198894	190709
C. sclerophylla	*C. jucunda*	*C. lamontii*	0.0106	0.0040	0.0155	531568	122618	120049
C. hystrix	*C. tibetana*	*C. lamontii*	0.0094	0.0041	0.0338	491968	133695	131194
C. tibetana	*C. fabri*	*C. chinensis*	0.0164	0.0066	0.0363	230797	196925	190579
C. tibetana	*C. fargesii*	*C. chinensis*	0.0154	0.0063	0.0379	232895	199845	193802
C. hystrix	*C. fabri*	*C. sclerophylla*	0.0151	0.0062	0.0468	245177	199645	193686
C. fargesii	*C. fabri*	*C. fordii*	0.0089	0.0029	0.0532	433180	133065	130712
C. tibetana	*C. fabri*	*C. sclerophylla*	0.0141	0.0057	0.0545	242274	197417	191936
C. sclerophylla	*C. jucunda*	*C. eyrei*	0.0072	0.0032	0.0695	527458	123036	121266
C. hystrix	*C. fargesii*	*C. sclerophylla*	0.0139	0.0058	0.0697	247776	202824	197257
C. tibetana	*C. fargesii*	*C. sclerophylla*	0.0127	0.0052	0.0730	244823	200535	195516
C. hystrix	*C. fabri*	*C. chinensis*	0.0139	0.0056	0.0797	232995	198494	193059
C. fabri	*C. lamontii*	*C. fordii*	0.0073	0.0023	0.0801	428885	126033	124212
C. hystrix	*C. fargesii*	*C. chinensis*	0.0131	0.0054	0.0801	235348	201712	196509
C. fargesii	*C. lamontii*	*C. hystrix*	0.0089	0.0029	0.0966	458117	122976	120795
C. jucunda	*C. sclerophylla*	*C. tibetana*	0.0080	0.0024	0.1081	546436	127682	125643
C. fabri	*C. carlesii*	*C. fissa*	0.0064	0.0009	0.1149	689575	94496	93289
C. sclerophylla	*C. jucunda*	*C. fabri*	0.0075	0.0026	0.1156	536878	123244	121403
C. sclerophylla	*C. jucunda*	*C. carlesii*	0.0068	0.0023	0.1157	532741	123763	122084
C. carlesii	*C. eyrei*	*C. fabri*	0.0086	0.0067	0.1248	207105	147395	144868
C. lamontii	*C. carlesii*	*C. fabri*	0.0078	0.0063	0.1632	195817	154703	152310
C. hystrix	*C. tibetana*	*C. fissa*	0.0048	0.0007	0.1694	773018	101985	101007
C. chinensis	*C. jucunda*	*C. fissa*	0.0049	0.0007	0.1740	724341	98595	97633
C. fordii	*C. hystrix*	*C. fissa*	0.0034	0.0005	0.1944	829942	98097	97439
C. jucunda	*C. sclerophylla*	*C. fordii*	0.0065	0.0020	0.2319	553900	127197	125551
C. sclerophylla	*C. jucunda*	*C. fargesii*	0.0054	0.0017	0.2319	539605	124772	123436
C. fargesii	*C. fabri*	*C. jucunda*	0.0050	0.0011	0.2525	453286	121902	120700
C. jucunda	*C. sclerophylla*	*C. hystrix*	0.0052	0.0017	0.2753	548453	128821	127476
C. lamontii	*C. carlesii*	*C. fordii*	0.0052	0.0015	0.2964	461595	116488	115284
C. tibetana	*C. hystrix*	*C. carlesii*	0.0045	0.0017	0.2968	490705	135154	133941
C. fabri	*C. lamontii*	*C. hystrix*	0.0045	0.0015	0.3061	424057	126906	125776
C. fordii	*C. sclerophylla*	*C. fissa*	0.0036	0.0008	0.3211	414995	153478	152379
C. fargesii	*C. fabri*	*C. hystrix*	0.0040	0.0015	0.3294	427706	134114	133032
C. fargesii	*C. fabri*	*C. tibetana*	0.0041	0.0013	0.3490	426347	132546	131461
C. carlesii	*C. lamontii*	*C. jucunda*	0.0042	0.0009	0.3508	480267	107448	106556
C. tibetana	*C. hystrix*	*C. chinensis*	0.0038	0.0010	0.3598	528258	132434	131431
C. lamontii	*C. carlesii*	*C. tibetana*	0.0031	0.0009	0.4953	453750	116540	115825
C. fargesii	*C. fabri*	*C. sclerophylla*	0.0021	0.0006	0.5879	461845	125257	124723
C. sclerophylla	*C. tibetana*	*C. fissa*	0.0017	0.0004	0.6496	418500	149757	149239
C. fargesii	*C. fabri*	*C. chinensis*	0.0015	0.0004	0.6817	447360	123949	123590
C. tibetana	*C. hystrix*	*C. fargesii*	0.0015	0.0006	0.6971	498131	136059	135647
C. lamontii	*C. carlesii*	*C. chinensis*	0.0017	0.0004	0.7083	473714	109331	108954
C. hystrix	*C. sclerophylla*	*C. fissa*	0.0012	0.0003	0.7364	421311	152380	152017
C. hystrix	*C. tibetana*	*C. eyrei*	0.0016	0.0008	0.7375	485543	133874	133448
C. tibetana	*C. hystrix*	*C. fabri*	0.0017	0.0007	0.7455	495160	134184	133733
C. tibetana	*C. hystrix*	*C. jucunda*	0.0014	0.0003	0.7767	535964	129169	128808
C. hystrix	*C. tibetana*	*C. sclerophylla*	0.0012	0.0003	0.7941	541870	133563	133249
C. carlesii	*C. lamontii*	*C. sclerophylla*	0.0000	0.0000	0.9947	489301	110150	110144

Table S9 Spearman's correlation values between f_{dM} and each of the other parameters (π, recombination rate, gene density, F_{ST}, and d_{XY}) in 51 tric
The "Trio" column shows the three ingroups in each trio with the order of P1_P2_P3. Correlation analyses were conducted for f_{dM} vs. π and recombination rate for each P1 and P2 species individually. Each row represents a test conducted in 10, 100, or 500 kb windows.
Significant tests ($P < 0.05$) are highlighted in bold.

Trio	π (P2)	π (P3)	Recombination rate (P2)	Recombination rate (P3)	F_{ST}	d_{XY}	Gene density
**10 kb windows**							
C. chinensis_C. sclerophylla_C. jucunda	**0.0867**	**0.0602**	**0.0213**	**-0.0188**	**-0.2428**	**-0.1586**	**-0.0221**
C. fargesii_C. carlesii_C. fabri	**0.3426**	**0.3009**	**0.1806**	**0.1971**	**-0.4516**	**0.0758**	**-0.0857**
C. fargesii_C. carlesii_C. fordii	**0.4098**	**0.3686**	**0.2140**	**0.1749**	**-0.4961**	**0.0340**	**-0.1122**
C. fargesii_C. carlesii_C. hystrix	**0.4240**	**0.3651**	**0.2309**	**0.2323**	**-0.5115**	**0.0158**	**-0.1109**
C. fargesii_C. carlesii_C. lamontii	**0.3149**	**0.2757**	**0.1701**	**0.1934**	**-0.4374**	**0.0691**	**-0.0816**
C. fargesii_C. carlesii_C. tibetana	**0.4157**	**0.3705**	**0.2163**	**0.1973**	**-0.4960**	**0.0237**	**-0.1076**
C. fargesii_C. eyrei_C. fabri	**0.1452**	**0.1306**	**0.0739**	**0.0693**	**-0.2713**	-0.0047	**-0.0395**
C. fargesii_C. eyrei_C. fordii	**0.2927**	**0.2555**	**0.1535**	**0.1074**	**-0.3976**	-0.0096	**-0.0899**
C. fargesii_C. eyrei_C. hystrix	**0.3069**	**0.2662**	**0.1707**	**0.1529**	**-0.4131**	-0.0077	**-0.0921**
C. fargesii_C. eyrei_C. tibetana	**0.3050**	**0.2665**	**0.1625**	**0.1088**	**-0.4028**	-0.0096	**-0.0798**
C. fargesii_C. lamontii_C. fabri	**0.1218**	**0.1102**	**0.0634**	**0.0719**	**-0.2589**	**-0.0385**	**-0.0440**
C. fargesii_C. lamontii_C. fordii	**0.2889**	**0.2607**	**0.1615**	**0.1139**	**-0.3981**	**-0.0298**	**-0.0705**
C. fordii_C. carlesii_C. chinensis	**0.2048**	**0.2164**	**0.1382**	**0.0929**	**-0.3583**	**-0.1424**	**-0.0723**
C. fordii_C. carlesii_C. fissa	**0.2612**	**0.2448**	**0.1451**	**0.0956**	**-0.3364**	**-0.0398**	**-0.0985**
C. fordii_C. carlesii_C. jucunda	**0.1974**	**0.1956**	**0.1230**	**0.0602**	**-0.3319**	**-0.1500**	**-0.0601**
C. fordii_C. carlesii_C. sclerophylla	**0.2045**	**0.2027**	**0.1289**	**0.0825**	**-0.3461**	**-0.1405**	**-0.0685**
C. fordii_C. chinensis_C. fissa	**0.2266**	**0.2189**	**0.1056**	**0.0934**	**-0.2943**	**-0.0594**	**-0.0844**
C. fordii_C. eyrei_C. chinensis	**0.1880**	**0.2022**	**0.1217**	**0.0770**	**-0.3428**	**-0.1083**	**-0.0643**
C. fordii_C. eyrei_C. fissa	**0.2473**	**0.2424**	**0.1498**	**0.0959**	**-0.3321**	**-0.0153**	**-0.1006**
C. fordii_C. eyrei_C. jucunda	**0.1790**	**0.1837**	**0.1070**	**0.0613**	**-0.3207**	**-0.1160**	**-0.0648**
C. fordii_C. eyrei_C. sclerophylla	**0.1822**	**0.1944**	**0.1231**	**0.0840**	**-0.3390**	**-0.1154**	**-0.0630**
C. fordii_C. fabri_C. chinensis	**0.1789**	**0.1862**	**0.1164**	**0.0573**	**-0.3202**	**-0.1274**	**-0.0566**
C. fordii_C. fabri_C. fissa	**0.2615**	**0.2253**	**0.1497**	**0.0889**	**-0.3228**	**-0.0250**	**-0.0983**
C. fordii_C. fabri_C. jucunda	**0.1702**	**0.1674**	**0.1127**	**0.0354**	**-0.2980**	**-0.1299**	**-0.0564**
C. fordii_C. fabri_C. sclerophylla	**0.1708**	**0.1735**	**0.1167**	**0.0727**	**-0.3117**	**-0.1257**	**-0.0526**
C. fordii_C. fargesii_C. chinensis	**0.1968**	**0.1933**	**0.1125**	**0.0765**	**-0.3306**	**-0.1202**	**-0.0624**
C. fordii_C. fargesii_C. fissa	**0.2504**	**0.2320**	**0.1455**	**0.0867**	**-0.3210**	**-0.0258**	**-0.0856**
C. fordii_C. fargesii_C. jucunda	**0.1856**	**0.1733**	**0.1019**	**0.0442**	**-0.3053**	**-0.1309**	**-0.0605**
C. fordii_C. fargesii_C. sclerophylla	**0.1913**	**0.1868**	**0.1052**	**0.0737**	**-0.3257**	**-0.1251**	**-0.0638**
C. fordii_C. hystrix_C. fabri	**0.3338**	**0.3282**	**0.1872**	**0.2197**	**-0.4538**	-0.0020	**-0.0906**
C. fordii_C. hystrix_C. fargesii	**0.3397**	**0.3393**	**0.1895**	**0.2180**	**-0.4624**	0.0014	**-0.0996**
C. fordii_C. hystrix_C. jucunda	**0.3467**	**0.3147**	**0.2168**	**0.1392**	**-0.4535**	**-0.0584**	**-0.1014**
C. fordii_C. hystrix_C. lamontii	**0.3316**	**0.3211**	**0.1895**	**0.2194**	**-0.4530**	-0.0035	**-0.1023**
C. fordii_C. hystrix_C. sclerophylla	**0.3548**	**0.3348**	**0.2199**	**0.1954**	**-0.4707**	**-0.0407**	**-0.1100**
C. fordii_C. hystrix_C. tibetana	**0.1284**	**0.1411**	**0.0712**	**0.0457**	**-0.3043**	**-0.0448**	**-0.0342**
C. fordii_C. lamontii_C. chinensis	**0.1841**	**0.2002**	**0.1235**	**0.0696**	**-0.3327**	**-0.1278**	**-0.0641**
C. fordii_C. lamontii_C. fissa	**0.2575**	**0.2301**	**0.1512**	**0.0848**	**-0.3247**	**-0.0303**	**-0.0984**
C. fordii_C. lamontii_C. jucunda	**0.1786**	**0.1853**	**0.1106**	**0.0572**	**-0.3125**	**-0.1298**	**-0.0665**
C. fordii_C. lamontii_C. sclerophylla	**0.1784**	**0.1866**	**0.1149**	**0.0766**	**-0.3210**	**-0.1206**	**-0.0639**
C. fordii_C. tibetana_C. fabri	**0.3250**	**0.3088**	**0.1470**	**0.1991**	**-0.4408**	**-0.0201**	**-0.0906**
C. fordii_C. tibetana_C. fargesii	**0.3312**	**0.3253**	**0.1446**	**0.1979**	**-0.4554**	**-0.0211**	**-0.0929**
C. fordii_C. tibetana_C. fissa	**0.3061**	**0.2735**	**0.1796**	**0.1348**	**-0.3300**	**0.0698**	**-0.1157**
C. fordii_C. tibetana_C. jucunda	**0.3503**	**0.2999**	**0.1777**	**0.1300**	**-0.4345**	**-0.0528**	**-0.0928**
C. fordii_C. tibetana_C. lamontii	**0.3213**	**0.3066**	**0.1406**	**0.2014**	**-0.4407**	**-0.0264**	**-0.0948**
C. fordii_C. tibetana_C. sclerophylla	**0.3600**	**0.3254**	**0.1766**	**0.1755**	**-0.4594**	**-0.0484**	**-0.1061**
C. jucunda_C. chinensis_C. fordii	**0.2762**	**0.2887**	**0.1199**	**0.1384**	**-0.4140**	**-0.0930**	**-0.0866**
C. jucunda_C. chinensis_C. hystrix	**0.2864**	**0.2733**	**0.1329**	**0.1807**	**-0.4277**	**-0.1102**	**-0.0850**
C. jucunda_C. chinensis_C. tibetana	**0.2865**	**0.2937**	**0.1255**	**0.1489**	**-0.4139**	**-0.0943**	**-0.0715**
C. lamontii_C. eyrei_C. carlesii	**0.1153**	**0.1024**	**0.0933**	**0.0911**	**-0.3388**	**-0.0824**	**-0.0334**
C. lamontii_C. eyrei_C. fargesii	**0.1116**	**0.1015**	**0.0821**	**0.0665**	**-0.3296**	**-0.0813**	**-0.0313**
C. sclerophylla_C. jucunda_C. fissa	**0.2781**	**0.2801**	**0.1555**	**0.1366**	**-0.3299**	**0.0308**	**-0.1129**
**100 kb windows**							
C. chinensis_C. sclerophylla_C. jucunda	**0.1135**	**0.1002**	0.0076	-0.0069	**-0.2270**	-0.1450	**-0.0631**
C. fargesii_C. carlesii_C. fabri	**0.3948**	**0.3515**	**0.1891**	**0.2156**	**-0.4290**	0.1243	**-0.0572**
C. fargesii_C. carlesii_C. fordii	**0.4458**	**0.4047**	**0.2600**	**0.1310**	**-0.4907**	-0.0041	**-0.0994**
C. fargesii_C. carlesii_C. hystrix	**0.4679**	**0.4142**	**0.2593**	**0.2150**	**-0.5102**	-0.0390	**-0.1002**
C. fargesii_C. carlesii_C. lamontii	**0.3692**	**0.3330**	**0.1792**	**0.1842**	**-0.4259**	0.1041	**-0.0933**
C. fargesii_C. carlesii_C. tibetana	**0.4413**	**0.4084**	**0.2326**	**0.1271**	**-0.4875**	-0.0173	**-0.1148**
C. fargesii_C. eyrei_C. fabri	**0.1357**	**0.1194**	**0.0430**	0.0262	**-0.1842**	0.0351	**-0.0189**
C. fargesii_C. eyrei_C. fordii	**0.3071**	**0.2875**	**0.2014**	**0.0667**	**-0.3853**	-0.0370	**-0.0756**
C. fargesii_C. eyrei_C. hystrix	**0.3206**	**0.2845**	**0.2014**	**0.1073**	**-0.3942**	-0.0555	**-0.0716**
C. fargesii_C. eyrei_C. tibetana	**0.3199**	**0.2831**	**0.2102**	**0.0531**	**-0.3851**	-0.0485	**-0.0756**
C. fargesii_C. lamontii_C. fabri	**0.0836**	**0.0902**	0.0099	0.0338	**-0.1698**	-0.0280	-0.0275

C. fargesii_C. lamontii_C. fordii	**0.2935**	**0.2601**	**0.1596**	**0.0662**	**-0.3673**	**-0.0740**	**-0.0457**
C. fordii_C. carlesii_C. chinensis	**0.2474**	**0.2159**	**0.1449**	**0.0829**	**-0.3177**	**-0.1444**	**-0.0874**
C. fordii_C. carlesii_C. fissa	**0.2927**	**0.2692**	**0.1938**	0.0139	**-0.3609**	**-0.1279**	**-0.0984**
C. fordii_C. carlesii_C. jucunda	**0.2369**	**0.2162**	**0.1395**	**0.0409**	**-0.3103**	**-0.1672**	**-0.0871**
C. fordii_C. carlesii_C. sclerophylla	**0.2270**	**0.2254**	**0.1242**	**0.0615**	**-0.3095**	**-0.1445**	**-0.0922**
C. fordii_C. chinensis_C. fissa	**0.2447**	**0.2197**	**0.1024**	0.0150	**-0.2816**	**-0.1133**	**-0.0900**
C. fordii_C. eyrei_C. chinensis	**0.2352**	**0.2368**	**0.1492**	**0.0784**	**-0.3169**	**-0.0930**	**-0.0945**
C. fordii_C. eyrei_C. fissa	**0.2598**	**0.2533**	**0.1609**	**0.0470**	**-0.3303**	**-0.0672**	**-0.1109**
C. fordii_C. eyrei_C. jucunda	**0.2210**	**0.2196**	**0.1457**	0.0213	**-0.3013**	**-0.1159**	**-0.0848**
C. fordii_C. eyrei_C. sclerophylla	**0.2206**	**0.2238**	**0.1346**	**0.0535**	**-0.3062**	**-0.1031**	**-0.0944**
C. fordii_C. fabri_C. chinensis	**0.2054**	**0.2074**	**0.1273**	**0.0679**	**-0.2836**	**-0.1143**	**-0.0866**
C. fordii_C. fabri_C. fissa	**0.2999**	**0.2577**	**0.1897**	0.0354	**-0.3585**	**-0.0988**	**-0.0880**
C. fordii_C. fabri_C. jucunda	**0.1740**	**0.1773**	**0.0958**	-0.0115	**-0.2560**	**-0.1399**	**-0.0624**
C. fordii_C. fabri_C. sclerophylla	**0.1896**	**0.1809**	**0.1095**	**0.0442**	**-0.2669**	**-0.1108**	**-0.0862**
C. fordii_C. fargesii_C. chinensis	**0.2397**	**0.2208**	**0.1175**	**0.0737**	**-0.3086**	**-0.1117**	**-0.0742**
C. fordii_C. fargesii_C. fissa	**0.2750**	**0.2515**	**0.1413**	0.0180	**-0.3318**	**-0.0960**	**-0.1006**
C. fordii_C. fargesii_C. jucunda	**0.2279**	**0.2082**	**0.1121**	0.0365	**-0.2965**	**-0.1317**	**-0.0804**
C. fordii_C. fargesii_C. sclerophylla	**0.2144**	**0.2198**	**0.1135**	**0.0557**	**-0.3015**	**-0.1387**	**-0.0901**
C. fordii_C. hystrix_C. fabri	**0.3812**	**0.3638**	**0.1712**	**0.2555**	**-0.4630**	**-0.0608**	**-0.1099**
C. fordii_C. hystrix_C. fargesii	**0.4025**	**0.4033**	**0.1904**	**0.2430**	**-0.4974**	**-0.0700**	**-0.0978**
C. fordii_C. hystrix_C. jucunda	**0.3772**	**0.3722**	**0.1867**	**0.0659**	**-0.4671**	**-0.1615**	**-0.1025**
C. fordii_C. hystrix_C. lamontii	**0.3887**	**0.3667**	**0.1699**	**0.2459**	**-0.4681**	**-0.0486**	**-0.0783**
C. fordii_C. hystrix_C. sclerophylla	**0.3986**	**0.3938**	**0.2139**	**0.1391**	**-0.4883**	**-0.1343**	**-0.0958**
C. fordii_C. hystrix_C. tibetana	**0.1745**	**0.1918**	**0.0830**	**0.0368**	**-0.2799**	0.0144	**-0.0778**
C. fordii_C. lamontii_C. chinensis	**0.1915**	**0.2001**	**0.1123**	**0.0553**	**-0.2820**	**-0.1358**	**-0.0824**
C. fordii_C. lamontii_C. fissa	**0.2920**	**0.2698**	**0.1756**	**0.0459**	**-0.3537**	**-0.0949**	**-0.0842**
C. fordii_C. lamontii_C. jucunda	**0.1960**	**0.2001**	**0.1001**	0.0036	**-0.2772**	**-0.1444**	**-0.0825**
C. fordii_C. lamontii_C. sclerophylla	**0.1972**	**0.2028**	**0.1073**	**0.0496**	**-0.2845**	**-0.1323**	**-0.0858**
C. fordii_C. tibetana_C. fabri	**0.3613**	**0.3426**	**0.0844**	**0.2282**	**-0.4366**	**-0.0602**	**-0.0959**
C. fordii_C. tibetana_C. fargesii	**0.3433**	**0.3534**	**0.0786**	**0.2002**	**-0.4375**	**-0.0898**	**-0.0711**
C. fordii_C. tibetana_C. fissa	**0.2882**	**0.2718**	**0.1067**	**0.0588**	**-0.3149**	-0.0034	**-0.1201**
C. fordii_C. tibetana_C. jucunda	**0.3789**	**0.3471**	**0.1076**	**0.0744**	**-0.4415**	**-0.1481**	**-0.0728**
C. fordii_C. tibetana_C. lamontii	**0.3649**	**0.3548**	**0.0693**	**0.2310**	**-0.4488**	**-0.0631**	**-0.0814**
C. fordii_C. tibetana_C. sclerophylla	**0.3836**	**0.3483**	**0.0859**	**0.1341**	**-0.4530**	**-0.1339**	**-0.0699**
C. jucunda_C. chinensis_C. fordii	**0.3015**	**0.2914**	**0.0689**	**0.0708**	**-0.3910**	**-0.1740**	**-0.0833**
C. jucunda_C. chinensis_C. hystrix	**0.3235**	**0.3154**	**0.0783**	**0.1617**	**-0.4441**	**-0.2207**	**-0.0897**
C. jucunda_C. chinensis_C. tibetana	**0.3091**	**0.3164**	**0.0910**	**0.0805**	**-0.4036**	**-0.1771**	**-0.1026**
C. lamontii_C. eyrei_C. carlesii	**0.1811**	**0.1642**	**0.1359**	**0.1271**	**-0.3192**	**-0.0419**	**-0.0818**
C. lamontii_C. eyrei_C. fargesii	**0.1576**	**0.1792**	**0.1285**	**0.0967**	**-0.3113**	**-0.0420**	**-0.0640**
C. sclerophylla_C. jucunda_C. fissa	**0.2528**	**0.2455**	**0.0782**	**0.0418**	**-0.2853**	**-0.0699**	**-0.0854**
**500 kb windows**							
C. chinensis_C. sclerophylla_C. jucunda	0.0114	**0.0220**	0.0044	0.0423	**-0.0831**	**-0.1405**	**-0.0911**
C. fargesii_C. carlesii_C. fabri	**0.3372**	**0.3155**	**0.2146**	**0.2316**	**-0.3545**	**0.1088**	**-0.1031**
C. fargesii_C. carlesii_C. fordii	**0.4725**	**0.4503**	**0.3620**	**0.2087**	**-0.4947**	**-0.0652**	**-0.1050**
C. fargesii_C. carlesii_C. hystrix	**0.5150**	**0.4936**	**0.4076**	**0.3497**	**-0.5514**	**-0.1330**	**-0.0871**
C. fargesii_C. carlesii_C. lamontii	**0.4054**	**0.3833**	**0.2405**	**0.2468**	**-0.4183**	**0.1965**	**-0.1538**
C. fargesii_C. carlesii_C. tibetana	**0.4563**	**0.4420**	**0.3500**	**0.2083**	**-0.4865**	**-0.0820**	**-0.1125**
C. fargesii_C. eyrei_C. fabri	**0.0584**	**0.0534**	-0.0215	-0.0187	**-0.0901**	0.0020	**-0.0982**
C. fargesii_C. eyrei_C. fordii	**0.3155**	**0.3109**	**0.2262**	**0.1073**	**-0.3607**	-0.0358	**-0.1494**
C. fargesii_C. eyrei_C. hystrix	**0.3602**	**0.3716**	**0.2564**	**0.2275**	**-0.3931**	-0.0025	**-0.1130**
C. fargesii_C. eyrei_C. tibetana	**0.3523**	**0.3200**	**0.2779**	**0.1398**	**-0.3747**	-0.0184	**-0.0960**
C. fargesii_C. lamontii_C. fabri	0.0283	0.0125	-0.0158	-0.0030	-0.0454	-0.0212	-0.0771
C. fargesii_C. lamontii_C. fordii	**0.2760**	**0.2712**	**0.1757**	**0.0952**	**-0.3145**	-0.0540	**-0.1021**
C. fordii_C. carlesii_C. chinensis	**0.1875**	**0.1919**	**0.1322**	**0.0928**	**-0.2219**	**-0.1359**	**-0.0981**
C. fordii_C. carlesii_C. fissa	**0.3584**	**0.3373**	**0.2457**	**0.0762**	**-0.3878**	**-0.1647**	**-0.0965**
C. fordii_C. carlesii_C. jucunda	**0.1671**	**0.1575**	**0.1135**	0.0592	**-0.1960**	**-0.1316**	**-0.1247**
C. fordii_C. carlesii_C. sclerophylla	**0.1676**	**0.1873**	**0.1277**	0.0135	**-0.2153**	**-0.1346**	**-0.0956**
C. fordii_C. chinensis_C. fissa	**0.2993**	**0.2859**	**0.1817**	0.0413	**-0.3230**	**-0.1506**	**-0.1147**
C. fordii_C. eyrei_C. chinensis	**0.1621**	**0.1759**	**0.1185**	**0.0839**	**-0.2037**	**-0.0773**	**-0.1267**
C. fordii_C. eyrei_C. fissa	**0.3219**	**0.3397**	**0.2652**	0.0564	**-0.3790**	**-0.0990**	**-0.0966**
C. fordii_C. eyrei_C. jucunda	**0.1315**	**0.1566**	**0.0994**	0.0646	**-0.1831**	**-0.0953**	**-0.1214**
C. fordii_C. eyrei_C. sclerophylla	**0.1807**	**0.2129**	**0.1423**	0.0403	**-0.2411**	**-0.0899**	**-0.1281**
C. fordii_C. fabri_C. chinensis	**0.1459**	**0.1599**	**0.1040**	0.0428	**-0.1878**	**-0.1076**	-0.0642
C. fordii_C. fabri_C. fissa	**0.3651**	**0.3433**	**0.2264**	0.0516	**-0.3972**	**-0.1277**	**-0.1081**
C. fordii_C. fabri_C. jucunda	**0.0854**	**0.1038**	**0.0707**	0.0408	**-0.1356**	**-0.1213**	**-0.1080**
C. fordii_C. fabri_C. sclerophylla	**0.1810**	**0.1944**	**0.1343**	**0.0755**	**-0.2250**	**-0.1210**	**-0.1007**
C. fordii_C. fargesii_C. chinensis	**0.1452**	**0.1425**	**0.0907**	**0.0876**	**-0.1813**	**-0.1260**	-0.0730
C. fordii_C. fargesii_C. fissa	**0.3320**	**0.3129**	**0.2069**	0.0476	**-0.3609**	**-0.1092**	-0.0710
C. fordii_C. fargesii_C. jucunda	**0.1720**	**0.1597**	**0.1023**	0.0628	**-0.2034**	**-0.1225**	**-0.1396**
C. fordii_C. fargesii_C. sclerophylla	**0.1960**	**0.1875**	**0.0911**	0.0387	**-0.2328**	**-0.0991**	**-0.1030**
C. fordii_C. hystrix_C. fabri	**0.4277**	**0.4366**	**0.2538**	**0.3293**	**-0.4695**	-0.0672	**-0.1506**

C. fordii_C. hystrix_C. fargesii	**0.4615**	**0.4632**	**0.2780**	**0.3352**	**-0.5047**	**-0.0845**	**-0.2067**
C. fordii_C. hystrix_C. jucunda	**0.4127**	**0.4047**	**0.2686**	**0.0823**	**-0.4598**	**-0.2111**	**-0.1546**
C. fordii_C. hystrix_C. lamontii	**0.4632**	**0.4816**	**0.2637**	**0.3223**	**-0.5109**	-0.0412	**-0.1190**
C. fordii_C. hystrix_C. sclerophylla	**0.4799**	**0.4860**	**0.3151**	**0.1708**	**-0.5261**	**-0.1686**	**-0.1598**
C. fordii_C. hystrix_C. tibetana	**0.2461**	**0.2500**	**0.1778**	**0.0959**	**-0.2948**	**0.1162**	**-0.0768**
C. fordii_C. lamontii_C. chinensis	**0.2044**	**0.2175**	**0.0649**	**0.0962**	**-0.2383**	**-0.0823**	**-0.0968**
C. fordii_C. lamontii_C. fissa	**0.3402**	**0.3225**	**0.2246**	-0.0044	**-0.3846**	**-0.1695**	-0.0532
C. fordii_C. lamontii_C. jucunda	**0.1738**	**0.1370**	0.0447	0.0372	**-0.1744**	-0.0581	**-0.0930**
C. fordii_C. lamontii_C. sclerophylla	**0.1720**	**0.1848**	**0.0822**	0.0560	**-0.2169**	**-0.1007**	-0.0550
C. fordii_C. tibetana_C. fabri	**0.4047**	**0.4145**	**0.0822**	**0.3104**	**-0.4531**	-0.0584	-0.0685
C. fordii_C. tibetana_C. fargesii	**0.4264**	**0.4361**	**0.1170**	**0.2652**	**-0.4802**	**-0.0979**	**-0.1139**
C. fordii_C. tibetana_C. fissa	**0.2720**	**0.2768**	**0.1481**	**0.0981**	**-0.2930**	-0.0512	-0.0718
C. fordii_C. tibetana_C. jucunda	**0.3825**	**0.3827**	**0.1020**	**0.1306**	**-0.4222**	**-0.1583**	**-0.1293**
C. fordii_C. tibetana_C. lamontii	**0.4093**	**0.4147**	**0.1124**	**0.2973**	**-0.4538**	-0.0318	-0.0544
C. fordii_C. tibetana_C. sclerophylla	**0.4245**	**0.4250**	**0.1381**	**0.1905**	**-0.4682**	**-0.1675**	**-0.1018**
C. jucunda_C. chinensis_C. fordii	**0.3364**	**0.3402**	**0.1527**	**0.1285**	**-0.4006**	**-0.1986**	**-0.1384**
C. jucunda_C. chinensis_C. hystrix	**0.3829**	**0.3760**	**0.1486**	**0.2257**	**-0.4425**	**-0.2188**	**-0.1346**
C. jucunda_C. chinensis_C. tibetana	**0.3103**	**0.3321**	**0.1098**	0.0323	**-0.3742**	**-0.1957**	**-0.1184**
C. lamontii_C. eyrei_C. carlesii	**0.2788**	**0.2848**	**0.2605**	**0.2322**	**-0.3853**	0.0398	**-0.1413**
C. lamontii_C. eyrei_C. fargesii	**0.2506**	**0.2702**	**0.2416**	**0.2149**	**-0.3529**	0.0492	**-0.1166**
C. sclerophylla_C. jucunda_C. fissa	**0.3116**	**0.2893**	0.0514	**0.0877**	**-0.3270**	**-0.1267**	**-0.1243**

Table S10 Characteristics of potential introgression regions across the 51 trios. The "Trio" column displays the three ingroups in each trio with the order of P1_P2_P3. The columns "N_e (P2)" and "N_e (P3)" show the effective population size of P2 and P3 species, respectively. The "f_4-ratio" column illustrates the admixture proportion between each pair of species linked by gene flow. Estimates for each statistic, including π, recombination rate, gene density, F_{ST}, d_{XY}, |nSL|, H12, additive load, and recessive load, were calculated in introgression regions relative to the genomic background. Estimates specific to P2 and P3 species are indicated in parentheses after the respective values.

Trio[a]	P2	P3	N_e (P2)[b]	N_e (P3)[b]	No. of 100 kb windows	f_4-ratio	π (P2)[c]	π (P3)[c]	Recombination rate (P2)[c]	Recombination rate (P3)[c]	Gene density[c]	F_{ST}[c]	d_{XY}[c]	|nSL| (P2)[c]	|nSL| (P3)[c]	H12 (P2)[c]	H12 (P3)[c]	Additive load (P2)[c]	Additive load (P3)[c]	Recessive load (P2)[c]	Recessive load (P3)[c]
(C. fordii, C. carlesii), C. fissa	*C. carlesii*	*C. fissa*	2.28E+04	1.55E+04	71	0.0123	1.1610**	1.4287**	0.9155	1.0772	0.9627	0.6744**	0.8468**	1.1044**	1.1002*	1.4181**	1.2248*	1.2089**	0.9527	1.2824**	1.1406*
(C. fordii, C. carlesii), C. jucunda	*C. carlesii*	*C. jucunda*	2.28E+04	1.43E+04	120	0.0205	1.1899**	1.3485**	1.2785*	1.2185	0.8978	0.6124**	0.8480**	1.0393	1.0902*	1.0799*	1.1078**	1.0096	1.1474**	1.1364	0.7576**
(C. fordii, C. eyrei), C. fissa	*C. eyrei*	*C. fissa*	2.50E+04	1.55E+04	96	0.0166	1.1490**	1.3601**	1.1340	1.1771	0.9607	0.6356**	0.8881**	1.1426**	1.0730*	1.2367**	1.0524*	0.9296**	0.8505**	0.9648	0.6882**
(C. fordii, C. eyrei), C. jucunda	*C. eyrei*	*C. jucunda*	2.50E+04	1.43E+04	154	0.0261	1.1613**	1.4351**	1.4694**	1.1587	0.9055	0.7389**	0.8765**	1.0497*	1.0698*	1.1504**	1.2074**	0.9204	1.0021	0.9010*	1.0225
(C. fordii, C. fabri), C. fissa	*C. fabri*	*C. fissa*	2.44E+04	1.55E+04	67	0.0114	1.2022**	1.3188**	1.1144	1.0405	0.9118	0.609**	1.0652**	1.0922**	1.0611**	1.0618*	1.0611*	0.9732	1.0965*	0.8956*	0.9638
(C. fordii, C. fabri), C. jucunda	*C. fabri*	*C. jucunda*	2.44E+04	1.43E+04	94	0.0161	1.2052**	1.4271**	1.4542**	1.0727*	0.8451*	0.6459**	0.9328**	1.0332**	1.0600**	0.9928*	1.0218**	1.1796**	1.0528	1.0618	0.9298
(C. fordii, C. fargesii), C. fissa	*C. fargesii*	*C. fissa*	2.44E+04	1.55E+04	55	0.0094	1.1895**	1.3722**	0.8545	1.0475	1.0176	0.6724**	0.9054**	1.0697*	1.0571**	1.0917*	1.0261**	1.0761**	1.0359*	1.0409	1.0403
(C. fordii, C. fargesii), C. jucunda	*C. fargesii*	*C. jucunda*	2.44E+04	1.43E+04	88	0.0149	1.1880**	1.3291**	1.2754*	1.1496	0.8841	0.5971**	0.8925**	1.1125**	1.0555*	1.1867**	1.1015**	1.1665**	1.2776**	1.1699**	1.1070
(C. fordii, C. hystrix), C. jucunda	*C. hystrix*	*C. jucunda*	2.80E+04	1.43E+04	42	0.0071	1.3006**	1.5603**	1.2608	0.9457	0.9194	0.7032**	0.8843**	1.0668**	1.0516**	1.1466**	1.0885**	1.0507	1.1550**	1.1906**	1.165**
(C. fordii, C. lamontii), C. fissa	*C. lamontii*	*C. fissa*	2.19E+04	1.55E+04	81	0.0141	1.2039**	1.3695**	1.3211*	1.1431	0.9941	0.7213**	0.9284**	1.0499**	1.0375**	1.0217*	1.0428**	1.3431**	1.1724**	1.3203**	1.0585
(C. fordii, C. lamontii), C. jucunda	*C. lamontii*	*C. jucunda*	2.19E+04	1.43E+04	124	0.0213	1.2058**	1.3738**	1.2890*	0.9636	0.9314	0.6733**	0.8601**	1.0956**	1.0362*	1.0255**	1.0295**	1.1658**	1.1888**	1.1201*	1.1336*
(C. chinensis, C. sclerophylla), C. jucunda	*C. sclerophylla*	*C. jucunda*	2.19E+04	1.43E+04	371	0.0639	1.0249*	1.0137	1.0367	1.0399	1.0235	0.8142**	0.9615	1.0453	1.0321	1.1333**	0.9963	1.0539	0.8487**	0.5179**	0.5457**
(C. fordii, C. tibetana), C. fissa	*C. tibetana*	*C. fissa*	2.13E+04	1.55E+04	8	0.0013	1.3719*	1.4728*	0.4130	0.8018	0.7098	0.5807**	1.0159	1.0727**	1.0125	1.0727*	0.9911	0.8463**	0.9329	0.6675**	0.6984**
(C. fordii, C. tibetana), C. jucunda	*C. tibetana*	*C. jucunda*	2.13E+04	1.43E+04	41	0.0070	1.3898**	1.4284**	0.7181	1.4457	0.9194	0.7812**	0.8618**	1.0223	1.0060	1.0385*	1.0442**	1.3553**	1.205**	1.3972**	1.2772**
(C. fordii, C. carlesii), C. chinensis	*C. carlesii*	*C. chinensis*	2.28E+04	1.98E+04	126	0.0213	1.2643**	1.4714**	1.478**	1.2799**	0.9705	0.6308**	0.8613**	1.0210	1.1553**	1.0355	1.3020**	1.0074	1.1495**	0.7522**	0.8484**
(C. fargesii, C. carlesii), C. fabri	*C. carlesii*	*C. fabri*	2.28E+04	2.44E+04	102	0.0165	1.3574**	1.3323**	1.5000**	1.4664**	0.8919	0.8646**	0.8142**	1.0269	1.1054	1.0033	1.4890**	1.0884	0.6105**	1.2297	0.8104**
(C. fargesii, C. carlesii), C. fordii	*C. carlesii*	*C. fordii*	2.28E+04	2.38E+04	40	0.0065	1.6134**	1.5057**	1.7508*	1.3659**	0.8428	0.8058**	0.9175*	1.0748**	1.0986**	1.1348**	1.1310**	0.9821	0.8840**	1.1287	0.9464
(C. fargesii, C. carlesii), C. hystrix	*C. carlesii*	*C. hystrix*	2.28E+04	2.80E+04	53	0.0086	1.5957**	1.4368**	1.744**	1.6468**	0.8762	0.5637**	1.1057**	1.0758**	1.0902**	1.0545*	1.1053**	1.0109	1.2181**	1.0024	1.0720
(C. fargesii, C. carlesii), C. lamontii	*C. carlesii*	*C. lamontii*	2.28E+04	2.19E+04	118	0.0191	1.3843**	1.4004**	1.5593**	1.6381**	0.8549*	0.8619**	0.8791**	1.0392**	1.0855**	1.1144**	1.1605**	1.1858**	1.2469**	1.2530**	1.2596**
(C. fordii, C. carlesii), C. sclerophylla	*C. carlesii*	*C. sclerophylla*	2.28E+04	2.19E+04	114	0.0192	1.2432**	1.3234**	1.3624	1.3151*	0.8723*	0.6903**	0.9646	1.0426**	1.0847**	1.0000	1.1684**	1.4918**	1.2143**	1.7082**	1.2118**
(C. fargesii, C. carlesii), C. tibetana	*C. carlesii*	*C. tibetana*	2.28E+04	2.13E+04	42	0.0069	1.4798**	1.5074**	1.3254	1.5927**	0.8255	0.7495**	0.9048**	1.0671**	1.0832*	1.0873**	1.0401*	0.9199*	0.8993**	0.9744	0.8415*
(C. fordii, C. chinensis), C. fissa	*C. chinensis*	*C. fissa*	1.98E+04	1.55E+04	20	0.0035	1.1830	1.0610	1.0000	1.1901	0.9725	0.7514**	0.9149**	1.0385*	1.0824**	1.0072	1.1419**	1.2094**	1.1960**	1.1367*	1.1396**
(C. jucunda, C. chinensis), C. fordii	*C. chinensis*	*C. fordii*	1.98E+04	2.38E+04	61	0.0104	1.1685*	1.1712**	1.2900	0.9480	1.0511	0.6843**	0.9938	1.0264	1.0817**	1.0355*	1.1100**	1.0622	1.1320**	1.0178	1.0195
(C. jucunda, C. chinensis), C. hystrix	*C. chinensis*	*C. hystrix*	1.98E+04	2.80E+04	77	0.0132	1.2784**	1.2332**	1.1178	1.2077	1.0608	0.7358**	0.9434**	1.0640**	1.0807**	1.0576**	1.0861**	1.2111**	0.9226**	1.4633**	0.8216**
(C. jucunda, C. chinensis), C. tibetana	*C. chinensis*	*C. tibetana*	1.98E+04	2.13E+04	67	0.0115	1.2350**	1.2695**	1.2688*	1.0712	1.0569	0.7126**	0.8914**	1.0411*	1.0765**	1.0936**	1.0318**	0.9931	1.0091	0.9183*	0.9627
(C. lamontii, C. eyrei), C. carlesii	*C. eyrei*	*C. carlesii*	2.50E+04	2.28E+04	346	0.0568	1.0754**	1.1040**	1.3057**	1.3051**	1.0491	0.6017**	0.9339**	1.0456	1.0759**	1.0799	1.0131	0.9949	1.0564	0.7307**	0.9421
(C. fordii, C. eyrei), C. chinensis	*C. eyrei*	*C. chinensis*	2.50E+04	1.98E+04	165	0.0277	1.1759**	1.4222**	1.4124**	1.3325**	0.9194	0.6545**	0.8738**	1.0920**	1.0752*	1.0145**	1.0351**	0.9662	1.2046**	0.7961**	1.0539
(C. fargesii, C. eyrei), C. fabri	*C. eyrei*	*C. fabri*	2.50E+04	2.44E+04	145	0.0235	1.0360	1.0462	0.9271	0.9130	0.9902	0.7628**	0.8785**	1.0812**	1.0747*	1.0436**	1.1313**	1.0250	1.1832**	0.9066	1.2287**
(C. lamontii, C. eyrei), C. fargesii	*C. eyrei*	*C. fargesii*	2.50E+04	2.44E+04	219	0.0356	1.0518**	1.1113**	1.1701*	1.2519**	1.0177	0.6964**	0.8939**	1.0832**	1.0737*	1.1561**	1.0235	1.0903**	1.1301**	1.0313	0.9415
(C. fargesii, C. eyrei), C. fordii	*C. eyrei*	*C. fordii*	2.50E+04	2.38E+04	82	0.0133	1.2382**	1.2351**	1.4564*	1.0896	1.0314	0.7378**	0.8437**	1.0617*	1.0719**	1.1124**	1.0769*	0.8487**	1.1785**	0.9703	1.5349**
(C. fargesii, C. eyrei), C. hystrix	*C. eyrei*	*C. hystrix*	2.50E+04	2.80E+04	93	0.0152	1.3493**	1.2798**	1.6719**	1.3927**	0.9450	0.7760**	0.8993**	1.0622**	1.0707	1.0821**	1.1711	0.9857	1.2458**	0.9071**	1.3924**
(C. fordii, C. eyrei), C. sclerophylla	*C. eyrei*	*C. sclerophylla*	2.50E+04	2.19E+04	151	0.0253	1.1773**	1.3434**	1.3858**	1.2730**	0.9075	0.5940**	0.8818**	1.1617**	1.0665**	1.3244**	1.1301**	1.1779**	1.2293**	1.1729	1.0313
(C. fargesii, C. eyrei), C. tibetana	*C. eyrei*	*C. tibetana*	2.50E+04	2.13E+04	92	0.0150	1.3007**	1.3608**	1.4663**	1.3373	0.9391	0.7572**	0.9136**	1.0624*	1.0618	1.0873*	1.1338**	1.0849**	1.2293**	0.9226	1.3401**
(C .fordii, C. fabri), C. chinensis	*C. fabri*	*C. chinensis*	2.44E+04	1.98E+04	89	0.0150	1.1856**	1.3389**	1.2517	1.2864**	0.9549	0.6600**	0.9798	1.0595**	1.0616**	1.0072	1.0266**	1.4691**	1.1775**	1.5175**	1.0737
(C. fordii, C. fabri), C. sclerophylla	*C. fabri*	*C. sclerophylla*	2.44E+04	2.19E+04	82	0.0139	1.3068**	1.3788**	1.4058**	1.4080**	0.9608	0.6305**	0.9140**	1.0448**	1.059**	1.0144*	1.0892**	1.1810**	1.1240**	1.0193	0.8717**
(C. fordii, C. fargesii), C. chinensis	*C. fargesii*	*C. chinensis*	2.44E+04	1.98E+04	88	0.0146	1.2580**	1.3786**	1.1600	1.2789*	0.9037	0.8635**	0.9742	1.0559**	1.0572**	1.0683**	1.1048**	1.1530**	1.0331	1.2355	0.9722
(C. fordii, C. fargesii), C. sclerophylla	*C. fargesii*	*C. sclerophylla*	2.44E+04	2.19E+04	80	0.0134	1.2662**	1.2584**	1.361*	1.5302*	0.8880	0.8287**	0.9099**	1.0102	1.0546**	1.0360	1.0478**	0.9524	0.8470**	1.0618	0.8132**
(C. fordii, C. hystrix), C. fabri	*C. hystrix*	*C. fabri*	2.80E+04	2.44E+04	60	0.0100	1.3490**	1.3251**	1.2217*	1.2815**	0.9784	0.8094**	0.8733**	1.0552**	1.0545	1.0667**	1.0809**	1.1748**	1.1771**	1.5516**	1.3470**
(C. fordii, C. hystrix), C. fargesii	*C. hystrix*	*C. fargesii*	2.80E+04	2.44E+04	65	0.0107	1.3715**	1.4457**	1.2562**	1.2279**	1.0314	0.6289**	0.9772	1.1582**	1.0537	1.1636**	1.0604	1.1658**	1.1523**	1.0121	0.7706**
(C. fordii, C. hystrix), C. lamontii	*C. hystrix*	*C. lamontii*	2.80E+04	2.19E+04	59	0.0097	1.3326**	1.3349**	1.2104*	1.2605*	1.0665	0.8019**	0.8658**	1.0469*	1.0476*	1.1086**	1.1066**	1.0356	0.9054**	1.0272	0.8559**
(C. fordii, C. hystrix), C. sclerophylla	*C. hystrix*	*C. sclerophylla*	2.80E+04	2.19E+04	46	0.0077	1.2601**	1.3416**	1.3536**	1.0733	0.9705	0.6078**	0.8576**	1.0934**	1.0457*	1.3512**	1.0590*	1.1103**	1.2927**	0.8102**	1.0588
(C. fordii, C. hystrix), C. tibetana	*C. hystrix*	*C. tibetana*	2.80E+04	2.13E+04	171	0.0284	1.1357**	1.2811**	1.2628**	1.2094*	0.9902	0.7409**	0.8491**	1.0032	1.0432*	1.0217*	1.1367**	1.3321**	1.0380	1.5012**	0.7719**
(C. sclerophylla, C. jucunda), C. fissa	*C. jucunda*	*C. fissa*	1.43E+04	1.55E+04	17	0.0029	1.6684**	1.5378**	1.0870	1.3937	0.8255	0.5661**	0.9449**	1.0575*	1.0418*	1.0531*	1.0632*	1.1204*	1.2747**	0.8927*	1.0264
((C. fordii, C. lamontii), C. chinensis	*C. lamontii*	*C. chinensis*	2.19E+04	1.98E+04	123	0.0208	1.2170**	1.3620**	1.3194*	1.1461*	0.9550	0.7005**	0.9120**	1.0460**	1.0409	1.0072	1.0201*	1.2584**	1.1319**	1.1077	0.9581
(C. fargesii, C. lamontii), C. fabri	*C. lamontii*	*C. fabri*	2.19E+04	2.44E+04	64	0.0105	0.9942	1.0463	0.7546	0.9172	0.9061	0.5930**	0.9273**	1.0568*	1.0406*	1.0678**	1.0865**	0.9927	1.0915	0.9123	1.0124
(C. fargesii, C. lamontii), C. fordii	*C. lamontii*	*C. fordii*	2.19E+04	2.38E+04	31	0.0051	1.1135*	1.1514*	0.5991	0.9677	1.0196	0.7122**	0.8901**	1.0478*	1.0368	1.0784**	1.0918**	1.1898**	1.0509	1.1032**	0.9067
(C. fordii, C. lamontii), C. sclerophylla	*C. lamontii*	*C. sclerophylla*	2.19E+04	2.19E+04	113	0.0191	1.2200**	1.2867**	1.1507	1.101	0.9137	0.8283**	0.9150**	1.0647**	1.0332	1.1179**	1.0662*	0.9105**	1.0778**	0.7915**	1.1317**
(C. fordii, C. tibetana), C. fabri	*C. tibetana*	*C. fabri*	2.13E+04	2.44E+04	57	0.0094	1.4124**	1.2528**	0.8418	0.9112	1.0274	0.8101**	0.8800**	1.0542*	1.0229	1.0072*	1.0478**	1.1059*	1.0390	1.3913**	1.1995**
(C. fordii, C. tibetana), C. fargesii	*C. tibetana*	*C. fargesii*	2.13E+04	2.44E+04	62	0.0102	1.4382**	1.3303**	0.7493	0.989	0.8863	0.6680**	0.8530**	1.0571*	1.0135	1.0301**	0.9971	1.4306**	1.1657**	1.3464**	1.1857*
(C. fordii, C. tibetana), C. lamontii	*C. tibetana*	*C. lamontii*	2.13E+04	2.19E+04	82	0.0137	1.4554**	1.3064**	0.9015	1.2903**	0.9511	0.6048**	1.0261	1.0960**	0.9985	1.1159*	1.0089	1.2542**	1.1030	1.1820	0.7615**
(C. fordii, C.tibetana), C. sclerophylla	*C. tibetana*	*C. sclerophylla*	2.13E+04	2.19E+04	48	0.0081	1.4501**	1.4175**	0.8254	0.9832	0.8310	0.7660**	0.8898	1.1287	0.9284	1.0432	1.0294	1.2950**	3.1015**	0.6816**	1.5193**

[a]The first 14 trios, which exhibit an effective population size for P2 that is twice as large as that of P3, are highlighted in bold.

[b]The ffection population size (N_e) was estimated using the formula: $N_e = \boldsymbol{\pi}/4\boldsymbol{\mu}$, where $\boldsymbol{\pi}$ is the nucleotide diversity and $\boldsymbol{\mu}$ is the mutation rate (8.04×10^{-8} per site per generation).

[c]Estimates of each statistic in introgression regions relative to the genomic background, with asterisks indicating significance ($*P < 0.05$, $**P < 0.01$) assessed by Wilcoxon-Mann Whitney U-test.

Table S11 Enrichment of resistance gene analogs (RGAs) in introgression regions across 51 trios. The enrichment analyses were conducted for all RGAs collectively, as well as for specific RGA types, which include NBS-LRR (Nucleotide-Binding Leucine Repeat Sequence) genes, RLP (Receptor-Like Proteins), RLK (Receptor-Like Kinases) and TM-CC (Transmembrane-Coiled-Coil) genes.

	All RGAs						NBS-encoding						RLP						RLK						TM-CC					
Trio	No. of genes in introgression regions	No. of genes in non-introgression regions	No. of RGA genes in introgression regions	No. of RGA genes in non-introgression regions	Enrichment of RGA genes*	*P*-value in the hypergeometric test**	No. of genes in introgression regions	No. of genes in non-introgression regions	No. of RGA genes in introgression regions	No. of RGA genes in non-introgression regions	Enrichment of NBS-encoding genes*	*P*-value in the hypergeometric test**	No. of genes in introgression regions	No. of genes in non-introgression regions	No. of RGA genes in introgression regions	No. of RGA genes in non-introgression regions	Enrichment of RLP genes*	*P*-value in the hypergeometric test**	No. of genes in introgression regions	No. of genes in non-introgression regions	No. of RGA genes in introgression regions	No. of RGA genes in non-introgression regions	Enrichment of RLK genes*	*P*-value in the hypergeometric test**	No. of genes in introgression regions	No. of genes in non-introgression regions	No. of RGA genes in introgression regions	No. of RGA genes in non-introgression regions	Enrichment of TM-CC genes*	*P*-value in the hypergeometric test**
(C. fordii, C. fabri), C. sclerophylla	422	40462	30	1464	1.9648	**0.0005**	422	40462	17	494	3.2996	**0.0000**	422	40462	4	168	2.2829	0.1034	422	40462	9	650	1.3276	0.2435	422	40462	0	153	0.0000	1.0000
(C. fordii, C. tibetana), C. fabri	299	41187	22	1470	2.0616	**0.0013**	299	41187	10	496	2.7772	**0.0040**	299	41187	2	168	1.6399	0.3469	299	41187	8	652	1.6902	0.1068	299	41187	2	155	1.7774	0.3128
(C. fordii, C. lamontii), C. sclerophylla	536	40644	31	1466	1.6035	**0.0081**	536	40644	16	488	2.4862	**0.0011**	536	40644	6	166	2.7408	**0.0256**	536	40644	9	661	1.0325	0.5085	536	40644	0	152	0.0000	1.0000
(C. fordii, C. lamontii), C. fissa	409	42513	24	1509	1.6532	**0.0128**	409	42513	14	501	2.9046	**0.0005**	409	42513	1	171	0.6079	0.8080	409	42513	7	679	1.0716	0.4803	409	42513	2	159	1.3075	0.4547
(C. fordii, C. hystrix), C. lamontii	325	41274	20	1476	1.7208	**0.0146**	325	41274	9	488	2.3422	**0.0170**	325	41274	3	168	2.2678	0.1503	325	41274	7	669	1.3288	0.2787	325	41274	1	152	0.8355	0.6995
(C. fordii, C. carlesii), C. sclerophylla	537	39380	29	1425	1.4924	**0.0239**	537	39380	15	473	2.3256	**0.0028**	537	39380	7	155	3.3118	**0.0066**	537	39380	7	647	0.7934	0.7783	537	39380	0	151	0.0000	1.0000
(C. fordii, C. carlesii), C. chinensis	636	40591	33	1452	1.4505	**0.0244**	636	40591	13	482	1.7213	**0.0455**	636	40591	6	157	2.4391	**0.0414**	636	40591	9	661	0.8690	0.7076	636	40591	5	153	2.0857	0.0987
(C. fordii, C. fabri), C. fissa	316	42289	18	1505	1.6006	**0.0364**	316	42289	4	503	1.0642	0.5199	316	42289	3	175	2.2942	0.1466	316	42289	10	668	2.0034	**0.0312**	316	42289	1	160	0.8364	0.6991
(C. fordii, C. hystrix), C. sclerophylla	230	40509	14	1471	1.6763	**0.0433**	230	40509	8	488	2.8873	**0.0075**	230	40509	2	166	2.1220	0.2452	230	40509	4	665	1.0594	0.5236	230	40509	0	153	0.0000	1.0000
(C. fordii, C. lamontii), C. chinensis	602	41876	30	1495	1.3959	**0.0461**	602	41876	14	497	1.9595	**0.0153**	602	41876	4	168	1.6562	0.2281	602	41876	8	676	0.8232	0.7550	602	41876	4	155	1.7951	0.1899
(C. fargesii, C. carlesii), C. hystrix	237	39934	14	1436	1.6427	**0.0498**	237	39934	5	476	1.7699	0.1566	237	39934	2	157	2.1465	0.2414	237	39934	6	652	1.5506	0.1948	237	39934	1	152	1.1085	0.5963
(C. fordii, C. lamontii), C. jucunda	606	40645	30	1466	1.3725	0.0548	606	40645	11	487	1.5149	0.1195	606	40645	4	165	1.6260	0.2377	606	40645	13	664	1.3131	0.2003	606	40645	2	151	0.8884	0.6596
(C. fordii, C. fargesii), C. fissa	290	42522	16	1519	1.5445	0.0598	290	42522	8	505	2.3228	**0.0246**	290	42522	1	168	0.8728	0.6837	290	42522	6	685	1.2843	0.3276	290	42522	1	162	0.9051	0.6704
(C. fordii, C. eyrei), C. sclerophylla	741	40376	35	1460	1.3062	0.0714	741	40376	17	486	1.9060	**0.0107**	741	40376	6	161	2.0306	0.0826	741	40376	12	658	0.9937	0.5490	741	40376	0	156	0.0000	1.0000
(C. fordii, C. eyrei), C. fissa	477	40488	23	1461	1.3362	0.1025	477	40488	8	484	1.4030	0.2176	477	40488	3	161	1.5816	0.2987	477	40488	11	662	1.4104	0.1653	477	40488	1	155	0.5476	0.8397
(C. fargesii, C. carlesii), C. fabri	520	40289	24	1438	1.2931	0.1255	520	40289	9	481	1.4497	0.1765	520	40289	1	160	0.4842	0.8737	520	40289	13	647	1.5568	0.0825	520	40289	1	151	0.5131	0.8581
(C. fordii, C. fargesii), C. sclerophylla	392	40655	19	1476	1.3350	0.1281	392	40655	13	493	2.7348	**0.0013**	392	40655	3	162	1.9206	0.2096	392	40655	3	666	0.4672	0.9552	392	40655	0	156	0.0000	1.0000
(C. fordii, C. fabri), C. chinensis	450	41645	21	1488	1.3061	0.1340	450	41645	9	503	1.6559	0.1010	450	41645	3	169	1.6428	0.2794	450	41645	5	662	0.6990	0.8422	450	41645	4	155	2.3882	0.0917
(C. fordii, C. carlesii), C. fissa	368	41120	17	1462	1.2993	0.1683	368	41120	6	485	1.3823	0.2714	368	41120	2	160	1.3967	0.4218	368	41120	8	662	1.3503	0.2464	368	41120	1	156	0.7163	0.7538
(C. fordii, C. hystrix), C. fabri	320	41135	15	1470	1.3117	0.1771	320	41135	6	493	1.5645	0.1904	320	41135	3	170	2.2685	0.1502	320	41135	5	653	0.9843	0.5755	320	41135	1	155	0.8293	0.7021
(C. fargesii, C. carlesii), C. fordii	175	40907	9	1468	1.4331	0.1803	175	40907	4	490	1.9082	0.1608	175	40907	2	158	2.9589	0.1490	175	40907	2	665	0.7030	0.7790	175	40907	1	156	1.4984	0.4891
(C. lamontii, C. eyrei), C. carlesii	1854	40767	75	1474	1.1188	0.1822	1854	40767	27	492	1.2067	0.1951	1854	40767	13	161	1.7755	**0.0412**	1854	40767	26	666	0.8584	0.8046	1854	40767	9	156	1.2686	0.2916
(C. fordii, C. hystrix), C. tibetana	882	42315	37	1515	1.1717	0.1878	882	42315	8	505	0.7600	0.8239	882	42315	4	170	1.1289	0.4762	882	42315	18	683	1.2644	0.1920	882	42315	7	158	2.1255	0.0534
(C. fargesii, C. lamontii), C. fordii	159	42245	8	1510	1.4076	0.2114	159	42245	4	502	2.1171	0.1233	159	42245	0	169	0.0000	1.0000	159	42245	3	681	1.1704	0.4741	159	42245	1	159	1.6710	0.4524
(C. fordii, C. hystrix), C. jucunda	214	40460	10	1469	1.2870	0.2535	214	40460	6	493	2.3010	**0.0494**	214	40460	1	166	1.1389	0.5864	214	40460	2	661	0.5721	0.8657	214	40460	1	150	1.2604	0.5498
(C. fordii, C. hystrix), C. fargesii	341	41189	15	1488	1.2176	0.2560	341	41189	8	497	1.9443	0.0584	341	41189	3	166	2.1829	0.1626	341	41189	4	669	0.7222	0.8047	341	41189	0	157	0.0000	1.0000
(C. fordii, C. eyrei), C. chinensis	808	40515	33	1459	1.1341	0.2577	808	40515	13	486	1.3413	0.1828	808	40515	6	160	1.8803	0.1083	808	40515	9	660	0.6838	0.9079	808	40515	5	154	1.6280	0.2015
(C. fordii, C. tibetana), C. lamontii	424	41398	18	1475	1.1915	0.2590	424	41398	6	493	1.1883	0.3945	424	41398	1	166	0.5882	0.8183	424	41398	7	665	1.0278	0.5237	424	41398	4	152	2.5694	0.0750
(C. fordii, C. fargesii), C. chinensis	428	41908	18	1508	1.1688	0.2850	428	41908	6	505	1.1634	0.4135	428	41908	2	163	1.2014	0.4983	428	41908	5	682	0.7179	0.8256	428	41908	5	159	3.0791	**0.0260**
(C. fargesii, C. carlesii), C. tibetana	203	39974	9	1435	1.2350	0.3082	203	39974	4	480	1.6410	0.2298	203	39974	2	158	2.4926	0.1939	203	39974	3	648	0.9116	0.6412	203	39974	0	150	0.0000	1.0000
(C. fargesii, C. carlesii), C. lamontii	574	40577	23	1451	1.1205	0.3206	574	40577	10	482	1.4666	0.1531	574	40577	3	160	1.3255	0.3976	574	40577	9	659	0.9654	0.5879	574	40577	1	151	0.4682	0.8822
(C. fordii, C. tibetana), C. fargesii	309	41280	12	1485	1.0795	0.4356	309	41280	4	499	1.0709	0.5151	309	41280	1	165	0.8096	0.7107	309	41280	5	666	1.0029	0.5590	309	41280	2	156	1.7127	0.3283
(C. fargesii, C. eyrei), C. tibetana	435	39820	16	1441	1.0164	0.5095	435	39820	3	482	0.5698	0.8969	435	39820	3	158	1.7381	0.2528	435	39820	8	650	1.1266	0.4179	435	39820	2	152	1.2045	0.4971
(C. lamontii, C. eyrei), C. fargesii	1143	40506	40	1464	0.9683	0.6039	1143	40506	12	486	0.8750	0.7157	1143	40506	10	160	2.2149	**0.0194**	1143	40506	16	665	0.8527	0.7700	1143	40506	2	154	0.4602	0.9300
(C. fordii, C. tibetana), C. jucunda	208	40501	7	1468	0.9285	0.6312	208	40501	1	496	0.3926	0.9228	208	40501	0	164	0.0000	1.0000	208	40501	5	661	1.4729	0.2551	208	40501	1	148	1.3157	0.5345
(C. fordii, C. tibetana), C. fissa	30	42443	1	1524	0.9283	0.6662	30	42443	0	519	0.0000	1.0000	30	42443	0	172	0.0000	1.0000	30	42443	0	676	0.0000	1.0000	30	42443	1	158	8.9542	0.1065
(C. fordii, C. tibetana), C. sclerophylla	220	40576	7	1466	0.8807	0.6847	220	40576	4	495	1.4904	0.2833	220	40576	1	163	1.1315	0.5888	220	40576	1	658	0.2803	0.9725	220	40576	1	151	1.2214	0.5611
(C. fordii, C. chinensis), C. fissa	104	43000	3	1538	0.8065	0.7232	104	43000	2	515	1.6057	0.3551	104	43000	0	172	0.0000	1.0000	104	43000	1	692	0.5975	0.8151	104	43000	0	160	0.0000	1.0000
(C. jucunda, C. chinensis), C. hystrix	429	41940	13	1508	0.8428	0.7703	429	41940	3	501	0.5854	0.8865	429	41940	2	168	1.1638	0.5149	429	41940	5	683	0.7157	0.8275	429	41940	3	157	1.8681	0.2211
(C. fordii, C. fabri), C. jucunda	439	40442	13	1457	0.8220	0.7986	439	40442	4	492	0.7490	0.7811	439	40442	2	167	1.1033	0.5433	439	40442	5	647	0.7119	0.8308	439	40442	2	152	1.2121	0.4938
(C. fordii, C. carlesii), C. jucunda	575	39310	17	1425	0.8156	0.8325	575	39310	4	476	0.5745	0.9168	575	39310	3	155	1.3232	0.3987	575	39310	8	647	0.8453	0.7294	575	39310	2	148	0.9239	0.6388
(C. jucunda, C. chinensis), C. tibetana	378	41934	10	1505	0.7371	0.8723	378	41934	4	509	0.8718	0.6743	378	41934	1	165	0.6723	0.7752	378	41934	3	678	0.4909	0.9438	378	41934	2	154	1.4407	0.4068
(C. sclerophylla, C. jucunda), C. fissa	67	41649	1	1506	0.4128	0.9152	67	41649	0	504	0.0000	1.0000	67	41649	0	170	0.0000	1.0000	67	41649	1	675	0.9209	0.6656	67	41649	0	158	0.0000	1.0000
(C. fargesii, C. eyrei), C. hystrix	472	40359	12	1465	0.7004	0.9229	472	40359	2	489	0.3497	0.9782	472	40359	2	161	1.0622	0.5637	472	40359	6	662	0.7750	0.7858	472	40359	2	154	1.1105	0.5400
(C. jucunda, C. chinensis), C. fordii	345	43017	8	1547	0.6448	0.9299	345	43017	2	518	0.4814	0.9200	345	43017	3	170	2.2004	0.1599	345	43017	2	698	0.3573	0.9761	345	43017	1	162	0.7697	0.7287
(C. fordii, C. eyrei), C. jucunda	737	38850	20	1421	0.7419	0.9325	737	38850	5	477	0.5526	0.9467	737	38850	3	155	1.0203	0.5660	737	38850	10	643	0.8198	0.7757	737	38850	2	147	0.7172	0.7679
(C. fargesii, C. eyrei), C. fordii	415	40403	10	1462	0.6659	0.9336	415	40403	3	488	0.5985	0.8775	415	40403	1	157	0.6201	0.8017	415	40403	5	663	0.7342	0.8108	415	40403	1	155	0.6281	0.7975
(C. fordii, C. fargesii), C. jucunda	433	40632	10	1474	0.6366	0.9524	433	40632	3	490	0.5745	0.8938	433	40632	1	163	0.5757	0.8248	433	40632	4	668	0.5619	0.9253	433	40632	2	154	1.2187	0.4909
(C. fargesii, C. lamontii), C. fabri	321	41690	5	1474	0.4406	0.9890	321	41690	1	494	0.2629	0.9781	321	41690	0	170	0.0000	1.0000	321	41690	4	657	0.7907	0.7453	321	41690	0	154	0.0000	1.0000
(C. chinensis, C. sclerophylla), C. jucunda	1924	41653	51	1505	0.7336	0.9912	1924	41653	18	504	0.7732	0.8860	1924	41653	7	168	0.9020	0.6576	1924	41653	18	675	0.5773	0.9955	1924	41653	8	159	1.0893	0.4581
(C. fargesii, C. eyrei), C. fabri	798	40117	17	1446	0.5910	0.9933	798	40117	3	481	0.3135	0.9960	798	40117	1	160	0.3142	0.9583	798	40117	10	654	0.7687	0.8357	798	40117	3	152	0.9922	0.5846

*The ratio of RGAs in the irogression regions relative to non-introgression regions. For example Enrichment of RGA genes = (No. of RGA genes in introgression regions / No. of genes in introgression regions) / (No. of RGA genes in non-introgression regions / No. of genes in non-introgression regions)

**Significant test ($P < 0.05$) was highlighted in bold.

Table S12 Gene Ontology (GO) terms enriched for genes located in introgression regions of 30 trios. The column "Ontology" illustrates the biological domain of Ontology in three aspects: MF (Molecular Function), BP (Biological Process), and CC (Cellular Component). The column "Description" provides a brief overview of the function associated with the enriched GO term. The column "*P*-value" indicates the significance of the enrichment test, while "FDR" represents the Benjamini-Hochberg false discovery rate used for correcting multiple testing.

GO term*	Ontology	Description	*P*-value	FDR
(*C. chinensis*, *C. sclerophylla*), *C. jucunda*				
GO:0016701	MF	oxidoreductase activity, acting on single donors with incorporation of molecular oxygen	0.0001	0.0186
GO:0003868	MF	4-hydroxyphenylpyruvate dioxygenase activity	0.0001	0.0186
(*C. fargesii*, *C. carlesii*), *C. lamontii*				
GO:0000278	BP	mitotic cell cycle	0	0.0059
(*C. fargesii*, *C. carlesii*), *C. tibetana*				
GO:0051225	BP	spindle assembly	0.0007	0.0449
GO:0070652	CC	HAUS complex	0.0004	0.0372
GO:0120013	MF	lipid transfer activity	0.0002	0.0278
(*C. fargesii*, *C. eyrei*), *C. fabri*				
GO:0004523	MF	RNA-DNA hybrid ribonuclease activity	0	0.0025
(C. fargesii, C. eyrei), C. hystrix				
GO:0006414	BP	translational elongation	0.0001	0.0122
GO:0020037	MF	heme binding	0	0.0025
GO:0016705	MF	oxidoreductase activity, acting on paired donors, with incorporation or reduction of molecular oxygen	0.0001	0.0093
GO:0003746	MF	translation elongation factor activity	0.0002	0.0148
GO:0005506	MF	iron ion binding	0.0002	0.0148
(*C. fargesii*, *C. eyrei*), *C. tibetana*				
GO:0006414	BP	translational elongation	0.0001	0.0201
GO:0003746	MF	translation elongation factor activity	0.0001	0.0201
(*C. fargesii*, *C. lamontii*), *C. fordii*				
GO:0046938	BP	phytochelatin biosynthetic process	0	0.001
GO:0010038	BP	response to metal ion	0	0.003
GO:0005975	BP	carbohydrate metabolic process	0.0001	0.0037
GO:0006400	BP	tRNA modification	0.0004	0.0165
GO:0016756	MF	glutathione gamma-glutamylcysteinyltransferase activity	0	0.001
GO:0008193	MF	tRNA guanylyltransferase activity	0	0.001
GO:0004553	MF	hydrolase activity, hydrolyzing O-glycosyl compounds	0.0002	0.0104
(*C. fordii*, *C. carlesii*), *C. chinensis*				
GO:0004523	MF	RNA-DNA hybrid ribonuclease activity	0.0001	0.0272
(*C. fordii*, *C. carlesii*), *C. fissa*				
GO:0019464	BP	glycine decarboxylation via glycine cleavage system	0.0001	0.0209
GO:0005960	CC	glycine cleavage complex	0.0001	0.0209
(*C. fordii*, *C. carlesii*), *C. sclerophylla*				
GO:0006368	BP	transcription elongation from RNA polymerase II promoter	0.0006	0.0356
GO:0030001	BP	metal ion transport	0.0007	0.0356
GO:0019464	BP	glycine decarboxylation via glycine cleavage system	0.0008	0.0356
GO:0016593	CC	Cdc73/Paf1 complex	0.0006	0.0356
GO:0005960	CC	glycine cleavage complex	0.0008	0.0356
GO:0046873	MF	metal ion transmembrane transporter activity	0	0.006
GO:0008168	MF	methyltransferase activity	0.0002	0.029
GO:0004930	MF	G protein-coupled receptor activity	0.0005	0.0356
(*C. fordii*, *C. eyrei*), *C. fissa*				
GO:0048544	BP	recognition of pollen	0	0.0026
GO:0019464	BP	glycine decarboxylation via glycine cleavage system	0.0002	0.0214
GO:0005960	CC	glycine cleavage complex	0.0002	0.0214
(*C. fordii*, *C. fabri*), *C. chinensis*				
GO:0019464	BP	glycine decarboxylation via glycine cleavage system	0.0004	0.0383
GO:0005960	CC	glycine cleavage complex	0.0004	0.0383
GO:0016868	MF	intramolecular transferase activity, phosphotransferases	0.0001	0.0289
GO:0004930	MF	G protein-coupled receptor activity	0.0004	0.0383
GO:0043531	MF	ADP binding	0.0007	0.0467
(*C. fordii*, *C. fabri*), *C. fissa*				
GO:0019464	BP	glycine decarboxylation via glycine cleavage system	0.0002	0.0216
GO:0005960	CC	glycine cleavage complex	0.0002	0.0216
(C. fordii, C. fabri), C. jucunda				
GO:0009733	BP	response to auxin	0.0009	0.0456
GO:0010333	MF	terpene synthase activity	0	0.0014
GO:0016829	MF	lyase activity	0	0.0014
GO:0000287	MF	magnesium ion binding	0.0003	0.0272
GO:0004930	MF	G protein-coupled receptor activity	0.0003	0.0272
GO:0004970	MF	ionotropic glutamate receptor activity	0.0007	0.0454
(*C. fordii*, *C. fabri*), *C. sclerophylla*				
GO:0019464	BP	glycine decarboxylation via glycine cleavage system	0.0004	0.0308
GO:0005960	CC	glycine cleavage complex	0.0004	0.0308
GO:0043531	MF	ADP binding	0	0.0085
GO:0004970	MF	ionotropic glutamate receptor activity	0.0001	0.0127
GO:0004930	MF	G protein-coupled receptor activity	0.0004	0.0308
(*C. fordii*, *C. fargesii*), *C. chinensis*				
GO:0019464	BP	glycine decarboxylation via glycine cleavage system	0.0004	0.0347
GO:0005960	CC	glycine cleavage complex	0.0004	0.0347
GO:0071949	MF	FAD binding	0.0003	0.0347

GO:0004930	MF	G protein-coupled receptor activity	0.0004	0.0347
(*C. fordii*, *C. fargesii*), *C. fissa*				
GO:0043531	MF	ADP binding	0.0001	0.0352
(*C. fordii*, *C. fargesii*), *C. jucunda*				
GO:0016868	MF	intramolecular transferase activity, phosphotransferases	0	0.0087
GO:0004930	MF	G protein-coupled receptor activity	0.0003	0.0428
(*C. fordii*, *C. hystrix*), *C. fabri*				
GO:0004523	MF	RNA-DNA hybrid ribonuclease activity	0.0001	0.0195
(*C. fordii*, *C. hystrix*), *C. jucunda*				
GO:0004252	MF	serine-type endopeptidase activity	0.0001	0.0183
(*C. fordii*, *C. hystrix*), *C. tibetana*				
GO:0004869	MF	cysteine-type endopeptidase inhibitor activity	0	0.0026
GO:0004650	MF	polygalacturonase activity	0.0003	0.0422
(*C. fordii*, *C. lamontii*), *C. fissa*				
GO:0043531	MF	ADP binding	0	0.0027
(*C. fordii*, *C. lamontii*), *C. sclerophylla*				
GO:0030001	BP	metal ion transport	0.0005	0.039
GO:0006368	BP	transcription elongation from RNA polymerase II promoter	0.0006	0.039
GO:0016593	CC	Cdc73/Paf1 complex	0.0006	0.039
GO:0046873	MF	metal ion transmembrane transporter activity	0.0003	0.039
GO:0004970	MF	ionotropic glutamate receptor activity	0.0004	0.039
GO:0004930	MF	G protein-coupled receptor activity	0.0008	0.0484
(*C. fordii*, *C. tibetana*), *C. fabri*				
GO:0020037	MF	heme binding	0	0.0027
GO:0043531	MF	ADP binding	0	0.0029
GO:0016705	MF	oxidoreductase activity, acting on paired donors, with incorporation or reduction of molecular oxygen	0	0.003
GO:0005506	MF	iron ion binding	0.0001	0.0102
GO:0120013	MF	lipid transfer activity	0.0005	0.028
GO:0120013	MF	lipid transfer activity	0	0.001
GO:0004869	MF	cysteine-type endopeptidase inhibitor activity	0	0.001
(*C. fordii*, *C. tibetana*), *C. jucunda*				
GO:0003690	MF	double-stranded DNA binding	0.0001	0.0266
(*C. fordii*, *C. tibetana*), *C. lamontii*				
GO:0019464	BP	glycine decarboxylation via glycine cleavage system	0.0005	0.0475
GO:0005960	CC	glycine cleavage complex	0.0005	0.0475
GO:0004869	MF	cysteine-type endopeptidase inhibitor activity	0.0004	0.0475
GO:0020037	MF	heme binding	0.0006	0.0475
(*C. fordii*, *C. tibetana*), *C. sclerophylla*				
GO:0006086	BP	acetyl-CoA biosynthetic process from pyruvate	0.0001	0.0157
GO:0004739	MF	pyruvate dehydrogenase (acetyl-transferring) activity	0.0001	0.0157
(*C. lamontii*, *C. eyrei*), *C. carlesii*				
GO:0000373	BP	Group II intron splicing	0.0003	0.0427
GO:0008193	MF	tRNA guanylyltransferase activity	0	0.003
GO:0043531	MF	ADP binding	0.0003	0.0427
(*C. lamontii*, *C. eyrei*), *C. fargesii*				
GO:0000373	BP	Group II intron splicing	0.0001	0.027
GO:0008193	MF	tRNA guanylyltransferase activity	0.0002	0.027
(*C. sclerophylla*, *C. jucunda*), *C. fissa*				
GO:0009765	BP	photosynthesis, light harvesting	0	0.002

*No GO terms were enriched for genes in introgression regions of the other trios.

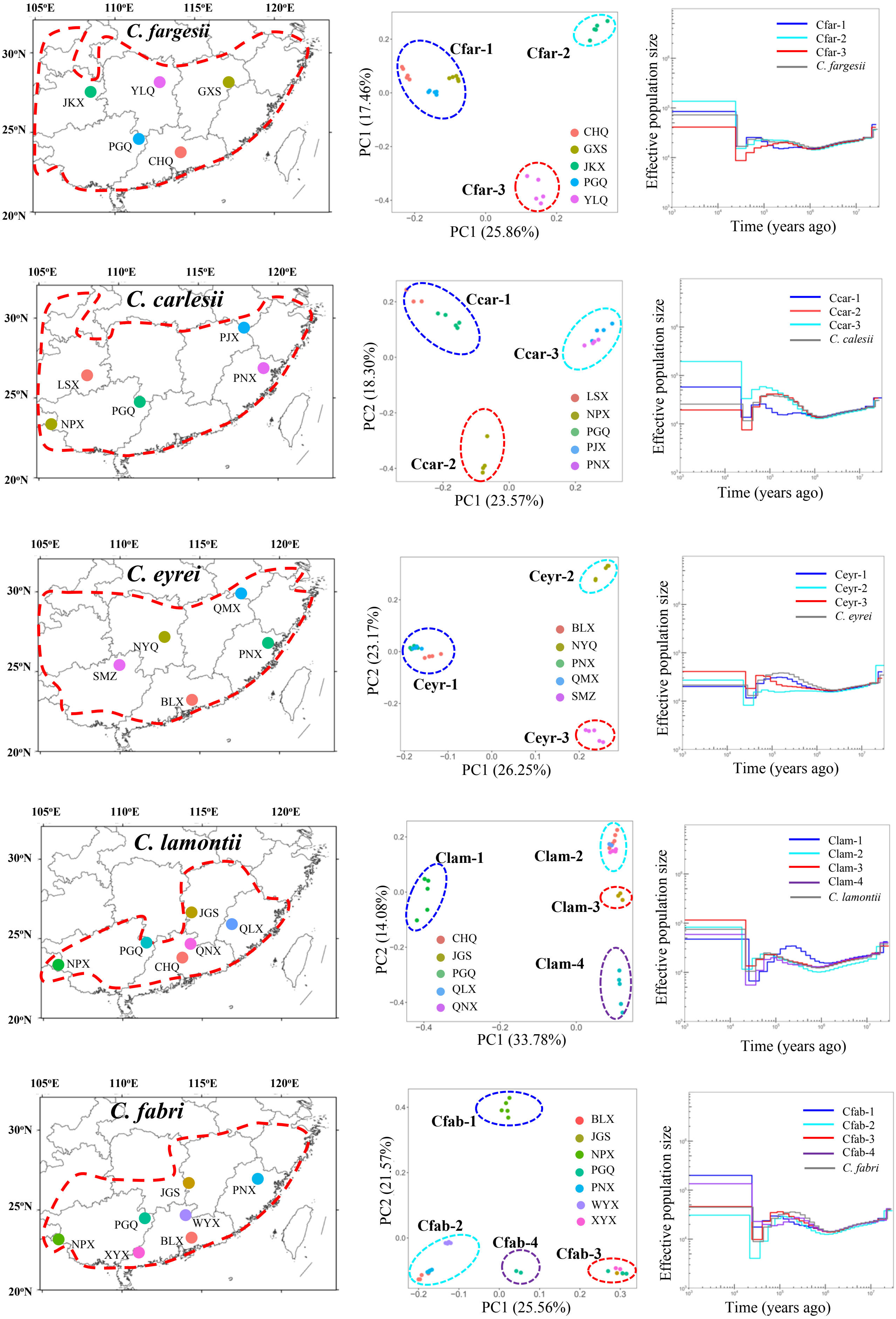

105°E
110°E
115°E
120°E
30°N
25°N
20°N
C. fargesii
JKX
YLQ
GXS
PGQ
CHQ
Cfar-1
Cfar-2
Cfar-3
PC1 (17.46%)
PC1 (25.86%)
CHQ
GXS
JKX
PGQ
YLQ
Cfar-1
Cfar-2
Cfar-3
C. fargesii
Effective population size
Time (years ago)
C. carlesii
PJX
PNX
LSX
PGQ
NPX
Ccar-1
Ccar-3
Ccar-2
PC2 (18.30%)
PC1 (23.57%)
LSX
NPX
PGQ
PJX
PNX
Ccar-1
Ccar-2
Ccar-3
C. calesii
C. eyrei
QMX
NYQ
PNX
SMZ
BLX
Ceyr-2
Ceyr-1
Ceyr-3
PC2 (23.17%)
PC1 (26.25%)
BLX
NYQ
PNX
QMX
SMZ
Ceyr-1
Ceyr-2
Ceyr-3
C. eyrei
C. lamontii
JGS
QLX
PGQ
QNX
NPX
CHQ
Clam-1
Clam-2
Clam-3
Clam-4
PC2 (14.08%)
PC1 (33.78%)
CHQ
JGS
PGQ
QLX
QNX
Clam-1
Clam-2
Clam-3
Clam-4
C. lamontii
C. fabri
PNX
JGS
PGQ
WYX
NPX
BLX
XYX
Cfab-1
Cfab-2
Cfab-4
Cfab-3
PC2 (21.57%)
PC1 (25.56%)
BLX
JGS
NPX
PGQ
PNX
WYX
XYX
Cfab-1
Cfab-2
Cfab-3
Cfab-4
C. fabri

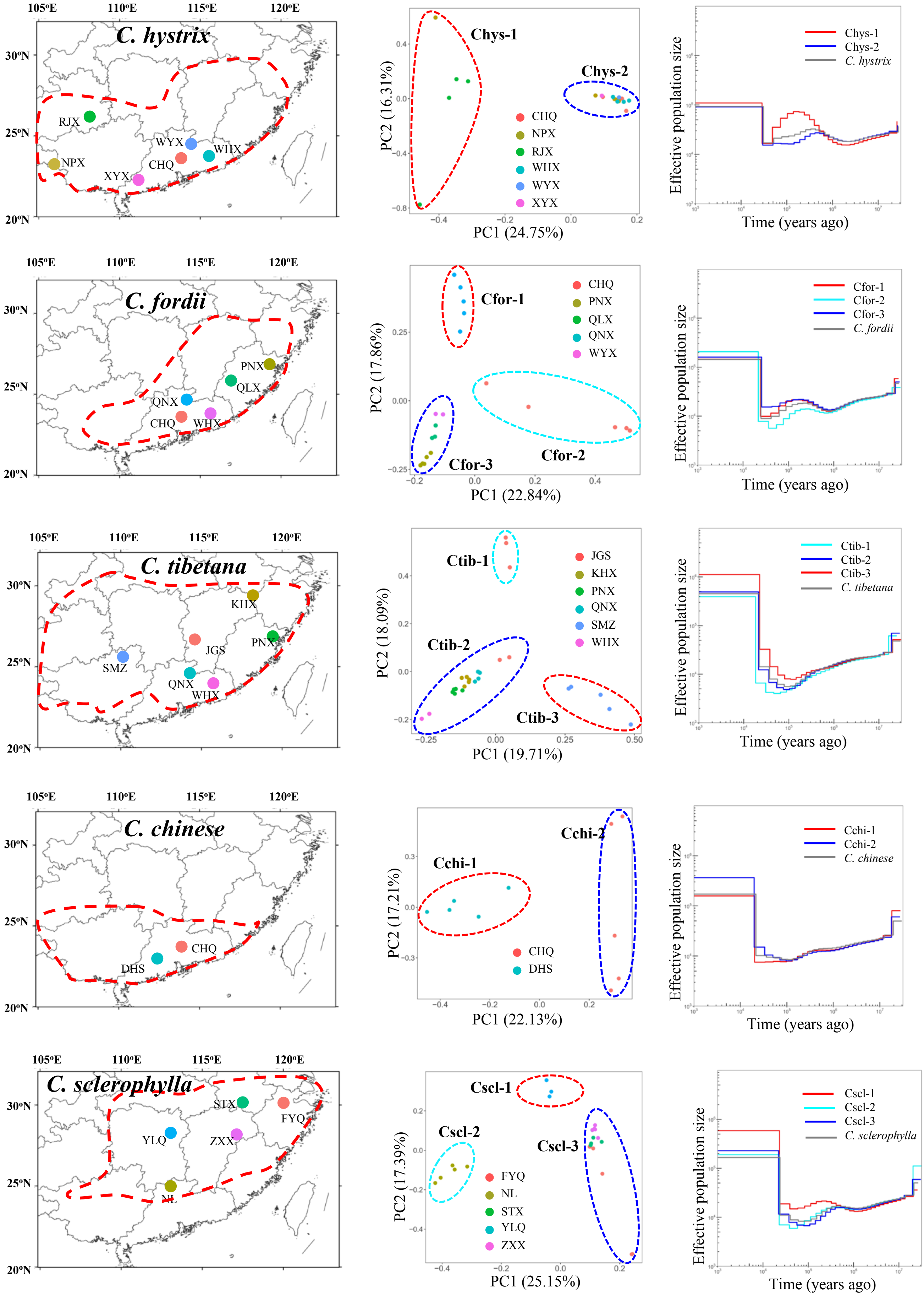
C. hystrix
105ºE
110ºE
115ºE
120ºE
30ºN
25ºN
20ºN
RJX
NPX
WYX
WHX
CHQ
XYX
Chys-1
Chys-2
CHQ
NPX
RJX
WHX
WYX
XYX
PC1 (24.75%)
PC2 (16.31%)
Chys-1
Chys-2
C. hystrix
Effective population size
Time (years ago)
C. fordii
PNX
QLX
QNX
CHQ
WHX
Cfor-1
Cfor-2
Cfor-3
CHQ
PNX
QLX
QNX
WYX
PC1 (22.84%)
PC2 (17.86%)
Cfor-1
Cfor-2
Cfor-3
C. fordii
Effective population size
Time (years ago)
C. tibetana
KHX
JGS
PNX
SMZ
QNX
WHX
Ctib-1
Ctib-2
Ctib-3
JGS
KHX
PNX
QNX
SMZ
WHX
PC1 (19.71%)
PC2 (18.09%)
Ctib-1
Ctib-2
Ctib-3
C. tibetana
Effective population size
Time (years ago)
C. chinese
CHQ
DHS
Cchi-1
Cchi-2
CHQ
DHS
PC1 (22.13%)
PC2 (17.21%)
Cchi-1
Cchi-2
C. chinese
Effective population size
Time (years ago)
C. sclerophylla
STX
FYQ
YLQ
ZXX
NL
Cscl-1
Cscl-2
Cscl-3
FYQ
NL
STX
YLQ
ZXX
PC1 (25.15%)
PC2 (17.39%)
Cscl-1
Cscl-2
Cscl-3
C. sclerophylla
Effective population size
Time (years ago)

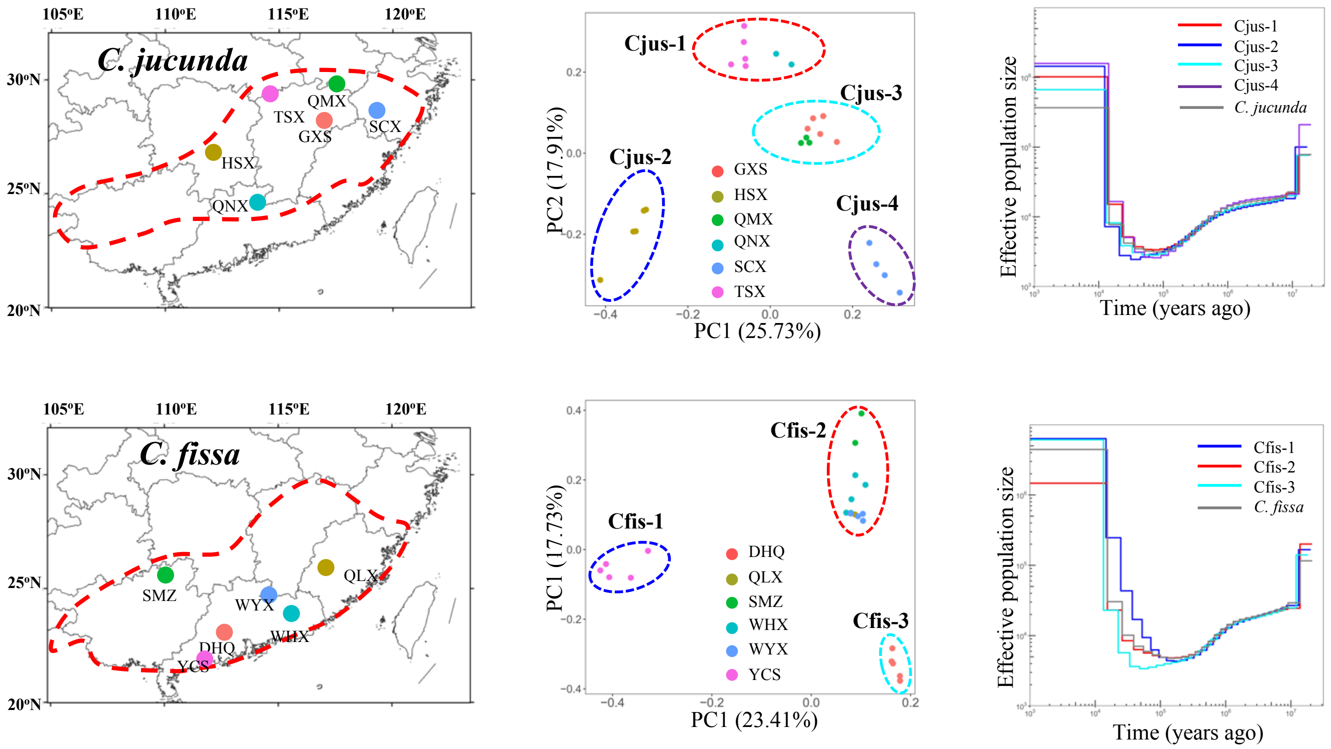

Fig. S1. Distribution range, population structure and demographic history of each *Castanopsis* species. Left panel: distribution range and sampled populations of each species. The red dashed line illustrates the distribution range based on specimen records, while colored dots indicate the location of the populations. Middle panel: population structure within each species revealed by principal component analysis (PCA). Genetic groups identified by PCA are outlined with dashed colored lines. Right panel: demographic history inferred for each genetic group (colored lines) and the entire species (grey line) using MSMC.

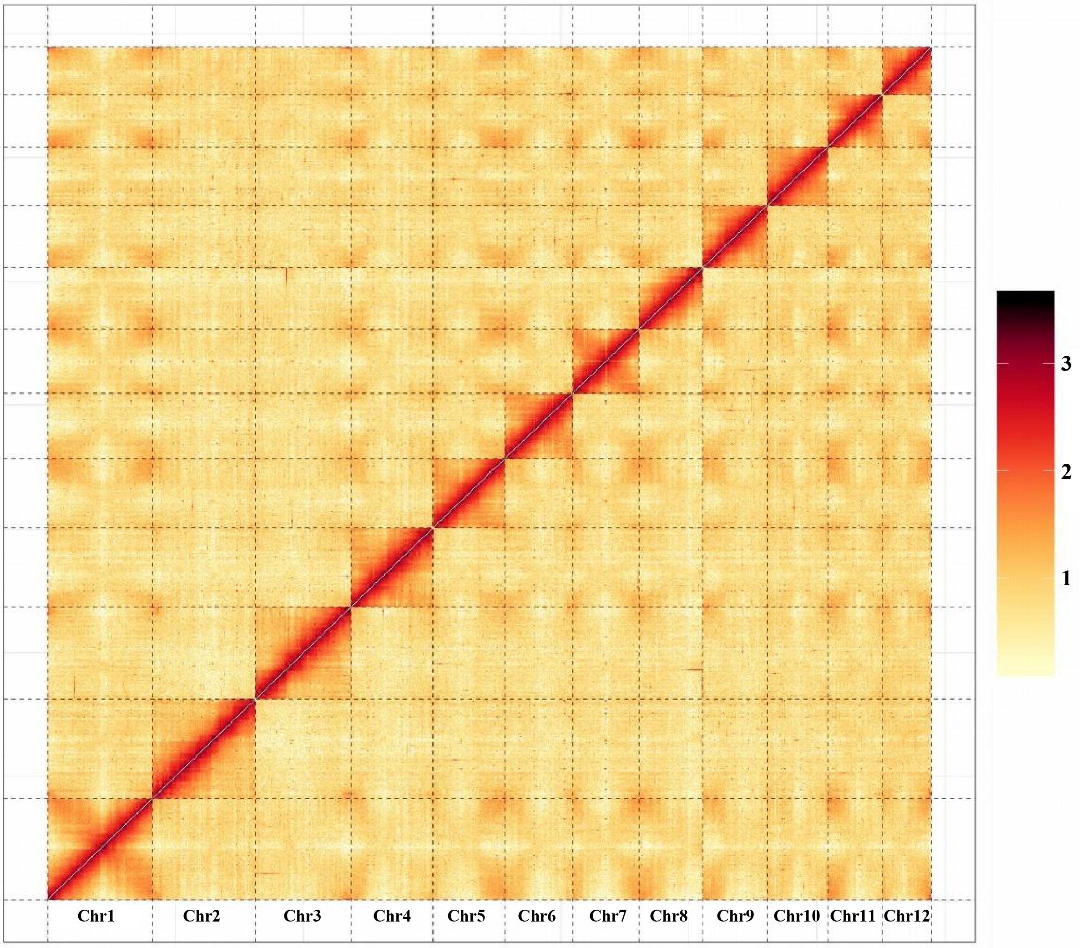

Fig. S2. Hi-C interaction heatmap for the 12 *C. eyrei* pseudo-chromosomes. The interactions within and between chromosomes were represented at a 100 kb resolution. Darker cells denote stronger and more frequent interactions.

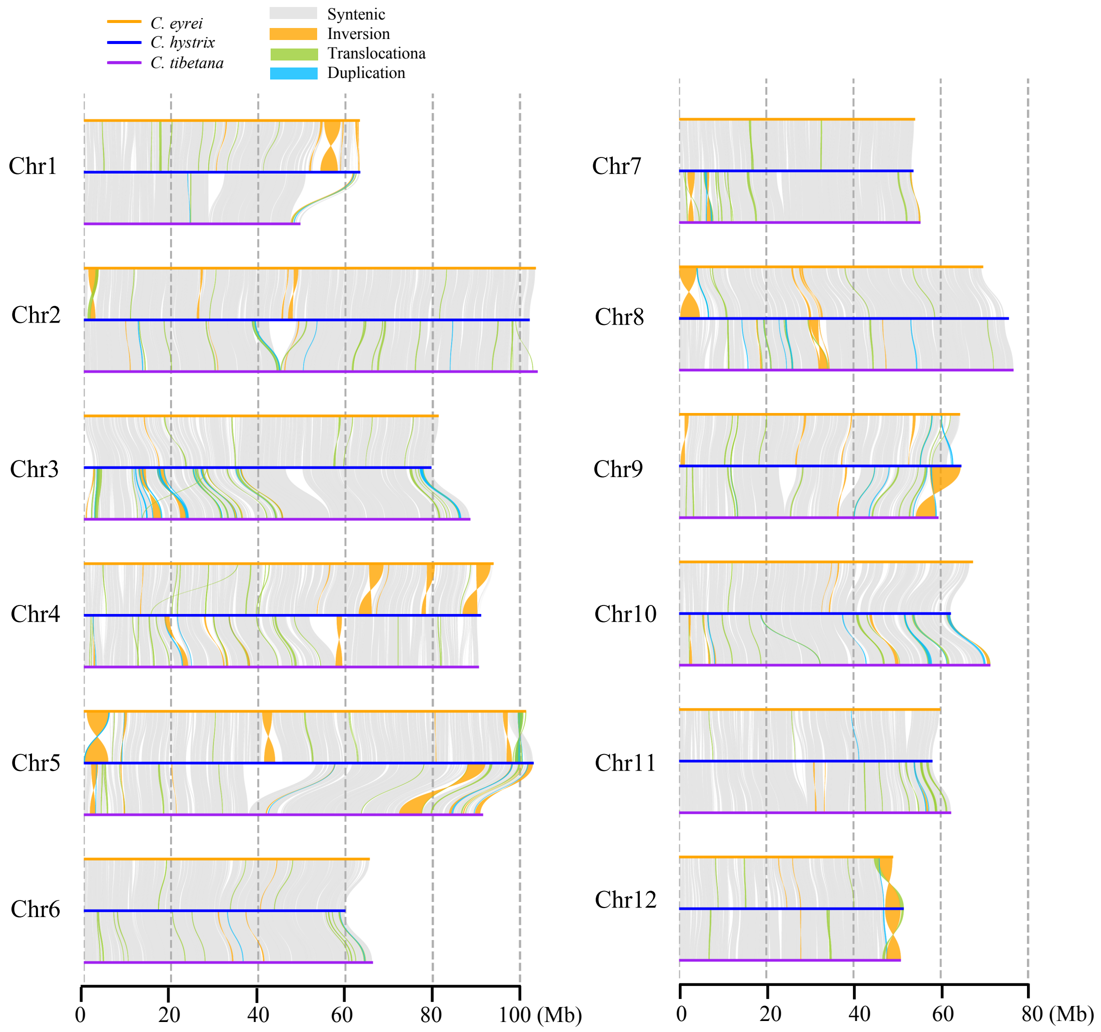

Fig. S3. Analysis of synteny and collinearity between the genomes of *C. eyrei*, *C. hystrix*, and *C. tibetana* using SyRI. Syntenic regions, inversions, translocations, and duplications larger than 100 kb are shown by different colors.

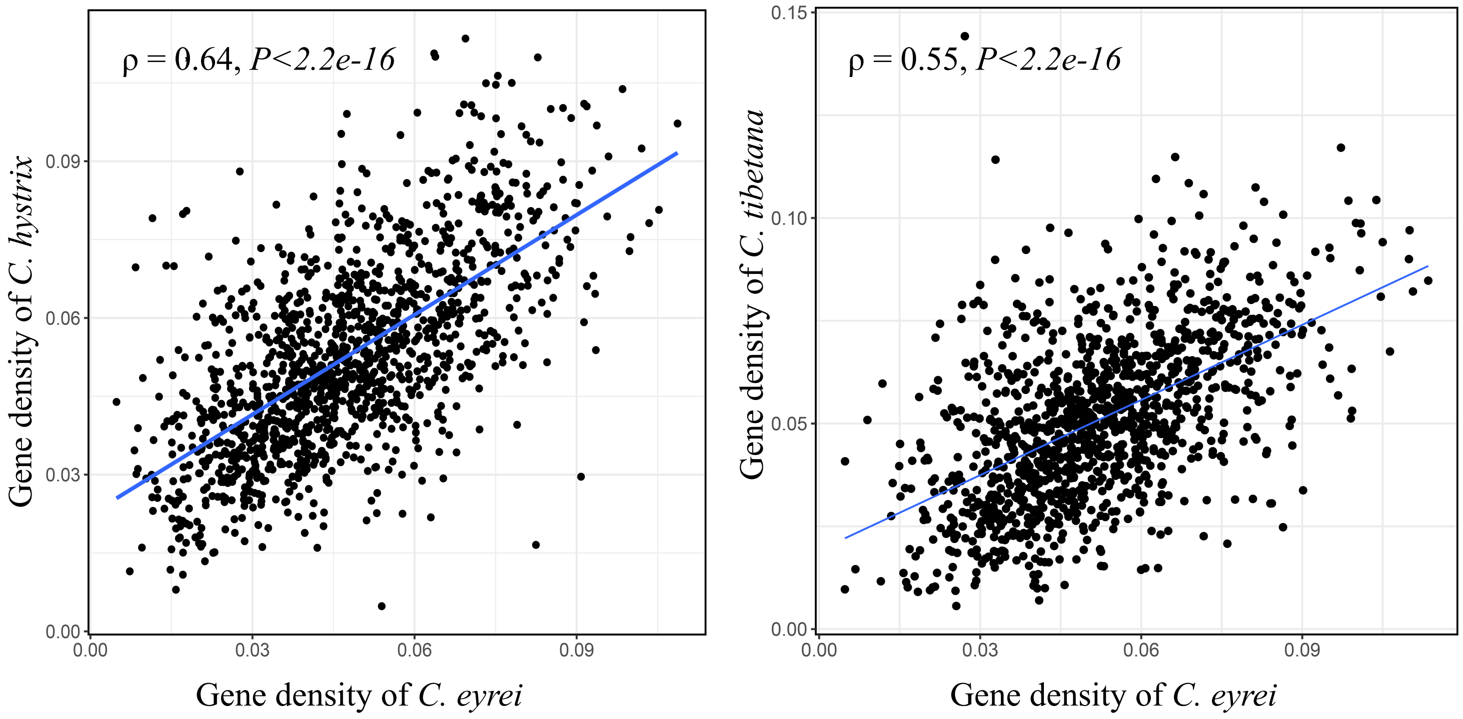

Fig. S4. Conserved gene densities between three *Castanopsis* species. Left panel: correlation of gene densities between the *C. eyrei* and *C. hystrix* genomes. Right panel: correlation of gene densities between the *C. eyrei* and *C. tibetana* genomes. Blue line, linear regression (lm) fit. Each black dot represents a 500 kb non-overlapping window.

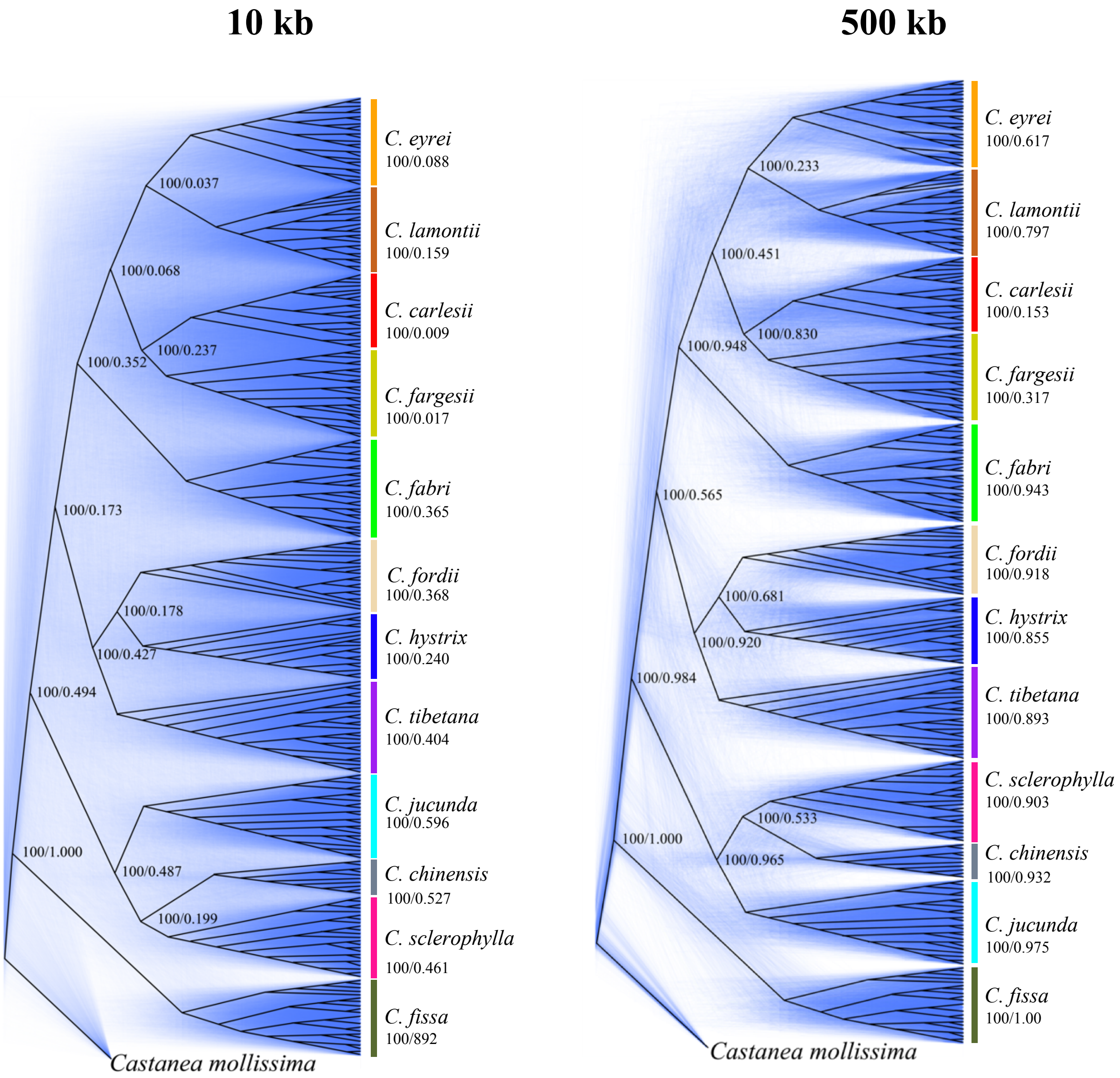

Fig. S5. Phylogenetic relationships between 12 *Castanopsis* species. The black tree was constructed based on genome-wide SNPs, while the blue trees represent 10 kb (left) and 500 kb (right) window-based trees. For each node or species, the first number is the bootstrap in the whole-genome tree, and the second number reflects the proportion of window-based trees supporting that clade.

A

B

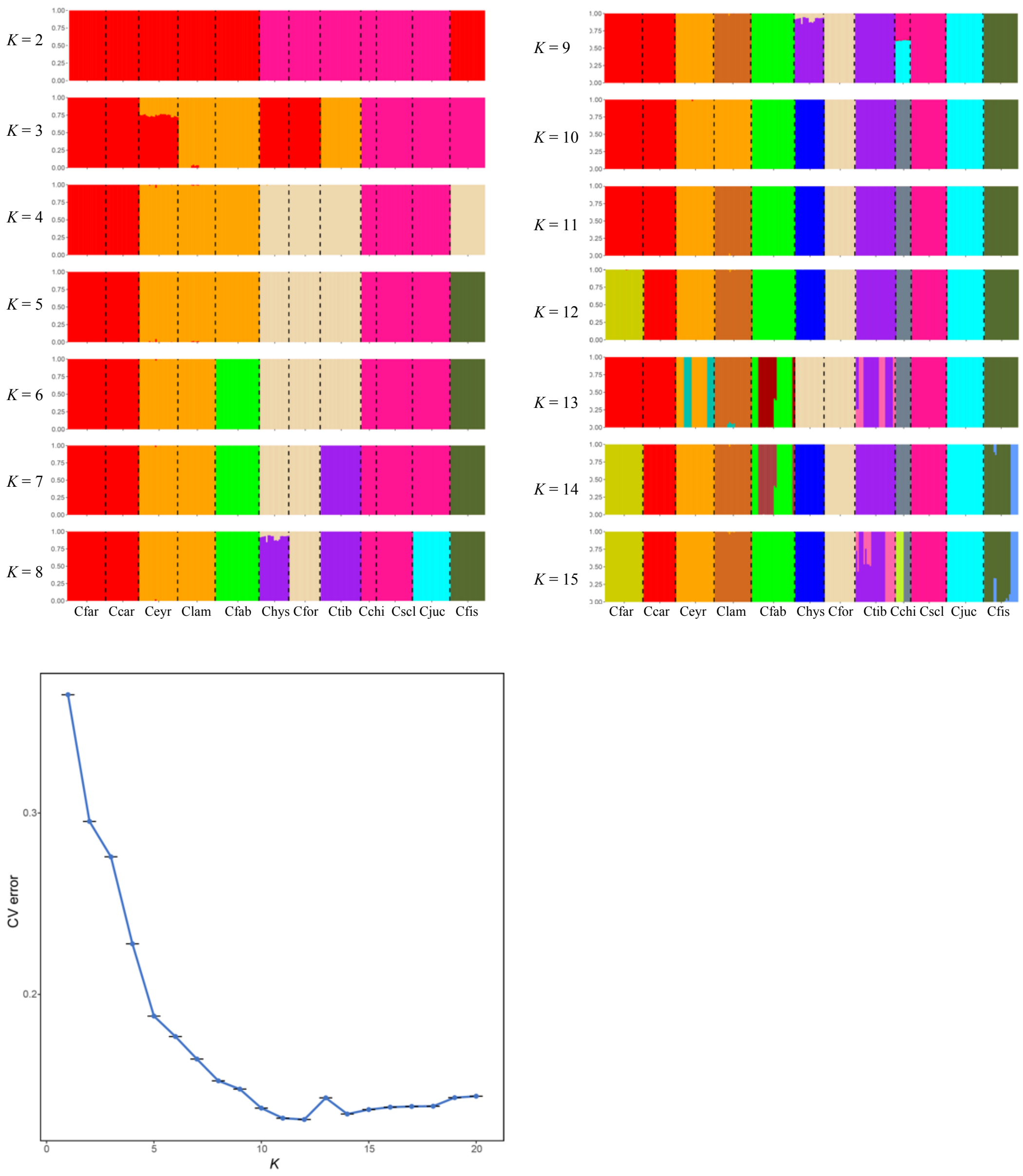

Fig. S6. Population structure of 12 *Castanopsis* species. (A) ADMIXTURE analyses for *K* = 2−15. Each bar represents an individual, with the various colors denoting distinct genetic ancestries. (B) Cross-validation error (CV-error) as *K* varies from 1 to 20. Black short lines indicate the standard deviation from 20 repetitions for each *K* value. Cfar, *C. fargesii*; Ccar, *C. carlesii*; Ceyr, *C. eyrei*; Clam, *C. lamontii*; Cfab, *C. fabri*; Chys, *C. hystrix*; Cfor, *C. fordii*; Ctib, *C. tibetana*; Cchi, *C. chinensis*; Cscl, *C. sclerophylla*; Cjuc, *C. jucunda*; Cfis, *C. fissa*.

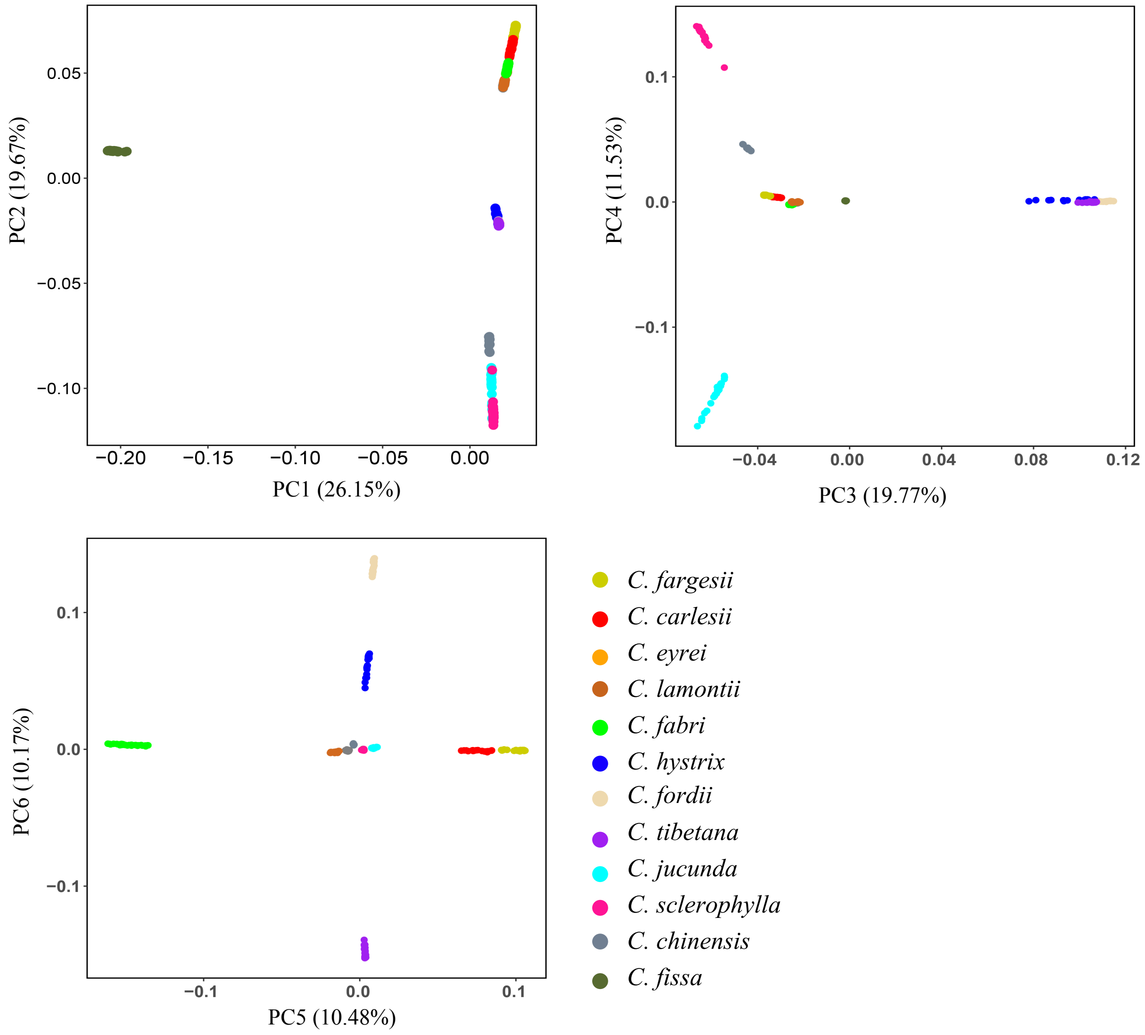

Fig. S7. Principal component analysis (PCA) of 12 *Castanopsis* species. Each colored dot represents an individual from these species, with the percentage of variance explained by the first six components (PC1–PC6) shown in parentheses.

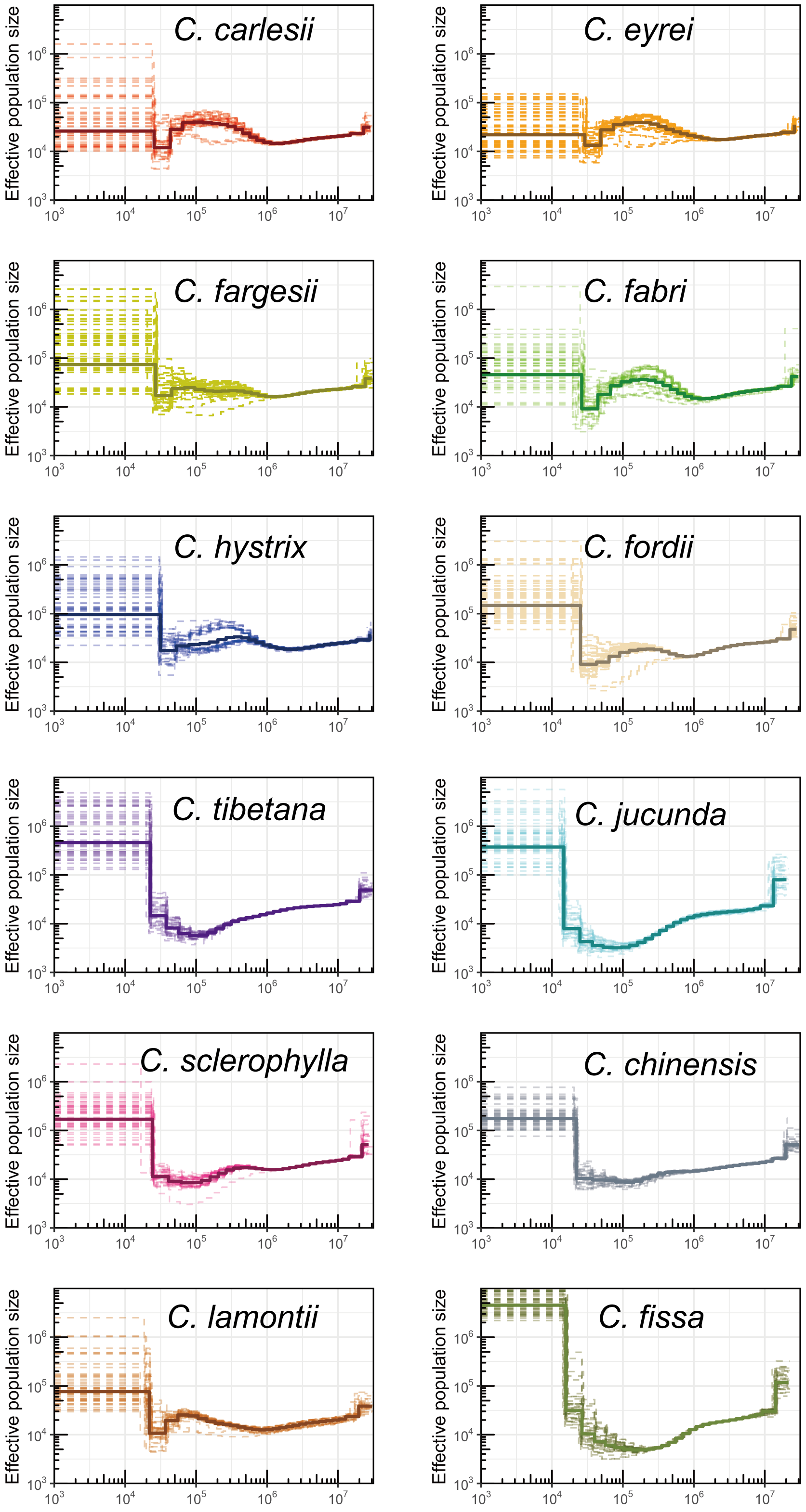

Fig. S8. Demographic history inference of 12 *Castanopsis* species using MSMC. The solid thick line denotes the mean value of the estimated effective population size (N_e) from 50 combinations of haplotypes, and each thin dashed line represents an individual replicate.

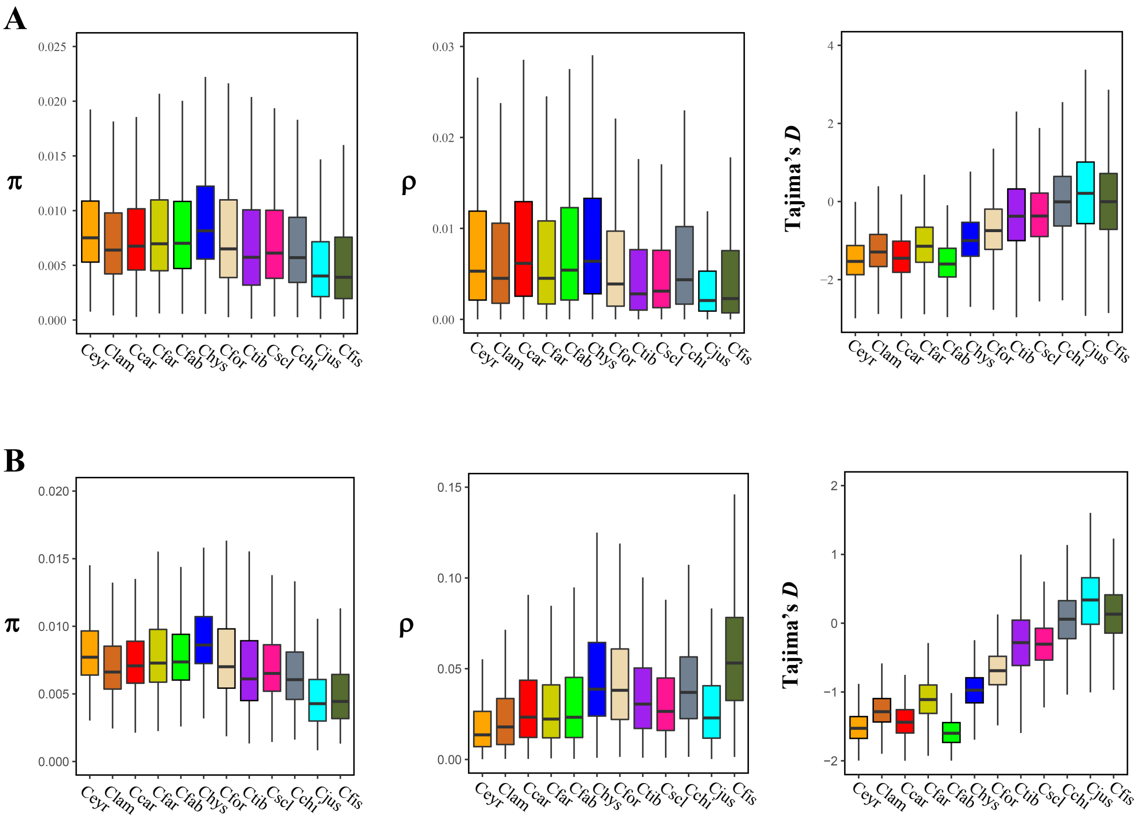

Fig. S9. Comparisons of nucleotide diversity (π), population-scaled recombination rate (*ρ*), and Tajima's *D* between 12 *Castanopsis* species. These three summary statistics were estimated in non-overlapping windows of 10 kb (A) and 500 kb (B). In each box plot, a horizontal line indicates the median, with the bottom and top of boxes representing the first and third quartiles, respectively. Whiskers extend to 1.5 times the interquartile range, and outliers are excluded from the plots. Ceyr, *C. eyrei*; Clam, *C. lamontii*; Ccar, *C. carlesii*; Cfar, *C. fargesii*; Cfab, *C. fabri*; Chys, *C. hystrix*; Cfor, *C. fordii*; Ctib, *C. tibetana*; Cscl, *C. sclerophylla*; Cchi, *C. chinensis*; Cjuc, *C. jucunda*; Cfis, *C. fissa*.

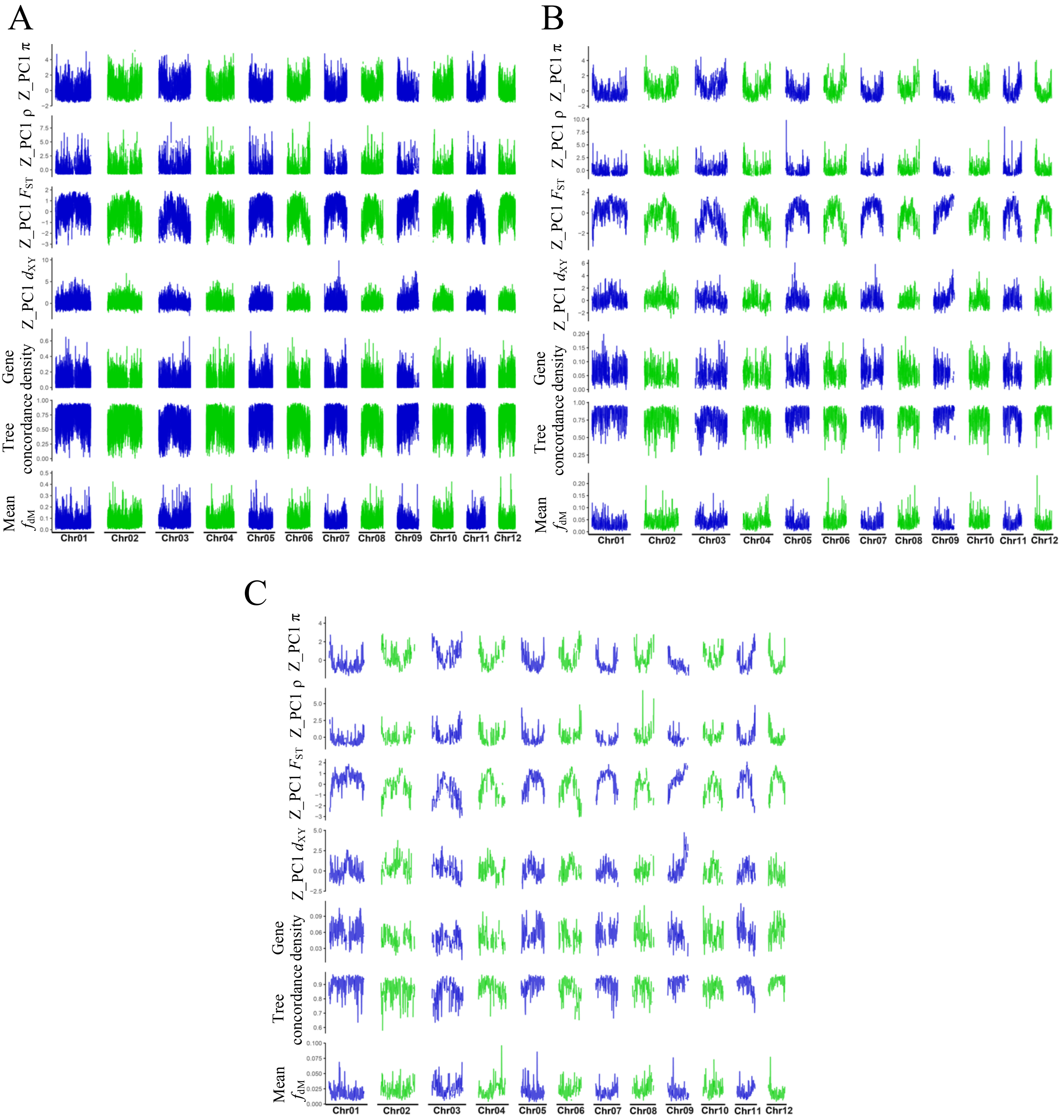

Fig. S10. Heterogeneous landscape of genomic variation across 12 *Castanopsis* species. PC1 scores of π, ρ, F_{ST}, and d_{XY} were *Z*-transformed, e.g. *Z*_PC1-F_{ST} = (per-window PC1-F_{ST} – mean PC1-F_{ST} across windows)/standard deviation of PC1-F_{ST} across windows. Mean f_{dM} represents the average across 51 trios for each window. All summary statistics are presented for non-overlapping genomic windows of 10 (A), 100 (B), and 500 (C) kb.

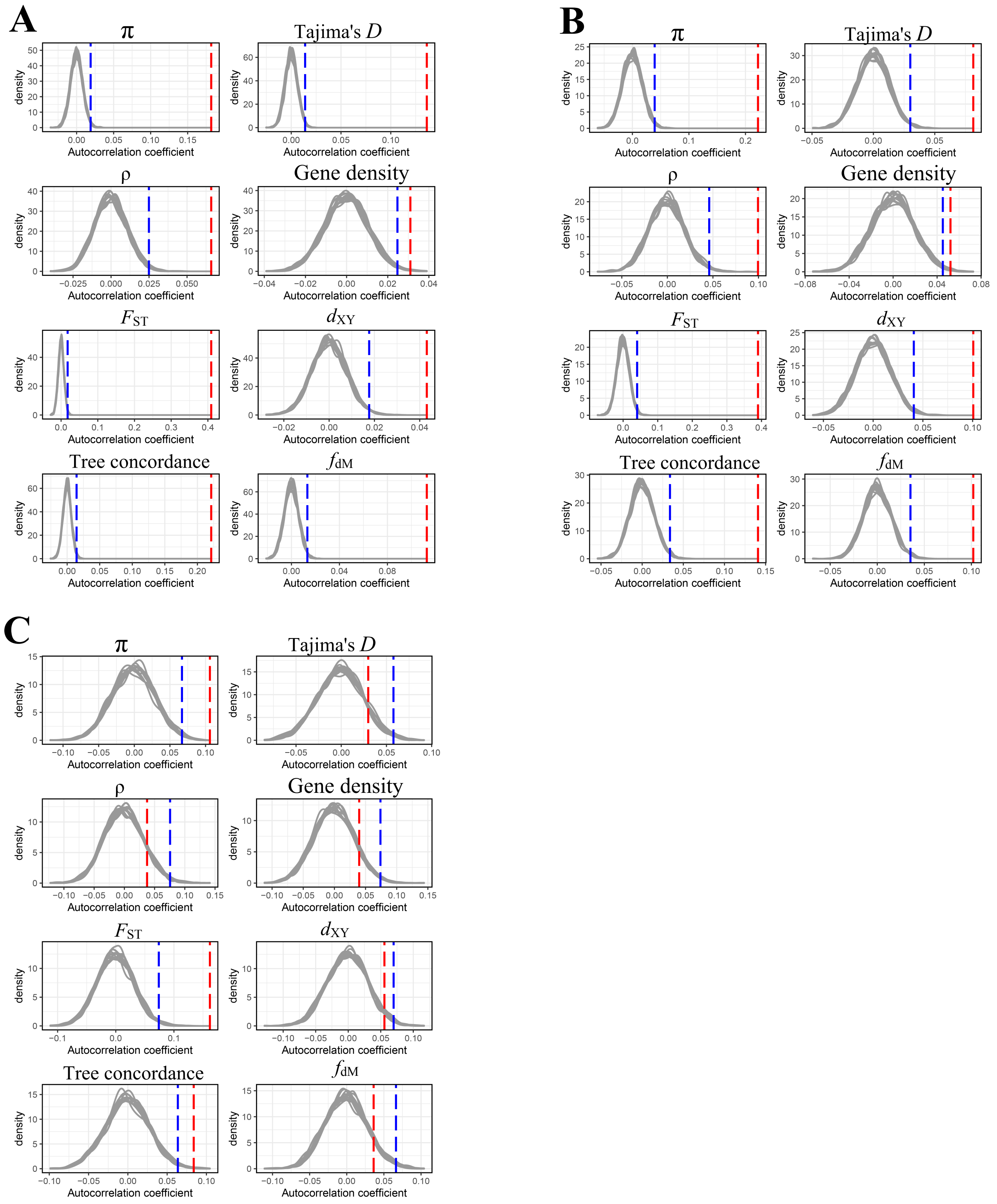

Fig. S11 Non-random distribution of population genetic parameters across the *Castanopsis* genome. Parameters were estimated using non-overlapping windows of 10 (A), 100 (B), and 500 kb. In each plot, gray lines show the distribution of autocorrelation coefficients generated from 1000 genome-wide data permutations. Blue dashed line: top 1% of autocorrelation coefficients from these permutations. Red dashed line: observed autocorrelation coefficient values.

10 kb

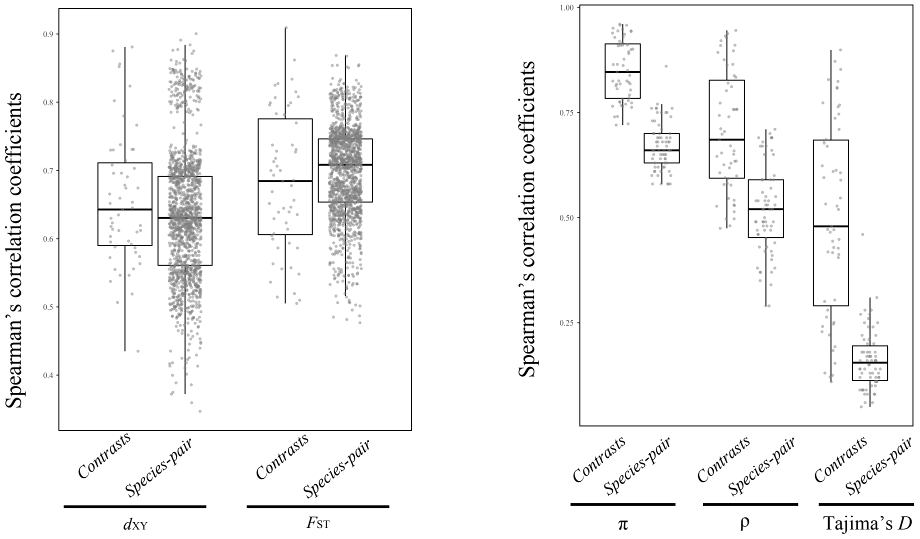

100 kb

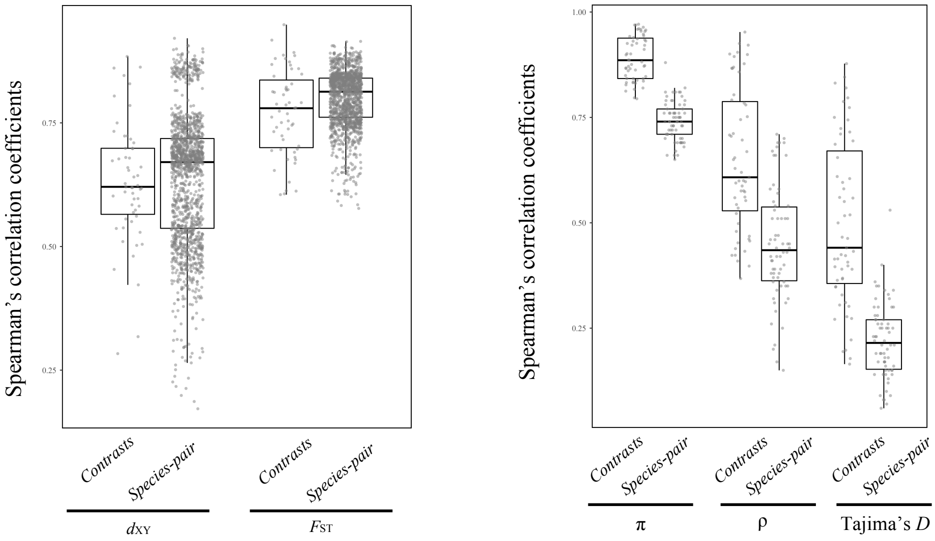

500 kb

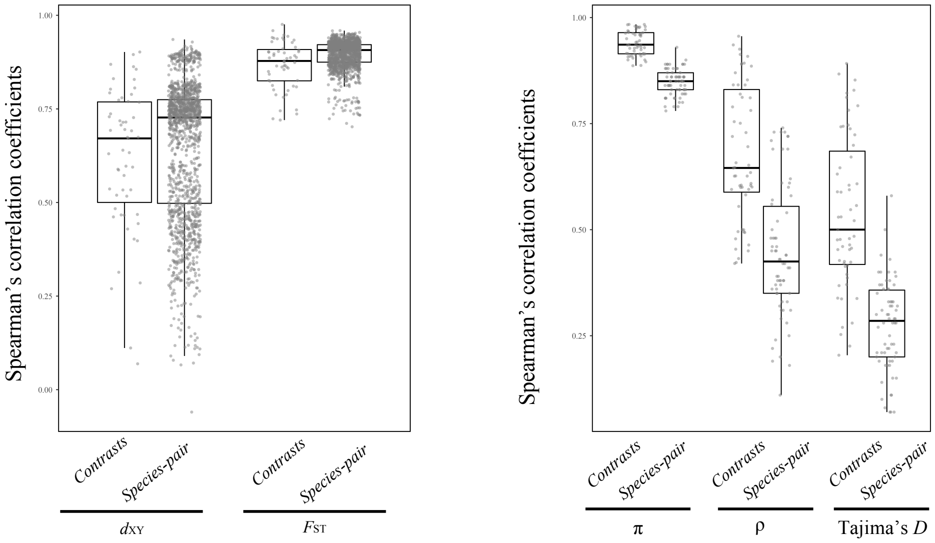

Fig. S12. Correlation between summary statistics for comparisons between species, species-pairs, and 11 contrasts. Summary statistics were estimated in non-overlapping windows of 10 kb (top panel), 100 kb (middle panel), and 500 kb (bottom panel). Estimates of F_{ST} and d_{XY} were calculated for each species-pair or contrasts, and then Spearman's correlation coefficients were determined for the estimates between all pairs of species-pairs or among the 11 contrasts. Estimates of π, ρ and Tajiam's *D* were calculated for each species or contrasts, and then Spearman's correlation coefficients were calculated for the estimates between all species-pairs, or between the 11 contrasts. In each plot, the grey dot represents a correlation coefficient for each comparison, and the horizontal line indicates the median, with the bottom and top of the boxes representing the first and third quartiles, respectively. Whiskers extend to 1.5 times the interquartile range, and outliers are excluded from the plots. Significant positive correlations were observed for all comparisons ($P < 0.05$), except for one d_{XY} comparison between species-pairs in the 500kb window.

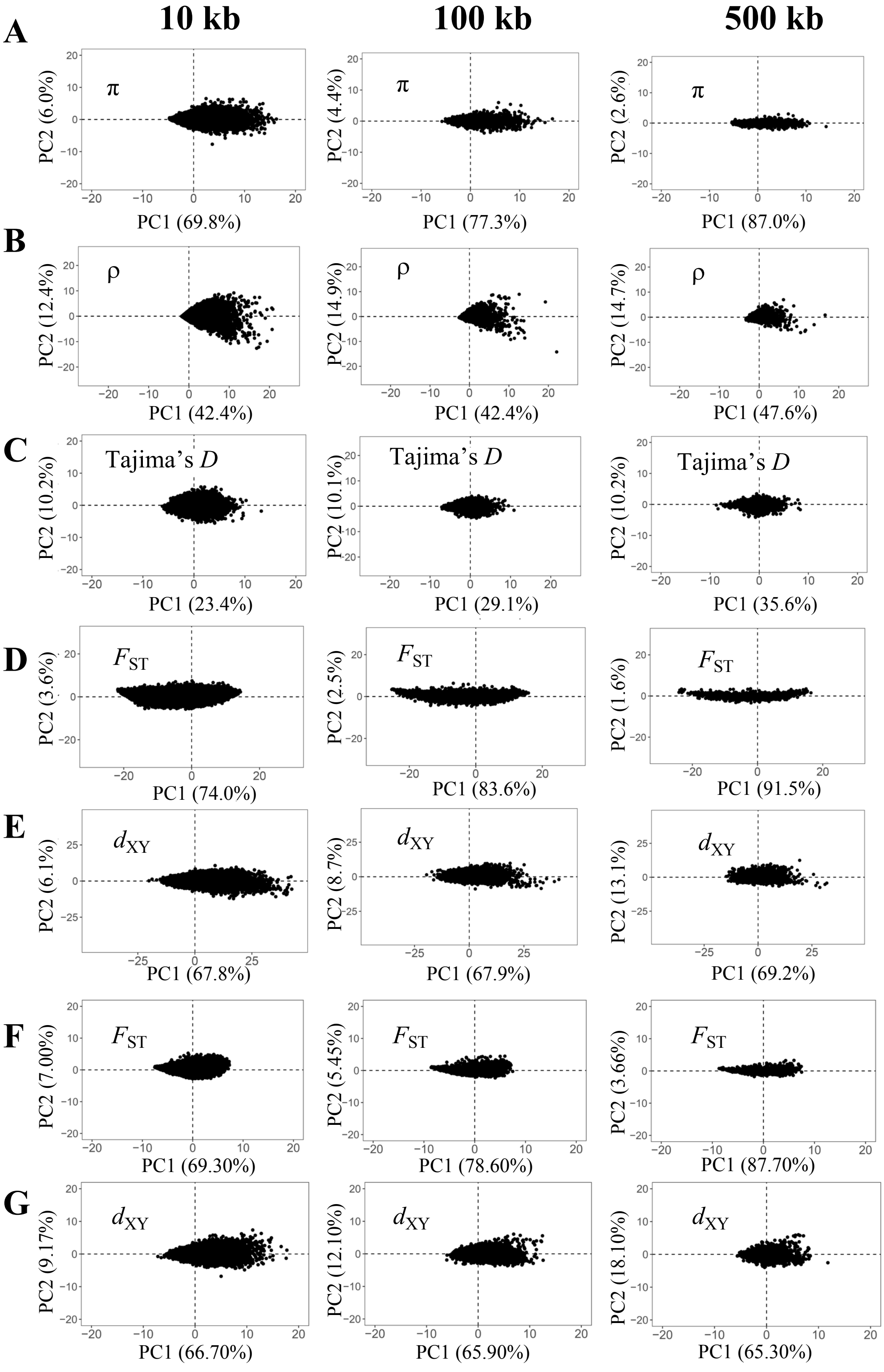
10 kb
100 kb
500 kb
A
π
PC2 (6.0%)
PC1 (69.8%)
PC2 (4.4%)
PC1 (77.3%)
PC2 (2.6%)
PC1 (87.0%)
B
ρ
PC2 (12.4%)
PC1 (42.4%)
PC2 (14.9%)
PC1 (42.4%)
PC2 (14.7%)
PC1 (47.6%)
C
Tajima's D
PC2 (10.2%)
PC1 (23.4%)
PC2 (10.1%)
PC1 (29.1%)
PC2 (10.2%)
PC1 (35.6%)
D
F_ST
PC2 (3.6%)
PC1 (74.0%)
PC2 (2.5%)
PC1 (83.6%)
PC2 (1.6%)
PC1 (91.5%)
E
d_XY
PC2 (6.1%)
PC1 (67.8%)
PC2 (8.7%)
PC1 (67.9%)
PC2 (13.1%)
PC1 (69.2%)
F
F_ST
PC2 (7.00%)
PC1 (69.30%)
PC2 (5.45%)
PC1 (78.60%)
PC2 (3.66%)
PC1 (87.70%)
G
d_XY
PC2 (9.17%)
PC1 (66.70%)
PC2 (12.10%)
PC1 (65.90%)
PC2 (18.10%)
PC1 (65.30%)

Fig. S13. Principal component analysis (PCA) summarizing the variation of each summary statistic. The PCA analyses summarizes the variation of π, ρ, and Tajima's *D* among 12 *Castanopsis* species (A-C), as well as the variation of F_{ST} and d_{XY} across 66 species pairs (D-E) and across 11 representative contrasts derived using a phylogenetic correction approach (F-G). Summary statistics were calculated within non-overlapping windows of 10 kb (left panel), 100 kb (middle panel), and 500 kb (right panel). Each dot denotes a sliding window. The percentages of variance explained by the first two principal components (PC1 and PC2) are indicated in parentheses.

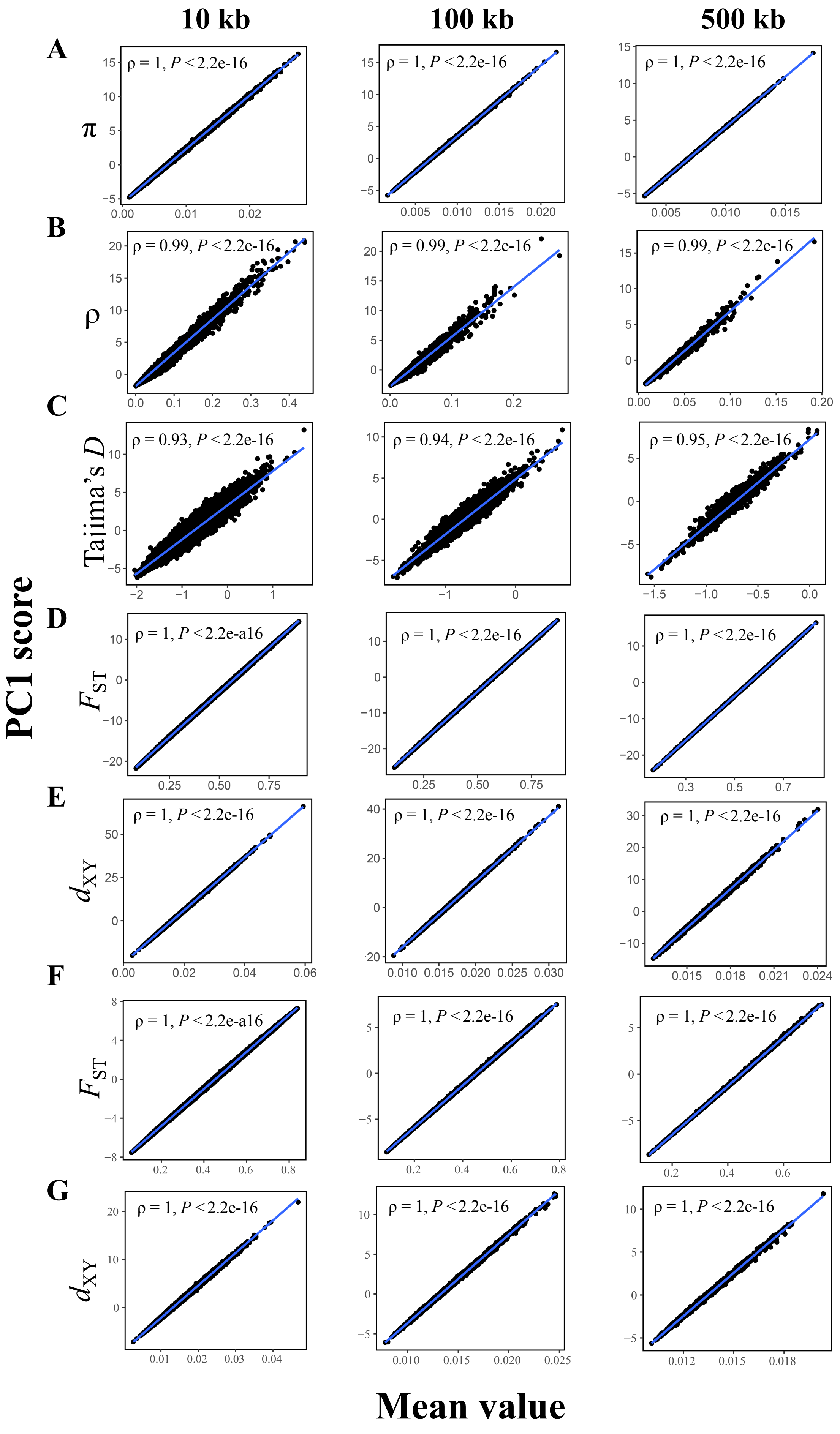
10 kb
100 kb
500 kb
A
B
C
D
E
F
G
π
ρ
Tajima's D
F_ST
d_XY
F_ST
d_XY
ρ = 1, P < 2.2e-16
ρ = 0.99, P < 2.2e-16
ρ = 0.93, P < 2.2e-16
ρ = 0.94, P < 2.2e-16
ρ = 0.95, P < 2.2e-16
ρ = 1, P < 2.2e-a16
PC1 score
Mean value

Fig. S14. Positive correlations between first principal component (PC1) scores and mean values of summary statistics. The PC1 score and mean value was calculated across 12 species for π, ρ, and Tajima's *D* (A-C). The PC1 score and mean value for F_{ST} and d_{XY} was determined across across 66 species-pairs (D-E), or 11 representative contrasts derived using a phylogenetic correction approach (F-G). All summary statistics were calculated within non-overlapping windows of 10 kb (left panel), 100 kb (middle panel), and 500 kb (right panel). In each plot, black dots denote sliding windows, while the blue line indicates the linear regression (lm) fit. Spearman's correlation coefficient values and their significance levels are indicated; all tests were significant ($P < 2.2e^{-16}$).

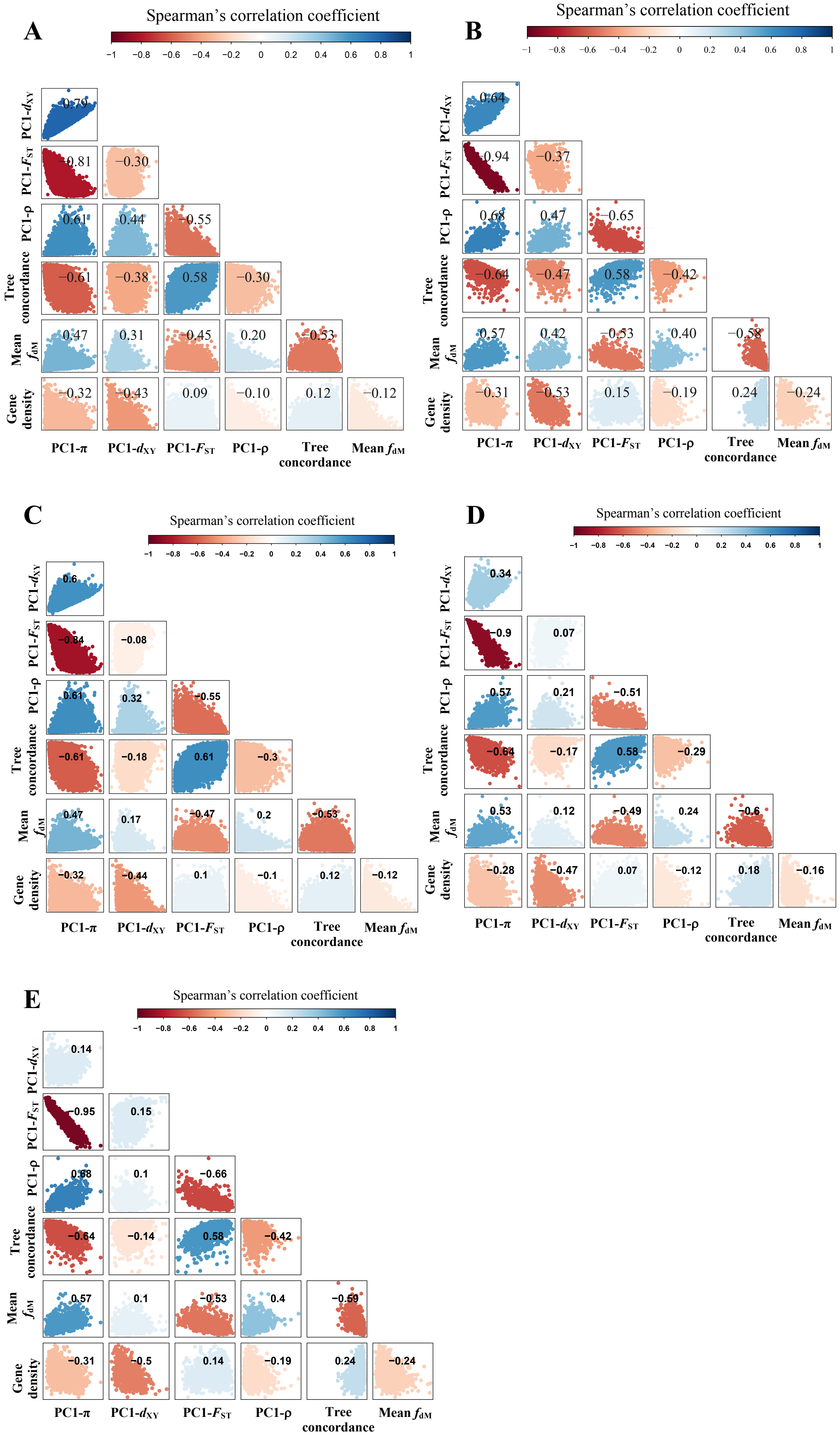

A
Spearman's correlation coefficient
−1 −0.8 −0.6 −0.4 −0.2 0 0.2 0.4 0.6 0.8 1
PC1-d_{XY} 0.79
PC1-F_{ST} −0.81 −0.30
PC1-ρ 0.61 0.44 −0.55
Tree concordance −0.61 −0.38 0.58 −0.30
Mean f_{dM} 0.47 0.31 −0.45 0.20 −0.53
Gene density −0.32 −0.43 0.09 −0.10 0.12 −0.12
PC1-π PC1-d_{XY} PC1-F_{ST} PC1-ρ Tree concordance Mean f_{dM}
B
Spearman's correlation coefficient
−1 −0.8 −0.6 −0.4 −0.2 0 0.2 0.4 0.6 0.8 1
PC1-d_{XY} 0.64
PC1-F_{ST} −0.94 −0.37
PC1-ρ 0.68 0.47 −0.65
Tree concordance −0.64 −0.47 0.58 −0.42
Mean f_{dM} 0.57 0.42 −0.53 0.40 −0.58
Gene density −0.31 −0.53 0.15 −0.19 0.24 −0.24
PC1-π PC1-d_{XY} PC1-F_{ST} PC1-ρ Tree concordance Mean f_{dM}
C
Spearman's correlation coefficient
−1 −0.8 −0.6 −0.4 −0.2 0 0.2 0.4 0.6 0.8 1
PC1-d_{XY} 0.6
PC1-F_{ST} −0.84 −0.08
PC1-ρ 0.61 0.32 −0.55
Tree concordance −0.61 −0.18 0.61 −0.3
Mean f_{dM} 0.47 0.17 −0.47 0.2 −0.53
Gene density −0.32 −0.44 0.1 −0.1 0.12 −0.12
PC1-π PC1-d_{XY} PC1-F_{ST} PC1-ρ Tree concordance Mean f_{dM}
D
Spearman's correlation coefficient
−1 −0.8 −0.6 −0.4 −0.2 0 0.2 0.4 0.6 0.8 1
PC1-d_{XY} 0.34
PC1-F_{ST} −0.9 0.07
PC1-ρ 0.57 0.21 −0.51
Tree concordance −0.64 −0.17 0.58 −0.29
Mean f_{dM} 0.53 0.12 −0.49 0.24 −0.6
Gene density −0.28 −0.47 0.07 −0.12 0.18 −0.16
PC1-π PC1-d_{XY} PC1-F_{ST} PC1-ρ Tree concordance Mean f_{dM}
E
Spearman's correlation coefficient
−1 −0.8 −0.6 −0.4 −0.2 0 0.2 0.4 0.6 0.8 1
PC1-d_{XY} 0.14
PC1-F_{ST} −0.95 0.15
PC1-ρ 0.68 0.1 −0.66
Tree concordance −0.64 −0.14 0.58 −0.42
Mean f_{dM} 0.57 0.1 −0.53 0.4 −0.59
Gene density −0.31 −0.5 0.14 −0.19 0.24 −0.24
PC1-π PC1-d_{XY} PC1-F_{ST} PC1-ρ Tree concordance Mean f_{dM}

Fig. S15. Significant correlations between population genetic parameters and genomic features in *Castanopsis* species. Principal component analysis (PCA) was employed to summarize the variation in each summary statistic. The first principal component (PC1) score was calculated across 12 species for π, ρ, and Tajima's *D*. The PC1 score for F_{ST} and d_{XY} was determined across 11 representative contrasts (A, B) derived using a phylogenetic correction approach, or across 66 species-pairs (C-E). PC1-π, PC1-ρ, PC1-F_{ST}, and PC1-d_{XY} refer to PC1 scores of π, ρ, F_{ST}, and d_{XY}, respectively. Spearman's correlation coefficients were calculated for comparisons between PC1-π , PC1-d_{XY}, PC1-F_{ST}, PC1-ρ, tree concordance, averaged f_{dM}, and gene density in non-overlapping windows of 10 kb (A, C), 100 kb (D), and 500 kb (B, E). Heatmap illustrating correlation strength and direction. All correlations were statistically significant ($P < 0.001$, Spearman's correlation test).

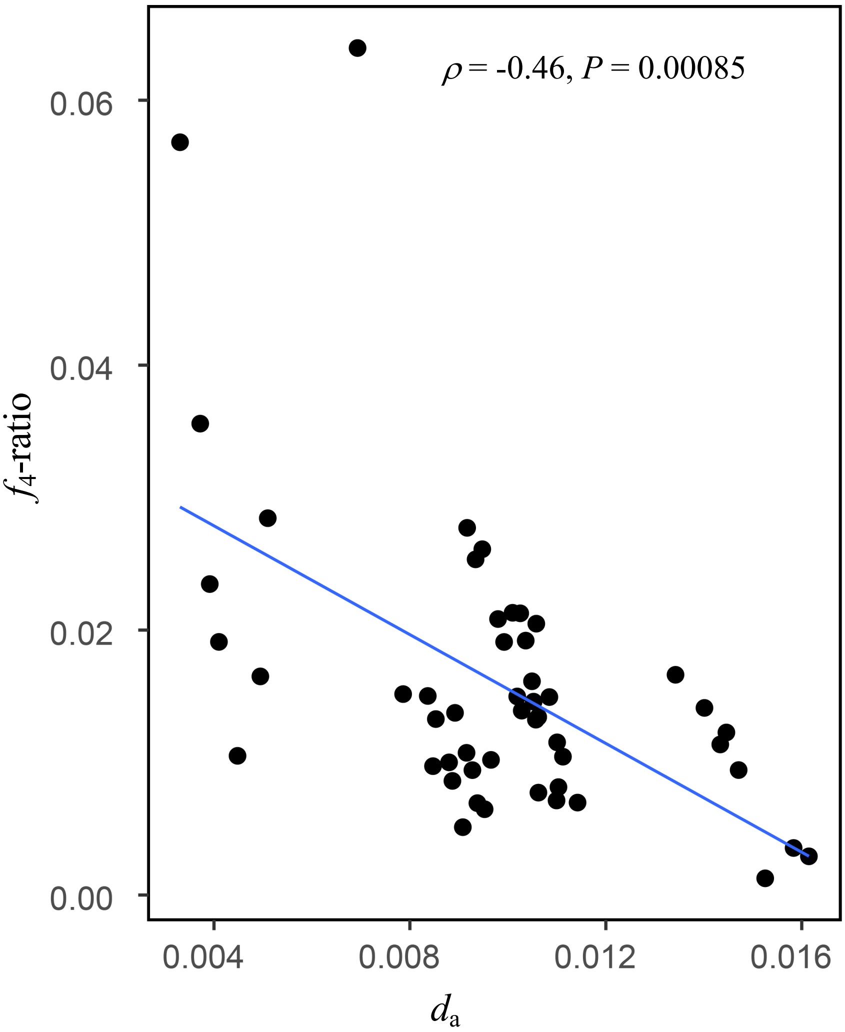

Fig. S16. Negative correlation between the proportion of admixture (f_4-ratio) and net nucleotide divergence (d_a). Spearman's correlation coefficient (ρ = –0.46, P = 0.00085) between f_4-ratio and d_a was determined using 51 trios. Each black dot denotes a trio, with the blue line indicating the linear regression (lm) fit.

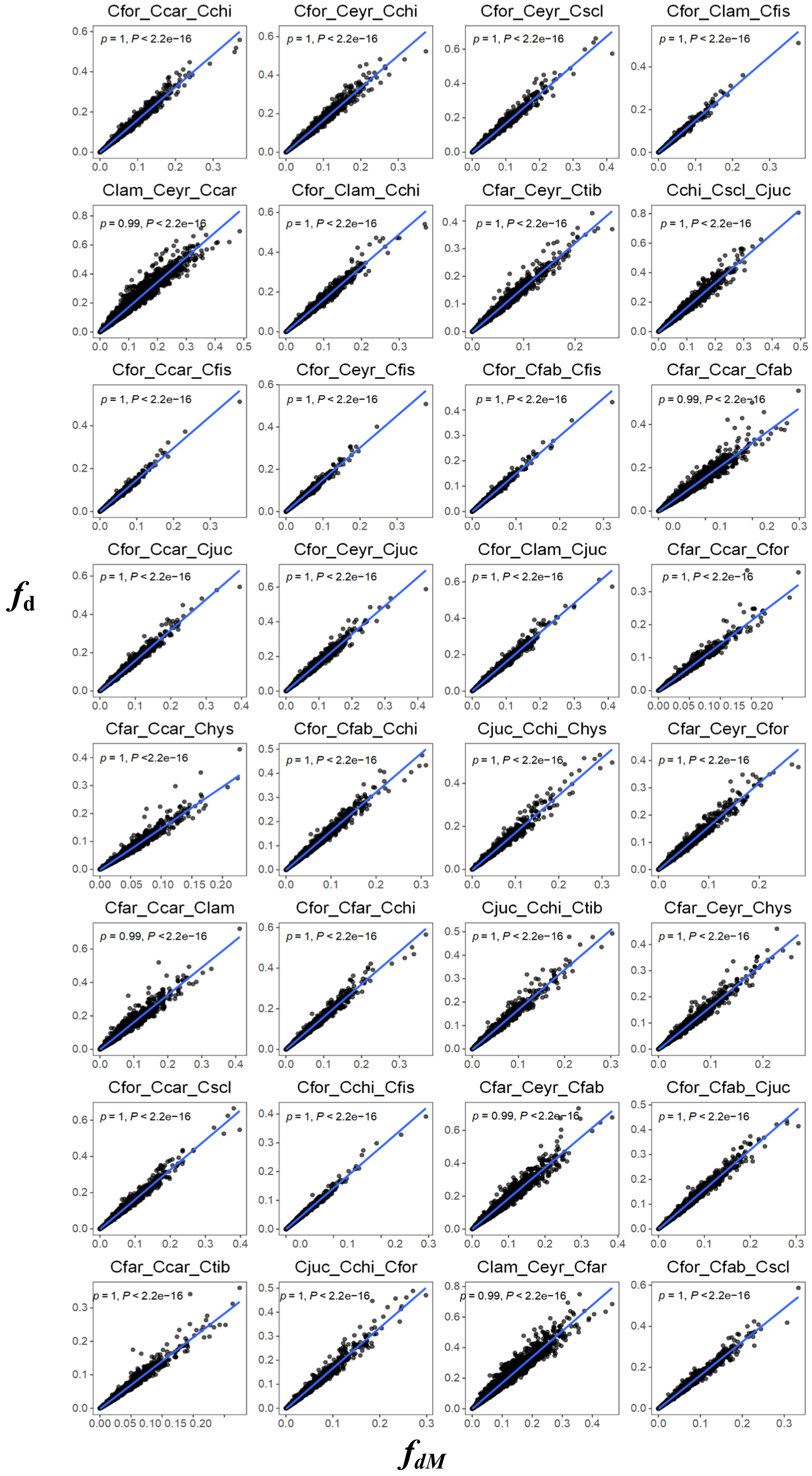
Cfor_Ccar_Cchi
Cfor_Ceyr_Cchi
Cfor_Ceyr_Cscl
Cfor_Clam_Cfis
Clam_Ceyr_Ccar
Cfor_Clam_Cchi
Cfar_Ceyr_Ctib
Cchi_Cscl_Cjuc
Cfor_Ccar_Cfis
Cfor_Ceyr_Cfis
Cfor_Cfab_Cfis
Cfar_Ccar_Cfab
Cfor_Ccar_Cjuc
Cfor_Ceyr_Cjuc
Cfor_Clam_Cjuc
Cfar_Ccar_Cfor
Cfar_Ccar_Chys
Cfor_Cfab_Cchi
Cjuc_Cchi_Chys
Cfar_Ceyr_Cfor
Cfar_Ccar_Clam
Cfor_Cfar_Cchi
Cjuc_Cchi_Ctib
Cfar_Ceyr_Chys
Cfor_Ccar_Cscl
Cfor_Cchi_Cfis
Cfar_Ceyr_Cfab
Cfor_Cfab_Cjuc
Cfar_Ccar_Ctib
Cjuc_Cchi_Cfor
Clam_Ceyr_Cfar
Cfor_Cfab_Cscl
$\rho = 1, P < 2.2e{-16}$
$\rho = 0.99, P < 2.2e{-16}$
f_d
f_{dM}

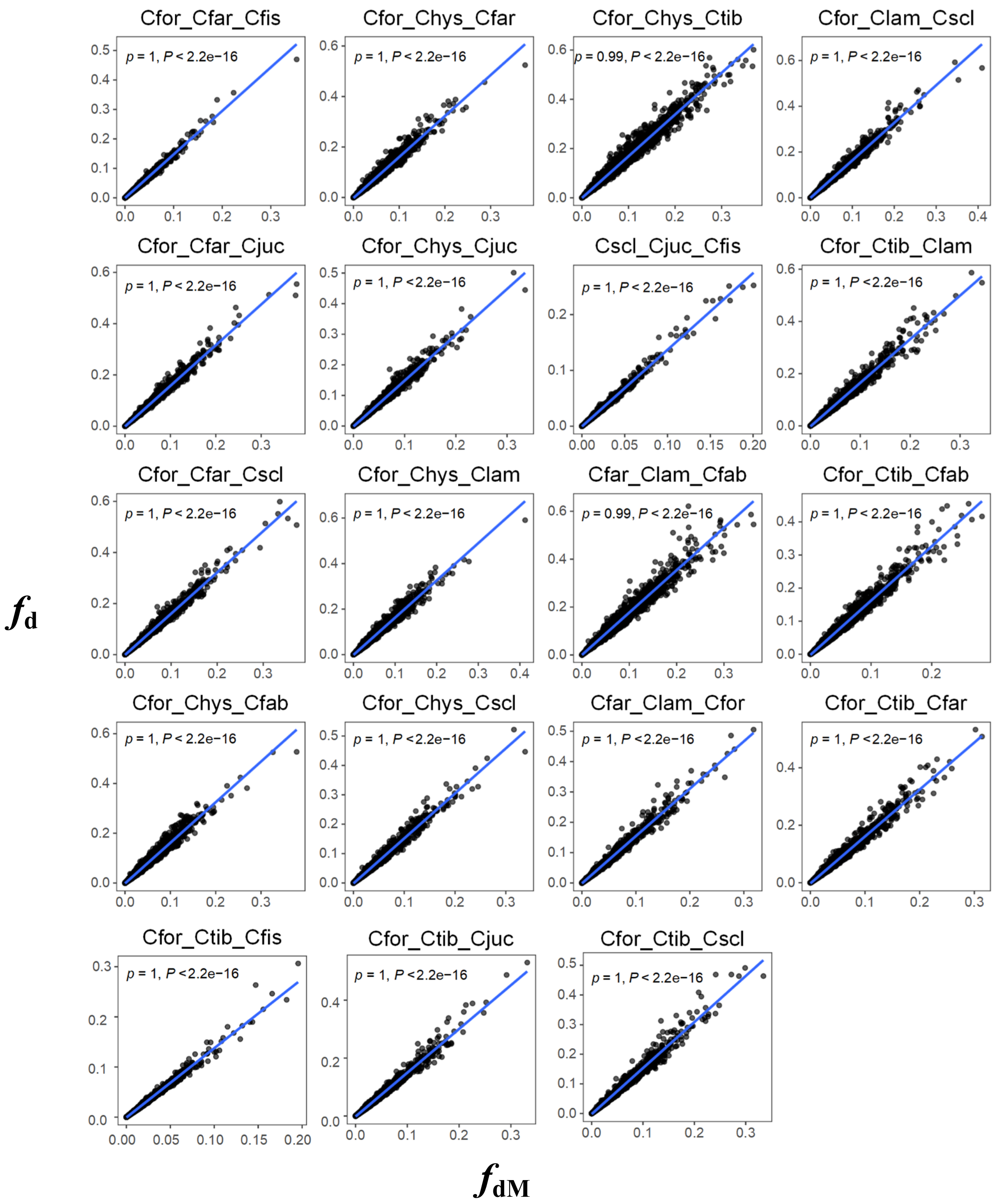

Fig. S17. Positive correlation between f_d and f_{dM} estimated for 51 trios. In each plot, black dots denote 100 kb non-overlapping windows, and the blue line indicates the linear regression (lm) fit. All tests showed significant results ($P < 2.2e^{-16}$, Spearman's correlation test). For each trio, the three ingroup species are represented as P1_P2_P3; for example, Cfor_Cfar_Cfis indicates a trio of (Cfor, Cfar), Cfis). Cfar, *C. fargesii*; Ccar, *C. carlesii*; Ceyr, *C. eyrei*; Clam, *C. lamontii*; Cfab, *C. fabri*; Chys, *C. hystrix*; Cfor, *C. fordii*; Ctib, *C. tibetana*; Cchi, *C. chinensis*; Cscl, *C. sclerophylla*; Cjuc, *C. jucunda*; Cfis, *C. fissa*. Values of fd and f_{dM}, estimated in 10 and 500 kb non-overlapping windows were also positively correlated (Spearman's $\rho = 0.97–1.00$, $P < 2.2e^{-16}$; data not shown).

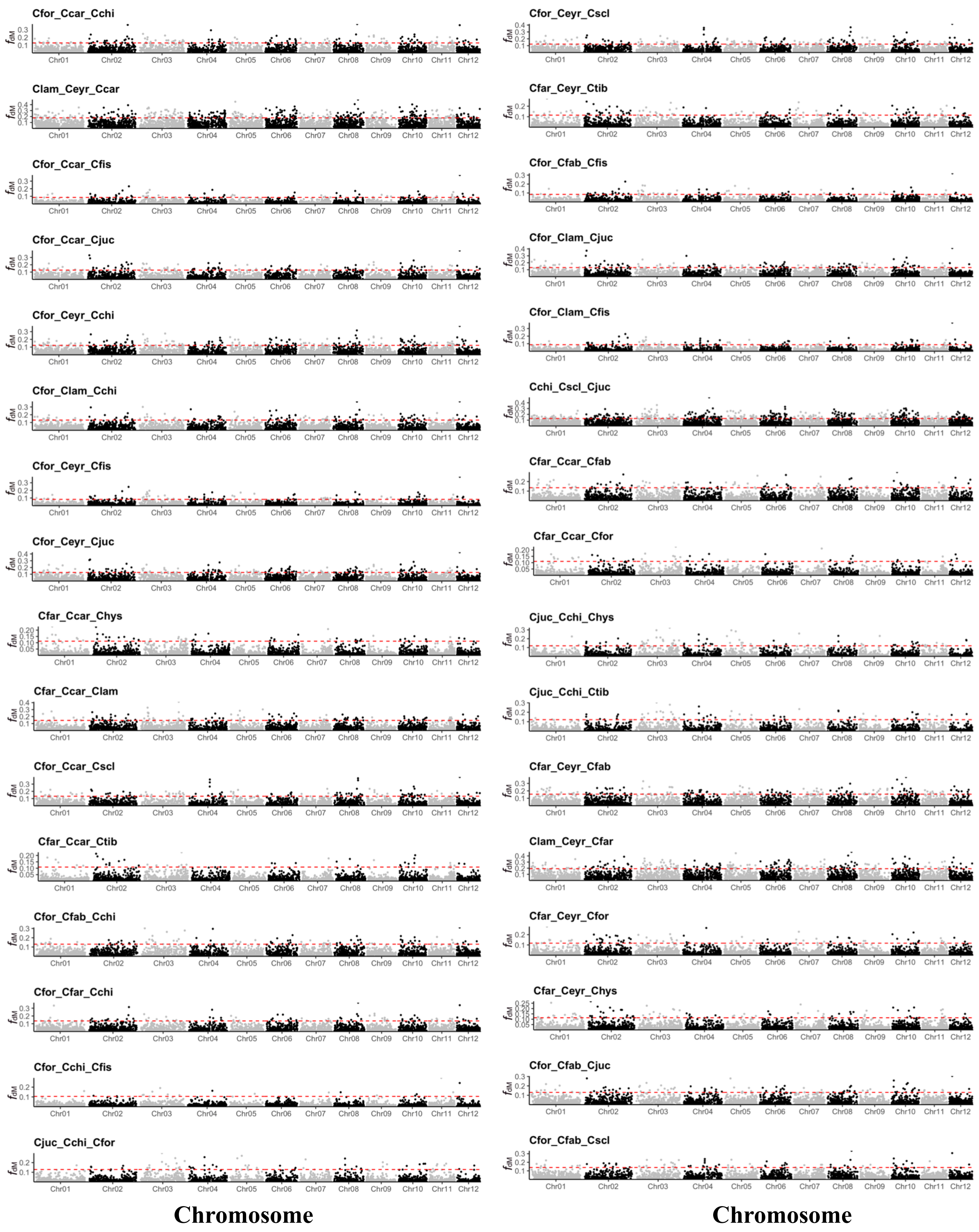
Cfor_Ccar_Cchi
Clam_Ceyr_Ccar
Cfor_Ccar_Cfis
Cfor_Ccar_Cjuc
Cfor_Ceyr_Cchi
Cfor_Clam_Cchi
Cfor_Ceyr_Cfis
Cfor_Ceyr_Cjuc
Cfar_Ccar_Chys
Cfar_Ccar_Clam
Cfor_Ccar_Cscl
Cfar_Ccar_Ctib
Cfor_Cfab_Cchi
Cfor_Cfar_Cchi
Cfor_Cchi_Cfis
Cjuc_Cchi_Cfor
Cfor_Ceyr_Cscl
Cfar_Ceyr_Ctib
Cfor_Cfab_Cfis
Cfor_Clam_Cjuc
Cfor_Clam_Cfis
Cchi_Cscl_Cjuc
Cfar_Ccar_Cfab
Cfar_Ccar_Cfor
Cjuc_Cchi_Chys
Cjuc_Cchi_Ctib
Cfar_Ceyr_Cfab
Clam_Ceyr_Cfar
Cfar_Ceyr_Cfor
Cfar_Ceyr_Chys
Cfor_Cfab_Cjuc
Cfor_Cfab_Cscl
f_{dM}
Chr01 Chr02 Chr03 Chr04 Chr05 Chr06 Chr07 Chr08 Chr09 Chr10 Chr11 Chr12
Chromosome
Chromosome

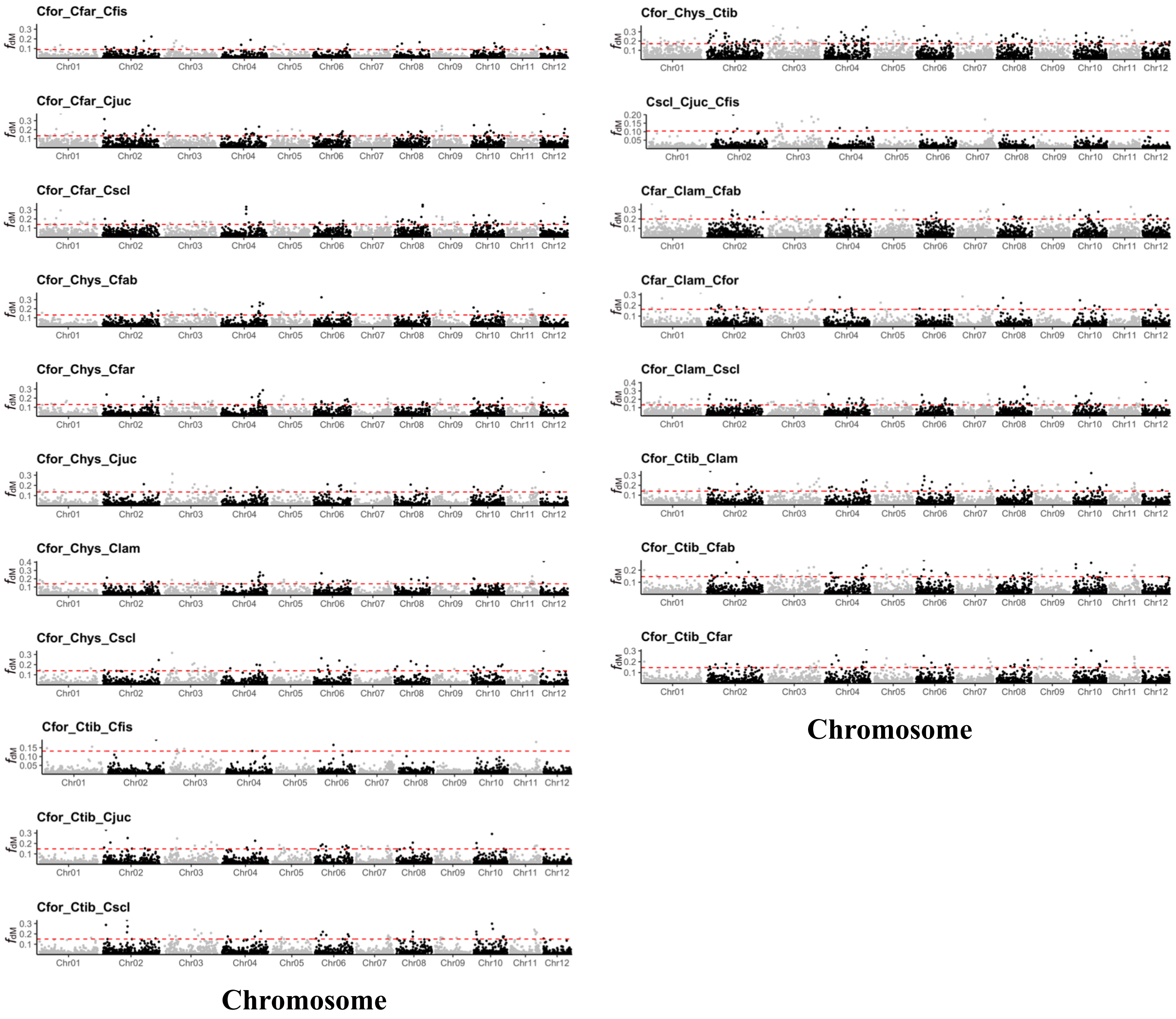

Fig. S18. Manhattan plot illustrating the genome-wide patterns of introgression for 51 *Castanopsis* species trios. In each plot, black dots denote 100 kb non-overlapping windows, and the red line indicates the threshold used to identify introgression windows. For each trio, the three ingroup species are represented as P1_P2_P3; for example, Cfor_Cfar_Cfis indicates a trio of (Cfor, Cfar), Cfis). Cfar, *C. fargesii*; Ccar, *C. carlesii*; Ceyr, *C. eyrei*; Clam, *C. lamontii*; Cfab, *C. fabri*; Chys, *C. hystrix*; Cfor, *C. fordii*; Ctib, *C. tibetana*; Cchi, *C. chinensis*; Cscl, *C. sclerophylla*; Cjuc, *C. jucunda*; Cfis, *C. fissa*.

A

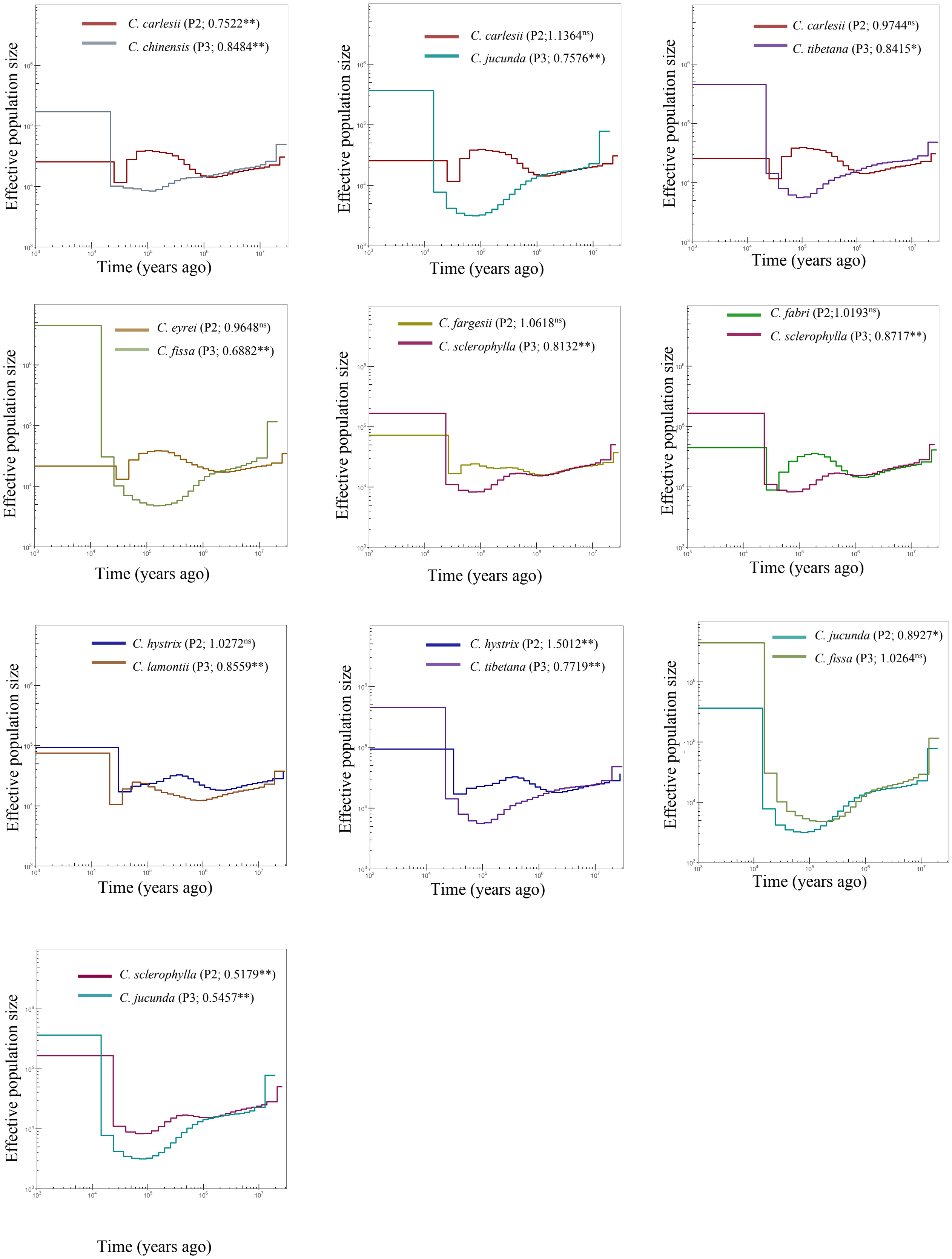

C. carlesii (P2; 0.7522**)
C. chinensis (P3; 0.8484**)
Effective population size
Time (years ago)
C. carlesii (P2;1.1364ns)
C. jucunda (P3; 0.7576**)
Effective population size
Time (years ago)
C. carlesii (P2; 0.9744ns)
C. tibetana (P3; 0.8415*)
Effective population size
Time (years ago)
C. eyrei (P2; 0.9648ns)
C. fissa (P3; 0.6882**)
Effective population size
Time (years ago)
C. fargesii (P2; 1.0618ns)
C. sclerophylla (P3; 0.8132**)
Effective population size
Time (years ago)
C. fabri (P2;1.0193ns)
C. sclerophylla (P3; 0.8717**)
Effective population size
Time (years ago)
C. hystrix (P2; 1.0272ns)
C. lamontii (P3; 0.8559**)
Effective population size
Time (years ago)
C. hystrix (P2; 1.5012**)
C. tibetana (P3; 0.7719**)
Effective population size
Time (years ago)
C. jucunda (P2; 0.8927*)
C. fissa (P3; 1.0264ns)
Effective population size
Time (years ago)
C. sclerophylla (P2; 0.5179**)
C. jucunda (P3; 0.5457**)
Effective population size
Time (years ago)

B

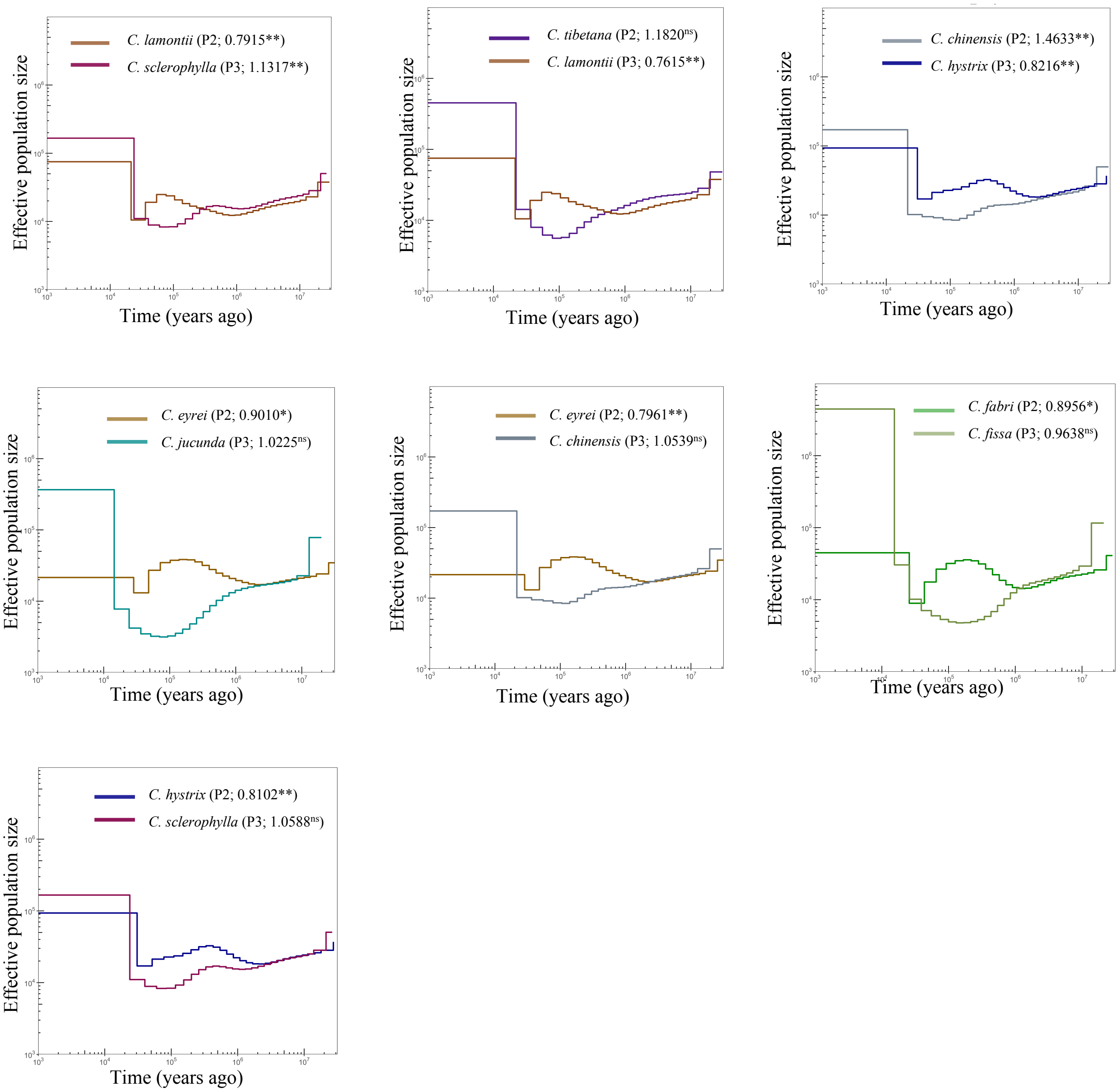
C. lamontii (P2; 0.7915**)
C. sclerophylla (P3; 1.1317**)
Effective population size
Time (years ago)
C. tibetana (P2; 1.1820^ns)
C. lamontii (P3; 0.7615**)
Effective population size
Time (years ago)
C. chinensis (P2; 1.4633**)
C. hystrix (P3; 0.8216**)
Effective population size
Time (years ago)
C. eyrei (P2; 0.9010*)
C. jucunda (P3; 1.0225^ns)
Effective population size
Time (years ago)
C. eyrei (P2; 0.7961**)
C. chinensis (P3; 1.0539^ns)
Effective population size
Time (years ago)
C. fabri (P2; 0.8956*)
C. fissa (P3; 0.9638^ns)
Effective population size
Time (years ago)
C. hystrix (P2; 0.8102**)
C. sclerophylla (P3; 1.0588^ns)
Effective population size
Time (years ago)

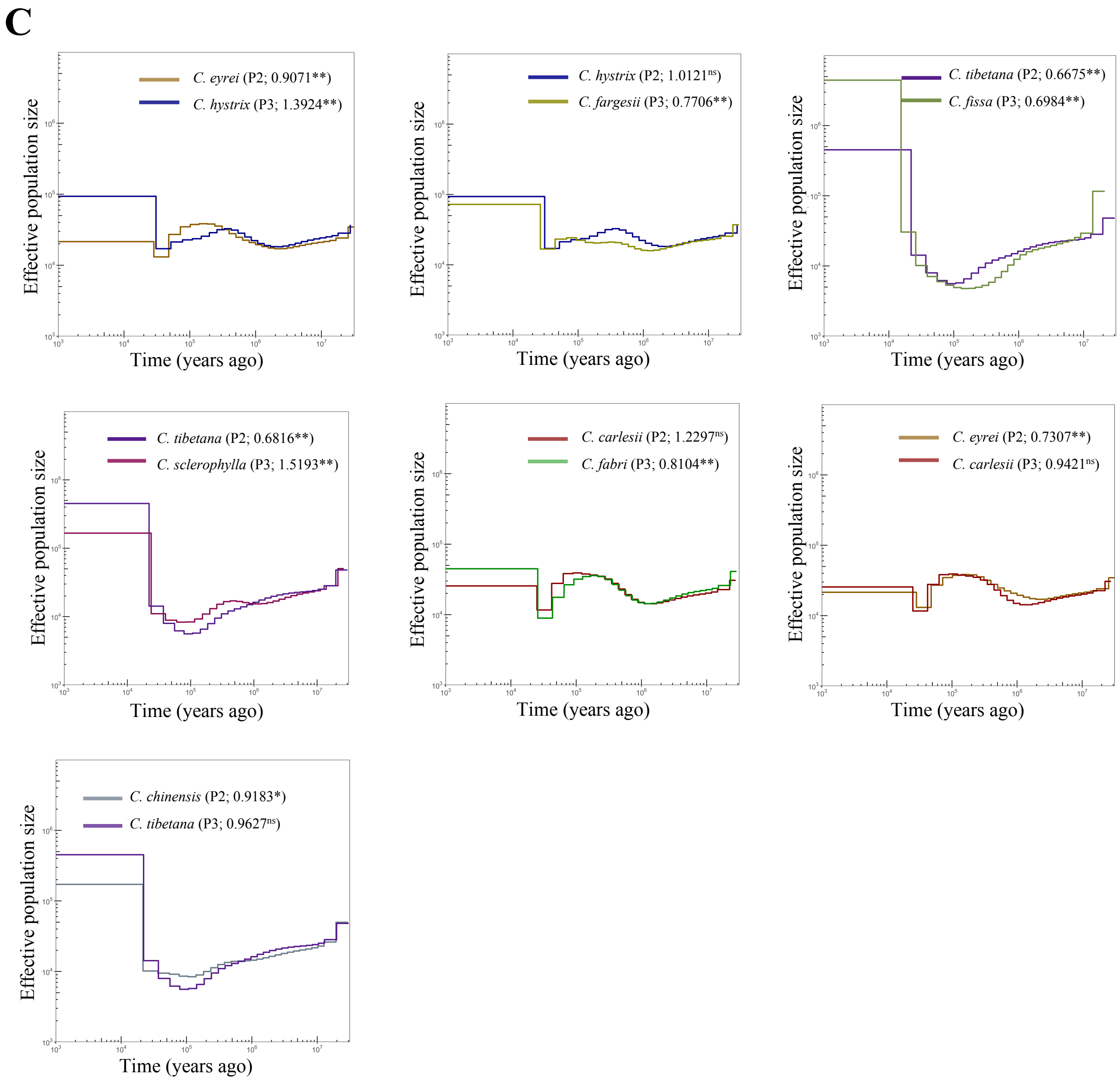
C
C. eyrei (P2; 0.9071**)
C. hystrix (P3; 1.3924**)
C. hystrix (P2; 1.0121^{ns})
C. fargesii (P3; 0.7706**)
C. tibetana (P2; 0.6675**)
C. fissa (P3; 0.6984**)
C. tibetana (P2; 0.6816**)
C. sclerophylla (P3; 1.5193**)
C. carlesii (P2; 1.2297^{ns})
C. fabri (P3; 0.8104**)
C. eyrei (P2; 0.7307**)
C. carlesii (P3; 0.9421^{ns})
C. chinensis (P2; 0.9183*)
C. tibetana (P3; 0.9627^{ns})
Effective population size
Time (years ago)

Fig. S19. Impact of effective population size (N_e) on the burden of deleterious mutations in introgression regions. Changes in N_e of P2 and P3 species were compared across 24 trios, each of which showed a reduced genetic burden in the introgression regions of at least one species. (A) Ten trios show a reduced genetic burden in species with a lower N_e. (B) Seven trios exhibit a reduced genetic burden in species with a larger N_e. (C) Seven trios show a reduced genetic burden in either the P2 or P3 species, yet there is no significant difference in N_e between these two species. In each plot, the N_e changes are derived from Fig. S7. The recessive genetic load within introgression regions was calculated relative to the overall genomic background for each species (see Table S10). Statistical significance: *$P < 0.05$, **$P < 0.01$, ***$P < 0.001$, ^{ns}(not significant).

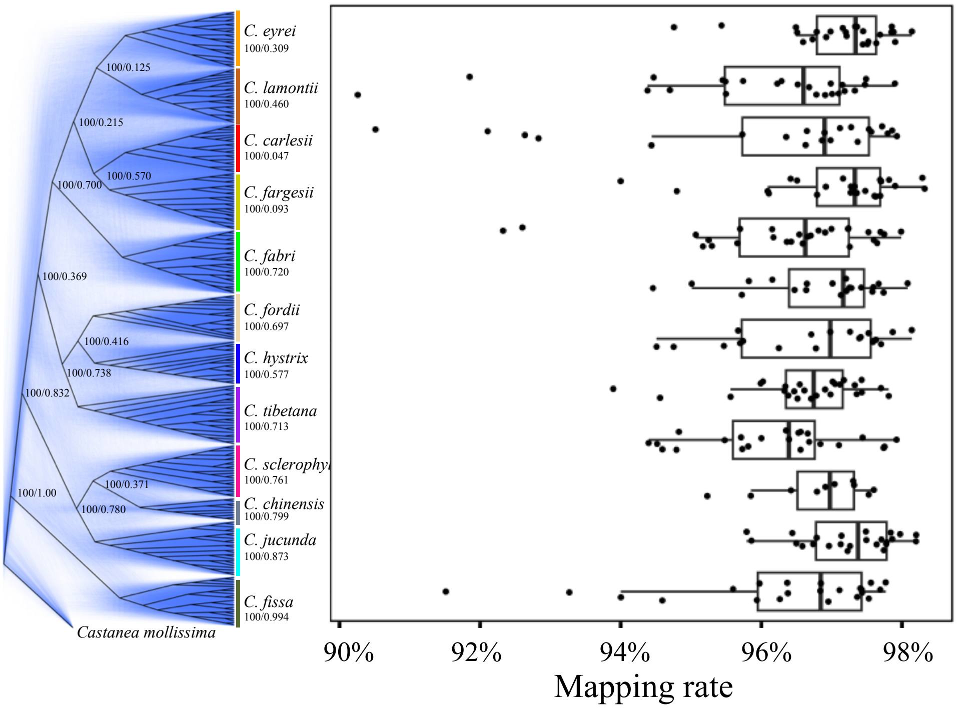

Fig. S20. Mapping rates obtained by aligning re-sequencing data of *Castanopsis* species to the *C. eyrei* reference genome. All individuals exhibited high mapping rates (90.26% – 98.32%), with relatively low mapping rates appearing to be individual-specific rather than species-specific, suggesting no species-specific bias due to divergence from the reference. Left panel: phylogenetic relationships of 12 *Castanopsis* species as presented in Fig. 1. The black tree was constructed based on genome-wide SNPs, while the blue trees represent 100 kb window-based trees. For each node or species, the first number indicates the bootstrap value in the whole-genome tree, and the second number reflects the proportion of window-based trees supporting that clade. Right panel: boxplot of mapping rates for each species. In each plot, each black dot represents an individual, and the horizontal line indicates the median, with the bottom and top of the boxes representing the first and third quartiles, respectively. Whiskers extend to 1.5 times the interquartile range.
